# ﻿*Austrelatus* gen. nov., a new genus of Australasian diving beetles (Coleoptera, Dytiscidae, Copelatinae), with the discovery of 31 new species from New Guinea

**DOI:** 10.3897/zookeys.1170.103834

**Published:** 2023-07-19

**Authors:** Helena Shaverdo, Jiří Hájek, Lars Hendrich, Suriani Surbakti, Rawati Panjaitan, Michael Balke

**Affiliations:** 1 Naturhistorisches Museum Wien, Burgring 7, 1010, Vienna, Austria Naturhistorisches Museum Wien Vienna Austria; 2 Department of Entomology, National Museum, Cirkusová 1740, CZ-193 00 Praha 9 – Horní Počernice, Czech Republic National Museum Prague Czech Republic; 3 SNSB-Zoologische Staatssammlung München, Münchhausenstraße 21, D-81247, Munich, Germany SNSB-Zoologische Staatssammlung München Munich Germany; 4 GeoBioCenter, Ludwig-Maximilians-University, Munich, Germany Ludwig-Maximilians-University Munich Germany; 5 Department of Biology, Universitas Cendrawasih, Jayapura, Papua, Indonesia Universitas Cendrawasih Jayapura Indonesia; 6 Department of Biology, Faculty of Sciences and Mathematics, State University of Papua (UNIPA), Jalan Gunung Salju Amban, Manokwari 98314, West Papua, Indonesia State University of Papua Manokwari Indonesia

**Keywords:** Australasia, lectotype designation, new combination, New Guinea, new species, species group, taxonomy

## Abstract

Herein, *Austrelatus***gen. nov.** (type species: *Copelatusirregularis* W.J. Macleay, 1871) is described for a distinctive lineage of predominantly Australasian species previously assigned to *Copelatus* Erichson, 1832. The new genus was retrieved as well supported, monophyletic clade in phylogenetic analysis of DNA sequences data using Bayesian and parsimony approaches. The main morphological diagnostic character of *Austrelatus* is a complex median lobe of the aedeagus, with evident dorsal and ventral sclerites usually divided in apical half into two lobes of different shape or otherwise modified. Morphological comparison of the new genus with other Copelatinae genera, especially with *Copelatus* and *Exocelina* Broun, 1886, and a generic key to the New Guinean Copelatinae are provided. New combinations are established for 31 already described species mainly from the Australian Region (all from *Copelatus*): *Austrelatusadelbert* (Megna, Atthakor, Manaono, Hendrich & Balke, 2017), **comb. nov.**; *A.badeni* (Sharp, 1882), **comb. nov.**; *A.bakewelli* (J. Balfour-Browne, 1939), **comb. nov.**; *A.baranensis* (Hájek, Shaverdo, Hendrich & Balke, 2021), **comb. nov.**; *A.bougainvillensis* (Hájek, Shaverdo, Hendrich & Balke, 2021), **comb. nov.**; *A.boukali* (Hendrich & Balke, 1998), **comb. nov.**; *A.clarki* (Sharp, 1882), **comb. nov.**; *A.daemeli* (Sharp, 1882), **comb. nov.**; *A.davidi* (Wewalka, 2017), **comb. nov.**; *A.deccanensis* (Sheth, Ghate & Hájek, 2018), **comb. nov.**; *A.fidschiensis* (Zimmermann, 1928), **comb. nov.**; *A.gestroi* (Régimbart, 1892), **comb. nov.**; *A.irregularis* (W.J. Macleay, 1871), **comb. nov.**; *A.kaszabi* (Guignot, 1956), **comb. nov.**; *A.kietensis* (Hájek, Shaverdo, Hendrich & Balke, 2021), **comb. nov.**; *A.laevipennis* (Hájek, Shaverdo, Hendrich & Balke, 2021), **comb. nov.**; *A.luteomaculatus* (Guignot, 1956), **comb. nov.**; *A.maushomi* (Sheth, Ghate & Hájek, 2018), **comb. nov.**; *A.neoguineensis* (Zimmermann, 1919), **comb. nov.**; *A.nigrolineatus* (Sharp, 1882), **comb. nov.**; *A.papuensis* (J. Balfour-Browne, 1939), **comb. nov.**; *A.parallelus* (Zimmermann, 1920a), **comb. nov.**; *A.schuhi* (Hendrich & Balke, 1998), **comb. nov.**; *A.sibelaemontis* (Hájek, Hendrich, Hawlitschek & Balke, 2010), **comb. nov.**; *A.strigosulus* (Fairmaire, 1878), **comb. nov.**; *A.ternatensis* (Régimbart, 1899), **comb. nov.**; *A.uludanuensis* (Hendrich & Balke, 1995), **comb. nov.**; *A.urceolus* (Hájek, Shaverdo, Hendrich & Balke, 2021), **comb. nov.**; *A.variistriatus* (Hájek, Shaverdo, Hendrich & Balke, 2021), **comb. nov.**; *A.wallacei* (J. Balfour-Browne, 1939), **comb. nov.** and *A.xanthocephalus* (Régimbart, 1899), **comb. nov.***Austrelatus* species from New Guinea are divided into two informal species groups, the *A.neoguineensis* group and *A.papuensis* group, and *A.fumato***sp. nov.** and *A.setiphallus***sp. nov.** standing aside of them. The *A.neoguineensis* group is introduced with three previously known species and 29 new species described here based on the morphological characters and Cox1 data: *Austrelatusbaliem***sp. nov.**, *A.bormensis***sp. nov.**, *A.brazza***sp. nov.**, *A.debulensis***sp. nov.**, *A.fakfak***sp. nov.**, *A.febrisauri***sp. nov.**, *A.fojaensis***sp. nov.**, *A.garainensis***sp. nov.**, *A.innominatus***sp. nov.**, *A.lembenensis***sp. nov.**, *A.lisae***sp. nov.**, *A.manokwariensis***sp. nov.**, *A.mimika***sp. nov.**, *A.mirificus***sp. nov.**, *A.moreguinensis***sp. nov.**, *A.nadjae***sp. nov.**, *A.oksibilensis***sp. nov.**, *A.pseudoneoguineensis***sp. nov.**, *A.pseudoksibilensis***sp. nov.**, *A.rajaampatensis***sp. nov.**, *A.rouaffer***sp. nov.**, *A.rugosus***sp. nov.**, *A.sandaunensis***sp. nov.**, *A.sarmiensis***sp. nov.**, *A.securiformis***sp. nov.**, *A.testegensis***sp. nov.**, *A.toricelli***sp. nov.**, *A.vagauensis***sp. nov.**, and *A.wanggarensis***sp. nov.***Copelatusvagestriatus* Zimmermann, 1919, **syn. nov.** is recognised as a junior subjective synonym of *A.clarki* (Sharp, 1882). The lectotypes of *Copelatusgestroi* Régimbart, 1892, *C.neoguineensis* Zimmermann, 1919 and *C.xanthocephalus* Régimbart, 1899 are designated. All species are (re)described, and their important species characters (genitalia, habitus, and colour patterns) are illustrated. Keys to all species are provided. The known distribution and habitat preferences of each species are outlined briefly. New Guinean *Austrelatus* occupy a variety of stagnant water habitats, either lentic *sensu stricto*, or standing water associated with lotic habitats (e.g., backflows, rockpools, intermittent / ephemeral stream pools).

## ﻿Introduction

Copelatinae, the second largest subfamily of Dytiscidae, has been so far represented in Australasia by two genera, *Copelatus* Erichson, 1832 and *Exocelina* Broun, 1886, and the majority of species occurs in New Guinea. One hundred and fifty-two species of New Guinean *Exocelina* were treated in our previous publications, the results being summarised by Shaverdo and Balke (2022). *Copelatus*, the most speciose diving beetle group, is known in New Guinea only from 17 described species (Régimbart 1899; Zimmermann 1919; Balfour-Browne 1939, 1945; Guignot 1942, 1956; Guéorguiev 1968, 1978; Guéorguiev and Rocchi 1993; Shaverdo et al. 2008; Megna et al. 2017; Hendrich et al. 2019; Nilsson and Hájek 2023). However, more than 80 undescribed species of Australasian Copelatinae have been discovered during a taxonomic research initiative, which so far resulted in the revisions of the species from Australia and Solomon Islands including descriptions of seven new species (Hendrich et al. 2019; Hájek et al. 2021). Most of the new species, however, are from New Guinea and Fiji. Our study of morphology (especially structure of the male genitalia) of the already described and new species and an unpublished molecular phylogenetic analysis (Toussaint, Villastrigo, Shaverdo, Balke et al., unpublished data) showed their distinct heterogeneity and led to the establishment of a new genus, *Austrelatus* gen. nov. It is introduced here for 31 previously described species (as *Copelatus*) and 31 new species from New Guinea treated in this paper.

## ﻿Materials and methods

### ﻿Material

The present work is based on material from the following collections:

**BMNH** The Natural History Museum [formerly British Museum (Natural History)], London, UK;

**CAS** Collection of Anders Skale, Gera, Germany;

**CLH** Collection of Lars Hendrich, Munich, Germany (property of NHMW);

**CMPK** Collection of Michael P. Kowalski, Charlotte, USA;

**HNHM**Hungarian Natural History Museum, Budapest, Hungary;

**KSP** Kelompok Serranga Papua Collection, UNCEN, Jayapura, Papua, Indonesia;

**MSNG**Museo di Storia Naturale Giacomo Doria, Genova, Italy;

**MNHN**Muséum national d´Histoire naturelle, Paris, France;

**MTD** Museum für Tierkunde, Dresden, Germany;

**MZB**Museum Zoologicum Bogoriense, Cibinong, West Java, Indonesia;

**NMPC**Národní muzeum, Prague, Czech Republic;

**NHMW**Naturhistorisches Museum Wien, Vienna, Austria;

**SDEI**Senckerberg Deutsches Entomologisches Institut, Müncheberg, Germany;

**SMNK**Staatliches Museum für Naturkunde Karlsruhe, Karlsruhe, Germany;

**SMNS**Staatliches Museum für Naturkunde, Stuttgart, Germany;

**ZSM**SNSB-Zoologische Staatssammlung München, Munich, Germany.

The extensive material, including types, of the following species treated in detail in our previous publications (Hendrich and Balke 1995; Hájek et al. 2010, 2021; Sheth et al. 2018; Hendrich et al. 2019; Jiang et al. 2022) was used: *Copelatusbakewelli* J. Balfour-Browne, 1939; *C.baranensis* Hájek, Shaverdo, Hendrich & Balke, 2021; *C.bougainvillensis* Hájek, Shaverdo, Hendrich & Balke, 2021; *C.daemeli* Sharp, 1882; *C.deccanensis* Sheth, Ghate & Hájek, 2018; *C.irregularis* W.J. Macleay, 1871; *C.kietensis* Hájek, Shaverdo, Hendrich & Balke, 2021; *C.laevipennis* Hájek, Shaverdo, Hendrich & Balke, 2021; *C.maushomi* Sheth, Ghate & Hájek, 2018; *C.nigrolineatus* Sharp, 1882; *C.parallelus* Zimmermann, 1920a; *C.schuhi* Hendrich & Balke, 1998; *C.sibelaemontis* Hájek, Hendrich, Hawlitschek & Balke, 2010; *C.uludanuensis* Hendrich & Balke, 1995; *C.urceolus* Hájek, Shaverdo, Hendrich & Balke, 2021; *C.variistriatus* Hájek, Shaverdo, Hendrich & Balke, 2021.

All specimen data are quoted as they appear on the labels attached to the specimens. Label text is cited using quotation marks; comments in square brackets are ours, including hw (handwritten) and pr (printed).

Administrative divisions of Indonesia and Papua New Guinea follow information from Wikipedia (2021a, b, c).

### ﻿Morphology

Specimens were examined and measured using a Leica M205 C stereomicroscope with an ocular graticule. For detailed study and illustration, male genitalia were removed and mounted on glass slides with DMHF (dimethyl hydantoin formaldehyde) or polyvinyl alcohol as temporary preparations. They were studied using a Leica DM 2500 microscope.

Habitus photographies were made by Harald Schillhammer (NHMW). Images were captured with a Nikon D4 (in combination with a Novoflex bellows and a Mitutoyo 5 and 10/0.25 Apo ELWD) tethered to a PC and controlled with Nikon Camera Control Pro. Resulting image stacks were treated with Zerene Stacker and then post-processed in Adobe Photoshop CS 5. Median lobes were photographed in dry condition by M. Balke and L. Hendrich with a Canon EOS R camera. They used a Mitutoyo 10× ELWD Plan Apo objective attached to a Carl Zeiss Jena Sonnar 3.5/135 MC. Illumination was with three LED segments SN-1 from Stonemaster (https://www.stonemaster-onlineshop.de). Image stacks were generated using the Stackmaster macro rail (Stonemaster), and images were assembled with Helicon Focus v. 7.61 on a MacPro 2019 with a Radeon Pro 6800X MPX GPU. Parameres and some median lobes were mounted on glass slides and photographed in wet condition by H. Shaverdo using a Nikon Eclipse 80i and treated with a NIS-Elements D 4.51.01 64-bit. For better demonstration of their sclerites, these median lobes were intensively treated with lactic acid. Due to that, their apical parts are much more separated from each other than in the freshly prepared genitalia; compare with ones photographed in dry condition for the original sclerite position. The taxon order of the morphological illustrations and maps follows the taxon order in both keys to make the identification and work with the paper more convenient.

The descriptive style generally follows Hendrich et al. (2019) but has some modifications based on Shaverdo et al. (2019) and for measurements on Megna and Epler (2012). The terminology to denote the orientation of the genitalia follows Miller and Nilsson (2003). The terminology of the structure of the prosternum follows Larson et al. (2000). Characters, given in the genus description and in species group diagnosis, are not repeated in the species descriptions. Numbers of the elytral striae are coded as e.g., (10–11)+(0–1) or 6+1 what is to understand as dorsal striae + submarginal stria.

The following abbreviations were used in the descriptions: **DBE** – minimum distance between eyes; **PL** – pronotal length (along midline from anterior to posterior margins); **PW** – pronotal width at level of posterior margin; **TL** – total length, measured from anterior margin of clypeus to apex of elytra; **TL-h** – total length minus head length, measured from anterior margin of pronotum to apex of elytra; **TW** – maximum width of body measured at right angle to TL.

### ﻿Molecular phylogenetics

Our unpublished molecular phylogenetic analysis (Toussaint, Villastrigo, Shaverdo, Balke et al., unpublished data) uses six gene fragments: the mitochondrial cox1, as well as the nuclear 18S rRNA, CAD, H3, wingless, and RNA polymerase I for 485 species of the Copelatinae worldwide. All the details of this study will be provided in the upcoming publication.

## ﻿Results

### ﻿*Copelatus* species in New Guinea

**Figures 1, 2.**
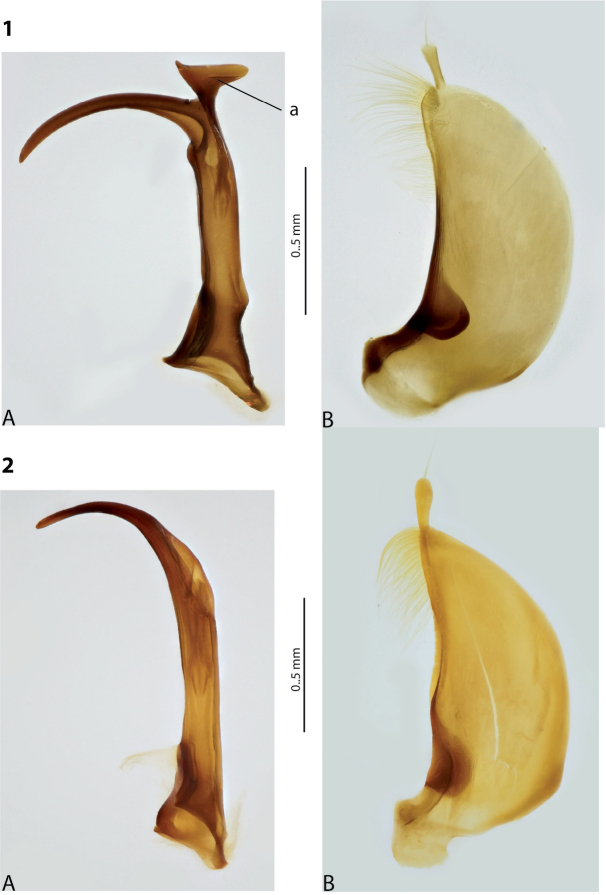
Median lobe in left lateral view **A** and left paramere in external view **B** of **1***Copelatusportior* Guignot, 1956 **2***C.martinbaehri*Hendrich et al., 2019; **a** median process.

Our study of 17 species previously classified as *Copelatus* and recorded from New Guinea revealed that nine of them belong to the new genus, *Austrelatus* gen. nov. The remaining eight known New Guinea species stay in the genus *Copelatus: C.biroi* Guignot, 1956, *C.gentilis* Sharp, 1882, *C.marginatus* Sharp, 1882, *C.martinbaehri* Hendrich, Shaverdo, Hájek & Balke, 2019, *C.portior* Guignot, 1956, *C.subterraneus* Guéorguiev, 1978, *C.tenebrosus* Régimbart, 1880, and *C.tulagicus* Guignot, 1942. These eight *Copelatus* species are charaterised by 6+1 elytral striae, a simple, hook- or sickle-like median lobe of the aedeagus, without division into dorsal and ventral sclerites, with or without median processes, with the paramere of broad, semi-elliptical form (Figs 1, 2), and TL < 6.75 mm. The species are most likely closely related to the representatives of the *C.doriae-masculinus* (*C.tenebrosus*) and *oblitus-sociennus* species complexes (*C.portior*), as well as to the larger *lineatus-gentilis* complex (the remained species) (Hájek, Hendrich, and Balke (2018). With the exception of *C.biroi* and *C.subterraneus* currently known only from the type material, the species are not endemics of New Guinea and were also recorded from Australia, the Solomon Islands and the Indonesian Archipelago. Additionally, there are approximately eight more new *Copelatus* species in New Guinea; their descriptions and revision of the previously known species, including an evaluation of the taxonomic status of *C.biroi* and *C.subterraneus*, will be provided in an upcoming publication (Shaverdo et al., unpublished data).

#### ﻿ Austrelatus gen. nov.

Taxon classificationAnimaliaColeopteraDytiscidae

8E4B4AA8-D794-551C-9CB9-14707B1C40F5

https://zoobank.org/622BD131-D45A-4CF6-BD14-0923592A0596

##### Type species.

*Copelatusirregularis* W.J. Macleay, 1871: 126, by present designation.

##### Material studied.

***Copelatusbadeni* Sharp, 1882**: 1 male “Taveuni. Fiji. (Viti Levu) Sylvester Evans” [hw], “Fiji. Dr. H.S. Evans” [hw], “Copelatus Badeni [hw] det. A. Zimmermann [pr]” (BMNH).

***Copelatusboukali* Hendrich & Balke, 1998: *Holotype***: male “S-Indien: Kerala (7), 15 km SW Munnar Kallar Valley 76°68'E 10°02'N”, “1000 m 6.-18.12.1993 leg. Boukal & Kejval” (NHMW).

***Copelatusdavidi* Wewalka, 2017: *Holotype***: male “India: Kerala, 1.1.1999 30 km NNE Trivandrum, 400 m Kallar Bridge, 08°45'N 77°05'E leg. D. Boukal (35)” (NHMW).

***Copelatusfidschiensis* Zimmermann, 1928: *Syntypes***: 1 male “Type”, [round label with red frame], “C81 Suva 25-3-27” [hw], “Fiji Islands. H.W. Simmonds.”, “Pres. by Imp. Bur. Ent. Brit. Mus. 1972–355”, “Copelatusfidschiensis Type. Zimerm. [hw] det. A. Zimmermann [pr]” (BMNH). 1 male “Co-type”, [round label with yellow frame], “C81 Suva 25-3-27” [hw], “Fiji Islands. [pr] R.W. Faime [hw]”, “Pres. by Imp. Bur. Ent. Brit. Mus. 1972–355”, “Copelatusfidschiensis, ♂ Zim. Cotype.” [hw] (BMNH).

***Copelatusgestroi* Régimbart, 1892: *Lectotype*** (by present designation for nomenclatural stability): male “N. GUINEA MER. RIGO *Luglio 1889* L.Loria”, “Neu Guinea Mus. Genua”, “Copelatus Gestroi Reg.” [hw], “Coll. Mus. Vindob.”, “Lectotype *Copelatusgestroi* Régimbart, 1892 des. H. Shaverdo 2023” [red] (NHMW). *Paralectotypes* (with red labels): 2 males, 6 females “N. GUINEA MER. RIGO *Luglio 1889* L.Loria”, “Neu Guinea Mus. Genua” (NHMW). 1 male “N. GUINEA MER. RIGO *Luglio 1889* L.Loria”, “Neu Guinea Mus. Genua”, “Copelatus Gestroi Reg.” [hw] (NHMW). 1 male “N. Guinea Rigo Luglio 1889 L.Loria” [hw], “Neu Guinea Mus. Genua” (NHMW). 2 males “N. GUINEA MER. RIGO *Luglio 1889* L.Loria”, “Cotype” [red], “Regimbart det., 1891: Copelatus (s. str.) Gestroi – Reg.” [partly hw] (ZSM). 2 males “N. GUINEA MER. RIGO *Luglio 1889* L.Loria”, “Neu Guinea 7407” [partly hw], “Copelatus Gestroi Rg det. Gschwendt.” [hw], “Staatl. Museum für Tierkunde, Dresden” (MTD). 2 females “N. GUINEA MER. RIGO *Luglio 1889* L.Loria”, “Neu Guinea 7407” [partly hw], “Staatl. Museum für Tierkunde, Dresden” (MTD). 3 males, 2 females “N. GUINEA MER. RIGO *Luglio 1889* L.Loria”, “Neu Guinea 8947” [partly hw], “Staatl. Museum für Tierkunde, Dresden” (MTD).

***Copelatusluteomaculatus* Guignot, 1956: *Holotype***: male “N. Guinea Biró 97.”, “Stephansort Astrolabe B”, “Holotypus 1956 ♂ Copelatusluteomaculatus Guignot” [label with red frame, partly hw by Guignot], “Type” [red label], “Dr F. Guignot det., 1955 Copelatusluteomaculatus sp.n. HoloType ♂” [partly hw by Guignot] (HNHM). ***Paratypes***: 2 females “N. Guinea Biró 1898”, “Simbang Huon Golf // IX.17. [hw on reverse side]”, “Paratypus 1956 Copelatusluteomaculatus Guignot” [label with red frame, partly hw by Guignot] (HNHM).

***Copelatuspapuensis* J. Balfour-Browne, 1939: *Holotype***: male “♂” [hw, next to beetle], “Type” [round label with red frame], “PAPUA: Kokoda. 1,200ft. v.1933. L.E. Cheesman. B.M.1933-577. // 57 [hw on reverse side]”, “Copelatuspapuensis, B-B. ♂ TYPE” [hw by J. Balfour-Browne], “Manuscript name” [printed in red], “Holotype” [red label] (BMNH). ***Paratypes***: 1 female “♀” [hw, next to beetle], “Type” [round label with red frame], “PAPUA: Kokoda. 1,200ft. v.1933. L.E. Cheesman. B.M.1933-577. // 57 [hw on reverse side]”, “Copelatuspapuensis, B-B. ♀ TYPE” [hw by J. Balfour-Browne] (BMNH).

***Copelatusstrigosulus* Fairmaire, 1878**: 1 male “♂”, “am3” [hw], “Fiji. Viti Levu near Suva Dr M. Larcb [?] 22.II.53” [hw], “put-hole stream edge” [hw], “Copelatus strigulosus Fairm [hw] C.M.F. von Hayek det., 1954 [pr]”, “Brit. Mus. 1987-14” (BMNH).

***Copelatusternatensis* Régimbart, 1899: *Syntypes***: 1 male “Ternate X [hw] Beccari 187 [pr] 5 [hw]”, “C. sp. 2882.” [yellow label, hw], “Copelatus sp. [hw] det.Régimbart [pr]”, “SYNTYPUS [pr] Copelatusternatensis Régimbart, 1899 [hw]” [red label], “Museo Civico di Genoa” [pr] (MSNG); 3 males “Ternate X [hw] Beccari 187 [pr] 5 [hw]”, “Copelatus sp. [hw] det.Régimbart [pr]”, “SYNTYPUS [pr] Copelatusternatensis Régimbart, 1899 [hw]” [red label], “Museo Civico di Genoa” [pr] (MSNG).

***Copelatuswallacei* J. Balfour-Browne, 1939**: 3 males, 5 females “INDONESIA, N Moluccas, Bacan Isl., 500–750 m, SE slopes of Mt. Sibela, 5 km SE of Makian vill., S. Jákl leg., 2.-12.V.2008” (NMPC).

***Copelatusxanthocephalus* Régimbart, 1899: *Lectotype*** (by present designation for nomenclatural stability): male [small, red, square label], “2795 78” [round label, hw], “Nolle [illegible, probably short form from Nouvelle] Guinée” [hw], “MUSEUM PARIS COLL. MAURICE REGIMBART 1908”, “Muséum Paris Nouvelle-Guinée (Amberbaki) A. Raffray 1878”, “SYNTYPE” [red], “SYNTYPE Copelatusxanthocephalus Régimbart, 1899”, “MNHN, Paris EC14220 [barcode]”, “Lectotype *Copelatusxanthocephalus* Régimbart, 1899 des. H. Shaverdo 2023” [red] (MNHN). *Paralectotype*: 1 female “2795 78” [round label, hw], “Nolle [illegible, probably short form from Nouvelle] Guinée Raffray” [hw], “MUSEUM PARIS COLL. MAURICE REGIMBART 1908”, “xanthocephalus Rég” [hw], “Muséum Paris Nouvelle-Guinée (Amberbaki) A. Raffray 1878”, “SYNTYPE” [red], “SYNTYPE Copelatusxanthocephalus Régimbart, 1899”, “MNHN, Paris EC14219 [barcode]”, “Paralectotype *Copelatusxanthocephalus* Régimbart, 1899 des. H. Shaverdo 2023” [red] (MNHN).

Additionally, please see the material of the species (re)described below.

##### Diagnosis.

Elytra with or without striae; metacoxal lines present but incomplete, absent close to metaventrite; male protarsomere 4 simple, with not modified (thin) anterolateral seta; median lobe of aedeagus complex, with evident dorsal and ventral sclerites usually divided in apical half into two lobes of different shape or otherwise modified; paramere simple, of narrow triangular form, with thin apex and numerous setae; male pro- and mesotarsomeres 1–3 dilated, with ca. 40–55 small stalked adhesive discs in five or six rows, larger discs can be present in second and third rows, two or three in each; female metatibia and metatarsus with dorsal and ventral rows of natatorial setae.

*Austrelatus* gen. nov. can be distinguished from all other genera of Copelatinae by the above combination of characters. From the most species of two co-occurring Australasian genera, *Exocelina* and *Copelatus*, representatives of the new genus differ by the median lobe of the aedeagus distinctly divided into dorsal and ventral sclerites, which are usually well-developed and differently modified; from *Exocelina*, additionally by anterolateral seta of the male protarsomere 4 thin, not modified into a hook; see also the generic description of the male genitalia, key to Copelatinae of New Guinea and illustrations below.

##### Description.

***Body size and form***: Beetles small to large, TL ca. 4–9 mm, with usually oblong-oval, more seldom oval or elongate, almost sub-parallel habitus, distinctly convex in lateral view; body outline continuous, without discontinuity between bases of pronotum and elytra.

***Colouration***: Beetles reddish to piceous, usually with paler head and pronotal sides, and often with paler, to yellowish, basal band or spot(s) and apical spot on elytron.

***Surface sculpture***: Elytron usually with up to 11 dorsal striae or sometimes without them; submarginal stria present or absent: (0–11)+(0–1). Head, pronotum and elytron sometimes with strioles. Dorsal and ventral surface with fine punctation and microreticulation.

***Structures***: Head with anterior margin of clypeus slightly and evenly concave, with distinct bead laterally on both sides. Pronotum broadest at posterior angles; lateral margins slightly to distinctly convergent anteriorly, posterior angles slightly rounded. Pronotal base as broad as base of elytra. Pronotum with thin lateral bead missing at anterior angles. Elytron with distinct lateral bead weakening at apex. Base of prosternum narrowly to broadly rounded anteriorly, convex medially; neck of prosternal process convex; neck and blade of prosternal process evenly joined; blade of prosternal process lanceolate, with distinct lateral bead laterally and broadly pointed apex. Lateral parts of metaventrite tongue-shaped, very slender. Metacoxal lines divergent, sometimes only slightly, distinct, or indistinct close to metaventrite, not reaching it. Abdominal ventrite 6 beaded, broadly rounded, seldom slightly truncate.

***Male***: Antenna simple, with antennomeres long and slender. Protibia usually simple, i.e., with roughly triangular outline; more seldom, modified: thinner proximally, broader medially and distally due to its curved ventral margin. Pro- and mesotarsomeres 1–3 distinctly broadened, with adhesive discs on their ventral side. Protarsomere 4 small, with several long, thin setae. Protarsomere 5 long, thin, with small, sometimes inconspicuous setae medially. Proclaws relatively long and equal; more seldom, anterior claw shorter, modified.

Median lobe of aedeagus with well-developed dorsal and ventral sclerites of different shape, modification, and degree of sclerotisation: in species from west of Wallace Line (India and Greater Sunda), apex of median lobe usually pointed in ventral view, ventral sclerite much shorter than dorsal one, sclerites apically not divided into two lobes; in species from east of Wallace Line, dorsal and ventral sclerites subequally developed, apex of each sclerites usually divided apically into two distinct lobes. Paramere simple, of triangular form, rarely elongate and often broad basally, either continuously or abruptly narrowing to slender apical part (sometimes paramere subdivided with less sclerotised area to broad basal and narrow apical parts); with dense long setae in apical half of dorsal margin; in some species, paramere setae can be divided by a median or submedian part with few sparse setae into distal and proximal setae; distal setae more numerous, denser, and usually longer than proximal ones; especially in left paramere of some species, proximal setae less numerous and sparse.

***Female***: Similar to males in external morphology, except for not modified protibial, pro- and mesotarsi, and proclaws. Most of the females have more intensive dorsal surface sculpture, e.g., sometimes with numerous thin, longitudinal strioles on elytra and pronotum that densely cover dorsal surface and make it matt; dimorphic females are known in one species from the Solomon Islands and several New Guinean species. Metatibia and metatarsus with dorsal and ventral rows of natatorial setae.

##### Molecular study.

The species characterised by the above-mentioned morphological characters were all retrieved in one clade, which is the sister to all other analysed *Copelatus*. To highlight this dichotomy, we also decided to propose a new genus name for all these species.

##### Etymology.

The name of the new genus *Austrelatus* combines the Latin part *Austr*- (southern) and Greek part -*elatus* (from elaunein, to row) and means “southern rower”. It has also been chosen to indicate that its type species and most of its known representatives, including the former *Copelatus* species, occur in and are even endemic to the Australian Region.

##### Distribution and species composition.

The new genus is mainly known from the Australian Region (Australia, New Guinea, Fiji, Solomon Islands, Maluku Island), with several species penetrating the Wallace Line into the Oriental and SE Palaearctic regions (Greater and Lesser Sunda Islands, India, SE China, and Japan); currently, no *Austrelatus* is known from continental SE Asia.

In Australia, *Austrelatus* is represented by six species with rather distinct morphology; only *A.irregularis* and *A.daemeli* can be grouped together based on similarity of their male genitalia.

Nine species of *Austrelatus* have so far been known from New Guinea. However, the genus comprises more than 60 different species there, most of which can be grouped in two large species groups: the *A.papuensis* group (ca. 40 species, including *A.gestroi*, *A.luteomaculatus*, *A.papuensis*, and *A.xanthocephalus* partly treated here), which will be defined in detail (Shaverdo et al., unpublished data), and the *A.neoguineensis* group introduced here with three previously known and 29 new species. *Austrelatussetiphallus* sp. nov. and *A.fumato* sp. nov. are very similar to the representatives of the *A.neoguineensis* group according to our unpublished molecular phylogenetic analysis but shows rather special morphology of the median lobe and parameres; therefore, they are treated separately.

From ten known Copelatinae species of Solomon Islands, *Austrelatus* gen. nov. is represented by six species in two species groups: *A.laevipennis*, *A.variistriatus*, and *A.urceolus* most likely form an inherent group presumably endemic to Solomons. *Austrelatusbaranensis*, *A.bougainvillensis*, and *A.kietensis* belong to the above-mentioned *A.papuensis* group.

All Fijian copelatine species belong to *Austrelatus* gen. nov. They form a monophyletic group again based on our unpublished molecular phylogenetic analysis, and are very similar morphologically. However, only five species have been described so far; more than 25 species have already been delineated based on molecular methods and morphology (Monaghan et al. 2006; Shaverdo et al., unpublished data).

Four species of the new genus are known from the Indonesian Archipelago. *Austrelatusuludanuensis* from Bali is morphologically different from other species, probably representing a well delineated clade. The Moluccan species *A.sibelaemontis*, *A.ternatensis* and *A.wallacei* most likely belong to the *A.papuensis* group. Several additional undescribed *Austrelatus* species are known to us across the Indonesian Archipelago, from Sumatra and Kalimantan to Flores, Ceram, and Kai (Hájek et al., unpublished data).

The Oriental and SE Palaearctic representatives of the new genus are five species from India, which all have a characteristic structure of the male genitalia, and *A.parallelus* from Japan and China. They build a rather interesting complex outside the Australasian species.

At present, 62 *Austrelatus* species are known, including the new, described here New Guinean species (Table 1).

**Table 1.** Checklist of the known species of *Austrelatus* gen. nov. For more information on some species refer to Nilsson and Hájek (2023) under the genus *Copelatus*.

	Species	Distribution
1	*A.adelbert* (Megna et al., 2017), comb. nov.	New Guinea
2	*A.badeni* (Sharp, 1882), comb. nov.	Fiji
3	*A.bakewelli* (J. Balfour-Browne, 1939), comb. nov.	Australia
4	*A.baliem* sp. nov.	New Guinea
5	*A.baranensis* (Hájek et al., 2021), comb. nov.	Solomon Islands
6	*A.bormensis* sp. nov.	New Guinea
7	*A.bougainvillensis* (Hájek et al., 2021), comb. nov.	Solomon Islands
8	*A.boukali* (Hendrich & Balke, 1998), comb. nov.	India
9	*A.brazza* sp. nov.	New Guinea
10	*A.clarki* (Sharp, 1882), comb. nov. (= *Copelatusvagestriatus* Zimmermann, 1919, syn. nov.)	Australia and New Guinea
11	*A.daemeli* (Sharp, 1882), comb. nov.	Australia
12	*A.davidi* (Wewalka, 2017), comb. nov.	India
13	*A.debulensis* sp. nov.	New Guinea
14	*A.deccanensis* (Sheth et al., 2018), comb. nov.	India
15	*A.fakfak* sp. nov.	New Guinea
16	*A.febrisauri* sp. nov.	New Guinea
17	*A.fidschiensis* (Zimmermann, 1928), comb. nov.	Fiji
18	*A.fojaensis* sp. nov.	New Guinea
19	*A.fumato* sp. nov.	New Guinea
20	*A.garainensis* sp. nov.	New Guinea
21	*A.gestroi* (Régimbart, 1892), comb. nov. (= *Copelatusneogestroi* Balke, 2008)	New Guinea
22	*A.innominatus* sp. nov.	New Guinea
23	*A.irregularis* (W.J. Macleay, 1871), comb. nov.	Australia
24	*A.kaszabi* (Guignot, 1956), comb. nov.	New Guinea
25	*A.kietensis* (Hájek et al., 2021), comb. nov.	Solomon Islands
26	*A.laevipennis* (Hájek et al., 2021), comb. nov.	Solomon Islands
27	*A.lembenensis* sp. nov.	New Guinea
28	*A.lisae* sp. nov.	New Guinea
29	*A.luteomaculatus* (Guignot, 1956), comb. nov.	New Guinea
30	*A.manokwariensis* sp. nov.	New Guinea
31	*A.maushomi* (Sheth et al., 2018), comb. nov.	India
32	*A.mimika* sp. nov.	New Guinea
33	*A.mirificus* sp. nov.	New Guinea
34	*A.moreguinensis* sp. nov.	New Guinea
35	*A.nadjae* sp. nov.	New Guinea
36	*A.neoguineensis* (Zimmermann, 1919), comb. nov.	New Guinea
37	*A.nigrolineatus* (Sharp, 1882), comb. nov.	Australia
38	*A.oksibilensis* sp. nov.	New Guinea
39	*A.papuensis* (J. Balfour-Browne, 1939), comb. nov.	New Guinea
40	*A.parallelus* (Zimmermann, 1920a), comb. nov.	Japan and China
41	*A.pseudoneoguineensis* sp. nov.	New Guinea
42	*A.pseudooksibilensis* sp. nov.	New Guinea
43	*A.rajaampatensis* sp. nov.	New Guinea
44	*A.rouaffer* sp. nov.	New Guinea
45	*A.rugosus* sp. nov.	New Guinea
46	*A.sandaunensis* sp. nov.	New Guinea
47	*A.sarmiensis* sp. nov.	New Guinea
48	*A.schuhi* (Hendrich & Balke, 1998), comb. nov.	India
49	*A.securiformis* sp. nov.	New Guinea
50	*A.setiphallus* sp. nov.	New Guinea
51	*A.sibelaemontis* (Hájek et al., 2010), comb. nov.	Northern Maluku: Bacan Island
52	*A.strigosulus* (Fairmaire, 1878), comb. nov.	Fiji
53	*A.ternatensis* (Régimbart, 1899), comb. nov.	Northern Maluku: Ternate Island
54	*A.testegensis* sp. nov.	New Guinea
55	*A.toricelli* sp. nov.	New Guinea
56	*A.uludanuensis* (Hendrich & Balke, 1995), comb. nov.	Bali
57	*A.urceolus* (Hájek et al., 2021), comb. nov.	Solomon Islands
58	*A.vagauensis* sp. nov.	New Guinea
59	*A.variistriatus* (Hájek et al., 2021), comb. nov.	Solomon Islands
60	*A.wallacei* (J. Balfour-Browne, 1939), comb. nov.	Northern Maluku: Bacan Island
61	*A.wanggarensis* sp. nov.	New Guinea
62	*A.xanthocephalus* (Régimbart, 1899), comb. nov.	New Guinea

### ﻿Key to Copelatinae of New Guinea

**Table:** 

1	Median lobe of aedeagus hook- or sickle-like, without distinct division into dorsal and ventral sclerites due to their fusion, sometimes with a short median process on ventral side. Paramere of broad, semi-elliptical form (Figs 1, 2). Elytron with striae and sometimes strioles	***Copelatus***
–	Median lobe of aedeagus complex, with distinct dorsal and ventral sclerites. Paramere of narrow triangular form, sometimes modified: divided in subdistal and proximal parts with a median notch. Elytron with or without striae and/or strioles	**2**
2	Male protarsomere 4 sometimes modified, usually with large, thick, hook-like anterolateral seta (Fig. 3A). Median lobe of aedeagus with ventral sclerite distinctly smaller and more weakly sclerotised than dorsal one, situated in ventral concavity of dorsal sclerite, weakly visible in lateral view; dorsal sclerite not bilobed apically (Fig. 3B, C). Paramere often with modifications in shape and setation (Fig. 3D). Elytron without striae and seldom with strioles (Fig. 3E)	***Exocelina***
–	Male protarsomere 4 simple, with long, thin setae. Median lobe of aedeagus with dorsal and ventral sclerites subequally developed; ventral sclerite large, usually strongly sclerotised and modified, distinctly visible in lateral view; dorsal sclerite and usually ventral one bilobed apically. Paramere simple. Elytron with or without striae and/or strioles	***Austrelatus* gen. nov. 3**
3	Left lobe of dorsal sclerite of median lobe with distinct surface sculpture: scales with fine spinulae or large seta-like spinulae (Figs 4–6)	**4**
–	Left lobe of dorsal sclerite of median lobe without distinct surface sculpture, smooth or with fine scales (Figs 8, 10–12)	**5**
4	Left lobe of dorsal sclerite of median lobe with numerous scales with short or rather long spinulae distinctly visible in lateral view; apexes of lobes of dorsal and ventral sclerites without strong modification, elongate, more or less pressed together (Figs 4, 5). Beetle larger, TL > 4.5 mm, body shape and colouration vary. Elytron with number of striae and degree of their development very variable among species and within one species, (0–11)+(0–1), usually with 11 dorsal and with or without submarginal stria	***A.papuensis* group**
–	Left lobe of dorsal sclerite of median lobe with numerous, very dense, long, seta-like spinulae; apexes of lobes of dorsal and ventral sclerites elongate, rounded, disposed more freely (Figs 6A–C, 7A–C). Beetle smaller, TL 3.95–4.35 mm, with elongate habitus, subparallel sides, broad head and long pronotum. Elytron with 6+1 setae, sometimes with strioles between them; dark brown to piceous, with a yellow basal band and large apical spot (Fig. 6D)	***A.setiphallus* sp. nov.**
5	Median lobe of aedeagus with dorsal and ventral sclerites distinctly separated medially forming a median hole in lateral view; left lobe of dorsal sclerite reduced, small, with pointed apex, closely pressed to enlarged right lobe; apex of right lobe broadly rounded (Fig. 8A). Elytron with (3–8)+(0–1) striae (Fig. 8B)	***A.clarki***
–	Median lobe of aedeagus with dorsal and ventral sclerites placed more closely, without an obvious median hole between them in lateral view; two lobes of dorsal sclerite developed more or less identically strong, almost equal in size. Elytron with different number of striae	**6**
6	Beetle broadly oval, extensively yellowish dorsally (Fig. 9). Paramere of narrow elongate form, with setae of equal length. Median lobe: ventral sclerite of complex folded form, not divided into two lobes, more weakly sclerotised than dorsal one; dorsal sclerite divided into two broad, rather subequal apically, in shape and length lobes (Fig. 10)	***A.fumato* sp. nov.**
–	Beetle usually narrower, distinctly more elongate, dorsally piceous or with distinct yellow pattern. Paramere of narrow triangular form, with distal setae longer than proximal or with setae of equal length. Median lobe: ventral sclerite of median lobe divided in apical half into two lobes of different shape; dorsal sclerite divided into two often very differently shaped apical lobes, right dorsal lobe of many species with a “swollen”, pea-like apex (e.g., Figs 11, 12, 40, 63)	***A.neoguineensis* group**

**Figure 3.**
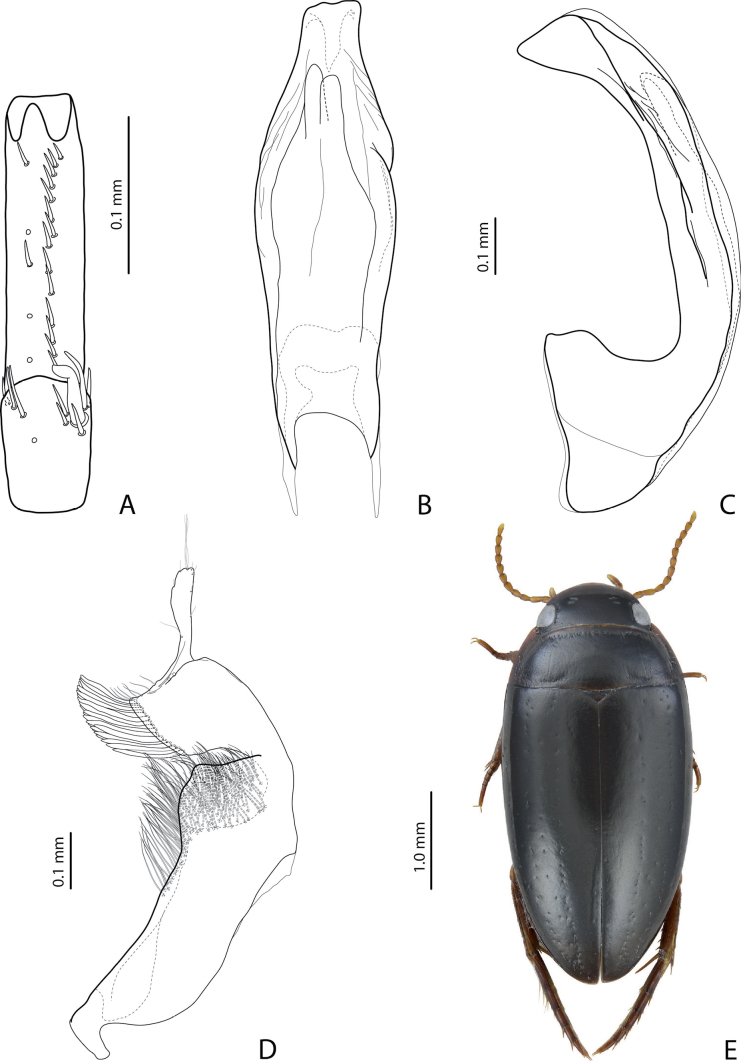
*Exocelinawaaf* Shaverdo, Surbakti & Balke, 2021 **A** right protarsomeres 4–5 in ventral view **B** median lobe in ventral view **C** median lobe in lateral view **D** left paramere in external view **E** habitus and colouration (Shaverdo et al. 2021: 54, 55, figs 2, 6).

**Figure 4.**
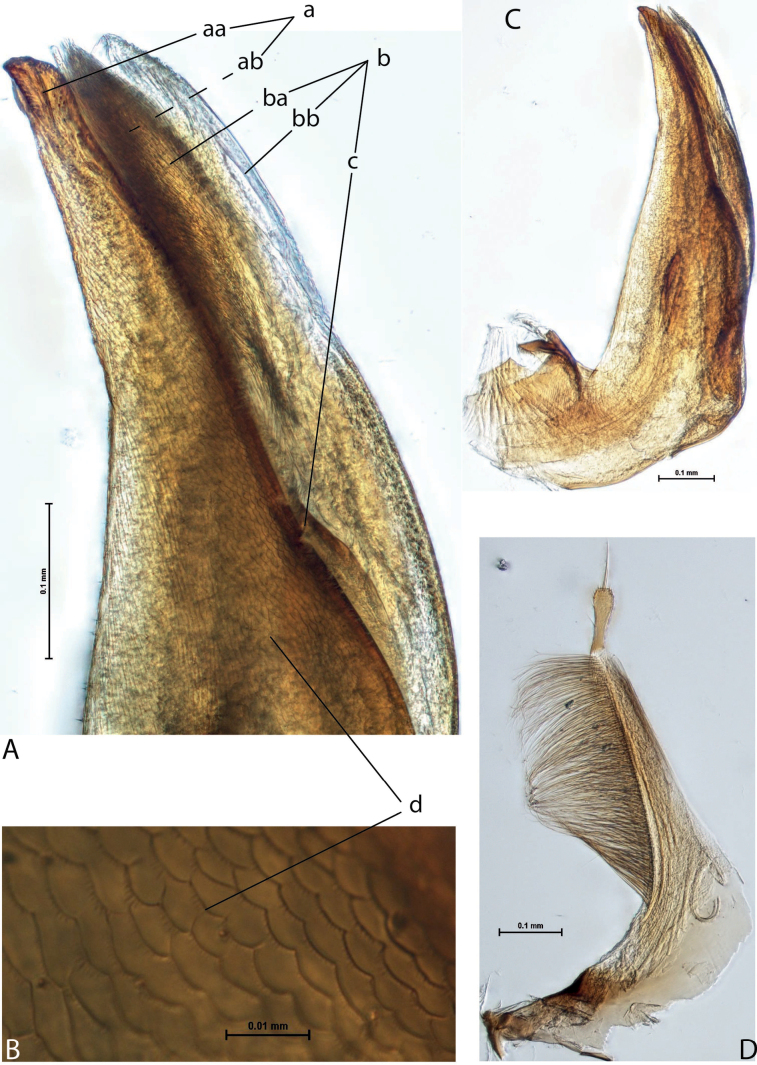
*Austrelatuspapuensis* (Balfour-Browne, 1939), paratype **A–C** median lobe in left lateral view at different magnifications **D** left paramere in external view. Abbreviations: **a** dorsal sclerite **aa** left dorsal lobe **ab** right dorsal lobe **b** ventral sclerite **ba** left ventral lobe **bb** right ventral lobe **c** sclerotised area of left ventral lobe **d** scales with fine spinulae.

**Figure 5.**
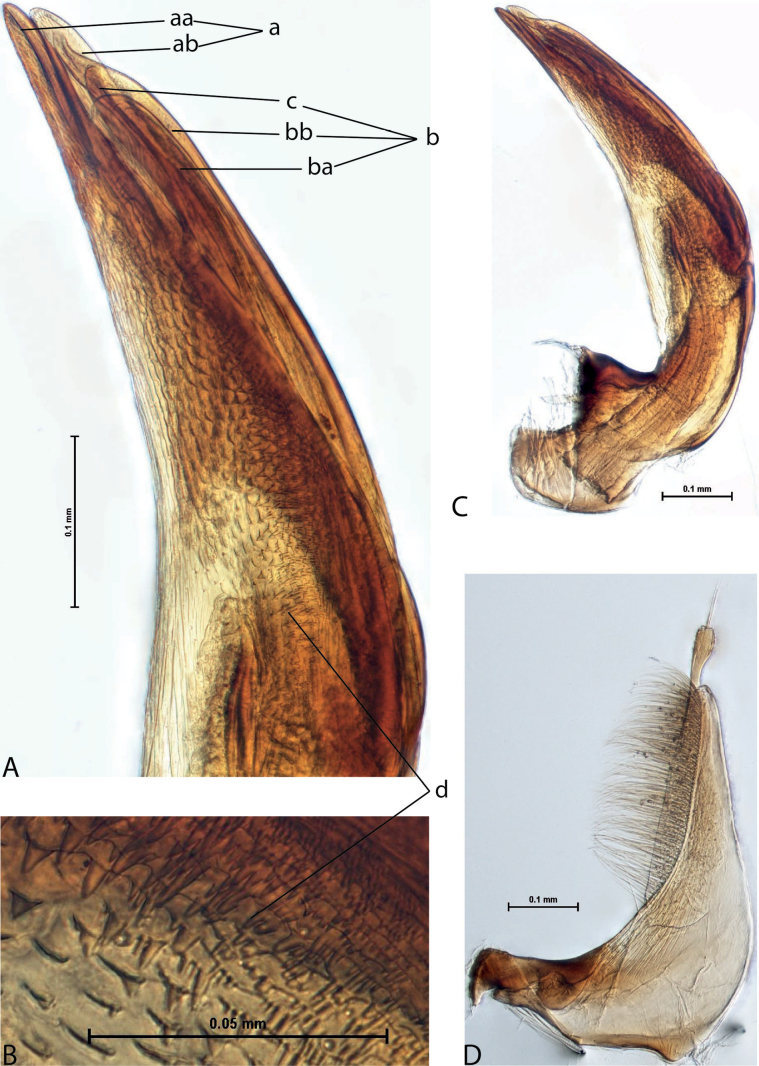
*Austrelatusgestroi* (Régimbart, 1892), lectotype **A–C** median lobe in left lateral view at different magnifications **D** left paramere in external view. Abbreviations: **a** dorsal sclerite **aa** left dorsal lobe **ab** right dorsal lobe **b** ventral sclerite **ba** left ventral lobe **bb** right ventral lobe **c** sclerotised area of left ventral lobe **d** scales with fine spinulae.

**Figure 6.**
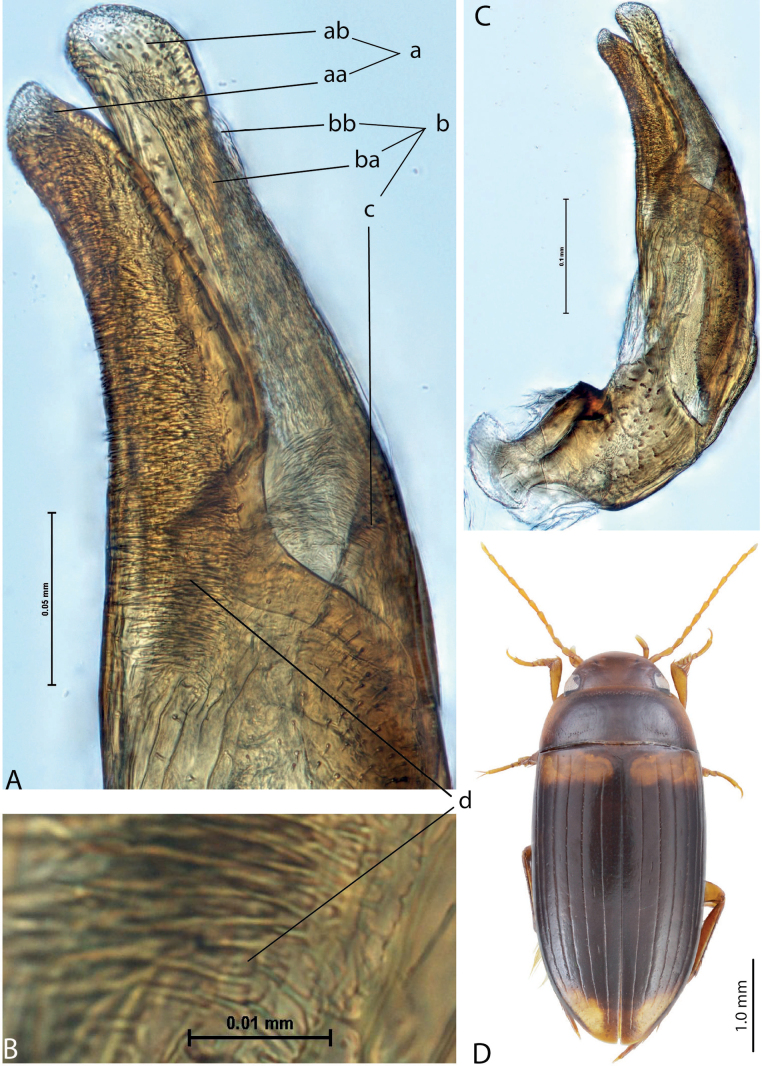
*Austrelatussetiphallus* sp. nov. **A–C** median lobe in left lateral view at different magnifications **D** habitus and coloration. Abbreviations: **a** dorsal sclerite **aa** left dorsal lobe **ab** right dorsal lobe **b** ventral sclerite **ba** left ventral lobe **bb** right ventral lobe **c** sclerotised area of left ventral lobe **d** seta-like spinulae.

**Figure 7.**
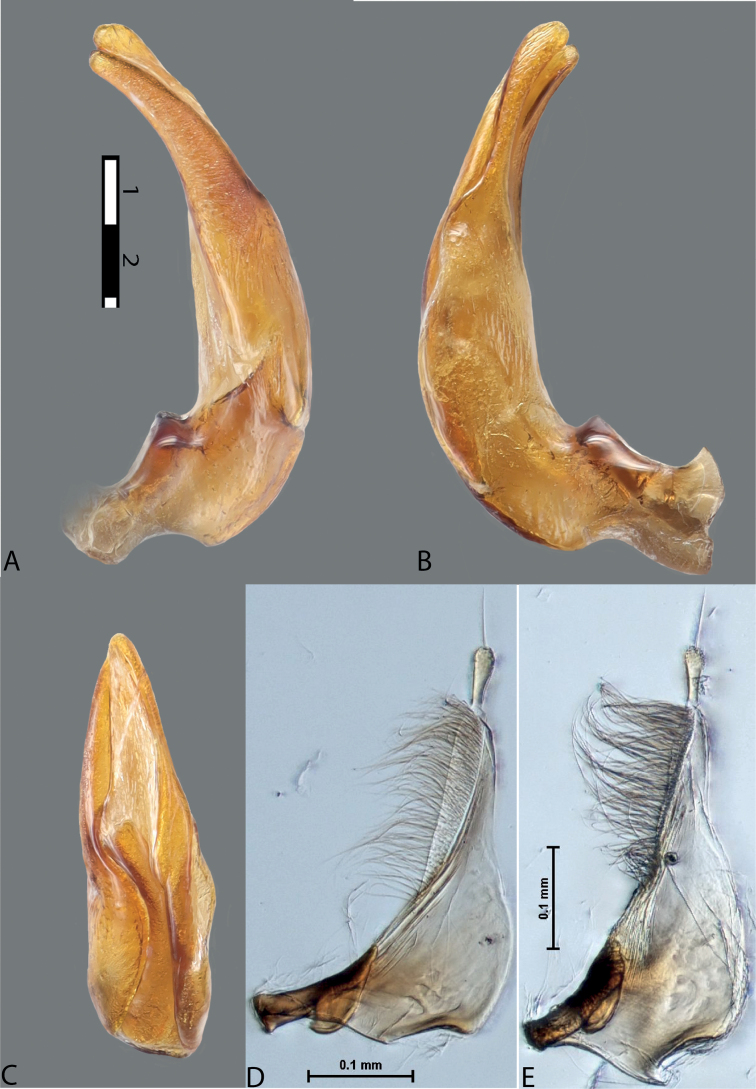
*Austrelatussetiphallus* sp. nov., median lobe **A** left lateral view **B** right lateral view **C** ventral view **D** left paramere in external view **E** right paramere in internal view. Abbreviation: **a** seta-like spinulae. Scale bar: one unit – 0.1 mm (**A–C**).

**Figures 8, 9.**
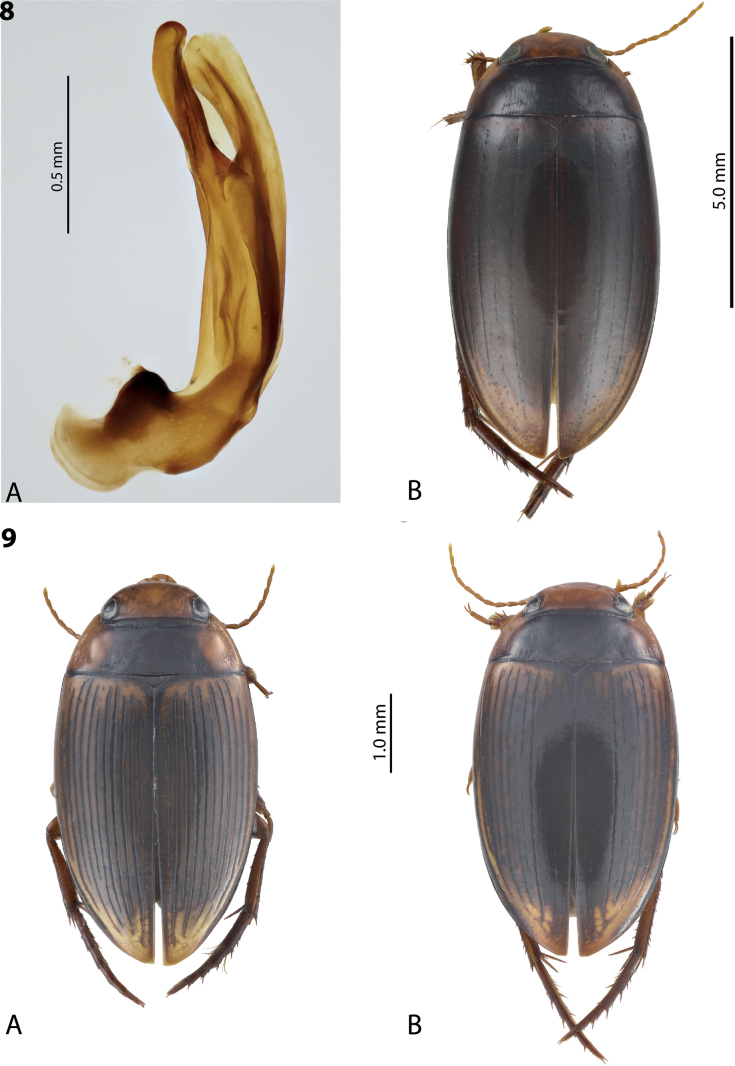
**8***Austrelatusclarki* (Sharp, 1882) **A** median lobe in left lateral view **B** habitus and colouration of holotype of *Copelatusvagestriatus* Zimmermann, 1919, syn. nov., female **9***Austrelatusfumato* sp. nov., habitus and colouration of **A** holotype **B** specimen from Sorong.

**Figure 10.**
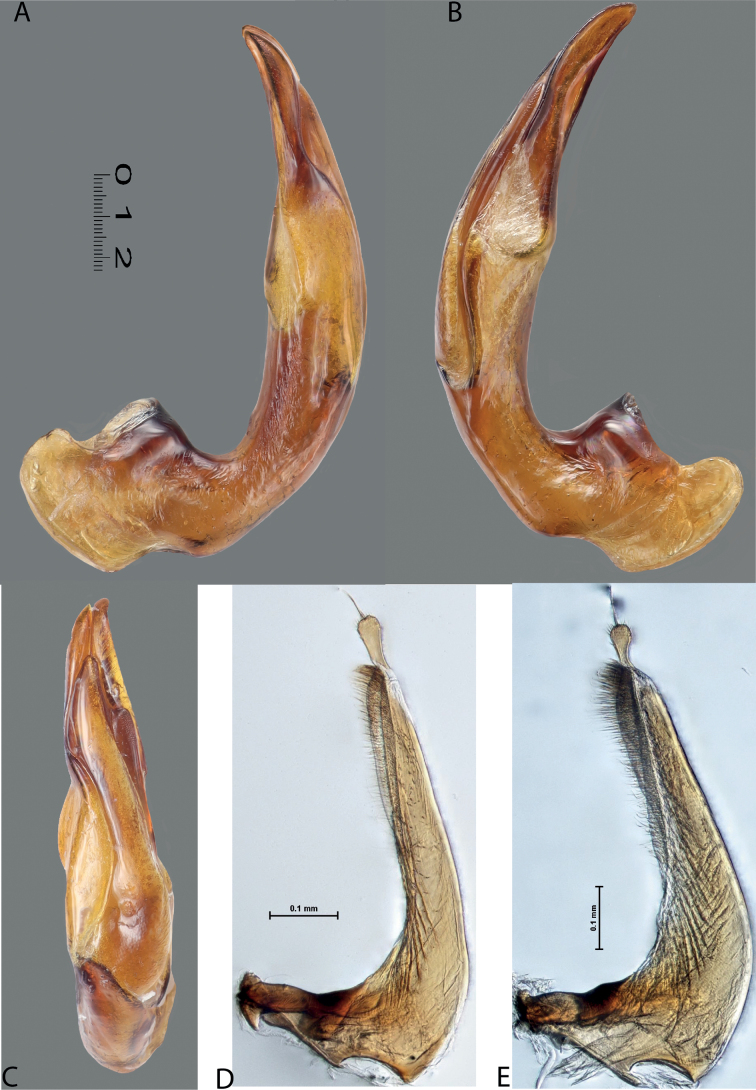
*Austrelatusfumato* sp. nov., median lobe **A** left lateral view **B** right lateral view **C** ventral view **D** left paramere in external view **E** right paramere in internal view. Scale bar: one unit – 0.1 mm (**A–C**).

**Figure 11.**
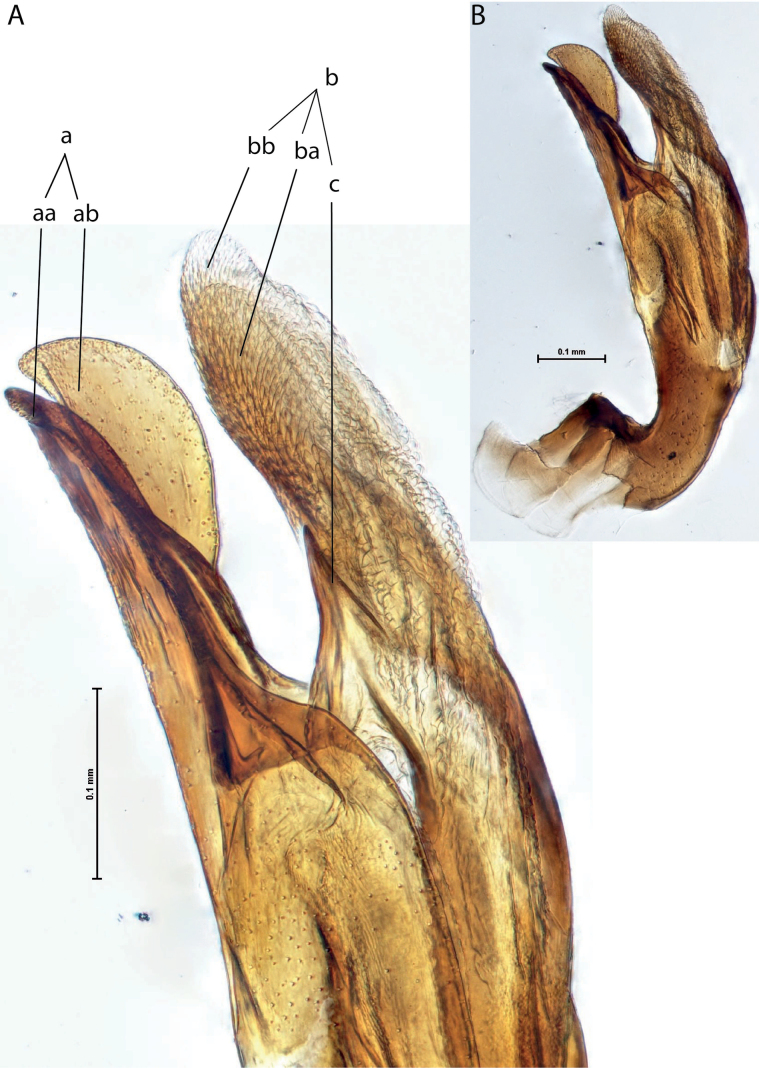
*Austrelatusneoguineensis* (Zimmermann, 1919) **A, B** median lobe in left lateral view at different magnifications, treated with lactic acid (see Materials and methods) . Abbreviations: **a** dorsal sclerite **aa** left dorsal lobe **ab** right dorsal lobe **b** ventral sclerite **ba** left ventral lobe **bb** right ventral lobe **c** sclerotised area of left ventral lobe.

**Figure 12.**
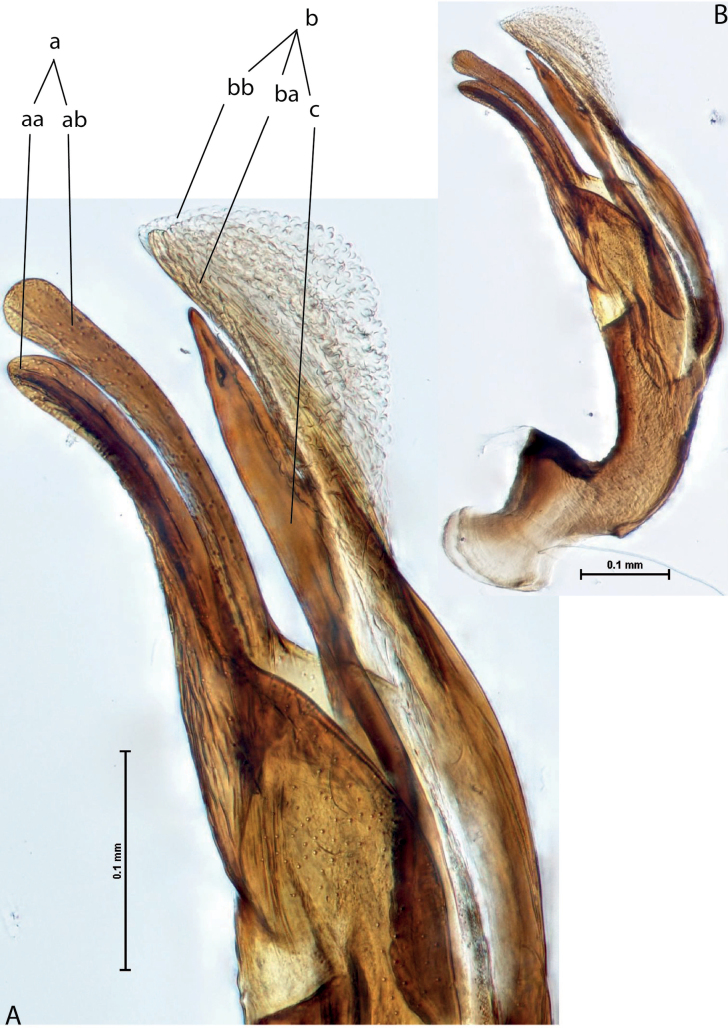
*Austrelatuskaszabi* (Guignot, 1956) **A, B** median lobe in left lateral view at different magnifications, treated with lactic acid (see Materials and methods). Abbreviations: **a** dorsal sclerite **aa** left dorsal lobe **ab** right dorsal lobe **b** ventral sclerite **ba** left ventral lobe **bb** right ventral lobe **c** sclerotised area of left ventral lobe.

### ﻿Taxonomy of New Guinean *Austrelatus* gen. nov.

#### ﻿ Austrelatus clarki

Taxon classificationAnimaliaColeopteraDytiscidae

(Sharp, 1882) comb. nov.

845362EC-E5F7-55B2-A066-BC0C6847C71F

Figs 8 , 13

Copelatus clarki Sharp 1882: 585; Hendrich et al. (2019: 89); Larson (1993: 52, 1997: 276); Watts (1978: 125, 1985: 26, 2002: 42); Nilsson and Hájek (2023: 47).
Copelatus vagestriatus Zimmermann 1919: 199; syn. nov.

##### Type localities.

*Copelatusclarki*: “Australia (Cape York)”; *C.vagestriatus*: “Neuguinea”.

##### Type material.

***Copelatusclarki. Lectotype*** (designated by Watts 1978: 125): male, “Lectotype” [round label with violet frame], “Type” [round printed label with red frame], “Cape York. 676” [hw label], “Sharp Coll. 1905-313.” [printed label], “Type to 76 Copelatusclarki n.sp. Cape Yorke” [hw label], “Copelatusclarki Sharp Det. C. Watts det. 1971” [hw label] (BMNH).

***Paralectotype***: female, “Paralectotype” [round label with blue frame], “Cotype” [roundish label with yellow frame], “Cape York. 676” [hw label], “Sharp Coll. 1905-313.” [printed label], “Copelatusclarki Cap York” [hw label], “Copelatusclarki Sharp Det. C. Watts det. 1971” [hw label] (BMNH).

***Copelatusvagestriatus. Holotype***: female “NeuGuinea”, “Coll. Kraatz”, “TYPUS” [red label], “Copelatusvagestriatus Rég. N. sp. typ.” [hw], “Holotypus” [red label] (SDEI).

##### Additional material studied.

PNG: Morobe Province, first record: 1 male, 1 female “Stn. No. 103.”, “NEW GUINEA: Morobe Dist., Finisterre Mts., Mt. Abilala, c.9,000 ft. 19–22.xi.1964.”, “M.E. Bacchus. B.M. 1965-120”, for male “measured J. Parkin 21” and for female “measured J. Parkin 22” (BMNH).

National Capital District, first record: 2 males, 1 female “Stn. No. 202.”, “PAPUA: Musgrave River, Sogeri Plateau, Nr. Pt. Moresby, 16.iii.1965”, “M.E. Bacchus. B.M. 1965-120” (BMNH). 1 male “Stn. No. 206.”, “PAPUA: Loloki c. 10 m. N. of Pt. Moresby. 19.iii.1965”, “M.E. Bacchus. B.M. 1965-120” (BMNH). 1 male “Stn. No. 207a.”, “PAPUA: Moitaka, c. 7 m. N. of Pt. Moresby. 17.iii.1965”, “M.E. Bacchus. B.M. 1965-120” (BMNH).

##### Diagnosis.

Beetle large: TL 7.2–8.0 mm, TL-H 6.5–7.2 mm, with oblong-oval habitus. Dorsal colouration piceous, with reddish brown head and pronotal sides, sometimes also elytron basally. Elytron with (3–8)+(0–1) striae, and no striae or only apical traces of two additional striae inwards from first row of serial punctures. Median lobe of aedeagus with dorsal and ventral sclerites distinctly separated medially forming a median hole; dorsal and ventral sclerites each divided into two lobes in apical half; lobes of dorsal sclerite unequally developed: left lobe reduced, small, with pointed apex, closely pressed to enlarged right lobe; apex of right lobe broadly rounded; left lobe of dorsal sclerite without distinct surface sculpture, smooth. Paramere with setae divided into distal and proximal; proximal setae slightly shorter and sparser than distal ones.

For the detailed morphological description, illustrations, NG material and data on its distribution and habitat, see Hendrich et al. 2019: 89–95, 126, 127, 129, 137, 143.

##### Description of holotype of *Copelatusvagestriatus*.

***Body size and form***: Beetle large, with oblong-oval habitus (Fig. 8B).

***Measurements***: TL 6.8 mm, TL-H 6.4 mm, MW 3.4 mm, TL/MW 2; PL 0.95 mm, PW 2.85 mm, PL/PW 0.33; DBE 1 mm, DBE/PW 0.35.

***Colouration***: Dorsally dark brown, with yellowish red head, pronotal sites and vague reddish basal band on elytron (Fig. 8B).

Head yellowish red, dark brown behind. Pronotum dark brown, paler laterally, with yellowish red lateral sites, yellowish at anterior angles. Elytron dark brown, with vague reddish basal band and reddish lines along suture. Scutellum reddish brown. Antennae and other head appendages yellow to dark yellow. Pro- and mesolegs yellow proximally and yellowish brown distally, metalegs darker. Venter dark brown, with reddish brown prosternum and abdominal ventrites.

***Surface sculpture***: Dorsally with 5 uncomplete elytral dorsal striae, submarginal stria absent: 5+0 (Fig. 8B).

Head without strioles, with relatively sparse, weakly impressed punctation (spaces between punctures 2–4× size of punctures); punctures relatively large (diameter of punctures equal to diameter of cells of microreticulation); head with a small uneven median depression probably due to some coarser punctures at each side; a row of coarse setigerous punctures along inner eye margin present; a very short row of such punctures present at frontal angle of each eye and a longer puncture row forms fronto-clypeal depression at each head side; head with strong microreticulation. Pronotum with inconspicuous, fine strioles at posterior angles and weak longitudinal wrinkles in middle, at posterior margin; pronotal punctation finer, denser, more distinct than on head; coarse setigerous punctures form a row along anterior margin and in lateral and basolateral depressions; some coarse setigerous punctures also evident along lateral parts of posterior margin; disc of pronotum with a long, thin, longitudinal median scratch. Pronotal microreticulation distinct. Elytron with 5 uncomplete elytral dorsal striae: stria 1 the most complete one but present as row of dense punctures and strioles, stria 2 the most reduced one, present as row of sparse punctures and strioles, striae 3 and 5 present subapically as striae and basally and apically from this part as rows of dense punctures and strioles, stria 4 completely reduced in basal part and present subapically as stria; submarginal stria absent; elytral punctation fine, dense, distinct; microreticulation distinct. Ventral part with very fine, scarce, inconspicuous punctation, invisible on metaventrite and metacoxae and more distinct on abdominal ventrites, especially laterally on abdominal ventrite 6; prosternum smooth medially; metaventrite and metacoxae with distinct microreticulation, weaker medially; abdominal ventrites with weakly impressed microreticulation; metacoxal plates with numerous longitudinal strioles, abdominal ventrites 1 and 2 with numerous, long, longitudinal strioles from margin to margin, on abdominal ventrites 3–5 strioles situated laterally and turn to middle, almost horizontal, abdominal ventrite 6 without strioles.

***Structures***: Head relatively broad. Pronotum short and broad; lateral margins distinctly convergent anteriorly. Base of prosternum rounded anteriorly, slightly convex medially; blade of prosternal process rather small and narrow, distinctly convex.

***Female genitalia***: Gonocoxosternum of usual Copelatinae shape; gonocoxa long, slender, with distinctly obliquely truncated apex.

##### Variability.

There is a variability in the colouration and elytral striation among and within the populations. Although majority of Australian and New Guinea specimens have piceous dorsal colouration, with reddish brown head and pronotal sides, some specimens from the same populations show presence of a vague or rather distinct, reddish or yellowish red elytral basal band. It is characteristic for all known New Guinean populations. Whilst Australian and majority of New Guinean specimens have seven or eight dorsal elytral striae and no striae or only apical traces two additional striae inwards from the first row of serial punctures, some New Guinean representatives have a stronger reduction of the striae. For example, the holotype of *C.vagestriatus* has only five dorsal striae and no submarginal stria on the elytron. An extreme reduction of the elytral striae is observed in some specimens from the Port Moresby localities: three or four uncomplete dorsal striae, sometimes present only as rows of dense punctures and strioles, submarginal stria absent.

##### Comments to classification.

Since the holotype of *C.vagestriatus* is a female, it was difficult to decide about its taxonomic position. However, it was recognised as belonging to *Austrelatus*, not *Copelatus*, because of its large size and 5+0 elytral striae (see above for New Guinean *Copelatus* species). Our careful examination of the holotype and its comparison with all known large-sized *Austrelatus* allows us to assume that *C.vagestriatus* is in fact *A.clarki* with a strong reduction of the elytral striae and paler colouration. It shares with *A.clarki* several similarities in body size and shape, punctation and microreticulation, shape of the prosternum and female genitalia, especially shape of apexes of the gonocoxae. Its dorsal colouration and elytral striation are similar to those of the *A.clarki* specimens from Port Moresby localities. Therefore, though the type locality of *C.vagestriatus* is indicated only as New Guinea, we assume that the holotype might been collected somewhere in the National Capital District. Thus, we consider *Copelatusvagestriatus* Zimmermann, 1919, syn. nov. to be a junior subjective synonym of *Austrelatusclarki* (Sharp, 1882).

##### Distribution.

**Figure 13.**
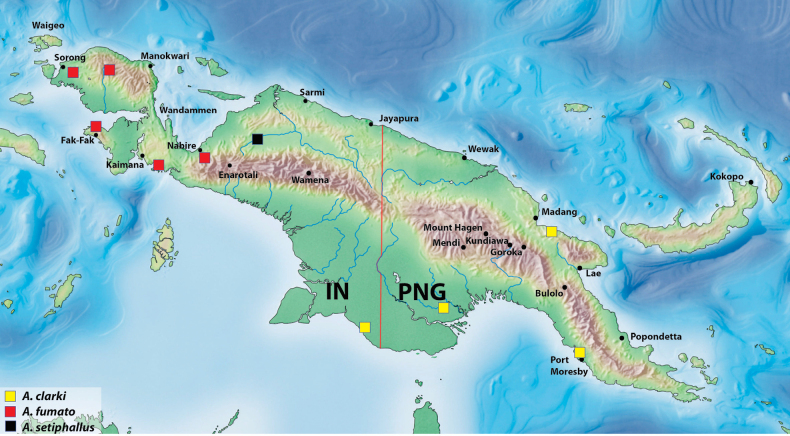
Map of New Guinea showing distribution of *Austrelatusclarki* (Sharp, 1882), *A.fumato* sp. nov., and *A.setiphallus* sp. nov.

In NG, this generally Australian species is known from the southern and eastern parts of the island: IN: Papua Province, Merauke Regency (Hendrich et al. 2019: 93) and PNG: Western Province (Hendrich et al. 2019: 93), Morobe and NCD provinces (Fig. 13).

##### Habitat.

In northern Australia and near Merauke (southern PNG), most specimens were collected in various smaller pools and puddles, also in the rest pools of intermittent creeks and smaller rivers of open and eucalypt woodland, often rich in rotten leaves and twigs. The species is also attracted at light (Hendrich et al. 2019).

#### ﻿ Austrelatus fumato sp. nov.

Taxon classificationAnimaliaColeopteraDytiscidae

9AFFBB88-65E3-56D3-BDC8-D3D1AA326935

https://zoobank.org/15DB8748-22D0-4F38-8A7C-EF44FFA4E497

Figs 9 , 10 , 13

##### Type locality.

Indonesia: West Papua Province: Manokwari Regency, Fumato, 0°54'15.4"S, 132°43'11.3"E, 820 m a.s.l.

##### Type material.

***Holotype***: male, Indonesia: Papua Barat, Fumato, forest stream, 820 m, -0.9042 132.7198 (BH027), 6209 [green text] (MZB).

***Paratypes***: IN: West Papua: Manokwari Regency: 3 males, 1 female with the same label as the holotype, one male additionally with green text label “6210” (KSP, NHMW, ZSM).

Kaimana Regency: 2 males, 2 females “INDONESIA: W-Papua 50km SE Kaimana, Triton bay, vic. Kamaka vill. trail to Kamakawalar lake, 3°48'22"S 134°14'02"E, 50–100 m, 03.II.2011 leg. A Skale (006a) small pool” (NHMW, ZSM). 1 male “INDONESIA: W-Papua ca. 50km SE Kaimana, Triton bay, vic. Kamaka village, trail to Kamakawalar lake, 3°48'22"S,134°14'02"E, 50–100 m, 03.II.2011 leg. A Skale (006a)”, “4438” [green text] (ZSM). 1 female “Ir 27-W.New Guinea, Fak-Fak, Kali Mati 4km N F.-F., 260 m, 8.-9.viii.1991 Balke & Hendrich leg.” (ZSM).

Sorong Regency: 1 male “Indonesia: Papua Barat, Sorong-Teminabuan, 50 m, 2.x.2014, -1,1092904 131,6125645 (BH046)”, “6450” [green text] (ZSM).

Papua: Nabire/Painai Regencies: 2 males, 1 female “Irian Jaya: Paniai Prov. road Nabire – Ilaga, km 54, 30.8.1996, 750 m leg. M. Balke (96 # 9)” (NHMW). 1 male, 2 females “IRIAN JAYA: Nabire dist. rd. Nabire-Ilaga, km 54 03°29.51'S, 135°43.91'E, 750 m, IV. 1998 leg. Balke”, “Restpfütze eines Baches” (NHMW).

##### Description.

***Body size and form***: Beetle medium-sized, relatively broad, with oval, egg-shaped habitus (Fig. 9A, B).

***Measurements***: TL 5.1–5.8 mm, TL-H 4.6–5.3 mm, MW 2.7–3.1 mm, TL/MW 1.78–1.92; PL 0.7–0.9 mm, PW 2.3–2.7 mm, PL/PW 0.3–0.35; DBE 1.0–1.1 mm, DBE/PW 0.41–0.43.

Holotype: TL 5.5 mm, TL-H 5.05 mm, MW 3.1 mm, TL/MW 1.78; PL 0.9 mm, PW 2.55 mm, PL/PW 0.35; DBE 1.1 mm, DBE/PW 0.43.

***Colouration***: Dorsally yellowish, with dark brown pronotal disc and thin dark brown lines along elytral striae (Fig. 9A, B).

Head pale yellow to dark yellow, dark brown behind eyes and narrowly yellowish brown to brown posteriorly. Pronotum pale yellow to dark yellow, with a large dark brown area on disc from anterior to posterior margins, sides of this brown area concave; sometimes dark brown colouration continues as a narrow band along posterior margin till lateral bead. Elytron pale yellow to dark yellow, with thin dark brown lines tracing precisely elytral striae; margins of lines sometimes vague and spaces between lines sometimes yellowish brown on elytral disc; sometimes elytron darker due to broader, vague dark brown lines, merged together, especially on elytral disc. Antennae and other head appendages yellow to dark yellow. Pro- and mesolegs yellow proximally and yellowish brown distally, metalegs darker. Venter brown to dark brown, with paler prosternum.

***Surface sculpture***: Dorsally with 4–11 often uncomplete elytral dorsal striae, submarginal stria absent: (4–11)+0 (Fig. 9A, B).

Head without strioles, with relatively dense, even punctation (spaces between punctures 1–3× size of punctures); punctures relatively large (diameter of punctures more or less equal to diameter of cells of microreticulation) but not coarse; head with a small uneven median depression probably due to some coarser punctures at each side; a row of coarse setigerous punctures along inner eye margin present; a very short row of such punctures present at frontal angle of each eye and a longer puncture row forms fronto-clypeal depression at each head side; head with strong microreticulation. Pronotum with several short strioles at very posterior angles and longitudinal wrinkles along lateral parts of posterior margin; pronotal punctation slightly finer and sparser than on head; coarse setigerous punctures form a row along anterior margin and in lateral and basolateral depressions; some coarse setigerous punctures also evident along lateral parts of posterior margin; disc of pronotum with a short, thin, longitudinal median scratch and sometimes with two small, oval, shallow, transverse lateral depressions. Pronotal microreticulation distinct. Elytron with maximum 11 dorsal striae: stria 1 often completely absent, if present, distinctly shortened and interrupted basally and especially apically: almost to the elytral half; striae 2 and 3 usually absent, rarely present as sparse strioles or complete; stria 4 usually visible as first elytral stria, relatively strongly impressed or present as a row of strioles; striae 5–7 strongly reduced: absent, or present as sparse strioles, rarely as rows of strioles apically or more or less complete, shortened and/or interrupted basally; striae 8–11 distinct, striae 9 and 10 sometimes reduced basally; a few additional very short striae can be present between striae, especially between striae 9–11; submarginal stria absent; elytral punctation fine, sparse or more distinct; single coarse, setigerous punctures observed in striae; microreticulation weak. Ventral part with very fine, scarce, inconspicuous punctation, invisible on metaventrite and metacoxae and more distinct on abdominal ventrites; prosternum smooth medially; metaventrite and metacoxae with distinct, strong microreticulation, much weaker medially; abdominal ventrites with very weakly impressed microreticulation; metacoxal plates with several longitudinal strioles, abdominal ventrites 1 and 2 with numerous, long, longitudinal strioles from margin to margin, on abdominal ventrites 3–5 strioles situated laterally and turn to middle, almost horizontal, abdominal ventrite 6 with a few very inconspicuous, small strioles near row of setigerous punctures at each side; abdominal ventrites 5 and 6 with distinct punctation that sparser medially and forms a very dense lateral area at each side.

***Structures***: Head relatively broad. Pronotum short and broad; lateral margins distinctly convergent anteriorly. Base of prosternum broadly rounded anteriorly, slightly convex medially; blade of prosternal process small, slightly, evenly convex.

***Male***: Protibia straight, not modified. Proclaws long, equal in length. Median lobe of aedeagus broad, sickle-shaped, with dorsal and ventral sclerites not separated medially, strongly pressed together. Dorsal sclerite without distinct surface sculpture, smooth; divided into two lobes apically; lobes developed more or less identically strong, with broadly pointed, slightly curved downwards apexes in lateral view. Ventral sclerite more weakly sclerotised than dorsal one, of complex folded form, not divided into two lobes. Paramere of narrow elongate form, with short setae of subequal length, not divided into distal and proximal (Fig. 10).

***Female***: Pronotal strioles more numerous than in males.

##### Variability.

There is a significant variability in the elytral striation and colouration among the populations. The specimens from Fumato show especial differences from the other material having 11 almost complete dorsal striae on elytron, almost yellow dorsal colouration, finer elytral punctation and shorter setae of the paramere. The single specimen from Fakfak has a piceous, without striae disc of the elytron, while the single beetle from Sorong has, similar to Fumato specimens, almost yellow elytra but only five or six dorsal striae on elytron (Fig. 9B).

##### Affinities.

The species is very characteristic in general appearance and structure of the male genitalia; therefore, it can be easily distinguished from the other New Guinean *Austrelatus*. Based on shape of their median lobe, they remind representatives of the *A.neoguineensis* group but distinctly differs from them by the ventral sclerite not divided into two lobes, the narrow shape of the paramere and its shorter, even setation.

##### Etymology.

The name refers to the Fumato Village where the specimens with more intensively striated elytra were collected. The species name is a noun in the nominative singular, standing in apposition.

##### Distribution.

New Guinean endemic. Indonesia: West Papua and Papua provinces (Fig. 13).

##### Habitat.

The single specimen from Fakfak, Kali Mati was collected in a water-filled tree hollow, shaded by primary rainforest. The specimens from Nabire were collected in a small rest pool of a forest stream.

#### ﻿ Austrelatus setiphallus sp. nov.

Taxon classificationAnimaliaColeopteraDytiscidae

28879699-653E-59A1-B43C-5206A6F3F5F2

https://zoobank.org/94837400-C661-466F-B41A-061C4462037D

Figs 6 , 7 , 13 , 90

##### Type locality.

Indonesia: Papua Province: Puncak Regency, Iratoi, 3°14'25.1"S, 137°19'58.7"E, 160 m a.s.l.

##### Type material.

***Holotype***: male, Indonesia: Papua, Rouaffer, Iratoi, hill in forest, 160 m, 6.ix.2014, -3,2403 137,3329, Sumoked (PAP028), 6477 [green text] (MZB).

***Paratypes***: 1 male, 4 females with the same label as the holotype, one female with an additional green text label “6478” (KSP).

##### Description.

***Body size and form***: Beetle small, with narrow, elongate habitus, elytral sides almost parallel (Fig. 6D).

***Measurements***: TL 3.95–4.35 mm, TL-H 3.6–3.9 mm, MW 1.8–1.9 mm, TL/MW 2.19–2.35; PL 0.6–0.7 mm, PW 1.6–1.7 mm, PL/PW 0.38–0.39; DBE 0.8 mm, DBE/PW 0.46–0.5.

Holotype: TL 4.3 mm, TL-H 3.9 mm, MW 1.9 mm, TL/MW 2.25; PL 0.65 mm, PW 1.7 mm, PL/PW 0.38; DBE 0.8 mm, DBE/PW 0.47.

***Colouration***: Dorsally brown to piceous, with reddish yellow to yellowish brown head, pronotal sides, and brighter, broad basal band and large apical spot on elytron (Fig. 6D).

Head reddish yellow to yellowish brown, darker behind or around eyes, sometimes with two small, dark median spots. Pronotum brown, piceous in posterior part, with broad, reddish yellow to yellowish brown sides. Elytron dark brown to piceous, with broad, yellow to reddish yellow basal band reaching or not suture and lateral elytral margin; its anterior margin reaching elytron basally and its posterior margin wavy, not distinctly notched; elytron with very large, elongate, not reaching suture, yellow to reddish yellow apical spot. Scutellum reddish brown or piceous. Antennae and other head appendages yellow. Pro- and mesolegs yellowish brown proximally and darker distally, especially metalegs. Venter reddish brown, with paler prosternum.

***Surface sculpture***: With six distinct, complete elytral dorsal striae, submarginal stria present: 6+1 (Fig. 6D).

Head without strioles, with relatively dense punctation (spaces between punctures 1–3× size of punctures); punctures relatively large (diameter of punctures equal to diameter of cells of microreticulation); head with row of coarse setigerous punctures along inner margin of each eye and sparse row of slightly weaker punctures at frontal angle of each eye connecting with puncture row that forms very shallow fronto-clypeal depression at each head side; head with strong microreticulation. Pronotum with few strioles at posterior angles (more numerous in female), with short, thin, rather inconspicuous longitudinal wrinkles at middle of posterior margin; pronotal punctation finer than on head; coarse setigerous punctures form a broad row along pronotal margins, absent in posterior middle; disc of pronotum with rather long longitudinal median scratch. Pronotal microreticulation similar to that on head. Elytron with six complete, strongly impressed dorsal striae and submarginal stria reaching to middle of elytron or slightly further; striae 2 and 5 slightly reduced basally; sometimes with few short strioles between striae. Elytron with fine, sparse punctation; microreticulation weak. Ventral part with extremely fine, scarce, inconspicuous punctation, invisible on metaventrite and metacoxae and more distinct on abdominal ventrites; prosternum smooth medially; metaventrite and metacoxae with distinct microreticulation; on abdominal ventrites microreticulation weaker; metacoxal plates with short almost longitudinal strioles, abdominal ventrites 1 and 2 with numerous, long, longitudinal strioles from margin to margin, on abdominal ventrites 3 and 4 strioles situated laterally and turn to middle, almost horizontal, abdominal ventrite 5 without strioles, abdominal ventrite 6 with few very inconspicuous, small strioles near row of setigerous punctures at each side and with rather distinct punctation that sparser medially and forms denser lateral area at each side.

***Structures***: Head large and broad. Pronotum large and long; lateral margins only slightly convergent anteriorly, subparallel, rounded towards anterior angles. Base of prosternum broadly rounded anteriorly, slightly convex medially; blade of prosternal process rather broad, evenly convex in middle.

***Male***: Protibia straight, not modified. Proclaws long, slender, equal in length; anterior claw with very weak incision subapically. Median lobe of aedeagus with dorsal and ventral sclerites not separated medially but not pressed very much to each other; dorsal sclerite sclerotised, divided into two rather narrow lobes in apical half; right lobe slightly longer than left one; in lateral view, their apexes rounded; left dorsal lobe with numerous, dense, thin, rather long spinulae distinctly visible in lateral view; right dorsal lobe with inconspicuous median impression in lateral right view. Ventral sclerite mostly membranous, indistinctly divided into right and left lobes. Paramere of narrow triangular form, evenly tapering to apex, with long, relatively dense, subequal in length setae, not divided into distal and proximal (Figs 6, 7).

***Female***: Pronotal strioles usually occupying entire lateral sides and elytral punctation stronger than in males.

##### Affinities.

Based on size, body form, colouration, and shape of the median lobe, the species is similar to some species of the *A.papuensis* group but distinctly differs from them by presence of numerous, dense seta-like spinulae on the left dorsal lobe of its median lobe.

##### Etymology.

The species name is a combination of the Latin words (*seta* and *phallus*) referring the seta-like, thin, long spinulae of the median lobe of aedeagus. The name is a noun in the nominative standing in apposition.

##### Distribution.

New Guinean endemic. Indonesia: Papua Province: Puncak Regency. The species is known only from the type locality (Fig. 13).

##### Habitat.

At the type locality, the species was collected in a side pool of a larger forest stream (Fig. 90).

### ﻿*Austrelatusneoguineensis* group

The group is represented in New Guinea by 32 species, 29 of which are new and described below (Table 2).

**Table 2.** Checklist, body size, number of elytral striae and distribution of the *Austrelatusneoguineensis* group species. IN: Indonesia, PNG: Papua New Guinea.

	Species	TL, mm	Elytral striae	Distribution
1	*adelbert* (Megna et al., 2017)	5.9–6.4	0+0	PNG: Madang
2	*baliem* sp. nov.	4.65–5.6	11+(0–1)	IN: Papua: Jayawijaya
3	*bormensis* sp. nov.	5.4–6.1	(5–10)+(0–1)	IN: Papua: Pegunungan Bintang, Yalimo
4	*brazza* sp. nov.	4.95–5.6	(0–10)+0	IN: Papua: Yahukimo
5	*debulensis* sp. nov.	4.1–4.5	11+1	IN: Papua: Yahukimo
6	*fakfak* sp. nov.	4.4–5	6+1	IN: West Papua: Fakfak, Kaimana
7	*febrisauri* sp. nov.	4.7–5.35	0+0	IN: Papua: Nabire
8	*fojaensis* sp. nov.	5.1–5.5	6+1	IN: Papua: Sarmi
9	*garainensis* sp. nov.	5.4–6.2	11+1	PNG: Morobe, EHL, Central, National Capital District, Milne Bay
10	*innominatus* sp. nov.	4.7–5.6	(10–11)+1	IN: West Papua: Teluk Wondama, Papua: Nabire
11	*kaszabi* (Guignot, 1956)	5–6.45	11+1	IN: Papua: Nabire, Sarmi, Puncak; PNG: Sandaun, East Sepik, Madang, East New Britain
12	*lembenensis* sp. nov.	5–5.25	0+0	PNG: East Sepik
13	*lisae* sp. nov.	5.6–6.2	6+0	IN: Papua: Mimika, Nabire, Sarmi
14	*manokwariensis* sp. nov.	4.6–5.7	6+1	IN: West Papua: Manokwari, Teluk Wondama
15	*mimika* sp. nov.	5.25–5.6	11+1	IN: Papua: Mimika
16	*mirificus* sp. nov.	4.85–5.5	(2–10)+(0–1)	IN: Papua: Jayapura, Yalimo, Sarmi; PNG: Sandaun, Madang, East Sepik
17	*moreguinensis* sp. nov.	4.8–5.5	11+1	PNG: Central
18	*nadjae* sp. nov.	4.8–5.1	0+0	IN: Papua: Puncak, Yahukimo
19	*neoguineensis* (Zimmermann, 1919)	5.2–6.9	6+(0–1)	IN: Papua: Biak Numfor, Yapen Islands, Mamberano Raya, Sarmi, Jayapura, Yalimo, Pegunungan Bintang; PNG: Sandaun, East Sepik, Madang, WHL, EHL, Morobe, East New Britain
20	*oksibilensis* sp. nov.	5–6.1	0+0	IN: Papua: Pegunungan Bintang; PNG: Western
21	*pseudoneoguineensis* sp. nov.	5.2–5.85	0+0	IN: Papua: Nabire, Jayawijaya
22	*pseudooksibilensis* sp. nov.	4.9–5.6	(0–9)+0	IN: West Papua: Teluk Wondama; Papua: Nabire
23	*rajaampatensis* sp. nov.	4.4–5.6	(10–11)+(0–1)	IN: West Papua: Raja Ampat: Batanta, Salawati, Waigeo; Sorong
24	*rouaffer* sp. nov.	4.8–5.5	11+1	IN: Papua: Puncak
25	*rugosus* sp. nov.	4.15–4.7	11+1	IN: West Papua: Teluk Wondama, Kaimana; Papua: Puncak, Nabire
26	*sandaunensis* sp. nov.	5.1–6	11+1	PNG: Sandaun
27	*sarmiensis* sp. nov.	4.95–5.2	11+1	IN: Papua: Sarmi
28	*securiformis* sp. nov.	5.3	6+1	IN: Papua: Puncak
29	*testegensis* sp. nov.	5.1	6+1	IN: West Papua: Manokwari
30	*toricelli* sp. nov.	5.7	10+1	PNG: Sandaun
31	*vagauensis* sp. nov.	6.1–7	11+1	PNG: Morobe
32	*wanggarensis* sp. nov.	4.9	11+1	IN: Papua: Nabire

The diagnostic characters of the group are mainly those of median lobe:

- median lobe of aedeagus with dorsal and ventral sclerites not separated medially, more or less pressed together;
- dorsal and ventral sclerites each divided into two lobes in apical half;
- dorsal sclerite more strongly sclerotised than partly membranous ventral one, left ventral lobe with a differently developed lateral sclerotised area;
- lobes of dorsal and ventral sclerites can be differently shaped; apex of right dorsal lobe often “swollen”, pea-shaped;
- left lobe of dorsal sclerite without distinct surface sculpture, smooth;
- paramere of narrow triangular form or with narrowing subapical part;
- elytron with number of striae and degree of their development very variable among species and within one species: (0–11)+(0–1); usual patterns 0+0, 11+1 and 6+(0–1).

The species of the group can be divided into the complexes based on shape of lobes of the dorsal sclerite and on shape of the sclerotised area of the left lobe of the ventral sclerite.

#### ﻿1. Austrelatus adelbert

Taxon classificationAnimaliaColeopteraDytiscidae

(Megna, Atthakor, Manaono, Hendrich & Balke, 2017) comb. nov.

403F20D6-F179-587A-B69A-53737961415B

Figs 14 , 18 , 82

Copelatus adelbert Megna, Atthakor, Manaono, Hendrich & Balke, 2017: 50.

##### Type locality.

Papua New Guinea: Madang Province, Adelbert Range, Keki birdwatching area, 04°42'21.5"S, 145°25'15.4"E.

##### Type material.

***Holotype***: male “Papua New Guinea: Madang, Adelbert Mts., Sewan – Keki, 700 m, 04°42'215"S, 145°25'154"E, 4.v.2006, leg Balke & Manaono (PNG 51)”, “HOLOTYPE Copelatusadelbert sp. n. Det.: Y. S. Megna, W. Atthakor, M. Manaono, L. Hendrich & M. Balke 2017” [red], “HOLOTYPUS” (ZSM).

***Paratypes***: 1 male, 1 female with the same label as the holotype and with “PARATYPE Copelatusadelbert sp. n. Det.: Y. S. Megna, W. Atthakor, M. Manaono, L. Hendrich & M. Balke 2017” [red] (ZSM). 2 males “Papua New Guinea: Madang, Adalbert [sic!] Mts., Keki, 850 m, 4.v.2006, nr 04.42.300S 145.25.089E, Balke & Manaono (PNG 52)” (ZMS). 3 males, 3 females “Papua New Guinea: Madang, Adalbert [sic!] Mts., below Keki, 790 m, 5.v.2006, 04.42.300S 145.25.089E, Balke & Manaono (PNG 53)” (ZMS). 1 male, 2 females “Papua New Guinea: Madang, Adalbert [sic!] Mts., creek nr Keki, 790 m, 26.xi.2006, 04.42.300S 145.25.089E, Binatang Boys leg. (PNG 53a)” (ZMS).

##### Additional material.

1 male “3204” [green label], “Papua New Guinea: Madang, Adalbert [sic!] Mts., Keki, 850 m, 4.v.2006, nr 04.42.300S 145.25.089E, Balke & Manaono (PNG 52)” (ZMS). 1 male “Papua New Guinea: Madang, Adalbert [sic!] Mts., 400 m, 29.xi.2006, 04.43.058S 145.24.437E, Binatang Boys (PNG 119)” (ZMS). 3 males, 1 female “Papua New Guinea: Madang, Keki, Adalbert [sic!] Mts., 500 m, 29.xi.2006, 04.43.058S 145.24.437E, Balke & Kinibel (PNG 118)” (NHMW, ZMS).

##### Diagnosis.

For complete description, see Megna et al. (2017: 51–53).

**Figures 14–17.**
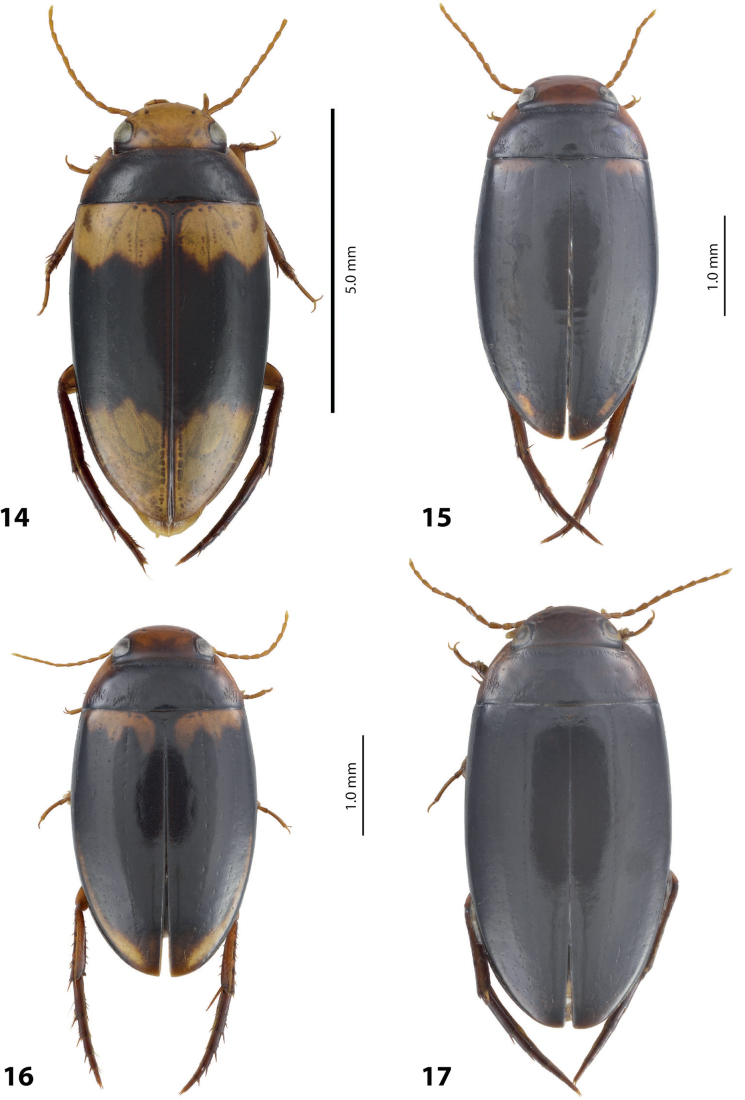
Habitus and colouration of **14***Austrelatusadelbert* (Megna et al., 2017) **15***A.lembenensis* sp. nov. **16***A.nadjae* sp. nov. **17***A.pseudoneoguineensis* sp. nov.

**Figure 18.**
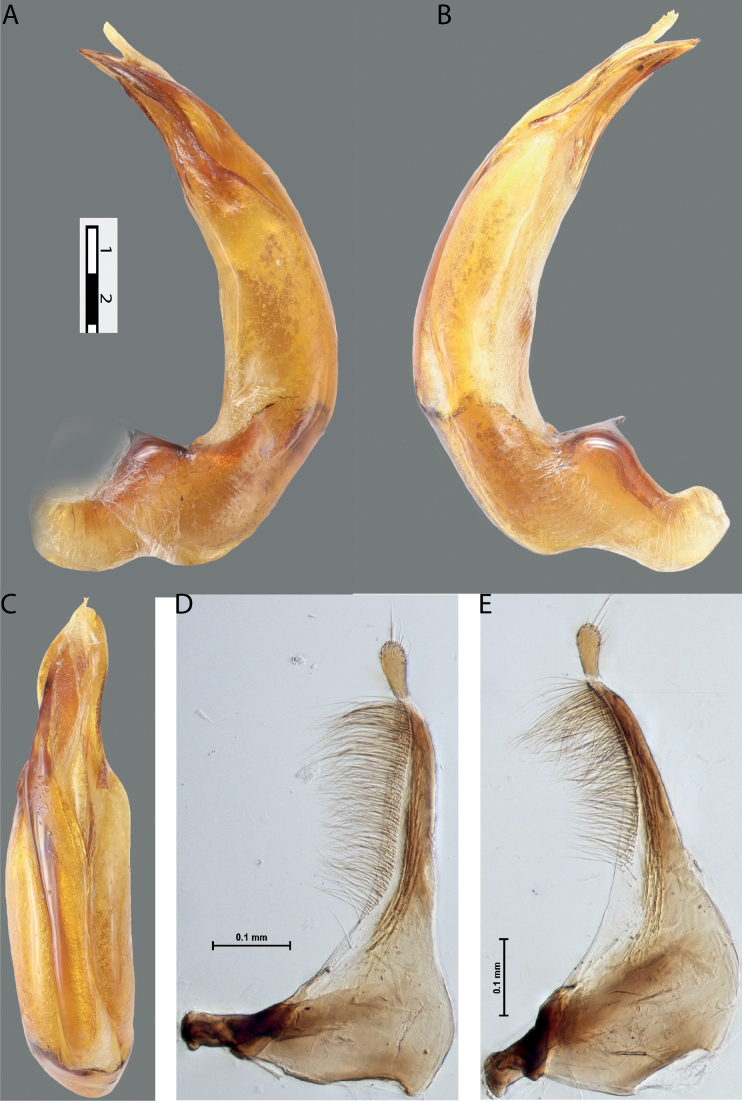
*Austrelatusadelbert* (Megna et al., 2017), median lobe **A** left lateral view **B** right lateral view **C** ventral view **D** left paramere in external view **E** right paramere in internal view. Scale bar: one unit – 0.1 mm (**A–C**).

Beetle medium-sized: TL 5.9–6.4 mm, TL-H 5.5–5.9 mm, with oblong-oval habitus of continuous outline. Dorsal colouration very distinct: yellow to orange head, black pronotum with yellow to orange lateral bands, elytron with broad basal and apical yellow to orange parts. Elytron without striae and strioles in both sexes. Proclaws relatively long, slender, equal in in length. Median lobe of aedeagus relatively broad; its dorsal and ventral sclerites almost equal in length, relatively thin apically, pointed; ventral sclerite weakly sclerotised: its right lobe membranous, long, and usually sticking out ventrally, with small lateral sclerotised area; left ventral lobe shorter, almost completely but weakly sclerotised, without a lateral sclerotised area. Paramere of narrow triangular form, more or less evenly tapering to apex, with setae not divided into distal and proximal, slightly sparser and shorter proximally (Figs 14, 18).

##### Affinities.

Due to its dorsal colouration and smooth elytra, the species cannot be mixed up with any other NG species. In addition, it differs from all other representatives of the *A.neoguineensis* group in less modified sclerites of the median lobe and less sclerotised ventral sclerite (left ventral lobe without lateral sclerotised area).

##### Distribution.

New Guinean endemic. Papua New Guinea: Madang Province, Adelbert Range (Fig. 82).

##### Habitat.

The species is collected from small puddles along forest creeks; the ground was red clay with sand, gravel and sometimes leaves (Megna et al. 2017).

#### ﻿2. Austrelatus baliem sp. nov.

Taxon classificationAnimaliaColeopteraDytiscidae

65351CDD-0A68-521E-BE84-34A95D50C3F2

https://zoobank.org/BEB089C2-3E74-459B-AD7A-CAD1A00FBE28

Figs 58 , 62 , 84 , 85–86

##### Type locality.

Indonesia: Papua Province: Jayawijaya Regency, Baliem Valley, Wamena, 1600 m a.s.l.

##### Type material.

***Holotype***: male “W.-Neuguinea/Baliem Valley Wamena (Ort), 1600 m /IR 1&6 31.8. & 6.9.1990 leg: Balke & Hendrich” (ZSM).

***Paratypes***: IN: Papua: Jayawijaya Regency: 13 males, 1 female “INDONESIA, Papua: Jayawijaya Distr.; Baliem valley, 15km NE of Wamena, forested gorge, Jiwika → salt spring 03°57.2-5'S, 138°57.2-6'E, 1715–2015 m; 7.ii.2015 J.Hájek & J.Šumpich leg.” (NHMW, NMPC). 1 male “INDONESIA, Papua: Jayawijaya Distr. Baliem valley, 15km NE of Wamena, forested gorge, Jiwika → salt spring 03°57.2-5'S, 138°57.2-6'E, 1715–2015 m; J.Hájek & J.Šumpich leg., 7.ii.2015”, “7288” [green text] (ZSM). 1 female “Indonesia: Papua, Jayawijaya Dist., Baliem valley 15km NE Jiwika, 1715–2015 m, 7.ii.2015, 03°57.2-5'S 138°57.2-6'E J. Hájek & J. Šumpich ()” (NMPC). 2 males “INDONESIA, Papua: Jayawijaya Distr.; Baliem valley, 14 km NNE of Wamena, wetland & gardens nr. Jiwika (Kurulu), 1660 m 03°58.0-5'S, 138°55.8-56.3'E, J.Hájek & J.Šumpich leg., 6.ii.2015” (NMPC). 9 males, 7 females “INDONESIA, Papua: Jayawijaya Distr.; Baliem valley, 10km NE of Wamena, forest above “Baliem valley resort”04°03.6'S, 139°01.9'E, 2050 m; 2–3.ii.2015 J.Hájek & J.Šumpich leg.” (MZB, NHMW, NMPC). 1 male “INDONESIA, Papua: Jayawijaya Distr.; Baliem valley, 10km NE of Wamena, forest above “Baliem valley resort” 04°03.6'S, 139°01.9'E, 2050 m J.Hájek & J.Šumpich leg., 2–3.ii.2015” (NMPC). 1 male “3351” [green label], “Indonesia: Papua, Jiwika, xi.2007, Riedel.”, “24.XI.2007 1875 m” [hw] (ZSM). 1 male “4231” [green label], “Indonesia: Papua, Wasior [crossed out, Shaverdo] Riedel () xi.2007 Jiwika [hw, Shaverdo]” (ZSM). 3 males, 9 females “Indonesia: Jiwika, 03°57.161'S 138°57.357'E, 1875 m, sifted, 11.vii.2010, Riedel” (NHMW, ZSM). 1 male, 1 female “Indonesia: W. Papua, Balem valley, Kurulu, (Jiwika), 1875 m, 24.XI.2007, 03°57.161'S 138°57.357'E, A. Riedel leg.” (ZSM).

##### Description.

***Body size and form***: Beetle small to medium-sized, oblong-oval to elongate (Fig. 58).

***Measurements***: TL 4.65–5.6 mm, TL-H 4.2–5.1 mm, MW 2.1–2.7 mm, TL/MW 2.07–2.17; PL 0.7–0.8 mm, PW 1.9–2.3 mm, PL/PW 0.35–0.37; DBE 0.9–1 mm, DBE/PW 0.44–0.47.

Holotype: TL 4.9 mm, TL-H 4.4 mm, MW 2.25 mm, TL/MW 2.17; PL 0.75 mm, PW 2.05 mm, PL/PW 0.37; DBE 0.95 mm, DBE/PW 0.46.

***Colouration***: Dorsally piceous, with brownish head and sides of pronotum (Fig. 58).

Head reddish brown to dark brown, narrowly darker posterior to eyes. Pronotum piceous on disc and reddish brown to dark brown on sides. Elytron piceous, sometimes brownish basally and apically and near suture. Scutellum piceous. Antennae, other head appendages, and pro- and mesolegs reddish brown to dark brown, metalegs darker, especially distally. Venter dark brown to piceous, with paler prosternum. Teneral beetles paler.

***Surface sculpture***: Elytron with 11 complete, not strongly impressed dorsal striae; submarginal stria present or absent: 11+(0–1). Dorsal surface with strong microreticulation, submatt (Fig. 58).

Head without strioles, with dense punctation (spaces between punctures 1–3× size of punctures); punctures coarse (diameter of punctures equal to diameter of microreticulation cells or larger than it); head with a row of coarse setigerous punctures along inner margin of each eye and a short row at frontal angle of each eye; a slightly longer puncture row forms fronto-clypeal depression at each head side; microreticulation strongly impressed. Pronotum with numerous strioles, often also on disc; pronotal punctation slightly finer than on head; setigerous punctures form a row along pronotal margins, absent in posterior middle; disc of pronotum with indistinct longitudinal median scratch. Pronotum with strong microreticulation. Elytron with 11 striae; striae 1, 5, 7, 9 sometimes shortly reduced or interrupted basally and apically, stria 9 sometimes interrupted or reduced to few striae; stria 10 can be shortly reduced basally; seldom small, solitary strioles present between striae; submarginal stria present or absent, often interrupted. Elytron with distinct punctation and strong microreticulation. Ventral part with fine punctation, invisible on metaventrite and metacoxae and weak on abdominal ventrites; prosternum smooth medially; metaventrite and metacoxae with distinct microreticulation; on abdominal ventrites microreticulation slightly finer; metacoxal plates with numerous, distinctly impressed longitudinal strioles, abdominal ventrites 1 and 2 with numerous, long, longitudinal strioles from margin to margin, on abdominal ventrites 3 and 4 strioles situated laterally and turn to middle, almost horizontal, abdominal ventrite 5 without strioles, abdominal ventrite 6 with distinct punctation that sparse medially and forms a dense, rugose lateral area at each side, with few lateral strioles.

***Structures***: Head large and broad. Pronotum relatively long; lateral margins distinctly convergent anteriorly. Base of prosternum rounded anteriorly, convex medially; blade of prosternal process rather narrow.

***Male***: Protibia straight, not modified. Proclaws long, subequal. Median lobe of aedeagus with two lobes of dorsal sclerite rather narrow; left dorsal lobe subequal in length to right one, with a long, narrow lateral crest in left lateral view; apex of left dorsal lobe straight, broadly pointed, dorsally with indistinct, fine surface structure (scales); right dorsal lobe with small, slightly curved downwards apex; ventral sclerite membranous: left ventral lobe without lateral sclerotised area. Paramere of narrow triangular form, with setae not clearly divided into distal and proximal; more distally situated setae slightly denser and longer than more proximal ones, with several the most proximal setae standing separately (Fig. 62).

***Female***: As male.

##### Variability.

There is an insignificant variation in the colouration and dorsal striation described above.

##### Affinities.

The species is very distinct due to its sub-matt dorsal surface, 11+(0–1) elytron striae, almost uniform dark brown to piceous colouration and shape of the median lobe.

##### Etymology.

The species is named after Baliem River Valley. The name is a noun in the nominative singular, standing in apposition.

##### Distribution.

New Guinean endemic. Indonesia: Papua Province: Jayawijaya Regency (Fig. 84).

##### Habitat.

All specimens were collected in small, shallow puddles near or on trekking paths, rich in rotten leaves and twigs. The specimens from Jiwika, close to the salt springs, were collected in very shady pools in the forest; pools were filled with rotten leaves (Fig. 85). The specimens from gardens close to Jiwika were mostly collected in a sun exposed irrigation canal close to road; there they co-habited with *Exocelinabaliem* Shaverdo, Hendrich & Balke, 2013 (Fig. 86). The specimens from the Baliem Valley Resort were found at night, during a rain, crawling in the thin film of water flowing on the forest path.

#### ﻿3. Austrelatus bormensis sp. nov.

Taxon classificationAnimaliaColeopteraDytiscidae

FE70A69D-4940-5272-B0F0-FD7ACA9ED36B

https://zoobank.org/151B1D04-02AC-454B-806E-0F3D645A440D

Figs 32 , 35 , 82

##### Type locality.

Indonesia: Papua Province, Pegunungan Bintang Regency, near Borme, 04°24'S, 140°25'E, 900 m a.s.l.

##### Type material.

***Holotype***: male “IRIAN JAYA 12.8.1992 Zentralmassiv, Borme 04°24'S, 140°25'E 900 m, leg. Balke (8)” (ZSM).

***Paratypes***: IN: Papua: Pegunungan Bintang Regency: 10 males, 4 females with the same label as the holotype (MZB, NHMW, ZSM). 9 males, 4 females “Borme-Omban 13.8.1992 leg. Balke (9)” (NHMW). 2 males “IR 92#9: West New Guinea, Borme, 1200 m, 13.viii.1992 leg., Balke” (NHMW). 2 males, 2 females “Indonesia: Papua, Omban, 23.xi.2016, P.J.A. de Vries (Pap75)” (ZSM). 1 male “IRIAN JAYA Zentralmassiv 140°25'E 04°24'S”, “Kali Takime, 1000 m, 15.8.1992 leg. Balke (14)” (NHMW).

Yalimo Regency: 2 males “INDONESIA: West Papua, Jayawijaya Prov., Elelim, 450–750 m, 17.xii.2004, Riedel”, one of them with an additional green label “1551” (ZSM).

##### Description.

***Body size and form***: Beetle medium-sized, with oblong-oval to elongate habitus (Fig. 32).

***Measurements***: TL 5.4–6.1 mm, TL-H 4.8–5.5 mm, MW 2.5–3.05 mm, TL/MW 2–2.16; PL 0.8–0.9 mm, PW 2.2–2.6 mm, PL/PW 0.34–0.36; DBE 0.95–1 mm, DBE/PW 0.39–0.43.

Holotype: TL 6.1 mm, TL-H 5.5 mm, MW 3.05 mm, TL/MW 2; PL 0.9 mm, PW 2.6 mm, PL/PW 0.35; DBE 1 mm, DBE/PW 0.39.

***Colouration***: Dorsally piceous, with reddish head, pronotal sides, and short basal band and apical spot on elytron (Fig. 32).

Head reddish brown, darker narrowly behind eyes. Pronotum brown, with paler towards sides, almost yellowish red on them. Elytron dark brown to piceous, often with reddish yellow to reddish brown short band on middle of elytral base, formed by confluence of two spots, which seldom present as almost separate, vague spots; band of different length but not reaching suture and lateral elytral margin; rather broad and distinct or narrow and vague; elytron with distinct, elongate apical spot. Scutellum brown to piceous. Antennae and other head appendages yellowish brown. Legs reddish yellowish brown proximally and darker distally, especially metalegs. Venter mostly brownish, with paler prosternum. Teneral beetles paler.

***Surface sculpture***: Elytron with 5–10 more or less complete dorsal striae, submarginal stria usually absent: (5–10)+(0–1) (Fig. 32).

Head without strioles, with relatively dense, distinct punctation (spaces between punctures 1–2× size of punctures); punctures relatively coarse (diameter of punctures equal to diameter of microreticulation cells); head with a row of coarse setigerous punctures along inner margin of each eye and a short row at frontal angle of each eye; a slightly longer puncture row forms fronto-clypeal depression at each head side; microreticulation distinct but not strong. Pronotum usually with distinct, numerous strioles, occupying whole pronotum, sparse and finer on disc and coarse and dense laterally from it, especially at posterior margin; pronotal punctation finer than on head; setigerous punctures form a row along pronotal margins, absent in posterior middle, generally less distinct because of strioles; disc of pronotum with indistinct longitudinal median scratch. Pronotal microreticulation rather weakly impressed. Pronotum often with numerous strioles in females. Elytron with 5–10 dorsal striae, usually with six complete striae and interrupted striae between them; very seldom with strong reduction of striae, to five incomplete striae; submarginal striae usually absent. Elytron with very fine, inconspicuous punctation and very weak microreticulation. Elytron often with numerous strioles in females. Ventral part with fine, inconspicuous punctation, invisible on metaventrite and metacoxae and weak on abdominal ventrites; prosternum smooth medially; metaventrite and metacoxae with weak microreticulation; on abdominal ventrites microreticulation almost invisible; metacoxal plates with short, numerous longitudinal strioles, abdominal ventrites 1 and 2 with numerous, long, longitudinal strioles from margin to margin, on abdominal ventrites 3 and 4 strioles situated laterally and turn to middle, almost horizontal, abdominal ventrites 5 and 6 without strioles but with rather distinct punctation that sparser medially and forms a dense, rugose lateral area at each side.

***Structures***: Head relatively broad. Pronotum short and broad; lateral margins distinctly convergent anteriorly. Base of prosternum narrowly rounded anteriorly, convex medially; blade of prosternal process relatively broad, convex in middle.

***Male***: Protibia not straight, thinner proximally and broader medially and distally due to its curved ventral margin. Proclaws relatively long, equal. Median lobe of aedeagus with two lobes of dorsal sclerite broad, almost straight, slightly curved downwards apically; right dorsal lobe slightly longer and broader than left one, with a broad but pointed apex and large median membranous impression in right lateral view; in left lateral view, left dorsal lobe with a lateral longitudinal crest. Lobes of ventral sclerite weakly sclerotised laterally (visible in left and right lateral views), mostly membranous, subequal, straight apically; sclerotised part of left ventral lobe long, with thin, straight apex (in left lateral view). Paramere with setae not divided into distal and proximal (Fig. 35).

***Female***: Most of the females have numerous thin, longitudinal strioles on elytra and pronotum that densely cover dorsal surface and make it matt.

##### Variability.

The species demonstrates insignificant variation in its dorsal colouration and strong variation in elytral striation described above.

##### Affinities.

Based on general appearance and shape of the median lobe, the species is close to *A.neoguineensis*. But differs from it in smaller size, unstable number of elytral striae: (5–10)+(0–1), in *A.neoguineensis*: 6+(0–1), pronotum with strioles, and shape of median lobe sclerites.

##### Etymology.

The species is named after its type locality, Borme. The name is an adjective in the nominative singular.

##### Distribution.

New Guinean endemic. Indonesia: Papua Province: Pegunungan Bintang and Yalimo regencies (Fig. 82).

##### Habitat.

The species was collected in stream-side puddles.

#### ﻿4. Austrelatus brazza sp. nov.

Taxon classificationAnimaliaColeopteraDytiscidae

4A266689-6464-5E1A-992A-64DAB4FF8F5C

https://zoobank.org/1FC96C7C-8022-46A8-824D-EE4175888340

Figs 29 , 30 , 33 , 82 , 91 , 92

##### Type locality.

Indonesia: Papua Province: Yahukimo Regency, Dekai, upper Brazza River, 04°44'27.9"S, 139°39'15.2"E, 273 m a.s.l.

##### Type material.

***Holotype***: male “Indonesia: Papua, Dekai, upper Brazza, 273 m, 2./3.vi.2015, -4,7410 139,6542, Sumoked (Pap044)” (MZB).

***Paratypes***: 12 males, 15 females with the same label as the holotype (MZB, KSP, NHMW, ZSM). 9 males, 3 females “Indonesia: Papua, Dekai, upper Brazza, 273 m, 2./3.vi.2015, -4,7410, -139,6542, Sumoked (Pap044)”, three males with additional green text labels “7218”, “7219” and “7220” (NHMW, ZSM).

##### Description.

***Body size and form***: Beetle small, with oblong-oval habitus (Figs 29, 30).

***Measurements***: TL 4.95–5.6 mm, TL-H 4.5–5.1 mm, MW 2.4–2.8 mm, TL/MW 2.06–2, PL 0.75–0.9 mm, PW 2.1–2.5 mm, PL/PW 0.36–0.38; DBE 0.85–0.9 mm, DBE/PW 0.4–0.41.

Holotype: TL 5.1 mm, TL-H 4.7 mm, MW 2.5 mm, TL/MW 2.04; PL 0.8 mm, PW 2.25 mm, PL/PW 0.36; DBE 0.9 mm, DBE/PW 0.41.

***Colouration***: Dorsally brown, with reddish head, pronotal sides, and elytral basal and apical spots (Figs 29, 30).

Head yellowish red to reddish brown, usually dark brown behind eyes. Pronotum gradually dark brown medially and reddish laterally, with reddish yellow sides, sometimes also with two symmetrical reddish yellow spots at anterior margin. Elytron reddish brown to dark brown, with two reddish yellow spots at middle of base, not reaching lateral margin, spots vague and confluent, bigger or smaller; seldom with a small indistinct spot at suture; at apex with very narrow yellow spot, sometimes indistinct or developed into distinct lateral line reaching middle of elytron. Scutellum reddish brown. Antennae and other head appendages yellow. Pro- and mesolegs yellow proximally and darker distally, metalegs reddish yellow, darker distally. Venter reddish brown, with paler prosternum.

***Surface sculpture***: Elytron without or with 6–10 dorsal striae, submarginal stria absent, (0–10)+0 (Figs 29, 30).

Head without strioles, with relatively sparse punctation (spaces between punctures 2–3× size of punctures); punctures relatively fine, coarser medially (diameter of punctures smaller or equal to diameter of cells of microreticulation); head with a row of coarse setigerous punctures along inner margin of each eye and a very short row of punctures at frontal angle of each eye; a slightly longer puncture row forms fronto-clypeal depression at each head side; head with strong microreticulation. Pronotum with or without strioles and with thin, rather inconspicuous longitudinal wrinkles at posterior margin; pronotal punctation slightly finer than on head; coarse setigerous punctures form a broad row along pronotal margins, absent in posterior middle; disc of pronotum with a short, thin, longitudinal median scratch. Pronotal microreticulation rather weakly impressed on disc. Elytron with 0–10 dorsal striae, submarginal stria absent. Specimens without elytral stria have three well-developed puncture lines and sometimes some punctures in between. In specimens with elytral striae, 6-stria pattern observed: sometimes stria 1 reduced to very small strioles in apical elytral half and elytron appears to have only five striae; sometimes additional, usually incomplete striae present between six complete striae and elytron appears to have up to ten striae. Elytron with fine punctation; microreticulation weak. Ventral part with extremely fine, scarce, inconspicuous punctation, invisible on metaventrite and metacoxae and more distinct on abdominal ventrites; prosternum smooth medially; metaventrite and metacoxae with distinct microreticulation; on abdominal ventrites microreticulation almost invisible; metacoxal plates with more or less short almost longitudinal strioles, abdominal ventrites 1 and 2 with numerous, long, longitudinal strioles from margin to margin, on abdominal ventrites 3 and 4 strioles situated laterally and turn to middle, almost horizontal, abdominal ventrites 5 and 6 without strioles but with rather distinct punctation that sparser medially and forms a dense lateral area at each side.

***Structures***: Head relatively broad. Pronotum short and broad; lateral margins distinctly convergent anteriorly. Base of prosternum rounded anteriorly, slightly convex medially; blade of prosternal process small, convex in middle.

***Male***: Protibia straight, not modified. Proclaws long, subequal in length, anterior claw more strongly curved downwards than posterior due to median incision of its inner margin. Median lobe of aedeagus with two lobes of dorsal sclerite rather narrow; lobes of dorsal sclerite subequal: right dorsal lobe slightly longer than left one; in lateral view, apex of left dorsal lobe more or less straight, with longitudinal crest and weak but distinct incision on its lateral margin; right dorsal lobe with weakly developed, inconspicuous median impression (in right lateral view) and rounded apex. Left lobe of ventral sclerite with its sclerotised area large, very strongly sclerotised, distinctly shorter than right ventral lobe; its apex bilobed: left part short, broad, and rounded, right one long, thin, hooked; this sclerotised area hidden under right ventral lobe and between left and right lobes of dorsal sclerite, usually invisible (only hook’s apex can be visible) in left lateral view. Paramere with setae divided into distal and proximal; proximal setae distinctly sparser and shorter than ones distal, especially in left paramere (Fig. 33).

***Female***: As male. There are no striolated matt forms.

##### Variability.

The species is so far known from one population of Brazza area only. Within it, there is insignificant variation in colouration and strong variation in elytral striation described above.

##### Affinities.

Based on shape of the median lobe, the species is close to *A.neoguineensis*. In shape of its median lobe sclerites, it is very similar and most likely closely related to *A.pseudooksibilensis* sp. nov. and especially to *A.oksibilensis* sp. nov. From *A.pseudooksibilensis* sp. nov., it differs by less extended yellow colouration of the elytra (absence of dorsal band), and weaker incision on the apex of the left lobe of the dorsal sclerite of the median lobe, as well as females without elytral strioles. The differences from *A.oksibilensis* sp. nov. see below.

##### Etymology.

The name refers to the Brazza River, in upper reaches of which this species was collected. The species name is a noun in the nominative singular standing apposition.

##### Distribution.

New Guinean endemic. Indonesia: Papua Province: Yahukimo Regency. The species is known only from the type locality (Fig. 82).

##### Habitat.

At the type locality, it was collected in puddles or pools among rotten leaves and twigs (Figs 91, 92).

#### ﻿5. Austrelatus debulensis sp. nov.

Taxon classificationAnimaliaColeopteraDytiscidae

2E24E1FC-5529-59FD-A3F3-69ADE0364CE2

https://zoobank.org/8CD3CFF6-F115-48E6-9009-9148731ACDDC

Figs 76 , 80 , 84 , 91 , 92

##### Type locality.

Indonesia: Papua Province: Yahukimo Regency, NE Dekai, upper Brazza River, Debula Village, 4°44'27.9"S, 139°39'15.2"E, 273 m a.s.l.

##### Type material.

***Holotype***: male “Indonesia: Papua, Dekai, upper Brazza, 273 m, 2./3.vi.2015, -4,741084724 139,654211075976, Sumoked (Pap044)” (MZB).

***Paratypes***: 11 males, 10 females, with the same label as the holotype, two males with additional green text labels “7222” and “7223” (MZB, NHMW, ZSM).

##### Description.

***Body size and form***: Beetle small, with oblong-oval habitus (Fig. 76).

***Measurements***: TL 4.1–4.5 mm, TL-H 3.75–4.05 mm, MW 2–2.1 mm, TL/MW 2.05–2.14; PL 0.6–0.65 mm, PW 1.7–1.8 mm, PL/PW 0.35–0.36; DBE 0.7–0.75 mm, DBE/PW 0.41–0.42.

Holotype: TL 4.45 mm, TL-H 4 mm, MW 2.1 mm, TL/MW 2.12; PL 0.65 mm, PW 1.8 mm, PL/PW 0.36; DBE 0.75 mm, DBE/PW 0.42.

***Colouration***: Dorsally piceous, with reddish head and pronotum, basal band and apical spot on elytron (Fig. 76).

Head yellowish red to brown, seldom darker, slightly darker narrowly behind eyes. Pronotum yellowish red to reddish brown, paler on sides, sometimes darker at anterior and posterior margins, seldom with dark brown disc. Elytron dark brown to piceous, not concolourous with head and pronotum, with yellowish red to reddish brown basal band usually from stria 1 till stria 11, seldom shorter; with slightly notched posterior margin; vague so that elytron seems to be paler basally; elytron with distinct or vague, elongate spot apically. Scutellum yellowish red to brown, usually concolourous with elytra. Antennae and other head appendages yellow. Pro- and mesolegs yellow and metalegs yellowish red proximally and darker distally. Venter mostly reddish brown, with yellowish red prosternum.

***Surface sculpture***: Elytron with 11 complete or interrupted dorsal striae; submarginal stria present: 11+1 (Fig. 76).

Head without strioles, with rather dense punctation (spaces between punctures 1–3× size of punctures); punctures relatively large (diameter of punctures equal to or slightly larger than diameter of cells of microreticulation); head with a row of setigerous punctures along inner margin of each eye and a short row at frontal angle of each eye; a slightly longer puncture row forms fronto-clypeal depression at each head side; head with relatively strong microreticulation. Pronotum with several strioles mainly posteriorly and laterally, seldom medially; with longitudinal wrinkles at posterior margin; pronotal punctation finer and sparser than on head; setigerous punctures form a broad row along pronotal margins, absent in posterior middle; disc of pronotum with thin, longitudinal median scratch. Pronotal microreticulation fine to distinct. Elytron with 11 dorsal striae; odd striae shortly reduced apically; stria 10 and sometimes stria 1 shortly reduced basally, striae 1–3, 9, 10 sometimes interrupted; submarginal stria present, weakly developed, often apical or interrupted, reaching maximally ½ of elytron. Elytron with fine punctation and microreticulation fine to distinct. Ventral part with fine, inconspicuous punctation, slightly visible on metaventrite and metacoxae and stronger on abdominal ventrites; prosternum smooth medially; metaventrite and metacoxae with distinct microreticulation; on abdominal ventrites microreticulation almost invisible; metacoxal plates with short, numerous, rather sparse, distinctly impressed longitudinal strioles, abdominal ventrites 1 and 2 with numerous, long, longitudinal strioles from margin to margin, on abdominal ventrites 3 and 4 strioles situated laterally and turn to middle, almost horizontal, abdominal ventrites 5 and 6 without strioles but with fine punctation that sparser medially and forms a dense lateral area at each side.

***Structures***: Head relatively broad. Pronotum short and broad; lateral margins distinctly convergent anteriorly. Base of prosternum rounded anteriorly, convex medially; blade of prosternal process relatively narrow, convex in middle.

***Male***: Protibia straight, not modified. Proclaws simple, relatively short, subequal. Median lobe of aedeagus with two lobes of dorsal sclerite rather narrow; left lobe distinctly shorter than right one; in lateral left view, left dorsal lobe with a lateral crest interrupted into apical and basal parts, lobe concave along rather short basal crest; apex of left dorsal lobe relatively long, more or less straight; its dorsal surface without denticulation, but with a strong median crest visible in left lateral view; right dorsal lobe with well-developed, more ventrally situated median impression in right lateral view, with broad, “swollen”, rounded apex. Lobes of ventral sclerite weakly sclerotised laterally, visible in left and right lateral views, mostly membranous, subequal (right part can be protruding), straight apically; sclerotised part of left ventral lobe long, thin, and straight apically, 2/3 of length of left dorsal lobe. Paramere with setae divided into distal and proximal; proximal setae distinctly sparser and shorter than ones distal, especially in left paramere (Fig. 80).

***Female***: Females have coarser and denser dorsal punctation and more strongly impressed microreticulation, often also with numerous, tiny elytral strioles instead of punctures. However, there are no strongly striolated matt forms.

##### Variability.

There is a strong variation in the colouration and dorsal striation described above.

##### Affinities.

The species is very similar to *A.rugosus* sp. nov., differential diagnosis see under that species.

##### Etymology.

The species is named after Debula Village. The name is an adjective in the nominative singular.

##### Distribution.

New Guinean endemic. Indonesia: Papua Province: Yahukimo Regency. The species is known only from the type locality area (Fig. 84).

##### Habitat.

At the type locality, it was collected in puddles or pools among rotten leaves and twigs (Figs 91, 92).

#### ﻿6. Austrelatus fakfak sp. nov.

Taxon classificationAnimaliaColeopteraDytiscidae

319606F2-532F-58CE-BF36-34F37F1A1F51

https://zoobank.org/07AB9EAD-7553-4F0D-AAF1-C95F28FB2374

Figs 45 , 49 , 83

##### Type locality.

Indonesia: West Papua Province: Fakfak Regency, 4 km N Fakfak, Kali Mati, 02°53'52.5"S, 132°18'23.8"Е, 260 m a.s.l.

##### Type material.

***Holotype***: male “West New Guinea/Fak-Fak/IR 27 Kali Mati, 4km N of Fak-Fak 260 m, 8. & 9.8.1991 leg: Balke & Hendrich” (ZSM).

***Paratypes***: IN: West Papua: Fakfak Regency: 4 males, 2 females, same label data as holotype, one male with hand-written label “Baumhöhlen-Art ex Fak-Fak” (CLH, MZB, NHMW). 17 males, 6 females “Indonesia: Irian Jaya Barat, Fak Fak, 310 m, 23.ii.2006, 2.53.756S 132 18.074E, Tindige, (FakFak)” (MZB, NHMW, ZSM). 1 male “Indonesia: Irian Jaya Barat, Fak Fak, 310 m, 23.ii.2006, Tindige”, “1322” [green label] (ZSM).

Kaimana Regency: 1 male “Indonesia: W-Papua vic. Kaimana, road 18 km NE 3°31'11"S, 133°40'15"E, 50–80 m 21.-25.II.2011 leg. A. Skale (014)”, “4429” [green label] (ZSM). 1 male, 1 female “INDONESIA: W-Papua ca. 50km SE Kaimana, Triton bay, vic. Kamaka village, trail to Kamakawalar lake, S3°48'22"/E134°14'02", 50–100 m, 03.II.2011 leg. A Skale (006a)”, “4428” [green label] and “4437” [green text], respectively (ZSM). 4 males, 3 females “INDONESIA: W-Papua 50km SE Kaimana, Triton bay, vic. Kamaka vill. trail to Kamakawalar lake, 3°48'22"S 134°14'02"E", 50–100 m, 03.II.2011 leg. A Skale (006a) small pool” (CAS, NHMW).

##### Description.

***Body size and form***: Beetle small, with oblong-oval to elongate habitus (Fig. 45).

***Measurements***: TL 4.4–5 mm, TL-H 4–4.55 mm, MW 2.1–2.5 mm, TL/MW 2–2.23; PL 0.6–0.7 mm, PW 1.8–2.1 mm, PL/PW 0.33–36; DBE 0.8–0.95 mm, DBE/PW 0.44–0.45.

Holotype: TL 4.9 mm, TL-H 4.3 mm, MW 2.2 mm, TL/MW 2.23; PL 0.7 mm, PW 1.95 mm, PL/PW 0.36; DBE 0.85 mm, DBE/PW 0.44.

***Colouration***: Dorsally piceous, with reddish yellow head, pronotal sides, and two basal and one apical spot on elytron (Fig. 45).

Head yellowish red to reddish brown, darker narrowly behind eyes. Pronotum reddish brown to dark brown on disc and paler towards sides, yellowish red to reddish brown on them. Sometimes only anterior and posterior margins medially dark. Elytron dark brown to piceous, with two yellow to reddish brown spots on elytral base: one, larger, between striae 2 and 4 and second, smaller, between striae 4 and 6; sometimes these spots distinct, sometimes vague, sometimes completely reduced, seldom confluent; elytron with more or less distinct elongate apical spot, seldom absent. Scutellum reddish brown to piceous. Antennae, other head appendages, and legs proximally yellow; legs darker distally, yellowish red, especially metalegs. Venter yellow, with paler prosternum. Teneral beetles paler.

***Surface sculpture***: Elytron with six complete dorsal striae; submarginal stria present: 6+1 (Fig. 45).

Head without strioles, with sparse punctation (spaces between punctures 1–4× size of punctures); punctures relatively fine (diameter of punctures usually equal to diameter of microreticulation cells); head with a row of coarse setigerous punctures along inner margin of each eye and a short row at frontal angle of each eye; a slightly longer puncture row forms fronto-clypeal depression at each head side; microreticulation strong. Pronotum usually with several strioles laterally or only in posterolateral angles; with fine longitudinal wrinkles at posterior margin; pronotal punctation finer than on head; setigerous punctures form a row along pronotal margins, absent in posterior middle; disc of pronotum with indistinct longitudinal median scratch. Pronotum with distinct microreticulation, sometimes finer. Elytron with 6 dorsal striae; striae 1–4 and 6 complete, stria 5 usually shortly reduced basally, stria 1 very seldom shortly reduced basally; submarginal striae present. Elytron with distinct punctation and microreticulation, microreticulation sometimes finer. Ventral part with fine, inconspicuous punctation, invisible on metaventrite and metacoxae and weak on abdominal ventrites; prosternum smooth medially; metaventrite and metacoxae with weak microreticulation; on abdominal ventrites microreticulation almost invisible; metacoxal plates with numerous, finely impressed longitudinal strioles, abdominal ventrites 1 and 2 with numerous, long, longitudinal strioles from margin to margin, on abdominal ventrites 3 and 4 strioles situated laterally and turn to middle, almost horizontal, abdominal ventrites 5 and 6 without strioles but with fine punctation that sparser medially and forms a dense, rugose lateral area at each side.

***Structures***: Head broad. Pronotum short and broad. Base of prosternum broadly rounded anteriorly, slightly convex medially; blade of prosternal process small, convex in middle.

***Male***: Protibia straight, not modified. Proclaws simple, subequal in length: anterior claw very slightly shorter than posterior one. Median lobe of aedeagus with two lobes of dorsal sclerite rather narrow; left dorsal lobe distinctly shorter that right one, with weak, long lateral crest and apex evenly curved downwards and to left, dorsally with distinct denticulation (spinulae) visible also in lateral left view; right dorsal lobe with small, indistinct, elongate median impression and modified apex: relatively large, slightly swollen, broadly rounded; left lobe of ventral sclerite with its sclerotised area large, rounded apically, shorter than right ventral and dorsal lobes and slightly more than 1/2 length of left dorsal lobe. Paramere with setae not clearly divided into distal and proximal, especially in left paramere; more distally situated setae slightly denser and longer than more proximal ones, with several the most proximal setae standing separately (Fig. 49).

***Female***: As male.

##### Variability.

There is a variation in the colouration, elytral striolation, dorsal microreticulation described above.

##### Affinities.

In general shape of median lobe, especially in shape of the sclerotised area of left ventral lobe and in shape of the lateral crest of the left dorsal lobe, the species is similar to *A.testegensis* sp. nov., *A.manokwariensis* sp. nov., and *A.wanggarensis* sp. nov. The species can be distinguished from them by its small, elongate habitus, elytron colouration, 6+1 elytral striae and shape of the median lobe; see more under the other species descriptions. *Austrelatustestegensis* sp. nov. is the most similar in the shape of median lobe sclerites to *A.fakfak* sp. nov.

##### Etymology.

The species is named for its Type locality. The name is a noun in the nominative standing in apposition.

##### Distribution.

New Guinean endemic. Indonesia: West Papua: Fakfak and Kaimana regencies (Fig. 83).

##### Habitat.

All seven specimens sampled in 1991 near Fakfak were collected in a shaded and water-filled tree hollow in primary lowland rainforest.

#### ﻿7. Austrelatus febrisauri sp. nov.

Taxon classificationAnimaliaColeopteraDytiscidae

28613DFC-B4A3-5CF4-AFC6-0BFE77138065

https://zoobank.org/5B682DD1-87DF-4085-99AC-8A6541838091

Figs 22 , 26 , 82 , 87

##### Type locality.

Indonesia: Papua Province: Nabire Regency, road Nabire-Enarotali, 65^th^ km, 250 m a.s.l.

##### Type material.

***Holotype***: male “Irian Jaya: Paniai Prov. road Nabire – Ilaga, km 65, 29.8.1996, 250 m leg. M. Balke (96 # 6)” (ZSM).

***Paratypes***: 29 males, 19 females with the same label as the holotype, one male additionally with a red type label “PARATYPE Copelatus speciosus sp. nov. des. Balke 1997” (MZB, NHMW, ZSM). 1 male “West New Guinea/Paniai Prov./IR 24 track Nabire-Ilaga km 54 Basecamp, 750 m, 25.7.1991 leg: Balke & Hendrich” (CLH). 1 male “IR 11” [hw] (ZSM). 2 males “Ir 23-W. New Guinea, track Nabire-Ilaga KM 62, 250 m, 24.vii.1991 Balke & Hendrich leg.” (ZSM). 2 females “West New Guinea/Paniai Prov./IR 22 track Nabire-Ilaga km 62, 250 m, 24.7.1991, forest pools leg: Balke & Hendrich” (CLH, ZSM).

##### Description.

***Body size and form***: Beetle small, with oblong-oval habitus (Fig. 22).

***Measurements***: TL 4.7–5.35 mm, TL-H 4.3–4.9 mm, MW 2.3–2.6 mm, TL/MW 2.04–2.06; PL 0.7–0.8 mm, PW 2–2.25 mm, PL/PW 0.33–0.36; DBE 0.85–0.95 mm, DBE/PW 0.42–0.43.

Holotype: TL 5.2 mm, TL-H 4.7 mm, MW 2.55 mm, TL/MW 2.04; PL 0.75 mm, PW 2.25 mm, PL/PW 0.33; DBE 0.95 mm, DBE/PW 0.42.

***Colouration***: Dorsally brown to piceous, with reddish yellow to brown head, pronotal sides, and basal band on elytron as well as elytral apical spot (Fig. 22).

Head reddish yellow to brown, darker behind eyes. Pronotum yellowish brown to piceous on disc and reddish yellow to brown on sides. Elytron brown to piceous, with reddish yellow to brown basal band of different length, usually almost reaching suture and lateral elytral margin; its anterior margin reaching elytron basally and its posterior margin wavy but not notched; apically elytron with distinct, reddish yellow to brown, elongate apical spot. Scutellum yellowish to piceous. Antennae and other head appendages yellowish brown. Pro- and mesolegs yellowish brown proximally and darker distally, especially metalegs. Venter yellowish brown to brown, with paler prosternum. Teneral beetles paler.

***Surface sculpture***: Elytron without striae: 0+0, but with distinct puncture lines.

Head without strioles, with relatively dense, even punctation (spaces between punctures 1–3× size of punctures); punctures relatively coarse (diameter of punctures equal to diameter of cells of microreticulation); head with a row of coarse setigerous punctures along inner margin of each eye and a short row at frontal angle of each eye; a slightly longer puncture row forms fronto-clypeal depression at each head side; sometimes head with large shallow median depression; microreticulation rather weak. Pronotum with strioles, usually only few, at posterior angles, seldom along posterior margin, and thin, longitudinal wrinkles mostly at middle of posterior margin; pronotal punctation finer than on head; coarse setigerous punctures form a broad row along pronotal margins, absent in posterior middle; disc of pronotum with thin, longitudinal median scratch. Pronotal microreticulation rather weakly impressed on disc. Elytron without elytral striae but with two distinct puncture lines on disc and one less distinct laterally; two additional lines of very sparse, coarse setigerous punctures can be seen between elytral lines; elytron with very fine, inconspicuous punctation; microreticulation weak. Ventral part with fine, inconspicuous punctation, invisible on metaventrite and metacoxae and weak on abdominal ventrites; prosternum smooth medially; metaventrite and metacoxae with weak microreticulation; on abdominal ventrites microreticulation almost invisible; metacoxal plates with short longitudinal strioles, abdominal ventrites 1 and 2 with numerous, long, longitudinal strioles from margin to margin, on abdominal ventrites 3 and 4 strioles situated laterally and turn to middle, almost horizontal, abdominal ventrites 5 and 6 without strioles but with rather distinct punctation that sparser medially and forms a dense lateral area at each side.

***Structures***: Head relatively broad. Pronotum short and broad; lateral margins distinctly convergent anteriorly. Base of prosternum narrowly rounded anteriorly, convex medially; blade of prosternal process elongate, narrow, convex in middle.

***Male***: Protibia straight, not modified. Proclaws relatively short, subequal in length. Median lobe of aedeagus with two lobes of dorsal sclerite rather narrow; right dorsal lobe slightly longer than left one; in lateral view, apex of left dorsal lobe more or less straight, with weak longitudinal crest on its lateral margin; right dorsal lobe with narrow median impression (in right lateral view) and flat, relatively broad apex. Left lobe of ventral sclerite with its sclerotised area weak, indistinct laterally, shorter than right ventral lobe; lobes of ventral sclerite pressed together; right ventral lobe its sclerotised area well-developed; apex of right ventral lobe straight. Paramere with setae not clearly divided into distal and proximal; more distally situated setae slightly denser than more proximal ones (Fig. 26).

***Female***: With pronotal strioles usually occupying entire lateral sides and stronger elytral punctation.

##### Affinities.

The new species co-occurs with *A.pseudooksibilensis* sp. nov., to which it is very similar in size, colouration and surface structures; see the comparision under the latter species. Also, *A.pseudoneoguineensis* sp. nov. is known from the area of the road Nabire-Enarotali, and *A.febrisauri* sp. nov. is probably more closely related to this species than to *A.pseudooksibilensis* sp. nov. based on the structure of their median lobes, shape of the paramere, and shape of the proclaws. However, the new species can be easily distinguished from *A.pseudoneoguineensis* sp. nov. by its smaller size and more prominent yellow elytral colouration.

##### Etymology.

The species name is Latin noun in the genitive, which means “of gold fever”, and refers to the intensive gold mining activity in the region where the species was collected.

##### Distribution.

New Guinean endemic. Indonesia: Papua Province: Nabire Regency. The species is known only from the Nabire-Ilaga area (Fig. 82).

##### Habitat.

All specimens were collected in shallow (up to 20 cm water depth), shaded or at least partly shaded forest pools and puddles of different size, rich in rotten leaves and twigs, as, for example, a small forest pool at kilometre 62 of the Nabire-Ilaga track (Fig. 87). Few specimens were also found in water-filled track hollows on forest tracks.

#### ﻿8. Austrelatus fojaensis sp. nov.

Taxon classificationAnimaliaColeopteraDytiscidae

0CD5B2DD-A173-5760-9B02-5526C4C02450

https://zoobank.org/0FE2B423-B4D9-44FA-BF0C-E956AB670257

Figs 44 , 48 , 83 , 89

##### Type locality.

Indonesia: Papua Province: Sarmi Regency, Foja Mts, Tor Atas, Waaf Village, 2°34'18.6"S, 138°43'02.1"E, 1,700 m a.s.l.

##### Type material.

***Holotype***: male “Indonesia: Papua, Foja Mountains, bog camp, 1700 m, 23.v.-3.vi.2016,”, “-2 571839 138.717250, Sumoked (Pap058)” (MZB).

***Paratypes***: 1 male, 1 female with the same label as the holotype, the female with an additional green text label “7359” (KSP). 1 male, 4 females male “Indonesia: Papua, Sarmi area 70 m, 25.ix.2014, -1.9713 138.8491, UNCEN team (Pap032)” (MZB, ZSM).

##### Description.

***Body size and form***: Beetle small to medium-sized, with oblong-oval to elongate habitus (Fig. 44).

***Measurements***: TL 5.1–5.5 mm, TL-H 4.6–4.9 mm, MW 2.5–2.6 mm, TL/MW 2.04–2.16; PL 0.8–0.85 mm, PW 2.1–2.3 mm, PL/PW 0.37–0.39; DBE 0.9–1 mm, DBE/PW 0.43–0.46.

Holotype: TL 5.4 mm, TL-H 4.9 mm, MW 2.5 mm, TL/MW 2.16; PL 0.85 mm, PW 2.2 mm, PL/PW 0.39; DBE 1 mm, DBE/PW 0.46.

***Colouration***: Dorsally piceous, with reddish brown head, yellowish red pronotal sides and three basal spots and one apical spot connected to a short, narrow lateral band on elytron (Fig. 44).

Head reddish brown to brown, darker narrowly behind eyes. Pronotum reddish brown to dark brown on disc and paler towards sides, yellowish red on them. Sometimes only anterior and posterior margins medially dark. Elytron dark brown to piceous, with three rather vague yellowish red spots on elytral base: one near stria 1; second between striae 2 and 4 and third between striae 4 and 6 more distinct; these spots almost confluent; elytron with distinct elongate apical spot connected to a short (almost whole apical 1/2 of elytron), narrow lateral band. Scutellum reddish brown. Antennae, other head appendages, and legs proximally yellowish; legs darker distally, yellowish brown, especially metalegs. Venter yellowish brown, with paler prosternum. Teneral beetles paler.

***Surface sculpture***: Elytron with six dorsal striae; submarginal stria present: 6+1 (Fig. 44).

Head without strioles, with rather dense punctation (spaces between punctures 1–3× size of punctures); punctures relatively coarse (diameter of punctures equal to diameter of microreticulation cells or larger than it); head with a row of coarse setigerous punctures along inner margin of each eye and a short row at frontal angle of each eye; a slightly longer puncture row forms fronto-clypeal depression at each head side; microreticulation strong. Pronotum usually with several strioles in posterolateral angles; with fine longitudinal wrinkles at posterior margin; pronotal punctation finer than on head; setigerous punctures form a row along pronotal margins, absent in posterior middle; disc of pronotum with indistinct longitudinal median scratch. Pronotum with fine microreticulation. Elytron with 6 dorsal striae; striae 2–4 and 6 complete, stria 1 shortly reduced basally; stria 5 reduced (ca. 1/5 of elytron length) and interrupted basally; tiny strioles can be present basally between striae 1 and 2; submarginal striae present, sometimes interrupted. Elytron with fine punctation and microreticulation. Ventral part with fine, inconspicuous punctation, invisible on metaventrite and metacoxae and weak on abdominal ventrites; prosternum smooth medially; metaventrite and metacoxae with weak microreticulation; on abdominal ventrites microreticulation almost invisible; metacoxal plates with numerous, weakly impressed longitudinal strioles, abdominal ventrites 1 and 2 with numerous, long, longitudinal strioles from margin to margin, on abdominal ventrites 3 and 4 strioles situated laterally and turn to middle, almost horizontal, abdominal ventrites 5 and 6 without strioles but with fine punctation that sparser medially and forms a dense, rugose lateral area at each side.

***Structures***: Head broad. Pronotum relative long; lateral margins distinctly convergent anteriorly. Base of prosternum narrowly rounded anteriorly, slightly convex medially; blade of prosternal process rather small, narrow, convex in middle.

***Male***: Protibia almost straight, not modified. Proclaws simple, rather long, subequal in length. Median lobe of aedeagus with two lobes of dorsal sclerite rather narrow; left dorsal lobe distinctly shorter that right one, with a lateral crest interrupted into apical and basal parts; apex of left dorsal lobe distinctly curved downwards, dorsally with denticulation (spinulae) invisible in lateral left view due to strong curvature downwards; right dorsal lobe with small, indistinct, elongate median impression and an angular convexity above it; apex of right dorsal lobe large, swollen, rounded; left lobe of ventral sclerite with its sclerotised area rather large, broad, slightly concave, rounded apically, shorter than right ventral and dorsal lobes and 1/2 length of left dorsal lobe. Paramere with setae not clearly divided into distal and proximal; more distally situated setae denser than more proximal ones (Fig. 48).

***Female***: As male. There are no striolated, matt forms.

##### Variability.

There is an insignificant variation in the body size, colouration and dorsal striolation.

##### Affinities.

In general shape of median lobe, especially in shape of the sclerotised area of left ventral lobe and in shape of the lateral crest of the left dorsal lobe, the species is similar to *A.innominatus* sp. nov. and *A.rouaffer* sp. nov. The species can be distinguished from them by its elytron colouration, 6+1 elytral striae, simple, rather long male proclaws and median lobe sclerites.

##### Etymology.

The species is named after Foja Mts. The name is an adjective in the nominative singular.

##### Distribution.

New Guinean endemic. Indonesia: Papua Province: Sarmi Regency (Fig. 83).

##### Habitat.

At the type locality, the species was collected in a large swampy area – a bog in mountain rainforest (Fig. 89).

#### ﻿9. Austrelatus garainensis sp. nov.

Taxon classificationAnimaliaColeopteraDytiscidae

167A474C-CF0B-5EE6-A0FD-FFAE17AAA106

https://zoobank.org/47004E0C-CB26-4DB3-8F3C-4BCD1E2DA174

Figs 60 , 64 , 83

##### Type locality.

Papua New Guinea: Morobe Province, Garaina, 07°51.032'S, 147°07.007'E, 720 m a.s.l.

##### Type material.

***Holotype***: male “Papua New Guinea: Garaina, 720 m, vi.2008, 07.51.032S 147.07.007E Ibalim & Sosanika PNG216” (ZSM).

***Paratypes***: *PNG: Morobe*: 122 males, 96 females with the same label as the holotype (BMNH, NHMW, ZSM). For additional paratypes see Appendix 1.

##### Description.

***Body size and form***: Beetle medium-sized, with oblong-oval habitus (Fig. 60).

***Measurements***: TL 5.4–6.2 mm, TL-H 4.6–5.9 mm, MW 2.7–3.25 mm, TL/MW 2–2.07; PL 0.8–0.9 mm, PW 2.3–2.7 mm, PL/PW 0.33–0.35; DBE 0.9–1.05 mm, DBE/PW 0.39.

Holotype: TL 6.2 mm, TL-H 5.6 mm, MW 3 mm, TL/MW 2.07; PL 0.85 mm, PW 2.55 mm, PL/PW 0.33; DBE 1 mm, DBE/PW 0.39.

***Colouration***: Dorsally piceous, with yellowish red head, pronotal sides and on elytron with three yellowish red basal spots or a posteriorly notched basal band, one apical spot, and often with narrow lateral band (Fig. 60).

Head yellow to reddish brown, piceous narrowly behind eyes. Pronotum dark brown to piceous on disc and paler towards sides, broadly yellow to reddish brown on them. Elytron piceous, with three rather distinct, yellow to reddish brown spots on elytral base: one between striae 1 and 3, second between striae 5 and 7 and third between striae 9 and 11; usually two latter spots confluent forming a posteriorly notched basal band; first spot sometimes vague or absent, seldom confluent with second; elytron with a distinct, elongate, small to large apical spot; sometimes a narrow lateral band present, confluent with apical spot. Scutellum yellow to brown. Antennae, other head appendages, and pro- and mesolegs proximally yellow, distally yellowish red; metalegs yellowish red, darker distally. Venter with yellowish red prosternum, dark brown meso- and metaventrites and metacoxae, and reddish brown abdominal ventrites; abdominal ventrites 5 and 6 with a yellowish red lateral spot on each side. Teneral beetles paler.

***Surface sculpture***: Elytron with 11 distinct, complete dorsal striae; submarginal stria present: 11+1 (Fig. 60).

Head without strioles, with rather dense punctation (spaces between punctures 1–3× size of punctures); punctures relatively fine (diameter of punctures equal to diameter of microreticulation cells); head with a row of coarse setigerous punctures along inner margin of each eye and a short row at frontal angle of each eye; a slightly longer puncture row forms fronto-clypeal depression at each head side; microreticulation weak. Pronotum with several weak strioles on sides or with numerous distinct strioles covering even disc, with numerous, fine longitudinal wrinkles at posterior margin; pronotal punctation finer and denser than on head; setigerous punctures form a row along pronotal margins, absent in posterior middle; disc of pronotum with indistinct longitudinal median scratch. Pronotum with fine microreticulation. Elytron with 11 distinctly impressed dorsal striae; striae complete, not reduced basally; striae 1, 3, 5, 7, and 9 reduced apically; submarginal striae present, long, well-developed, reaching ½ or more of elytron. Elytron with fine punctation and microreticulation. Ventral part with fine, inconspicuous punctation, invisible on metaventrite and metacoxae and weak on abdominal ventrites; prosternum smooth medially; metaventrite and metacoxae with weak microreticulation; on abdominal ventrites microreticulation almost invisible; metacoxal plates with numerous, strongly impressed longitudinal strioles, abdominal ventrites 1 and 2 with numerous, long, longitudinal strioles from margin to margin, on abdominal ventrites 3 and 4 strioles situated laterally and turn to middle, almost horizontal, abdominal ventrites 5 and 6 without strioles but with fine punctation that very sparse medially and forms a dense, rugose lateral area at each side.

***Structures***: Head relatively broad. Pronotum short and broad; lateral margins distinctly convergent anteriorly. Base of prosternum broadly rounded anteriorly, convex medially; blade of prosternal process long, narrow, convex in middle.

***Male***: Protibia straight, not modified. Proclaws rather long, subequal in length. Median lobe of aedeagus with two lobes of dorsal sclerite rather narrow; left dorsal lobe distinctly shorter that right one, with long lateral crest; apex of left dorsal lobe curved downwards and not to left; its dorsal surface without denticulation, it invisible in left lateral view due to strong curvature downwards; right dorsal lobe with distinct, but shallow, elongate median impression; apex of right dorsal lobe elongate, less swollen, rounded; lobes of ventral sclerite almost completely sclerotised, only with narrow membranous areas medially; sclerotised area of left ventral lobe as long as left dorsal lobe, with long, thin slightly curved to left apex, well visible in lateral left and ventral views; right ventral lobe with large sclerotised area, flat, not protruding, not covering left ventral lobe. Paramere with setae distinctly divided into distal and proximal; proximal setae sparser and shorter than distal (Fig. 64).

***Female***: Dimorphic: with elytron punctate as in males or elytron with denser punctation and additionally to it with very tiny strioles between striae. There are no strongly striolated, matt forms. The striolated females very seldom, ratio: 26:2 in PNG220; 91:5 in PNG116; 46:11 in PNG117.

##### Variability.

There is a variation in the body size, colouration and dorsal striolation. Pronotum with numerous strioles is characteristic for the specimens from Central Province (PNG183). These specimens are also the smallest ones.

##### Affinities.

In body shape, elytral striolation and dorsal colouration, the species is similar to *A.vague* sp. nov. and *A.kaszabi*. The species can be distinguished from them by shape of its median lobe sclerites, especially by the strongly sclerotised ventral sclerite and the sclerotised area of the left ventral lobe very well visible in lateral left and ventral views, with its apex curved to left like a slight hook.

##### Etymology.

The species is named after Garaina Village. The name is an adjective in the nominative singular.

##### Distribution.

New Guinean endemic. The species is widespread in PNG: Morobe, EHL, Central, National Capital District, and Milne Bay provinces (Fig. 83).

##### Habitat.

Unknown.

#### ﻿10. Austrelatus innominatus sp. nov.

Taxon classificationAnimaliaColeopteraDytiscidae

FAD2C195-4E08-54BF-AEDA-ABC3DF3CB09E

https://zoobank.org/C490555F-C2B1-428A-ACB9-5A03BBA78FF0

Figs 52 , 53 , 56 , 84 , 87

##### Type locality.

Indonesia: Papua Province, Nabire Regency, road Nabire – Ilaga, 62^nd^ km, 250 m a.s.l.

##### Type material.

***Holotype***: male “West New Guinea/Paniai Prov./IR 22 track Nabire-Ilaga km 62 250 m, 24.7.1991, forest pools leg: Balke & Hendrich” (ZSM).

***Paratypes***: IN: Papua: Nabire Regency: 7 males, 16 females with the same label as the holotype (CLH, MZB, NHMW, ZSM). Additional paratypes see in the Appendix 1.

##### Description.

***Body size and form***: Beetle small or medium-sized, with oblong-oval to elongate habitus (Figs 52, 53).

***Measurements***: TL 4.7–5.6 mm, TL-H 4.2–4.95 mm, MW 2.3–2.6 mm, TL/MW 2–2.07; PL 0.65–0.8 mm, PW 1.9–2.3 mm, PL/PW 0.34–0.36; DBE 0.8–0.95 mm, DBE/PW 0.4–0.42.

Holotype: TL 5.2 mm, TL-H 4.65 mm, MW 2.6 mm, TL/MW 2; PL 0.8 mm, PW 2.25 mm, PL/PW 0.36; DBE 0.9 mm, DBE/PW 0.4.

***Colouration***: Dorsally piceous, with yellowish red head, narrow pronotal sides and a broad basal band and one apical spot on elytron (Figs 52, 53).

Head yellowish red to reddish brown, darker narrowly behind eyes. Pronotum brown to piceous, narrowly yellowish red on sides or only at anterior angles. Elytron piceous, with a distinct yellow to reddish brown basal band, with notched posterior margin, not reaching lateral margin and suture; elytron with distinct elongate apical spot. Scutellum reddish brown to piceous. Antennae, other head appendages, and pro- and mesolegs proximally yellowish red, metalegs red, legs darker distally. Venter reddish brown. Teneral beetles paler.

***Surface sculpture***: Elytron usually with 10–11 complete dorsal striae, seldom with 6 complete and 4 reduced and interrupted striae; submarginal stria present: (10–11)+1 (Figs 52, 53).

Head without strioles, with rather dense punctation (spaces between punctures 1–3× size of punctures); punctures relatively coarse (diameter of punctures equal to diameter of microreticulation cells or larger than it); head with a row of coarse setigerous punctures along inner margin of each eye and a short row at frontal angle of each eye; a slightly longer puncture row forms fronto-clypeal depression at each head side; microreticulation strong. Pronotum with strioles in posterolateral parts; with fine longitudinal wrinkles at posterior margin; pronotal punctation finer than on head; setigerous punctures form a row along pronotal margins, absent in posterior middle; disc of pronotum with indistinct longitudinal median scratch. Pronotum with fine microreticulation. Elytron usually with 11 complete dorsal striae, striae weakly impressed, especially striae 1–3; stria 1 has tendency to reduction, it often completely or partly reduced or interrupted, especially basally; striae 5, 7, 9 and 10 sometimes interrupted basally; maximal stria reduction to 6 complete and 4 reduced to strioles striae. Elytron with fine punctation and microreticulation. Ventral part with fine, inconspicuous punctation, invisible on metaventrite and metacoxae and weak on abdominal ventrites; prosternum smooth medially; metaventrite and metacoxae with weak microreticulation; on abdominal ventrites microreticulation almost invisible; metacoxal plates with numerous, weakly impressed longitudinal strioles, abdominal ventrites 1 and 2 with numerous, long, longitudinal strioles from margin to margin, on abdominal ventrites 3 and 4 strioles situated laterally and turn to middle, almost horizontal, abdominal ventrites 5 and 6 without strioles but with fine punctation that sparser medially and forms a dense, rugose lateral area at each side.

***Structures***: Head relatively broad. Pronotum short and broad; lateral margins distinctly convergent anteriorly. Base of prosternum broadly rounded anteriorly, convex medially; blade of prosternal process narrow, convex in middle.

***Male***: Protibia almost straight, not modified. Proclaws relatively short, subequal in length; anterior claw slightly more strongly curved downwards than posterior. Median lobe of aedeagus with two lobes of dorsal sclerite rather narrow; left dorsal lobe distinctly shorter that right one, with a lateral crest interrupted into apical and basal parts; apex of left dorsal lobe distinctly curved downwards, dorsally with denticulation (spinulae) invisible in lateral left view due to strong curvature downwards; right dorsal lobe with small, indistinct, elongate median impression and modified apex: swollen, rounded; left lobe of ventral sclerite with its sclerotised area rather large, broad, slightly concave, rounded apically, shorter than right ventral and dorsal lobes and slightly more than 1/2 length of left dorsal lobe. Paramere with setae not clearly divided into distal and proximal; with few, thin the most proximal setae standing separately (Fig. 56).

***Female***: With more strongly striolated pronotum and more distinct dorsal punctation. There are no striolated, matt forms.

##### Variability.

There is a variation in the colouration and dorsal striolation described above. In addition, there is one specimen (locality 96#12) with strong reduction of elytral striae to 6 complete striae only, however, it shows the same dorsal colouration and shape of the male proclaws and median lobe sclerites as the other specimens of *A.innominatus*.

##### Affinities.

In general shape of median lobe, especially in shape of the sclerotised area of left ventral lobe and in shape of the lateral crest of the left dorsal lobe, the species is similar to *A.fojaensis* sp. nov. and *A.rouaffer* sp. nov. The species can be distinguished from them by its dorsal striolation, colouration, and median lobe sclerites.

##### Etymology.

The species name is Latin adjective and means “unnamed” because of the long and unsuccessful attempt to find a name for this species. The name is an adjective in the nominative singular.

##### Distribution.

New Guinean endemic. Indonesia: West Papua Province (Teluk Wondama Regency) and Papua Province (Nabire Regency) (Fig. 84).

##### Habitat.

At the type locality, the species was collected in a small forest pool (Fig. 87). At the Nabire-Ilaga track, all other specimens were collected in similar habitats: in shallow (up to 20 cm water depth), shaded or at least partly shaded forest pools and puddles of different size, rich in rotten leaves and twigs; few specimens were also found in water-filled track hollows on forest tracks.

#### ﻿11. Austrelatus kaszabi

Taxon classificationAnimaliaColeopteraDytiscidae

(Guignot, 1956) comb. nov.

87ACB6E8-87E8-53FF-AD24-FE5989192D1D

Figs 12 , 59 , 63 , 83 , 87 , 90

Copelatus kaszabi Guignot 1956: 52 (orig. descr.); Guignot (1956: 53); Guéorguiev (1968: 9); Guéorguiev & Rocchi (1993: 159); Nilsson & Hájek (2023: 64).

##### Type locality.

Papua New Guinea: Madang Province, Stephansort, 05°26'38.4"S, 145°44'47.8"E. In Guignot (1956: 53), as Stephansort, Astrolabe Bay.

##### Type material.

***Holotype***: male “N. Guinea Biró 97.”, “Stephansort Astrolabe Bai // 18.V. [hw on reverse side]”, “Holotypus 1956 Copelatus Kaszabi Guignot” [red border label, partly hw by Guignot], “Type” [on reverse side], “Dr F. Guignot., 1955 Copelatus Kaszabi n.sp. Holotype ♂” [partly hw by Guignot] (HNHM).

***Paratypes***: 3 males, 1 female “N. Guinea Biró 1898”, “Simbang Huon Golf// IX.17. [hw on reverse side]”, one male and female additionaly with “Paratypus 1956 Copelatus Kaszabi Guignot” [red border label, partly hw by Guignot], 2 males additionaly with “Paratypus 1957 Copelatus Kaszabi Guignot” [red border label, partly hw by Guignot] (HNHM). 1 male “N. Guinea Biró 97.”, “Stephansort Astrolabe Bai”, “Paratypus 1957 Copelatus Kaszabi Guignot” [red border label, partly hw by Guignot], “Staatl. Museum für Tierkunde Dresden” (MTD).

##### Additional material.

See Appendix 1.

##### Description.

***Body size and form***: Beetle small to medium-sized, with oval to oblong-oval habitus (Fig. 59).

***Measurements***: TL 5–6.45 mm, TL-H 4.5–5.8 mm, MW 2.6–3.15 mm, TL/MW 1.92–2.05; PL 0.75–0.9 mm, PW 2.2–2.7 mm, PL/PW 0.33–0.34; DBE 0.9–1.05 mm, DBE/PW 0.38–0.41.

Holotype: TL 5.4 mm, TL-H 4.9 mm, MW 2.8 mm, TL/MW 1.93; PL 0.8 mm, PW 2.35 mm, PL/PW 0.34; DBE 0.9 mm, DBE/PW 0.38.

***Colouration***: Dorsally piceous, with yellowish red head, pronotal sides, and on elytron with a basal band and apical spot, often connected with narrow lateral band (Fig. 59).

Head yellow to reddish brown, piceous narrowly behind eyes. Pronotum dark brown to piceous on disc and paler towards sides, yellow to reddish brown on them, especially towards anterior angles. Elytron piceous, with a distinct yellow to reddish brown basal band (often notched on posterior margin, not reaching suture and lateral margin) due to confluence of three spots on elytral base: one between striae 1 and 2 or 3 sometimes reduced, rarely completely absent; two other spots always well-developed, large and confluent; elytron with a distinct, elongate, small to very large apical spot; usually a narrow lateral band present, confluent with apical spot; sometimes apical spot strongly developed occupying whole apex and protruding as narrow band between striae 4 and 5; rarely strongly reduced together with lateral band. Scutellum yellow to brown. Antennae, other head appendages, and pro- and mesolegs proximally yellow, distally yellowish red; metalegs yellowish red, darker distally. Venter with yellowish red prosternum, dark brown meso- and metaventrites and metacoxae, and reddish brown abdominal ventrites; abdominal ventrites 5 and 6 sometimes with a yellowish red lateral spot on each side. Teneral beetles paler. The holotype is a teneral specimen.

***Surface sculpture***: Elytron with 11 distinct, complete dorsal striae; submarginal stria present: 11+1 (Fig. 59).

Head without strioles, with rather dense punctation (spaces between punctures 1–3× size of punctures); punctures relatively fine (diameter of punctures equal to diameter of microreticulation cells); head with a row of coarse setigerous punctures along inner margin of each eye and a short row at frontal angle of each eye; a slightly longer puncture row forms fronto-clypeal depression at each head side; microreticulation weak. Pronotum with several weak strioles on sides or with numerous distinct strioles; with numerous, fine longitudinal wrinkles at posterior margin; pronotal punctation finer and denser than on head; setigerous punctures form a row along pronotal margins, absent in posterior middle; disc of pronotum with indistinct longitudinal median scratch. Pronotum with fine microreticulation. Elytron with 11 distinct dorsal striae; stria 1 usually and stria 10 sometimes shortly reduced basally; striae 1, 3, 5, 7, and 9 reduced apically; seldom striae 1, 3, 6, and 7 interrupted, especially basally; submarginal striae present, weakly developed, sometimes interrupted, often only apically present. Elytron with fine punctation and microreticulation. Ventral part with fine, inconspicuous punctation, invisible on metaventrite and metacoxae and weak on abdominal ventrites; prosternum smooth medially; metaventrite and metacoxae with weak microreticulation; on abdominal ventrites microreticulation almost invisible; metacoxal plates with numerous, distinctly impressed longitudinal strioles, abdominal ventrites 1 and 2 with numerous, long, longitudinal strioles from margin to margin, on abdominal ventrites 3 and 4 strioles situated laterally and turn to middle, almost horizontal, abdominal ventrites 5 and 6 without strioles but with fine punctation that very sparse medially and forms a dense, rugose lateral area at each side.

***Structures***: Head relatively broad. Pronotum short and broad; lateral margins distinctly convergent anteriorly. Base of prosternum broadly rounded anteriorly, convex medially; blade of prosternal process long, narrow, convex in middle.

***Male***: Protibia straight, not modified. Proclaws rather short, subequal in length. Median lobe of aedeagus with two lobes of dorsal sclerite rather narrow; left dorsal lobe distinctly shorter that right one, with long lateral crest; apex of left dorsal lobe curved downwards and not to left; its dorsal surface without denticulation, invisible in left lateral view due to strong curvature downwards; right dorsal lobe with distinct, but shallow, elongate median impression; apex of right dorsal lobe pea-like “swollen”, rounded; lobes of ventral sclerite weakly sclerotised, with large membranous areas medially; sclerotised area of left ventral lobe shorter than left dorsal lobe, with slightly curved to left apex, hidden under the membranous parts of the left and right ventral lobes; therefore, only slightly visible in lateral left view and invisible in ventral view; right ventral lobe membranous, with small sclerotised area, protruding. Paramere with setae not clearly divided into distal and proximal; more distally situated setae slightly denser and longer than more proximal ones, sometimes with several the most proximal setae standing separately (Figs 12, 63).

***Female***: Dimorphic. Matt form with strioles rare; ratio shiny to with strioles is 5:1 in the locality 33/2013.

##### Variability.

There is a variation in the body size, colouration and insignificantly in dorsal striolation. The median lobes sclerites can vary in length and shape. The specimens with more strongly developed yellow apical spot on elytron are characteristic for New Britain populations and seldom in Madang and Sandaun. In New Britain specimens, sclerotised area of the left ventral lobe seems to be shorter, with its apex more strongly curved.

##### Affinities.

In elytral striolation and dorsal colouration, the species is similar to *A.vague* sp. nov. and especially to *A.garainensis* sp. nov. The species can be distinguished from them by its smaller size, more intensive yellow colouration of the elytron, and sometimes more oval habitus, as well as by shape of its median lobe sclerites, especially by the sclerotised area of the left ventral lobe hidden under the membranous parts of the left and right ventral lobes, not well visible in lateral left view and invisible in ventral view, with its apex curved to left like a slight hook.

##### Distribution.

New Guinean endemic. Literature records: in Guignot (1956: 53), Stephansort, Asrolabe Bay (Madang), paratypes are also from Friedrich-Wilhelmshafen (Madang) and Simbang, Huon Golg (Morobe); repeated by Guéorguiev (1968: 9) and Guéorguiev and Rocchi (1993: 159). Our records show that the species is more widely distributed in NG: It is known from IN: Nabire, Sarmi, and Puncak regencies, and is more numerous in PNG: Sandaun, East Sepik, Madang, and East New Britain provinces (Fig. 83).

##### Habitat.

At the locality PAP028, the species was collected in a large forest stream (Fig. 90); at the Nabire-Ilaga track, in a small forest pool (Fig. 87).

#### ﻿12. Austrelatus lembenensis sp. nov.

Taxon classificationAnimaliaColeopteraDytiscidae

65CFC2B1-8B5B-58D2-A44A-F9927B01EC81

https://zoobank.org/A029A4F6-9339-487C-8068-76E73D2D820E

Figs 15 , 19 , 82

##### Type locality.

Papua New Guinea: East Sepik Province, Lembena, 04°56'58.4"S, 143°56'59.7"E, 198 m a.s.l.

##### Type material.

***Holotype***: male “Papua New Guinea: East Sepik, Lembena, 198 m, 3.ix.2009, 04°56'97.4"S 143°56'99.5"E, Ibalim & Pius (PNG241)” (ZSM).

***Paratypes***: 1 female with the same label as the holotype (ZSM). 1 male, 1 female “Papua New Guinea: East Sepik, Lembena, 198 m, 3.ix.2009, 04 46 [!] 974S 143.56.995E, Ibalim & Pius (PNG243)” (ZSM). 2 males “Papua New Guinea: East Sepik, Lembena, 136 m, 3.ix.2009, 04.56.911S 143.56.870E, Ibalim & Pius (PNG244)” (NHMW, ZSM). 1 male “Papua New Guinea: East Sepik, Lembena, 297 m, 8.ix.2009, 04.57.329S 143.57.297E, Ibalim & Pius (PNG247)”, “6010” (ZSM). 2 females “Papua New Guinea: East Sepik, Lembena, 335 m, 10.ix.2009, 04 56.859S 143 59.375E, Ibalim & Pius (PNG250)” (NHMW, ZSM). 1 male, 2 females “Papua New Guinea: East Sepik, Lembena, 335 m, 10.ix.2009, 04.56.859S 143.57.379E, Ibalim & Pius (PNG251)” (NHMW, ZSM).

##### Description.

***Body size and form***: Beetle small, with rather elongate habitus (Fig. 15).

***Measurements***: TL 5–5.25 mm, TL-H 4.6–4.8 mm, MW 2.5–2.65 mm, TL/MW 1.98–2; PL 0.7–0.8 mm, PW 2.1–2.3 mm, PL/PW 0.33–0.4; DBE 0.9–0.95 mm, DBE/PW 0.41–0.43.

Holotype: TL 5.25 mm, TL-H 4.8 mm, MW 2.65 mm, TL/MW 1.98; PL 0.8 mm, PW 2.3 mm, PL/PW 0.4; DBE 0.95 mm, DBE/PW 0.41.

***Colouration***: Dorsally piceous, with reddish yellow head, pronotal sides, and basal band and apical spot on elytron (Fig. 15).

Head reddish yellow, dark brown behind eyes. Pronotum piceous, slightly paler anteriorly and laterally, with reddish yellow sides. Elytron piceous, slightly paler on disc and darker laterally, with reddish yellow basal band of different length but not reaching suture and lateral elytral margin; its anterior margin reaching elytron basally and its posterior margin not straight, sometimes vaguely notched; elytron with distinct, reddish yellow, elongate apical spot of medium size. Scutellum reddish yellow or piceous. Antennae and other head appendages yellowish brown. Pro- and mesolegs yellowish brown proximally and darker distally, especially metalegs. Venter brown to piceous, with paler prosternum. Teneral beetles paler.

***Surface sculpture***: Elytron without striae: 0+0, but with distinct puncture lines (Fig. 15).

Head without strioles, with relatively dense, even punctation (spaces between punctures 1–3× size of punctures); punctures relatively large (diameter of punctures more or less equal to diameter of cells of microreticulation) but not coarse; head with a row of coarse setigerous punctures along inner margin of each eye and a very short row of less coarser punctures at frontal angle of each eye; a longer puncture row forms fronto-clypeal depression at each head side; a small uneven median depression probably due to some coarser punctures sometimes present; head with strong microreticulation. Pronotum without strioles but with thin longitudinal wrinkles at middle of posterior margin; pronotal punctation slightly finer and sparser than on head; coarse setigerous punctures form a broad row along pronotal margins, absent in posterior middle; disc of pronotum with indistinct short, thin, longitudinal median scratch. Pronotal microreticulation rather weakly impressed on disc. Elytron without elytral striae but with two very distinct puncture lines on disc and one laterally, their coarse punctures contiguous, especially those of discal lines; three additional lines of very sparse, coarse setigerous punctures can be seen between elytral lines and between lateral line and lateral margin; elytron with fine but rather dense and distinct punctation; microreticulation weak. Ventral part with extremely fine, scarce, inconspicuous punctation, invisible on metaventrite and metacoxae and more distinct on abdominal ventrites; prosternum smooth medially; metaventrite and metacoxae with weak microreticulation; on abdominal ventrites microreticulation almost invisible; metacoxal plates with more or less short longitudinal strioles, abdominal ventrites 1 and 2 with numerous, long, longitudinal strioles from margin to margin, on abdominal ventrites 3 and 4 strioles situated laterally and turn to middle, almost horizontal, abdominal ventrites 5 and 6 without strioles but with distinct punctation that sparser medially and forms a very dense lateral area at each side.

***Structures***: Head relatively broad. Pronotum short and broad; lateral margins distinctly convergent anteriorly. Base of prosternum slightly rounded anteriorly, strongly convex medially; neck of prosternal process strongly convex; blade of prosternal process small, strongly convex in middle.

**Figure 19.**
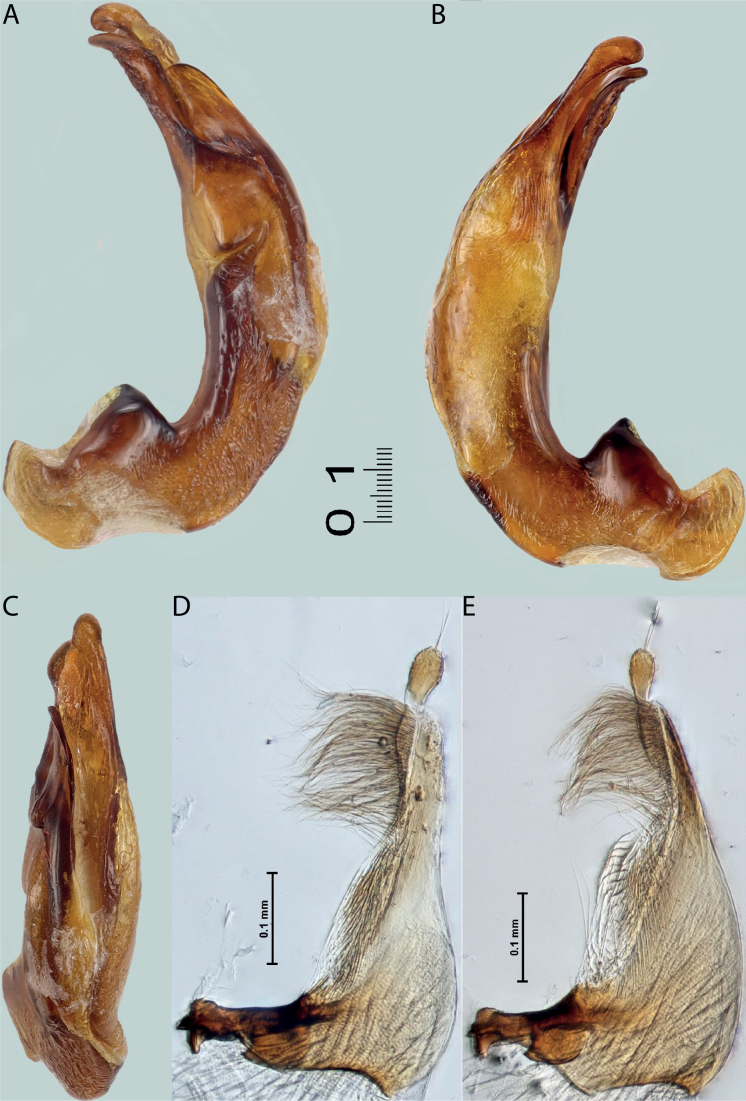
*Austrelatuslembenensis* sp. nov., median lobe **A** left lateral view **B** right lateral view **C** ventral view **D** left paramere in external view **E** right paramere in internal view. Scale bar: one unit – 0.1 mm (**A–C**).

***Male***: Protibia straight, not modified. Proclaws rather short, subequal in length: anterior claw slightly shorter than posterior one. Median lobe of aedeagus with two lobes of dorsal sclerite narrow; right dorsal lobe slightly longer than left one; in lateral view, apex of left dorsal lobe slightly curved downwards, with a small crest; left dorsal lobe dorsally with distinct denticulation (spinulae) visible also in lateral left view; apex of right dorsal lobe swollen, very broadly rounded. Left lobe of ventral sclerite with its sclerotised area visible laterally, well-developed, large, broad, shorter than right ventral lobe; its apex more or less rounded, not hooked. Paramere with setae not clearly divided into distal and proximal; more distally situated setae slightly denser than more proximal ones, sometimes with several the most proximal setae standing separately in right paramere (Fig. 19).

***Female***: Only form with smooth elytra present.

##### Affinities.

In general shape of median lobe, especially in shape of the sclerotised area of left ventral lobe and in shape of the lateral crest of the left dorsal lobe, the species is similar to *A.rouaffer* sp. nov., *A.fojaensis* sp. nov., and *A.innominatus* sp. nov. but it differs from them by elytron without striae, shape of the median lobe sclerites and colouration. In absence of the elytral striae and partly shape of the median lobe, it is similar to *A.nadjae* sp. nov. but differs from it distinctly more elongate habitus, less prominent yellow pattern, and different shape of the median lobe. There are two more species of the group known from Lembena area: *A.neoguineensis* and *A.mirificus* sp. nov. Both these species have distinct elytral striae. *Austrelatusneoguineensis* is larger and has two parts of dorsal sclerite of median lobe broader, with pointed apexes of bird beak-shape. *Austrelatusmirificus* sp. nov. is very similar in colouration and shape of the median lobe to *A.nadjae* sp. nov.

##### Etymology.

The species is named after its type locality, Lembena Village. The name is an adjective in the nominative singular.

##### Distribution.

New Guinean endemic. Papua New Guinea: East Sepik Province, Lembena area (Fig. 82).

##### Habitat.

The species was collected in stream-side puddles.

#### ﻿13. Austrelatus lisae sp. nov.

Taxon classificationAnimaliaColeopteraDytiscidae

66249313-4C0B-51E6-AA1E-83E284036325

https://zoobank.org/4DD58867-9495-4C0A-8A44-C8C848B978FC

Figs 31 , 34 , 82

##### Type locality.

Indonesia: Papua Province, Nabire Regency, 38 km of road Nabire – Ilaga, 150 m a.s.l.

##### Type material.

***Holotype***: male “Irian Jaya: Paniai Prov. road Nabire – Ilaga, km 38 18.9.1996, 150 m, leg. M. Balke (96 # 26)” (ZSM).

***Paratypes***: IN: Papua: Nabire Regency: 2 males, 8 females with the same label as the holotype (MZB, NHMW, ZSM). 2 males “Irian Jaya: Nabire Prov. rd. Nabire – Ilaga, Km 35 Kali Cemara, 100 m, 23.10.1997, leg. Balke (IR97#14)” (NHMW). 1 male “Irian Jaya: Nabire Prov. rd. Nabire – Ilaga, Km 35 Kali Cemara, 100 m, 27.9.1997, leg. Balke (IR97#6)” (NHMW). 1 female “Irian Jaya: Paniai Prov. road Nabire – Ilaga, Km 90, 1.9.1996, 150 m leg. Balke (96 # 11)” (NHMW). 1 male “Indonesia: Papua, Road Nabire-Enarotali KM 80, 250 m, 22.x.2011, 03.33.860S 135.46.473E, UNCEN team (PAP12)”, “5137” [green text] (ZSM). 1 male, 1 female “Irian Jaya: Kabup, Nabire, 30km S Nabire, Kali Cemara, 150 m, 15.8.1998, leg. M. Balke (CE 1)” (NHMW). 1 male “Irian: Nabire – Ikaya, “KM 35”, Seitenstr. nach K. Cemara, 1991, leg. M. Balke” (ZSM).

Sarmi Regency: 2 males, 1 female “Indonesia: Papua, Sarmi, Waaf, N Foja Mts, riverbank, 120 m, 23.ix.2014, -2,3445 138,7395, UNCEN team (Pap030)”, the males with additional green text labels “6457” and “6458” (ZSM). 1 male “Indonesia: Papua, N Foja Mts, Waaf Village, river Tor, 80 m, 4.-7.vi.2016,”, “-2.336679 138.749959, Sumoked (Pap062)” (NHMW). 1 female “Indonesia: Papua, Foja Mountaints N foot, N Waaf vill., pondok, 150 m, 4.-7.vi.2016,”, “-2.0614 138.7439, Sumoked (Pap061)” (ZSM).

Mimika Regency: 1 male “7890” [green text], “Indonesia: Papua, Kabupaten Mimika, Timika, 149 m, 25–30.v.2017”, “-4.252020° 136.643384°, B.Sumoked (Pap68-Bob06)” (ZSM).

##### Description.

***Body size and form***: Beetle medium-sized, with oblong-oval habitus (Fig. 31).

***Measurements***: TL 5.6–6.2 mm, TL-H 5–5.6 mm, MW 2.7–3 mm, TL/MW 2.07, PL 0.8–0.9 mm, PW 2.3–2.6 mm, PL/PW 0.35; DBE 0.95–1 mm, DBE/PW 0.39–0.41.

Holotype: TL 6.2 mm, TL-H 5.6 mm, MW 3 mm, TL/MW 2.07; PL 0.9 mm, PW 2.6 mm, PL/PW 0.35; DBE 1 mm, DBE/PW 0.39.

***Colouration***: Dorsally piceous, with red head, pronotal sides, and elytral basal spots as well as prominent yellow or red lateral band in apical 1/2 of elytron (Fig. 31).

Head yellowish red to red, usually narrowly dark brown behind eyes. Pronotum gradually dark brown to piceous medially and reddish laterally, with yellowish red to red sides. Elytron dark brown to piceous, usually with a reddish yellow to red, narrow band at middle of base, due to confluence of two basal spots; therefore, band sometimes notched at its posterior margin; seldom band reduced into two spots; elytron at apex with elongate but broad yellow or red spot, developed into distinct lateral band reaching middle of elytron, therefore elytron with a prominent apical pattern. Scutellum reddish brown. Antennae and other head appendages yellowish red to yellowish brown. Pro- and mesolegs yellowish red to yellowish brown proximally and darker distally, metalegs darker. Venter reddish brown to brown, with paler prosternum.

***Surface sculpture***: Elytron with six more or less complete dorsal striae; stria 1 usually reduced basally; submarginal stria absent: 6+0 (Fig. 31).

Head without strioles, with relatively sparse punctation (spaces between punctures 1–3× size of punctures); punctures relatively fine, coarser medially (diameter of punctures smaller or equal to diameter of cells of microreticulation); head with a row of coarse setigerous punctures along inner margin of each eye and a very short row of punctures at frontal angle of each eye; a slightly longer puncture row forms fronto-clypeal depression at each head side; head with strong microreticulation. Pronotum with strioles at posterolateral angles and with thin, rather inconspicuous longitudinal wrinkles at posterior margin; pronotal punctation slightly finer and sparser than on head; coarse setigerous punctures form a broad row along pronotal margins, absent in posterior middle; disc of pronotum with a short, thin, longitudinal median scratch. Pronotal microreticulation distinct, rather weakly impressed on disc. Elytron with 6 dorsal striae; stria 1 usually reduced in basal ¼ to ½; striae 2, 4, 6 complete; striae 3 and especially 5 usually reduced or interrupted basally, stria 5 seldom complete; submarginal striae absent, elytron in very seldom specimens with a tiny lateral striole. Elytron with distinct punctation and microreticulation. Elytron seldom with numerous strioles in females. Ventral part with extremely fine, scarce, inconspicuous punctation, invisible on metaventrite and metacoxae and more distinct on abdominal ventrites; prosternum smooth medially; metaventrite and metacoxae with fine microreticulation; on abdominal ventrites microreticulation almost invisible; metacoxal plates with more or less short almost longitudinal strioles, abdominal ventrites 1 and 2 with numerous, long, longitudinal strioles from margin to margin, on abdominal ventrites 3 and 4 strioles situated laterally and turn to middle, almost horizontal, abdominal ventrites 5 and 6 without strioles but with rather distinct punctation that sparser medially and forms a dense lateral area at each side.

***Structures***: Head relatively broad. Pronotum short and broad; lateral margins distinctly convergent anteriorly. Base of prosternum narrowly rounded anteriorly, slightly convex medially; blade of prosternal process small, convex in middle.

***Male***: Protibia almost straight, with only slightly curved ventral margin. Proclaws relatively long, subequal in length, anterior claw more strongly curved downwards than posterior due to basal incision of its inner margin that can be strongly or weakly developed. Median lobe of aedeagus with two lobes of dorsal sclerite narrow; right dorsal lobe distinctly longer than left one; in lateral view, apex of left dorsal truncate and slightly sinuate upwards; right dorsal lobe with well-developed, rather deep median impression (in right lateral view) and rounded apex. Left lobe of ventral sclerite with its sclerotised area very strongly sclerotised, large, slender, long, subequal with left ventral lobe, apically seems slightly hooked due to bent to left; this sclerotised area hidden under right ventral lobe and between left and right lobes of dorsal sclerite, slightly visible (usually hook’s apex) in left lateral view. Paramere with setae not clearly divided into distal and proximal; more distally situated setae slightly denser than more proximal ones, sometimes with few the most proximal setae standing separately (Fig. 34).

***Female***: In elytral striolation similar to males; seldom are there striolated, matt forms: 1 specimen from the locality Pap061.

##### Variability.

The species is so far known from two regions in Papua. The male from Sarmi have more strongly curved anterior proclaw than those of Nabire and slightly differently shaped median lobe sclerites. Also, there is an insignificant variation in the colouration and elytral striolation described above.

##### Affinities.

Based on size and elytral striolation, the species remands *A.neoguineensis* but it has different dorsal colouration and absolutely different shape of the median lobe. Its shape seems to be unique and the species can be easily distinguished from any similar species. However, based on presence of the very strongly sclerotised, hook-like part of the left lobe of ventral sclerite, the species can be put close to *A.brazza* sp. nov., *A.pseudooksibilensis* sp. nov., and *A.oksibilensis* sp. nov., which have the similar structure thought of the different form.

##### Etymology.

The species is named after Lisa, the younger daughter of H. Shaverdo. “Although it is sometimes difficult, I am very happy to have you.” The species name is a noun in the genitive case.

##### Distribution.

New Guinean endemic. Indonesia: Papua Province: Mimika, Nabire and Sarmi regencies (Fig. 82).

##### Habitat.

In Nabire Regency, all specimens were collected in shallow (up to 20 cm water depth), shaded or at least partly shaded forest pools and puddles of different size, rich in rotten leaves and twigs.

#### ﻿14. Austrelatus manokwariensis sp. nov.

Taxon classificationAnimaliaColeopteraDytiscidae

76650272-DDFE-5BD0-A156-CB3FECE80845

https://zoobank.org/32893BEE-916C-4BC7-A844-9FA7484139D1

Figs 46 , 50 , 83

##### Type locality.

Indonesia: West Papua Province: Manokwari Regency, near road Kebar to Aibogar, 0°51'44.6"S, 132°49'47.6"Е, 503 m a.s.l.

##### Type material.

***Holotype***: male “Indonesia: Papua Barat, Kebar to Aibogar, slow forest stream, 503 m, -0,8624 132,8299, UNIPA team (BH025)” (MZB).

***Paratypes***: IN: West Papua: Manokwari Regency: 32 males, 38 females with the same label as the holotype (MZB, NHMW, ZSM). 4 females “Indonesia: Papua Barat, lowland Manokwari, 66 m, 8.v.2015, -0,7433 133,3975, UNIPA team (BH065)” (ZSM). 2 males “Indonesia: Papua Barat, Manokwari, Maripi, swampy stream margin forest, 135 m 28.viii.2014, -0,9075 133,9214, Balke & Panjaitan (BH038)”, “6446” and “6447” [green text] (ZSM). 2 males, 1 female “Indonesia: Papua, Manokwari, 140 m, 8.ii.2006, 00.55.752S 133.54.448E, Tindige (BH 09)”, one male additionally with a green label “4249” (ZSM). 4 females “IN: West Papua: Manokwari Reg., on road Manokwari-Kebar, near Munbrani vill., ca. 600 m, 8.V., 09.15.458S 133.02.389E, roadside ditch (2015-WP36)” (NHMW). 6 males, 1 female “Indonesia: Papua Barat, Manokwari to Kebar, forest stream, 302 m, -0.8005 133.3321, UNIPA team (BH023)” (NHMW, ZSM). 9 males, 8 females “Indonesia: Papua Barat, Kebar to Manokwari, 1h from Kebar, limestone creek and roadside pools,”, “331 m, 8.xi.2013, -0.8013 133.3223, UNIPA team (BH035)”, one male additionally with a green text label “6244” (NHMW, ZSM). 11 males, 11 females “Indonesia: Papua Barat, Kebar Valley, 596 m, 6.v.2015, -0,8406 133,2682, UNIPA team (BH059)” (NHMW, ZSM). 19 males, 12 females “Indonesia: Papua Barat, Fumato, forest stream, 820 m, -0.9042 132.7198, UNIPA team (BH027)”, two males additionally with green text labels “6215” and “6216” (NHMW, ZSM).

Teluk Wondama Regency: 6 males, 5 females “Irian Jaya: Wandammen Bay, Wasior, Sararti 100–200 m, 7.-9.I.2001, leg. A. Riedel” (NMPC, SMNS).

##### Description.

***Body size and form***: Beetle small or medium-sized, with oblong-oval habitus (Fig. 46).

***Measurements***: TL 4.6–5.7 mm, TL-H 4.2–5.2 mm, MW 2.35–2.8 mm, TL/MW 1.96–2.04; PL 0.65–0.85 mm, PW 2–2.4 mm, PL/PW 0.33–35; DBE 0.85–1 mm, DBE/PW 0.42–0.43.

Holotype: TL 5.1 mm, TL-H 4.6 mm, MW 2.5 mm, TL/MW 2.04; PL 0.7 mm, PW 2.15 mm, PL/PW 0.33; DBE 0.9 mm, DBE/PW 0.42.

***Colouration***: Dorsally piceous, with reddish yellow head, pronotal sides, and distinct two or three basal and one apical spot on elytron (Fig. 46).

Head yellowish red to reddish brown, darker narrowly behind eyes. Pronotum reddish brown to piceous on disc and paler towards sides, yellow to reddish brown on them. Sometimes only anterior and posterior margins medially dark. Elytron dark brown to piceous, with two distinct yellow to reddish brown spots on elytral base: one, usually slightly larger, between striae 2 and 4 and second, smaller, between striae 4 and 6; usually these spots confluent forming basal band with notched posterior margin; sometimes third small or rather large, vague spot can be present at stria 1; elytron with distinct elongate apical spot. Scutellum reddish brown to piceous. Antennae, other head appendages, and legs proximally yellow; legs darker distally, yellowish red, especially metalegs. Venter yellow to yellowish red, with paler prosternum. Teneral beetles paler.

***Surface sculpture***: Elytron with six complete dorsal striae; submarginal stria present: 6+1 (Fig. 46).

Head without strioles, with rather dense punctation (spaces between punctures 1–3× size of punctures); punctures relatively fine (diameter of punctures usually equal to diameter of microreticulation cells); head with a row of coarse setigerous punctures along inner margin of each eye and a short row at frontal angle of each eye; a slightly longer puncture row forms fronto-clypeal depression at each head side; microreticulation distinct. Pronotum usually with several strioles in posterolateral angles or without them; with fine longitudinal wrinkles at posterior margin; pronotal punctation finer than on head; setigerous punctures form a row along pronotal margins, absent in posterior middle; disc of pronotum with indistinct longitudinal median scratch. Pronotum with fine microreticulation. Elytron with 6 dorsal striae; striae 1–4 and 6 complete, stria 5 usually shortly reduced basally, stria 1 very seldom shortly reduced basally; submarginal striae present. Elytron with fine punctation and microreticulation. Ventral part with fine, inconspicuous punctation, invisible on metaventrite and metacoxae and weak on abdominal ventrites; prosternum smooth medially; metaventrite and metacoxae with weak microreticulation; on abdominal ventrites microreticulation almost invisible; metacoxal plates with numerous, distinctly impressed longitudinal strioles, abdominal ventrites 1 and 2 with numerous, long, longitudinal strioles from margin to margin, on abdominal ventrites 3 and 4 strioles situated laterally and turn to middle, almost horizontal, abdominal ventrites 5 and 6 without strioles but with fine punctation that sparser medially and forms a dense, rugose lateral area at each side.

***Structures***: Head broad. Pronotum short and broad; lateral margins distinctly convergent anteriorly. Base of prosternum broadly rounded anteriorly, convex medially; blade of prosternal process rather long, narrow, convex in middle.

***Male***: Protibia straight, not modified. Proclaws simple, rather long, subequal in length. Median lobe of aedeagus with two lobes of dorsal sclerite rather narrow; left dorsal lobe shorter that right one, with weak, long lateral crest and apex evenly curved downwards, not really to left, dorsally with distinct denticulation (spinulae) weakly visible in lateral left view due to weak curvation to left; right dorsal lobe with small, indistinct, elongate median impression and modified apex: relatively large, swollen, rounded; left lobe of ventral sclerite with its sclerotised area large, rounded apically, shorter than right ventral and dorsal lobes and slightly more than 1/2 length of left dorsal lobe. Paramere with setae not divided into distal and proximal; more distally situated setae slightly denser than more proximal ones, sometimes with few the most proximal setae standing separately in left paramere (Fig. 50).

***Female***: As male.

##### Variability.

There is an insignificant variation in the colouration, dorsal striolation, and shape of the median lobe.

##### Affinities.

In general shape of median lobe, especially in shape of the sclerotised area of left ventral lobe and in shape of the lateral crest of the left dorsal lobe, the species is similar to *A.fakfak* sp. nov., *A.testegensis* sp. nov., and *A.wanggarensis* sp. nov. The latter can be distinguished from *A.manokwariensis* sp. nov. by 11+1 elytral striae and shape and size of the median lobe sclerites. From *A.fakfak* sp. nov. and *A.testegensis* sp. nov., *A.manokwariensis* sp. nov. differs in distinct maculation of elytron and shape of the median lobe sclerites, especially in apex of the left dorsal lobe more curved downwards than to left.

##### Etymology.

The species is named after Manokwari Regency. The name is an adjective in the nominative singular.

##### Distribution.

New Guinean endemic. Indonesia: West Papua Province: Manokwari and Teluk Wondama regencies (Fig. 83).

##### Habitat.

The species was collected in stream-side puddles and water-filled holes in large boulders, filled with leaves.

#### ﻿15. Austrelatus mimika sp. nov.

Taxon classificationAnimaliaColeopteraDytiscidae

EF26C428-E281-5506-8469-1EFD3645CA6F

https://zoobank.org/33E96E65-A3CF-450D-9838-B0CE76F3664F

Figs 61 , 65 , 84

##### Type locality.

Indonesia: Papua Province: Mimika Regency, 04°30.330'S, 136°46.53'E, 24 m a.s.l.

##### Type material.

***Holotype***: male “Indonesia: Papua, Kabupaten Mimika, 24 m, 25–30.v.2017, 04°30.330'S, 136°46.53'E, B. Sumoked (Pap69-Bob07)” (MZB).

***Paratypes***: 2 males, 2 females with the same label as the holotype (KSP, ZSM).

##### Description.

***Body size and form***: Beetle small, with oblong-oval habitus (Fig. 61).

***Measurements***: TL 5.25–5.6 mm, TL-H 4.8–5.1 mm, MW 2.65–2.8 mm, TL/MW 1.96–2; PL 0.75–0.85 mm, PW 2.25–2.45 mm, PL/PW 0.33–0.35; DBE 0.9–0.95 mm, DBE/PW 0.38–0.4.

Holotype: TL 5.6 mm, TL-H 5.1 mm MW 2.8 mm, TL/MW 2; PL 0.85 mm, PW 2.45 mm, PL/PW 0.35; DBE 0.95 mm, DBE/PW 0.39.

***Colouration***: Dorsally piceous, with yellowish red head, pronotal sides, and on elytron with a basal band and apical spot connected with narrow lateral band (Fig. 61).

Head yellowish red, piceous narrowly behind eyes. Pronotum dark brown to piceous on disc (sometimes only small area) and paler towards sides, yellowish red on them. Elytron piceous, with a distinct yellowish red basal band, notched on posterior margin, not reaching suture and lateral margin, due to confluence of three spots on elytral base; elytron with a distinct, elongate, large apical spot connecting with a narrow lateral band. Scutellum yellow to brown. Antennae, other head appendages, and pro- and mesolegs proximally yellow, distally yellowish red; metalegs yellowish red, darker distally. Venter dark brown, with yellowish red prosternum. Teneral beetles paler.

***Surface sculpture***: Elytron with 11 distinct, complete dorsal striae; submarginal stria present: 11+1 (Fig. 61).

Head without strioles, with rather dense punctation (spaces between punctures 1–3× size of punctures); punctures relatively fine (diameter of punctures equal to diameter of microreticulation cells); head with a row of coarse setigerous punctures along inner margin of each eye and a short row at frontal angle of each eye; a slightly longer puncture row forms fronto-clypeal depression at each head side; microreticulation weak. Pronotum with several strioles at posterior margin, mainly on sides; with numerous, fine longitudinal wrinkles at posterior margin; pronotal punctation finer and denser than on head; setigerous punctures form a row along pronotal margins, absent in posterior middle; disc of pronotum with indistinct longitudinal median scratch. Pronotum with fine microreticulation. Elytron with 11 distinct, complete dorsal striae; striae 1, 3, 5, 7, and 9 reduced shortly apically; submarginal striae present, strongly developed, reaching more than ½ of elytron. Elytron with fine punctation and microreticulation. Ventral part with fine, inconspicuous punctation, invisible on metaventrite and metacoxae and weak on abdominal ventrites; prosternum smooth medially; metaventrite and metacoxae with weak microreticulation; on abdominal ventrites microreticulation almost invisible; metacoxal plates with numerous, strongly impressed longitudinal strioles, abdominal ventrites 1 and 2 with numerous, long, longitudinal strioles from margin to margin, on abdominal ventrites 3 and 4 strioles situated laterally and turn to middle, almost horizontal, abdominal ventrites 5 and 6 without strioles but with fine punctation that very sparse medially and forms a dense, rugose lateral area at each side.

***Structures***: Head relatively broad. Pronotum short and broad; lateral margins distinctly convergent anteriorly. Base of prosternum broadly rounded anteriorly, convex medially; blade of prosternal process long, narrow, broadly pointed apically, convex in middle.

***Male***: Protibia straight, not modified. Proclaws rather short, subequal in length. Median lobe of aedeagus with two lobes of dorsal sclerite relative broad; left dorsal lobe as long as right one, with its lateral crest interrupted into apical and basal parts: apical crest short and lateral long and very broad; apex of left dorsal lobe strongly curved downwards and not to left; its dorsal surface with some surface sculpture but without distinct denticulation, it invisible in left lateral view due to strong curvature downwards; right dorsal lobe with indistinct, elongate median impression; apex of right dorsal lobe elongate, weakly “swollen”, rounded; lobes of ventral sclerite almost completely sclerotised, only with very narrow membranous areas medially; sclerotised area of left ventral lobe shorter than left dorsal lobe, broad, with large, angulate prolongation to left medially and with long, thin, hook-likely curved to left apex, well visible in lateral left and ventral views; right ventral lobe with large sclerotised area, flat, not protruding, only slightly covering medially left ventral lobe. Paramere with setae not divided into distal and proximal; more distally situated setae slightly denser and longer than more proximal ones (Fig. 65).

***Female***: As male.

##### Variability.

There is an insignificant variation in the body size and colouration.

##### Affinities.

For the body size, dorsal colouration, and elytral striolation, the species could be mistaken for *A.kaszabi* but it has completely different shape of the median lobe.

##### Etymology.

The species is named after Mimika Village. The species name is a noun in the nominative singular standing apposition.

##### Distribution.

New Guinean endemic. Indonesia: Papua Province: Mimika Regency (Fig. 84).

##### Habitat.

The species was collected in stream-side puddles.

#### ﻿16. Austrelatus mirificus sp. nov.

Taxon classificationAnimaliaColeopteraDytiscidae

BEE66DE2-5A40-5D21-B129-92A0EE13893A

https://zoobank.org/A1657A68-0928-4F09-A62C-A1A49E7C1178

Figs 37 , 41 , 83

##### Type locality.

Papua New Guinea: Sandaun Province, Mianmin, 04°52'51.5"S, 141°31'42.4"Е, 700 m a.s.l.

##### Type material.

***Holotype***: male “Papua New Guinea: Sandaun, Mianmin (pool), 700 m, 21.x.2008, 04.52.858S 141.31.706E, Ibalim (PNG 198)” (ZSM).

***Paratypes***: PNG: Sandaun: 8 males, 7 females with the same label as the holotype (NHMW, ZSM). For additional paratypes see Appendix 1.

##### Description.

***Body size and form***: Beetle small, with oval habitus (Fig. 37).

***Measurements***: TL 4.85–5.5 mm, TL-H 4.3–5 mm, MW 2.6–2.9 mm, TL/MW 1.84–1.9; PL 0.7–0.8 mm, PW 2.1–2.4 mm, PL/PW 0.33; DBE 0.9–1 mm, DBE/PW 0.4–0.43.

Holotype: TL 5.15 mm, TL-H 4.7 mm, MW 2.8 mm, TL/MW 1.84; PL 0.75 mm, PW 2.4 mm, PL/PW 0.33; DBE 1 mm, DBE/PW 0.42.

***Colouration***: Dorsally piceous, with yellow head, pronotal sides, and very prominent elytral colouration of basal band/spots and narrow longitudinal bands (Fig. 37); if elytral colour pattern maximally reduced, then present as basal band and interrupted apicolateral band; if fully developed, elytra almost completely yellow, with piceous disc and narrow spaces laterally.

Head yellow to brownish yellow, dark brown behind eyes, sometimes with vague, V-like, brown spot medially that can be merged with brownish posterior margin. Pronotum dark brown to piceous, slightly paler anteriorly and laterally, with yellow to reddish yellow sides. Elytron piceous, with yellow to reddish yellow basal band of different length but not reaching suture and lateral elytral margin; its anterior margin usually reaching elytron basally and its posterior margin with three distinct prolongations; often prolongation near suture separated from band in form of elongate spot disconnected with anterior elytral margin; elytron with a distinct yellow to reddish yellow pattern of three, narrow, longitudinal bands that can be very differently developed; lateral band always present, can be interrupted, be only in apical elytral half, or reaching almost to elytral shoulder; apically it forms a loop and continues upwards as a first elytral band that can be absent, be very short, or strongly developed and reaching to second prolongation of basal band; second elytral band situated between lateral and first bands, closer to first one; can be absent, partly present, or strongly developed and reaching to basal band; first band more often reduced than second one. Scutellum yellowish to piceous. Antennae and other head appendages yellow to yellowish brown. Legs yellow to yellowish brown proximally and darker distally, especially metalegs. Venter reddish brown to brown, with paler prosternum. Teneral beetles paler.

***Surface sculpture***: Evident tendency for forming 6-stria dorsal pattern on elytron. Elytron with 2–10, sometimes uncomplete, dorsal striae, submarginal stria usually absent (2–10)+(0–1) (Fig. 37).

Head without strioles, with relatively dense, even punctation (spaces between punctures 1–3× size of punctures); punctures relatively large (diameter of punctures equal to diameter of cells of microreticulation); head with a row of coarse setigerous punctures along inner margin of each eye and a very short row of much weaker punctures at frontal angle of each eye; a slightly longer puncture row forms fronto-clypeal depression at each head side; head with strong microreticulation. Pronotum usually with distinct strioles at posterior angles, with short, thin, rather inconspicuous longitudinal wrinkles at middle of posterior margin; pronotal punctation slightly finer than on head; coarse setigerous punctures form a broad row along pronotal margins, absent in posterior middle; disc of pronotum with short, thin, longitudinal median scratch. Pronotal microreticulation rather weakly impressed on disc. Elytron with 2–10 dorsal striae, submarginal stria usually absent, present apically only in some specimens. Using 11-stria pattern: stria 1 completely reduced (only weak strioles can be find in very few specimens); striae 2, 4, 6, 8, 10, and 11 usually present complete or slightly reduced basally, stria 2 can be more distinctly reduced basally; remained striae usually differently developed, often present as weak strioles, especially in apical half or completely reduced. Only one specimen with stronger stria reduction: striae 1–3, 5, 7, 9 completely absent, striae 4, 6, 8 reduced to differently developed puncture lines, striae 10 and 11 present, slightly reduced basally. Elytron with distinct, fine punctation; microreticulation weak. Ventral part with extremely fine, scarce, inconspicuous punctation, invisible on metaventrite and metacoxae and more distinct on abdominal ventrites; prosternum smooth medially; metaventrite and metacoxae with weak microreticulation; on abdominal ventrites microreticulation almost invisible; metacoxal plates with more or less short almost longitudinal strioles, abdominal ventrites 1 and 2 with numerous, long, longitudinal strioles from margin to margin, on abdominal ventrites 3 and 4 strioles situated laterally and turn to middle, almost horizontal, abdominal ventrites 5 and 6 without strioles but with rather distinct punctation that sparser medially and forms a dense lateral area at each side.

***Structures***: Head relatively broad. Pronotum short and broad; lateral margins distinctly convergent anteriorly. Base of prosternum rounded anteriorly, slightly convex medially; blade of prosternal process small, convex in middle. Abdominal ventrite 6 broadly rounded or slightly truncate.

***Male***: Protibia straight, not modified. Proclaws simple, subequal in length: anterior claw very slightly shorter than posterior one. Median lobe of aedeagus with two lobes of dorsal sclerite rather narrow; in lateral view, apex of left dorsal lobe evenly curved downwards; left dorsal lobe dorsally relatively narrow, with distinct denticulation (spinulae) visible also in lateral left view; right dorsal lobe with small, elongate median impression and modified apex: swollen, very broadly rounded, its outer margin more strongly curved; left lobe of ventral sclerite with its sclerotised area distinct, much shorter than right ventral lobe. Paramere with setae not divided into distal and proximal; more distally situated setae denser and longer than more proximal ones (Fig. 41).

***Female***: As male.

##### Variability.

***Colouration***: The species distinctly varies in intensity of yellow colouration of dorsal surface, especially elytra. The strongly developed yellow elytral pattern is characteristic for the Sandaun populations. Their representatives always have yellow longitudinal elytral bands though in most specimens not completely developed. There is only one specimen from Bewani without elytral bands. The most reduced yellow elytral pattern is characteristic for the East Sepik populations; their representatives have no yellow longitudinal elytral bands. Specimens of the Madang population are rarely without yellow longitudinal elytral bands though the specimens with intensive yellow colouration are less then in the Sandaun. Indonesian specimens are with yellow longitudinal elytral bands, except for the specimen from Sarmi Regency (Foja Mts).

***Elytral striation***: The species evidently varies in number and degree of development of elytral striae. The maximal number of the striae is ten and is characteristic for the Sandaun populations, although specimens with the 6-stria pattern prevail in the Bewani populations. The representatives of the East Sepik and Madang populations have almost exclusively the 6- stria pattern of the elytron, as well as specimens from the Cyclops Mts. Papuan specimen from Elelim has eight elytral striae. The most interesting is the male from Sarmi (Foja Mts) with strongly reduced elytral pattern described above.

##### Affinities.

Based on size, oval body shape, partly dorsal colouration and shape of the median lobe, this species is extremely similar and most likely related to *A.nadjae* sp. nov. but differs from it by presence of the elytral striae, more intensive yellow colouration (presence of longitudinal elytral bands), absence of elytral strioles in females (see under “Female” for both species), and differences in shape of the median lobe: right dorsal lobe with distinctly smaller median impression in right lateral view; apex of right dorsal lobe less “swollen”, smaller, its outer margin more strongly curved; left dorsal lobe dorsally narrower (in dorsal view), with more distinct denticulation well visible in lateral left view due to apex curved more to left than downwards as in *A.nadjae* sp. nov.

##### Etymology.

The species name is a Latin adjective *mirificus* (wonderful) meaning beautiful dorsal colouration of the species.

##### Distribution.

New Guinean endemic. The species has rather broad distribution, found in the Papuan Province of IN and the Sandaun, East Sepik, and Madang provinces of PNG (Fig. 83).

##### Habitat.

According to the type locality labels, all specimens were collected in a pool.

#### ﻿17. Austrelatus moreguinensis sp. nov.

Taxon classificationAnimaliaColeopteraDytiscidae

643CE165-CCE2-5C63-A71D-F918CC9FAA3A

https://zoobank.org/1DB6B938-C78D-414A-8933-593CCF361DE8

Figs 77 , 81 , 84

##### Type locality.

Papua New Guinea: Central Province, Moreguina, 10°00'57"S, 148°28'27"E.

##### Type material.

***Holotype***: male “Papua New Guinea: Central, Moreguina [10°00'57"S, 148°28'27"E] 16.viii.2008 Posman (PNG183)”, “3819” [green label] (ZSM).

***Paratypes***: 1 male, 1 female with the same label as the holotype (NHMW, ZSM).

##### Description.

***Body size and form***: Beetle small, with oblong-oval habitus (Fig. 77).

***Measurements***: TL 4.8–5.5 mm, TL-H 4.3–4.95 mm, MW 2.3–2.7 mm, TL/MW 2.04–2.09; PL 0.75–0.8 mm, PW 2–2.2 mm, PL/PW 0.36–0.4; DBE 0.85–0.95 mm, DBE/PW 0.43–0.44.

Holotype: TL 4.8 mm, TL-H 4.3 mm, MW 2.3 mm, TL/MW 2.09; PL 0.75 mm, PW 2.05 mm, PL/PW 0.37; DBE 0.9 mm, DBE/PW 0.44.

***Colouration***: Dorsally piceous, with reddish head and pronotal sides, and yellowish red basal band and apical spot on elytron (Fig. 77).

Head yellowish red to brown, darker narrowly behind eyes. Pronotum brown to piceous, paler towards sides, yellowish red to brown on them. Elytron dark brown to piceous, with yellow to yellowish red basal band usually almost reaching suture (sometimes shorter, only to striae 4) but not lateral margin; with almost straight posterior margin; elytron with distinct, elongate spot apically. Scutellum brown. Antennae and other head appendages yellow. Pro- and mesolegs yellow and metalegs yellowish red proximally and darker distally. Venter mostly reddish brown to brown, with yellowish red to reddish brown prosternum and abdominal ventrites that paler laterally.

***Surface sculpture***: Elytron with 11 complete dorsal striae; submarginal stria present: 11+1 (Fig. 77).

Head with strioles between eyes or without them, with rather dense punctation (spaces between punctures 1–3× size of punctures); punctures relatively large (diameter of punctures equal to or slightly larger than diameter of cells of microreticulation); head with a row of setigerous punctures along inner margin of each eye and a short row at frontal angle of each eye; a slightly longer puncture row forms fronto-clypeal depression at each head side; head with relatively strong microreticulation. Pronotum with numerous strioles in whole surface or they absent on disc; without longitudinal wrinkles; pronotal punctation slightly sparser than on head but equally coarse; setigerous punctures form a broad row along pronotal margins, absent in posterior middle; disc of pronotum with thin, longitudinal median scratch. Pronotal microreticulation distinct. Elytron with 11 dorsal striae; striae weakly to strongly impressed; odd striae shortly reduced apically; striae 1 and 10 sometimes shortly reduced basally, striae 1–3, 9, 10 sometimes interrupted; submarginal stria present, well-developed, sometimes reaching ½ of elytron. Elytron with distinct punctation and microreticulation. Ventral part with fine, inconspicuous punctation, slightly visible on metaventrite and metacoxae and stronger on abdominal ventrites; prosternum smooth medially; metaventrite and metacoxae with distinct microreticulation; on abdominal ventrites microreticulation almost invisible; metacoxal plates with short, numerous, rather dense, distinctly impressed longitudinal strioles, abdominal ventrites 1 and 2 with numerous, long, longitudinal strioles from margin to margin, on abdominal ventrites 3 and 4 strioles situated laterally and turn to middle, almost horizontal, abdominal ventrites 5 and 6 without strioles but with fine punctation that sparser medially and forms a dense lateral area at each side.

***Structures***: Head relatively broad. Pronotum short and broad; lateral margins distinctly convergent anteriorly. Base of prosternum straight anteriorly, slightly convex medially; blade of prosternal process relatively narrow, convex in middle.

***Male***: Protibia straight, not modified. Proclaws simple, relatively short, subequal. Median lobe of aedeagus with two lobes of dorsal sclerite rather narrow; left lobe distinctly shorter than right one; in lateral left view, left dorsal lobe with long lateral crest and apex curved downwards; its dorsal surface without denticulation; right dorsal lobe with distinct, almost laterally situated median impression in right lateral view and with “swollen”, slightly elongate apex. Lobes of ventral sclerite weakly sclerotised laterally, visible in left and right lateral views, mostly membranous, subequal (right part can be protruding), straight apically; sclerotised part of left ventral lobe long and straight apically, 2/3 or more of length of left dorsal lobe. Paramere with setae divided into distal and proximal; proximal setae distinctly sparser and shorter than distal ones (Fig. 81).

***Female***: As male.

##### Variability.

There is a variation in the colouration and dorsal striolation described above.

##### Affinities.

The species is similar to *A.rajaampatensis* sp. nov. but differs from it by elytron with 11+1 complete and well-developed striae and different shape of the dorsal sclerite lobes of the median lobe.

##### Etymology.

The species is named after Moreguina Village. The name is an adjective in the nominative singular.

##### Distribution.

New Guinean endemic. Papua New Guinea: Central Province. The species is known only from the type locality area (Fig. 84).

##### Habitat.

The species was collected in stream-side puddles.

#### ﻿18. Austrelatus nadjae sp. nov.

Taxon classificationAnimaliaColeopteraDytiscidae

4A3076CF-96A4-57D6-82BD-F5DA18DDBA43

https://zoobank.org/65C6FCB1-7B05-4F53-BC1A-B7A07E53F137

**Figure 20.**
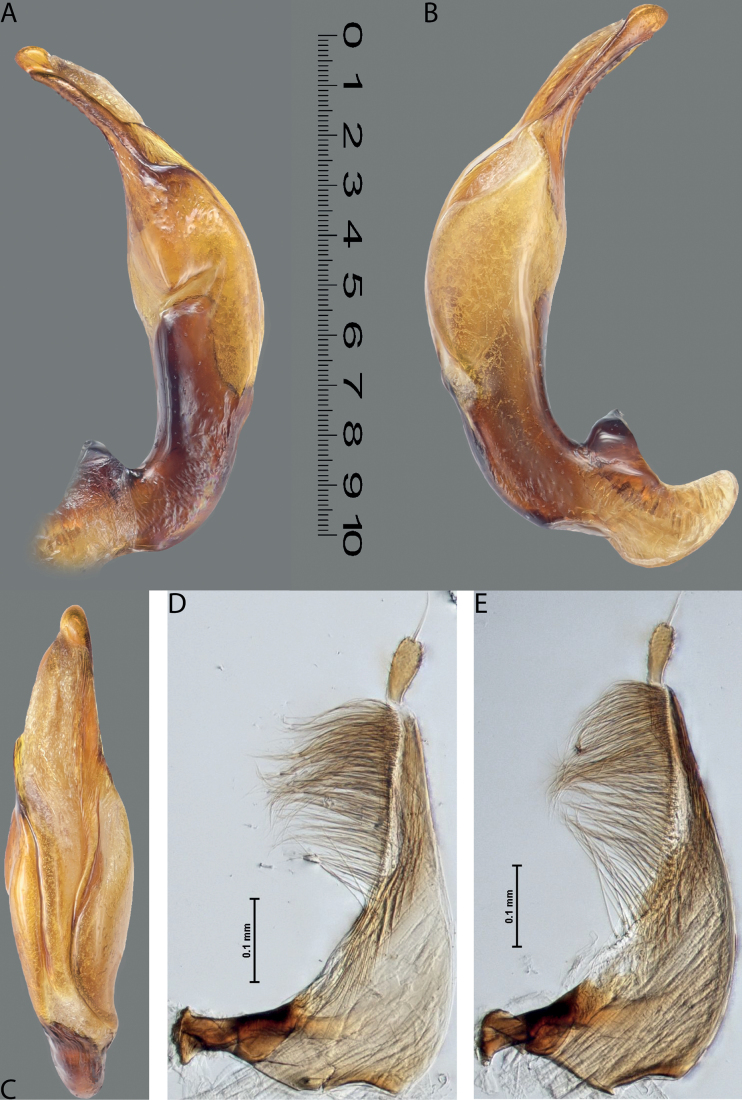
*Austrelatusnadjae* sp. nov., median lobe **A** left lateral view **B** right lateral view **C** ventral view **D** left paramere in external view **E** right paramere in internal view. Scale bar: one unit – 0.1 mm (**A–C**).

Figs 16 , 20 , 82 , 91–92 , 93

##### Type locality.

Indonesia: Papua Province: Puncak Regency, south from Iratoi, 03°21'08.3"S, 137°17'42.1"E, 161 m a.s.l.

##### Type material.

***Holotype***: male “Indonesia: Papua, S Iratoi, river camp, 161 m, 20./25.v.2015, -3,3522 137,2950, Sumoked (PAP035)” (MZB).

***Paratypes***: IN: Papua: Puncak Regency: 21 males, 11 females with the same label as the holotype (MZB, KSP, NHMW, ZSM). 1 male “Indonesia: Papua, S Iratoi, hunting camp, 150 m, 28.v.2015, -3,2801 137,3341, Sumoked (PAP042)”, “7232” [green text] (KSP, MZB, ZSM).

Yahukimo Regency: 2 males, 1 female “Indonesia: Papua, Dekai, upper Brazza, 273 m, 2/3.vi.2015, -4,7410 139,6542, Sumoked (PAP044)”, “7210”, “7211”, “7212” [green text], respectively (ZSM).

##### Description.

***Body size and form***: Beetle small, with oval habitus (Fig. 16).

***Measurements***: TL 4.8–5.1 mm, TL-H 4.4–4.7 mm, MW 2.55–2.8 mm, TL/MW 1.82–1.88; PL 0.7–0.8 mm, PW 2.1–2.4 mm, PL/PW 0.33–0.34; DBE 0.9–1 mm, DBE/PW 0.41–0.43.

Holotype: TL 4.9 mm, TL-H 4.45 mm, MW 2.6 mm, TL/MW 1.88; PL 0.75 mm, PW 2.2 mm, PL/PW 0.34; DBE 0.9 mm, DBE/PW 0.41.

***Colouration***: Dorsally piceous, with reddish yellow head, pronotal sides, elytral basal band and elytral apicolateral band (Fig. 16).

Head reddish yellow, dark brown behind eyes, sometimes vaguely brown medially and posteriorly. Pronotum dark brown to piceous, slightly paler anteriorly and laterally, with reddish yellow sides. Elytron piceous, slightly paler on disc and darker laterally, with reddish yellow basal band of different length but not reaching suture and lateral elytral margin; its anterior margin reaching elytron basally and its posterior margin strongly notched: with three distinct prolongations between puncture rows, longest near suture; elytron with distinct, reddish yellow, elongate apical spot continuing laterally as a thin band till middle of elytra or slightly further. Scutellum reddish yellow to piceous. Antennae and other head appendages yellowish brown. Pro- and mesolegs yellowish brown proximally and darker distally, especially metalegs. Venter brown to piceous, with paler prosternum. Teneral beetles paler.

***Surface sculpture***: Elytron without striae: 0+0, but with distinct puncture lines (Fig. 16).

Head without strioles, with relatively dense, even punctation (spaces between punctures 1–3× size of punctures); punctures relatively large (diameter of punctures more or less equal to diameter of cells of microreticulation) but not coarse; head with a row of coarse setigerous punctures along inner margin of each eye and a very short row of much weaker punctures at frontal angle of each eye; a slightly longer puncture row forms fronto-clypeal depression at each head side; head with strong microreticulation. Pronotum usually with distinct strioles at posterior angles that sometimes very few and inconspicuous, with short, thin, rather inconspicuous longitudinal wrinkles at middle of posterior margin; pronotal punctation slightly finer than on head; coarse setigerous punctures form a broad row along pronotal margins, absent in posterior middle; disc of pronotum with indistinct short, thin, longitudinal median scratch. Pronotal microreticulation rather weakly impressed on disc. Elytron without elytral striae but with two distinct puncture lines on disc and one laterally, some of their coarse punctures contiguous, especially those of discal lines; two additional lines of very sparse, coarse setigerous punctures can be seen between elytral lines; elytron with distinct, especially more laterally, rather dense punctation; microreticulation weak. Ventral part with extremely fine, scarce, inconspicuous punctation, invisible on metaventrite and metacoxae and more distinct on abdominal ventrites; prosternum smooth medially; metaventrite and metacoxae with weak microreticulation; on abdominal ventrites microreticulation almost invisible; metacoxal plates with more or less short longitudinal strioles, abdominal ventrites 1 and 2 with numerous, long, longitudinal strioles from margin to margin, on abdominal ventrites 3 and 4 strioles situated laterally and turn to middle, almost horizontal, abdominal ventrites 5 and 6 without strioles but with distinct punctation that sparser medially and forms a very dense lateral area at each side.

***Structures***: Head relatively broad. Pronotum short and broad; lateral margins distinctly convergent anteriorly. Base of prosternum rounded anteriorly, slightly convex medially; blade of prosternal process small, slightly convex in middle.

***Male***: Protibia straight, not modified. Proclaws short, subequal in length: anterior claw very slightly shorter than posterior one. Median lobe of aedeagus with two lobes of dorsal sclerite narrow; right dorsal lobe longer than left one; in lateral view, apex of left dorsal lobe slightly curved downwards; left dorsal lobe dorsally relatively broad, with fine but distinct denticulation (spinulae) slightly visible also in lateral left view; right dorsal lobe with large median impression (in right lateral view) and modified apex: swollen, very broadly rounded but with its outer margin more or less straight. Left lobe of ventral sclerite with its sclerotised area weakly visible laterally, distinctly shorter than right ventral lobe; its apex more or less rounded, not hooked. Paramere with setae not divided into distal and proximal; more distally situated setae denser and slightly longer than more proximal ones (Fig. 20).

***Female***: With stronger surface sculpture than males and very variable in it. They have many more strioles on pronotum, sometimes absent only on disc. Strioles can be present with different intensity on elytra, usually more numerous laterally. In rare cases (three specimens), there are completely striolated, matt forms. One female demonstrates development of the elytral puncture lines (especially discal) into weakly impressed striae.

##### Affinities.

In absence of the elytral striae and general shape of the median lobe, it is similar to *A.lembenensis* sp. nov., but differs from it distinctly more oval habitus, more prominent yellow pattern and different shape of the median lobe. The species is extremely similar and most likely related to broadly distributed *A.mirificus* sp. nov. We treat it here as a separate species because of absence of the elytral striae, less intensive yellow colouration, differences in female dorsal striolation (see under “Female” for both species) and in shape of the median lobe, for details see under *A.mirificus* sp. nov.

##### Etymology.

The species is named after Nadja, the elder daughter of H. Shaverdo. “You are beautiful and great as you are!” The species name is a noun in the genitive case.

##### Distribution.

New Guinean endemic. Indonesia: Papua Province: Puncak and Yahukimo regencies (Fig. 82).

##### Habitat.

At the locality PAP044, it was collected in puddles or pools among rotten leaves and twigs (Figs 91, 92); at the locality PAP042 in a forest pool (Fig. 93).

#### ﻿19. Austrelatus neoguineensis

Taxon classificationAnimaliaColeopteraDytiscidae

(Zimmermann, 1919) comb. nov.

D06CAF60-5BA2-54E8-A2D8-F7FF005EEA58

Figs 11 , 36 , 40 , 82

Copelatus neoguineensis Zimmermann, 1919: 199–200; Zimmermann (1920b: 141); Guignot (1956: 55); Guéorguiev (1968: 25); Guéorguiev and Rocchi (1993: 160); Nilsson and Hájek (2023: 49).

##### Type locality.

“Neuguinea, Herbertshöhe” [Papua New Guinea: East New Britain Province, Kokopo, ca. 04°21′S, 152°16′E].

##### Type material.

***Lectotype*** (by present designation): male [lacks left proleg and one paramere] “Herbertshöhe D.N. Guinea.”, “Coll. Bennigsen”, “Zimmermann det.”, “Lectotype *Copelatusneoguineensis* Zimmermann, 1919 des. H. Shaverdo 2023” [red] (SDEI).

***Paralectotypes***: 1 female [beetle and one paramere (sic!) glued on pined triangular] “Neu Guinea” [hw by ?Zimmermann], “Type” [round, hw by ?Zimmermann], “Samml. A. Zimmermann”, “Type” [red], “Copelatusneoguineensis” (ZSM). 1 male [without genitalia] “Herbertshöhe D.N. Guinea.”, “Coll. Bennigsen”, “TYPUS” [red], “Syntypus” [red], “Copelatus neo-guineensis Zimerm. [sic!] Type” [hw by ?Zimmermann] (SDEI). 1 male [only median lobe, one paramere and right protarsus glued on pined card, beetle probably lost or the parts belong to the previous specimen] “Penis C. neoguin” [hw by ?Zimmermann], “Samml. A. Zimmermann” (SDEI).

##### Notes.

The lectotype is designated for purpose of the stability of nomenclature, as the existing works do not allow undoubted identification of the species. Although five specimens are mentioned in the original description (Zimmermann 1919: 200), we have found only three of them.

##### Additional material.

See Appendix 1.

##### Description.

***Body size and form***: Beetle medium-sized, with oblong-oval habitus (Fig. 36).

***Measurements***: TL 5.2–6.9 mm, TL-H 4.7–6.35 mm, MW 2.6–3.35 mm, TL/MW 2–2.06; PL 0.7–0.95 mm, PW 2.2–2.85 mm, PL/PW 0.32–0.33; DBE 0.9–1.25 mm, DBE/PW 0.39–0.44.

Lectotype: TL 6.45 mm, TL-H 5.9 mm, MW 3.2 mm, TL/MW 2.02; PL 0.9 mm, PW 2.85 mm, PL/PW 0.32; DBE 1.1 mm, DBE/PW 0.39.

***Colouration***: Dorsally piceous, with reddish yellow head, pronotal sides, and two basal and one apical spot on elytron (Fig. 36).

Head yellowish red to reddish brown, darker narrowly behind eyes. Pronotum reddish brown to dark brown on disc and paler towards sides, yellowish red to reddish brown on them. Sometimes only anterior and posterior margins medially dark. Elytron dark brown to piceous, with two yellow to reddish brown spots on elytral base: one between striae 2 and 4 and second between striae 4 and 6; sometimes these spots distinct, sometimes vague; second usually better developed but not reaching lateral elytral margin; sometimes spots confluent like a denticule band, seldom absent; elytron with more or less distinct elongate apical spot. Scutellum reddish brown to piceous. Antennae, other head appendages, and legs proximally yellow to yellowish red; legs darker distally, especially metalegs. Venter yellowish red to reddish brown, with paler prosternum. Teneral beetles paler.

***Surface sculpture***: Elytron with six more or less complete dorsal striae; stria 1 usually reduced basally and present only in apical elytral half; submarginal stria usually absent: 6+(0–1) (Fig. 36).

Head without strioles, with relatively sparse punctation (spaces between punctures 1–3× size of punctures); punctures relatively fine (diameter of punctures usually smaller than diameter of microreticulation cells); head with a row of coarse setigerous punctures along inner margin of each eye and a short row at frontal angle of each eye; a slightly longer puncture row forms fronto-clypeal depression at each head side; microreticulation distinct but not strong. Pronotum usually with several strioles laterally or only in posterolateral angles, seldom without them; with fine longitudinal wrinkles at posterior margin; pronotal punctation finer than on head; setigerous punctures form a row along pronotal margins, absent in posterior middle; disc of pronotum with indistinct longitudinal median scratch. Pronotal microreticulation rather weakly impressed. Pronotum sometimes with numerous strioles in females. Elytron with 6 dorsal striae; stria 1 usually reduced basally and present only in apical elytral half; striae 2, 4, 6 complete; striae 3 and 5 usually reduced or interrupted basally, seldom complete; submarginal striae usually absent, seldom present. Elytron with very fine, inconspicuous punctation and very weak microreticulation. Elytron sometimes with numerous strioles in females. Ventral part with fine, inconspicuous punctation, invisible on metaventrite and metacoxae and weak on abdominal ventrites; prosternum smooth medially; metaventrite and metacoxae with weak microreticulation; on abdominal ventrites microreticulation almost invisible; metacoxal plates with numerous, finely impressed longitudinal strioles, abdominal ventrites 1 and 2 with numerous, long, longitudinal strioles from margin to margin, on abdominal ventrites 3 and 4 strioles situated laterally and turn to middle, almost horizontal, abdominal ventrites 5 and 6 without strioles but with rather distinct punctation that sparser medially and forms a dense, rugose lateral area at each side.

***Structures***: Head relatively broad. Pronotum short and broad; lateral margins distinctly convergent anteriorly. Base of prosternum narrowly rounded anteriorly, convex medially; blade of prosternal process relatively narrow, convex in middle.

***Male***: Protibia not straight, thinner proximally and broader medially and distally due to its slightly curved ventral margin. Proclaws relatively long, subequal. Median lobe of aedeagus with two lobes of dorsal sclerite broad, almost straight or slightly curved downwards apically; right dorsal lobe slightly longer and broader than left one, with a broadly pointed apex and large median membranous impression in right lateral view; in left lateral view, left dorsal lobe with a lateral longitudinal crest. Lobes of ventral sclerite weakly sclerotised laterally (visible in left and right lateral views), mostly membranous, subequal, strongly pressed together, usually slightly curved upwards apically; sclerotised part of left ventral lobe small, short, sometimes indistinct (in left lateral view). Paramere with setae not divided into distal and proximal, more or less uniform (Figs 11, 40).

***Female***: Dimorphic. Some females have numerous thin, longitudinal strioles on elytra and pronotum that densely cover dorsal surface and make it matt. Ratio shiny: striolated is different in populations. Usually shiny forms are more numerous. E.g., 24: 2 in PNG54; 1: 1 38+37+?36/2013; only with strioles in 44/2013; 5: 3 in 33/2013; 3: 7 in PNG188; 16: 2 in PNG 115; 18: 7 in PNG27; 40: 2 in leg. Messer 26Mar.; 25: 5 in leg. Messer 14Feb.; 34: 8 in leg. Messer 7Feb.; 13: 7 in leg. Messer 14Jan.; 20: 26 in PNG196; 8: 27 in PNG198.

##### Variability.

The species demonstrates some variation in its dorsal colouration and variation in elytral striolation described above.

##### Affinities.

Based on general appearance and shape of the median lobe, the species is close to *A.bormensis* sp. nov., but also similar to *A.pseudoneoguineensis* sp. nov. See the differences under these species.

##### Distribution.

New Guinean endemic. Literature records: New Guinea (Guignot 1956; Guéorguiev 1968) and PNG: the species is described from New Britain Island (Zimmermann 1919, 1920b; Guéorguiev and Rocchi 1993); however, our study shows that the species is widespread in New Guinea, and especially numerous in Sandaun, East Sepik, and Madang provinces (Fig. 82).

##### Habitat.

In the Cyclops Mountains, it was collected in different kind of small and shallow pools and puddles alongside smaller forest streams. Around Mount Wilhelm, numerous specimens have been collected with flight intercept traps during the IBISCA project.

#### ﻿20. Austrelatus oksibilensis sp. nov.

Taxon classificationAnimaliaColeopteraDytiscidae

C1D2843A-2E81-5879-8E07-A2065EF29BC5

https://zoobank.org/76FF855A-CF1E-4C74-824C-54BA8C7446C3

Figs 23 , 27 , 82

##### Type locality.

Indonesia: Papua Province: Pegunungan Bintang Regency, south from Ok Sibil, tributary Digul River, 05°03'25.9"S, 140°43'21.1"E, 359 m a.s.l.

##### Type material.

***Holotype***: male “Indonesia: Papua, S Ok Sibil, tributary of Digul Riv, 359 m, 9.vi.2018, -5,0571 140,7225, Sumoked (Pap051)” (MZB).

***Paratypes***: IN: Papua: Pegunungan Bintang Regency: 9 males, 6 females with the same label as the holotype (MZB, NHMW, ZSM). 7 males, 4 females “Indonesia: Papua, S Ok Sibil, tributary of Digul Riv, 359 m, 9.vi.2015, -5,0571 140,7225, Sumoked (Pap051)” (MZB, KSP, ZSM).

PNG: Western: 2 males “Tabubil, 05°18.466'S 141°19.531'E, 810 m, beaten”, “25-iii-2019 – pos. 4 Dytiscidae” (SMNK). 2 males, 1 female “Papua New Guinea: Western, Tabubil, 810 m, 25.iii.2019”, "S05° 18.466' "E 141°19.531' Riedel”, all with additional green text labels “8679”, “8680”, and “8681” (ZSM).

##### Additional material.

PNG: Sandaun: 1 female “NEW GUINEA W-Sepik Pr.: Yapsiei 4°38'S-141°05'E 250 m, 17.I.1989 Leg. M. & R. Holyński”, “water in rotten fallen trunk” (ZSM).

##### Description.

***Body size and form***: Beetle medium-sized, with oblong-oval habitus (Fig. 23).

***Measurements***: TL 5–6.1 mm, TL-H 4.6–5.6 mm, MW 2.5–3.1 mm, TL/MW 1.97–2.03; PL 0.75–0.95 mm, PW 2.2–2.7 mm, PL/PW 0.34–0.35; DBE 0.9–1.05 mm, DBE/PW 0.38–0.41.

Holotype: TL 6.1 mm, TL-H 5.6 mm, MW 3 mm, TL/MW 2.03; PL 0.9 mm, PW 2.6 mm, PL/PW 0.35; DBE 1 mm, DBE/PW 0.38.

***Colouration***: Dorsally piceous, with reddish yellow to brown head, pronotal sides, and notched basal band or spots on elytron as well as elytral apical spot and narrow lateral band (Fig. 23).

Head reddish yellow to brown, darker behind eyes. Pronotum yellowish brown to piceous on disc and reddish yellow to brown on sides. Elytron piceous, with reddish yellow to brown basal band of different length but not reaching suture and lateral elytral margin; its anterior margin reaching elytron basally and its posterior margin strongly notched: usually with two prolongation between puncture rows, sometimes with vague basal spot near suture, sometimes band splitted into basal spots; elytron with distinct, reddish yellow to brown, elongate apical spot usually continuing laterally as a thin band maximum till middle of elytra. Scutellum usually brown to piceous. Antennae and other head appendages yellowish brown. Pro- and mesolegs yellowish brown proximally and darker distally, especially metalegs. Venter yellowish brown to dark brown, with paler prosternum. Teneral beetles paler.

***Surface sculpture***: Elytron without striae: 0+0, but with distinct puncture lines (Fig. 23).

Head without strioles, with relatively dense, even punctation (spaces between punctures 1–3× size of punctures); punctures relatively coarse (diameter of punctures equal to diameter of cells of microreticulation); head with a row of coarse setigerous punctures along inner margin of each eye and a short row at frontal angle of each eye; a slightly longer puncture row forms fronto-clypeal depression at each head side; sometimes head with large shallow median depression; microreticulation strongly impressed. Pronotum with strioles, sometimes few and indistinct, at posterior angles and thin, longitudinal wrinkles at middle of posterior margin; pronotal punctation finer than on head; coarse setigerous punctures form a broad row along pronotal margins, absent in posterior middle; disc of pronotum with thin, longitudinal median scratch. Pronotal microreticulation rather weakly impressed on disc. Elytron without elytral striae but with two distinct puncture lines on disc and one less distinct laterally; two additional lines of very sparse, coarse setigerous punctures can be seen between elytral lines; elytron with very fine, inconspicuous punctation; microreticulation weak. Ventral part with fine, inconspicuous punctation, invisible on metaventrite and metacoxae and weak on abdominal ventrites; prosternum smooth medially; metaventrite and metacoxae with distinct microreticulation; on abdominal ventrites microreticulation almost invisible; metacoxal plates with more or less short longitudinal strioles, abdominal ventrites 1 and 2 with numerous, long, longitudinal strioles from margin to margin, on abdominal ventrites 3 and 4 strioles situated laterally and turn to middle, almost horizontal, abdominal ventrites 5 and 6 without strioles but with rather distinct punctation that sparser medially and forms a dense lateral area at each side.

***Structures***: Head relatively broad. Pronotum short and broad; lateral margins distinctly convergent anteriorly. Base of prosternum rounded anteriorly, convex medially; neck of prosternal process distinctly convex; blade of prosternal process elongate, narrow, distinctly convex in middle.

***Male***: Protibia straight, not modified. Proclaws long; anterior claw shorter, slightly thicker and more strongly curved downwards than posterior due to slight, median incision of its inner margin. Median lobe of aedeagus with two lobes of dorsal sclerite rather narrow; right dorsal lobe slightly longer than left one; in lateral view, apex of left dorsal lobe more or less straight, with longitudinal crest and weak but distinct incision on its lateral margin; right dorsal lobe with weakly developed, inconspicuous median impression (in right lateral view) and rounded apex. Left lobe of ventral sclerite with its sclerotised area large, very strongly sclerotised, distinctly shorter than right ventral lobe; its apex bilobed: left part short, broad, and rounded, right one long, thin, hooked; this sclerotised area hidden under right ventral lobe and between left and right lobes of dorsal sclerite, usually invisible (only hook’s apex can be visible) in left lateral view. Paramere with setae divided into distal and proximal; proximal setae distinctly sparser and slightly shorter than distal (Fig. 27).

***Female***: Usually with more numerous pronotal strioles, often occupying entire lateral sides.

##### Affinities.

In absence of the elytral striae and general shape of the median lobe, it is most similar and most likely closely related to *A.pseudooksibilensis* sp. nov. and especially to *A.brazza* sp. nov. but differs from them by its slightly larger body size. From *A.pseudooksibilensis* sp. nov., it differs also by less extended yellow colouration of the elytra (presence of lateral band, absence of dorsal one), and weaker incision on the apex of the left lobe of the dorsal sclerite of the median lobe, as well as males with pronotal strioles and females without elytral strioles. The species has median lobe very similar to that of *A.brazza* sp. nov. but they differ in shape of their left and right lobes of the dorsal sclerite: In *A.oksibilensis* sp. nov., apical part of left dorsal lobe (before incision) more elongate and part after incision flatter; apical part of right dorsal lobe more elongate. In *A.brazza* sp. nov., apical part of left dorsal lobe (before incision) smaller, shorter, and part after incision more prominent; apical part of right dorsal lobe shorter, more rounded. Additionally, *A.oksibilensis* sp. nov. has darker dorsal colouration of the body and elytron without striae: 0+0. *Austrelatusbrazza* sp. nov. is paler dorsally and its elytron without or with 6–10 dorsal striae, submarginal stria absent: (0–10)+0.

##### Etymology.

The species is named after its type locality area, Ok Sibil. The name is an adjective in the nominative singular.

##### Distribution.

New Guinea endemic. Indonesia: Papua Province: Pegunungan Bintang Regency and PNG: Western. If the record from Sandaun is confirmed, the species will be considered more widely spread in the region (Fig. 82).

##### Habitat.

The species was collected in stream-side puddles and a water-filled fallen trunk.

#### ﻿21. Austrelatus pseudoneoguineensis sp. nov.

Taxon classificationAnimaliaColeopteraDytiscidae

B313110E-1350-5C3D-8F59-1DCA9CC2132A

https://zoobank.org/586CCCB1-C491-43F9-B736-26F2C9F32D05

Figs 17 , 21 , 82

##### Type locality.

Indonesia: Papua Province: Nabire Regency, road Nabire-Enarotali, 54^th^ km, ca. 03°29.52'S, 135°43.91'E, 750 m a.s.l.

##### Type material.

***Holotype***: male “Irian Jaya: Nabire distr., road Nabire-Ilaga, km 54, 03°29'517"S 135°43'913"E, 750 m, iv.1998” (MZB).

***Paratypes***: IN: Papua: Nabire Regency: 11 males, 5 females with the same label as the holotype (NHMW, ZSM). 1 male “Irian Jaya: Nabire Prov., rd. Nabire – Ilaga, Km 54, ca. 750 m, X.1997” (NHMW). 1 female “Irian Jaya: Nabire Prov., rd. Nabire – Ilaga, km 54, 03°29'51"S 135°43'91"E 750 m, IV.1998” (NHMW). 1 male, 1 female “Irian Jaya: Nabire Prov., Nabire – Ilaga, km 54, 26.9.1997, 750 m (# 4)” (NHMW). 1 male “Indonesia: Papua, Nabire – Ilaga km 54, 750 m, 25 & 27.vii.1991, Balke, IR#91-7 (IR 24)” (ZSM). 2 male, 4 females “Indonesia: Papua, Road Nabire-Enarotali KM 55, 774 m, 22.x.2011, 03.29.796S 135.43.885E, team UNCEN (PAP09)” one female with an additional green text label “5130” (KSP, MZB). 1 male, 1 female “Indonesia: Papua, Road Nabire-Enarotali KM 35, r. Topo, 130 m, 22.x.2011, 03.28.727S 135.38.734E, team UNCEN (PAP09A)” (ZSM). 1 male “Indonesia: Papua, Road Nabire-Enarotali KM 40, 350 m, 23.x.2011, 03.29.314S 135.41.188E, team UNCEN (PAP18)” (ZSM). 1 male, 1 female “Irian Jaya: Nabire Prov., Nabire – Ilaga, km 35, Kali Cemara, 26.9.1997 (# 5)” (NHMW).

Jayawijaya Regency: 1 male “Indonesia: Papua, Wamena, 20 mins towd Jiwika, limestone creek, 1620 m, 03 56.953'S 138 54.375'E, team UNCEN (PAP05)” (ZSM).

##### Description.

***Body size and form***: Beetle medium-sized, with oblong-oval habitus (Fig. 17).

***Measurements***: TL 5.2–5.85 mm, TL-H 4.7–5.3 mm, MW 2.5–2.85 mm, TL/MW 2.05–2.08; PL 0.7–0.8 mm, PW 2.2–2.5 mm, PL/PW 0.32; DBE 0.9–1 mm, DBE/PW 0.4–0.41.

Holotype: TL 5.85 mm, TL-H 5.3 mm, MW 2.85 mm, TL/MW 2.08; PL 0.8 mm, PW 2.5 mm, PL/PW 0.32; DBE 1 mm, DBE/PW 0.4.

***Colouration***: Dorsally piceous, with yellowish brown to brown head and pronotum or pronotal sides, rarely with small, yellowish brown to reddish brown elytral latero-basal spot (Fig. 17).

Head yellowish brown to brown, darker behind eyes. Pronotum yellowish brown to almost piceous, usually with paler sides and darker disc. Elytron uniformly piceous, rarely with small, yellowish brown to reddish brown basal spot situated between second dorsal puncture line and lateral puncture line. Scutellum reddish yellow to piceous. Antennae and other head appendages yellowish brown. Pro- and mesolegs yellowish brown proximally and darker distally, especially metalegs. Venter yellowish brown to dark brown, with paler prosternum. Teneral beetles paler.

***Surface sculpture***: Elytron without striae: 0+0, but with distinct puncture lines (Fig. 17).

Head without strioles, with relatively dense, even punctation (spaces between punctures 1–3× size of punctures); punctures relatively small (diameter of punctures slightly smaller than diameter of cells of microreticulation); head with a row of coarse setigerous punctures along inner margin of each eye and a short row at frontal angle of each eye; a slightly longer puncture row forms fronto-clypeal depression at each head side; head with strong microreticulation. Pronotum with distinct strioles at posterior angles and thin, longitudinal wrinkles at middle of posterior margin; pronotal punctation finer than on head; coarse setigerous punctures form a broad row along pronotal margins, absent in posterior middle; disc of pronotum with thin, longitudinal median scratch. Pronotal microreticulation rather weakly impressed on disc. Elytron without elytral striae but with two distinct puncture lines on disc and one less distinct laterally; two additional lines of very sparse, coarse setigerous punctures can be seen between elytral lines; elytron with fine, dense, rather inconspicuous punctation; microreticulation weak. Ventral part with fine, inconspicuous punctation, almost invisible on metaventrite and metacoxae and rather distinct on abdominal ventrites; prosternum smooth medially; metaventrite and metacoxae with weak microreticulation; on abdominal ventrites microreticulation almost invisible; metacoxal plates with more or less short longitudinal strioles, abdominal ventrites 1 and 2 with numerous, long, longitudinal strioles from margin to margin, on abdominal ventrites 3 and 4 strioles situated laterally and turn to middle, almost horizontal, abdominal ventrites 5 and 6 without strioles but with rather distinct punctation that sparser medially and forms a dense lateral area at each side.

***Structures***: Head relatively broad. Pronotum short and broad; lateral margins distinctly convergent anteriorly. Base of prosternum rounded anteriorly, slightly convex medially; neck of prosternal process distinctly convex; blade of prosternal process elongate, narrow, distinctly convex in middle.

**Figure 21.**
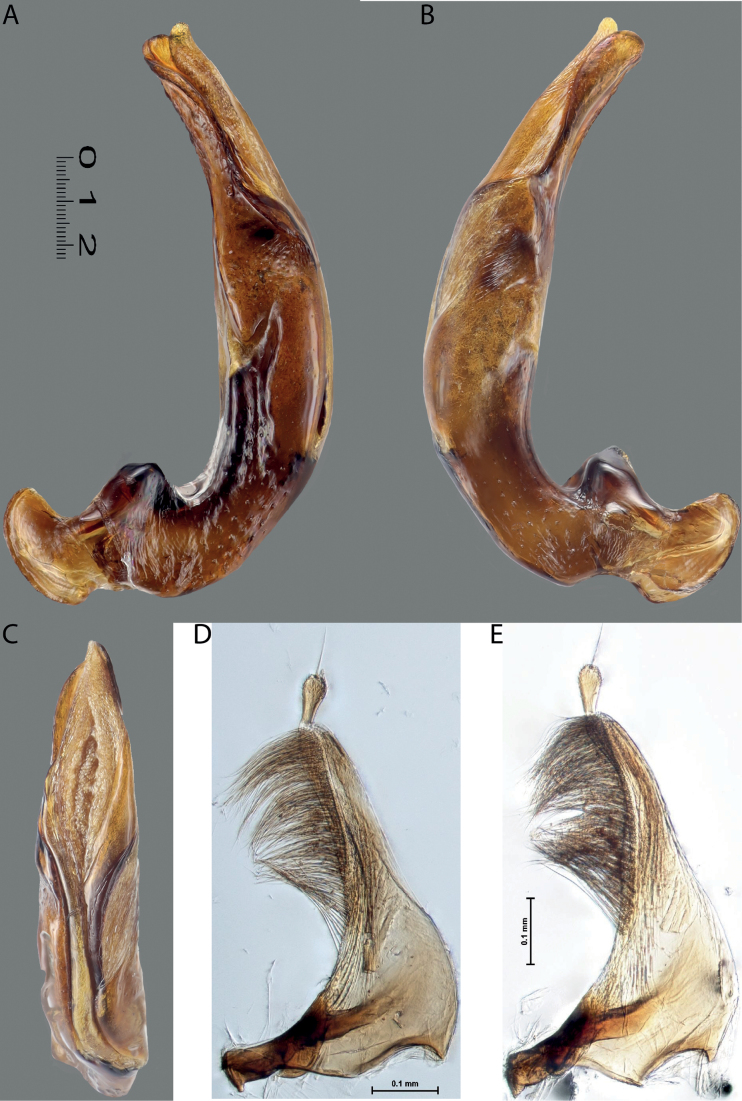
*Austrelatuspseudoneoguineensis* sp. nov., median lobe **A** left lateral view **B** right lateral view **C** ventral view **D** left paramere in external view **E** right paramere in internal view. Scale bar: one unit – 0.1 mm (**A–C**).

***Male***: Protibia straight, not modified. Proclaws relatively long, equal in length. Median lobe of aedeagus with two lobes of dorsal sclerite rather narrow; right dorsal lobe slightly longer than left one; in lateral view, apex of left dorsal lobe more or less straight, with weak longitudinal crest on its lateral margin; right dorsal lobe with large median impression (in right lateral view) and flat, relatively broad apex. Left lobe of ventral sclerite with its sclerotised area weak, indistinct laterally, shorter than right ventral lobe; lobes of ventral sclerite pressed together; right ventral lobe its sclerotised area well-developed, tip of apex of right ventral lobe often slightly curved upwards. Paramere with setae not divided into distal and proximal; sometimes more distally situated setae denser and slightly longer than more proximal ones in left paramere (Fig. 21).

***Female***: As male.

##### Affinities.

Based on shape of the median lobe, the species is close to *A.neoguineensis* but differs from it in smaller and narrower habitus, absence of elytral striae and slightly different shape of median lobe sclerites. In absence of the elytral striae and general shape of the median lobe, it is similar to *A.oksibilensis* sp. nov., *A.pseudooksibilensis* sp. nov. and *A.febrisauri* sp. nov. but differs from them by more uniformly coloured elytra, almost without yellowish and different shape of the median lobe.

##### Etymology.

The species is named for morphological similarity (especially of its median lobe) to *A.neoguineensis*. The name is a noun in the nominative standing in apposition.

##### Distribution.

New Guinean endemic. Indonesia: Papua Province: Nabire and Jayawijaya regencies (Fig. 82).

##### Habitat.

All specimens were collected in shallow (up to 20 cm water depth), shaded or at least partly shaded forest pools and puddles of different size, rich in rotten leaves and twigs; few specimens also found in water-filled track hollows on forest tracks.

#### ﻿22. Austrelatus pseudooksibilensis sp. nov.

Taxon classificationAnimaliaColeopteraDytiscidae

83BE36CE-B473-5C62-B42C-0CF436588FBE

https://zoobank.org/E566E8BE-ED93-458F-97A4-B40968AE7333

Figs 24 , 25 , 28 , 82 , 87

##### Type locality.

Indonesia: Papua Province: Nabire Regency, road Nabire-Enarotali, 62^nd^ km, 250 m a.s.l.

##### Type material.

***Holotype***: male “West New Guinea/Paniai Prov./IR 22 track Nabire-Ilaga km 62 250 m, 24.7.1991, forest pools leg: Balke & Hendrich” (ZSM).

***Paratypes***: IN: Papua Province: Nabire Regency: 23 males, 4 females, with the same label as the holotype (MZB, NHMW, ZSM). 2 males, 3 females “Ir 23-W. New Guinea, track Nabire-Ilaga KM 62, 250 m, 24.vii.1991 Balke & Hendrich leg.” (ZSM). 2 males, 4 females “West New Guinea/Paniai Prov./IR 20 track Nabire-Ilaga KM 59, ca.750 m, 18.7.1991 leg: Balke & Hendrich leg.” (ZSM). 2 males “West New Guinea/Paniai Prov./IR 18 River n. Nabire, 2 m, 15.7.1991 leg: Balke & Hendrich leg.” (ZSM). 10 males, 16 females “Irian Jaya: Paniai Prov. road Nabire – Ilaga, km 65, 29.8.1996, 250 m leg. M. Balke (96 # 6)” (MZB, NHMW, ZSM).

West Papua Province: Teluk Wondama Regency: 2 males, 1 female “Indonesia: West Papua, DMP, Wasior, 7.-10.i.2001, Riedel leg.” (NHMW, ZSM). 1 male “Indonesia: Irian Jaya, Wandammen, Wasior, DMP, 7.-10.I.2001, Riedel, MB 51”, “51” [green label] (ZSM). 1 male “Indonesia: Irian Jaya, Wandammen, Wasior, DMP, 7.-10.I.2001, Riedel, “MB 52”, “52” [green label] (ZSM).

##### Description.

***Body size and form***: Beetle small, with oblong-oval habitus (Figs 24, 25).

***Measurements***: TL 4.9–5.6 mm, TL-H 4.5–5.2 mm, MW 2.4–2.8 mm, TL/MW 2–2.04; PL 0.7–0.8 mm, PW 2.1–2.4 mm, PL/PW 0.33–0.35; DBE 0.9–1 mm, DBE/PW 0.41–0.43.

Holotype: TL 5.5 mm, TL-H 5 mm, MW 2.75 mm, TL/MW 2; PL 0.8 mm, PW 2.3 mm, PL/PW 0.35; DBE 0.95 mm, DBE/PW 0.41.

***Colouration***: Dorsally brown to piceous, with reddish yellow to reddish brown head, pronotum or pronotal sides, and notched basal band, one narrow dorsal band and apical spot on elytron (Figs 24, 25).

Head reddish yellow to reddish brown, darker narrowly behind eyes. Pronotum reddish yellow to reddish brown, with darker disc and paler sides; sometimes darkest parts just along middle of anterior and posterior margins. Elytron brown to piceous, with reddish yellow to reddish brown basal band of different length but not reaching suture and lateral elytral margin; its anterior margin reaching elytron basally and its posterior margin strongly notched: with two prolongation between puncture rows, sometimes with vague basal spot near suture; elytron with distinct, elongate apical spot and usually with narrow dorsal band slightly in front of second puncture row starting near basal band and of different length (maximum till slightly behind elytral length); elytron without lateral band. Scutellum usually reddish brown to piceous. Antennae and other head appendages yellow. Legs reddish yellow proximally and darker distally, especially metalegs. Venter mostly reddish yellow, with paler prosternum. Teneral beetles paler.

***Surface sculpture***: Elytron without striae, but with distinct puncture lines, or with 3–9 more or less complete dorsal striae, submarginal stria absent: (0–9)+0 (Figs 24, 25).

Head without strioles, with relatively sparse, inconspicuous punctation (spaces between punctures 1–4× size of punctures); punctures relatively small, weakly impressed (diameter of punctures slightly smaller than diameter of cells of microreticulation); head with a row of coarse setigerous punctures along inner margin of each eye and a short, weak row at frontal angle of each eye; a slightly longer puncture row forms fronto-clypeal depression at each head side; microreticulation strongly impressed. Pronotum with strioles only in females; with thin, longitudinal wrinkles at posterior margin; pronotal punctation finer than on head; coarse setigerous punctures form a broad row along pronotal margins, absent in posterior middle; disc of pronotum with thin, longitudinal median scratch. Pronotal microreticulation rather weakly impressed on disc. Pronotum often with fine strioles in females. Elytron without elytral striae but with two distinct puncture lines on disc and one less distinct laterally; two additional lines of very sparse, coarse setigerous punctures can be seen between elytral lines; or with 3–9 more or less complete dorsal striae, usually missing near suture and getting denser towards lateral margin. Elytron with very fine, inconspicuous punctation and weak microreticulation. Elytron often with fine strioles in females. Ventral part with fine, inconspicuous punctation, invisible on metaventrite and metacoxae and weak on abdominal ventrites; prosternum smooth medially; metaventrite and metacoxae with weak microreticulation; on abdominal ventrites microreticulation almost invisible; metacoxal plates with short, not numerous longitudinal strioles, abdominal ventrites 1 and 2 with numerous, long, longitudinal strioles from margin to margin, on abdominal ventrites 3 and 4 strioles situated laterally and turn to middle, almost horizontal, abdominal ventrites 5 and 6 without strioles but with rather distinct punctation that sparser medially and forms a dense lateral area at each side.

***Structures***: Head relatively broad. Pronotum short and broad; lateral margins distinctly convergent anteriorly. Base of prosternum narrowly rounded anteriorly, convex medially; blade of prosternal process relatively broad, convex in middle.

***Male***: Protibia straight, not modified. Proclaws long; anterior claw shorter, thicker anteriorly (almost denticulate) and more strongly curved downwards than posterior due to median incision of its inner margin. Median lobe of aedeagus with two lobes of dorsal sclerite rather narrow; right dorsal lobe slightly longer than left one; in lateral view, apex of left dorsal lobe more or less straight, with longitudinal crest, deep incision and below it, concavity in lateral margin; right dorsal lobe with weakly developed, inconspicuous median impression (in right lateral view) and rounded apex. Left lobe of ventral sclerite with its sclerotised area large, very strongly sclerotised, distinctly shorter than right ventral lobe; its apex bilobed: left part short, broad, and rounded, right one long, thin, hooked; this sclerotised area hidden under right ventral lobe and between left and right lobes of dorsal sclerite, usually almost invisible (only hook’s apex can be visible) in left lateral view. Paramere with setae distinctly divided into distal and proximal; proximal setae distinctly sparser and slightly shorter than distal (Fig. 28).

***Female***: With pronotal strioles usually occupying entire lateral sides, and often presence of numerous but weak elytral strioles. Most of the females have strioles on elytra and pronotum but they are not very dense and not strongly impressed; therefore, dorsal surface is not matt, rather shiny.

##### Variability.

The species demonstrates insignificant variation in its dorsal colouration. It is so far known from two regions: Nabire and Wasior (Wandammen) and shows a distinct separation for them in the elytral striolation: specimens from Nabire without elytral striae and those from Wasior with them. Additionally, Wasior males have slightly differently shaped left lobe of the dorsal sclerite of the median lobe. However, this structure seems to be slightly variable within and among the populations. Therefore, more material is needed from Wandammen to continue study on the taxonomic status of its populations.

##### Affinities.

In absence of the elytral striae and general shape of the median lobe, it is most similar and most likely closely related to *A.oksibilensis* sp. nov. and *A.brazza* sp. nov.; see the comparison under these species. The new species co-occurs with *A.febrisauri* sp. nov., to which is very similar in size, colouration and surface structures but differs by more notched basal elytral band and presence of dorsal elytral band. Males can be also distinguished by lateral incision on the left lobe of the dorsal sclerite of the median lobe, as well as modified anterior claw; females by the presence of elytral strioles.

##### Etymology.

The species is named for its morphological similarity and close relationship to *A.oksibilensis* sp. nov. The name is a noun in the nominative standing in apposition.

##### Distribution.

New Guinean endemic. Indonesia: Papua Province: Nabire Regency and West Papua Province: Teluk Wondama Regency (Fig. 82).

##### Habitat.

At the type locality, the species was collected in a small forest pool (Fig. 87). At the track Nabire-Ilaga, all specimens were collected in shallow (up to 20 cm water depth), shaded or at least partly shaded forest pools and puddles of different size, rich in rotten leaves and twigs; few specimens also found in water-filled track hollows on forest tracks.

#### ﻿23. Austrelatus rajaampatensis sp. nov.

Taxon classificationAnimaliaColeopteraDytiscidae

E7EB8609-C026-5879-8EA1-BD118299C969

https://zoobank.org/1FE319B7-6EE2-4939-8ED6-E584D4D39A41

Figs 54 , 55 , 57 , 84

##### Type locality.

Indonesia: Papua Province: Raja Ampat Regency, northern Batanta, 00°50.125'S, 130°42.856'E, 20 m a.s.l.

##### Type material.

***Holotype***: male “Indonesia: Papua, Batanta Utara, 20 m, 14.ii.2006, 00.50.125S 130.42.856E, Tindige et al. (BH 12)” (MZB).

***Paratypes***: IN: West Papua: Raja Ampat Regency: Batanta: 34 males, 6 females with the same label as the holotype (MZB, NHMW, ZSM). For additional paratypes see Appendix 1.

##### Description.

***Body size and form***: Beetle small to medium-sized, with oblong-oval habitus (Figs 54, 55).

***Measurements***: TL 4.4–5.6 mm, TL-H 3.9–5.1 mm, MW 2.1–2.65 mm, TL/MW 2–2.11; PL 0.65–0.85 mm, PW 1.85–2.3 mm, PL/PW 0.35–0.37; DBE 0.8–0.95 mm, DBE/PW 0.41–0.43.

Holotype: TL 5 mm, TL-H 4.55 mm, MW 2.5 mm, TL/MW 2; PL 0.75 mm, PW 2.15 mm, PL/PW 0.35; DBE 0.9 mm, DBE/PW 0.42.

***Colouration***: Dorsally piceous, with yellowish red head, pronotal sides, and basal band and apical spot on elytron (Figs 54, 55).

Head yellowish red to reddish brown, darker narrowly behind eyes. Pronotum brown to piceous, paler towards sides, yellowish red to reddish brown on them. Elytron piceous, with yellow to reddish brown basal band starting at striae 1–3 and not reaching suture and lateral margin; with slightly to distinctly notched posterior margin; elytron with distinct, elongate spot apically. Scutellum yellowish red to piceous. Antennae and other head appendages yellow. Pro- and mesolegs yellow and metalegs yellowish red proximally and darker distally. Venter mostly yellowish red, with to yellow base of prosternum and abdominal ventrites 1–3, abdominal ventrites 4–6 yellowish red medially and paler laterally.

***Surface sculpture***: Elytron with 10–11 dorsal striae, complete or interrupted and reduced; submarginal stria present or absent: (10–11)+(0–1) (Figs 54, 55).

Head without strioles, with rather dense punctation (spaces between punctures 1–3× size of punctures); punctures relatively small (diameter of punctures equal to diameter of cells of microreticulation); head with a row of setigerous punctures along inner margin of each eye and a short row at frontal angle of each eye; a slightly longer puncture row forms fronto-clypeal depression at each head side; head with relatively strong microreticulation. Pronotum with sparse strioles in whole surface or with only few at posterior margin or posterior angles; with or without longitudinal wrinkles at posterior margin; pronotal punctation finer than on head; setigerous punctures form a broad row along pronotal margins, absent in posterior middle; disc of pronotum with thin, longitudinal median scratch. Pronotal microreticulation fine. Elytron usually with 11 dorsal striae; striae weakly to strongly impressed; odd striae shortly reduced apically; stria 1 or striae 1–3 shortly reduced basally; sometimes stronger stria reduction can take place: stria 1 reduced completely, elytron with only ten reduced and interrupted striae; submarginal stria present, well-developed, sometimes reaching ½ of elytron; in specimens with reduced stria pattern, absent or weak, short, apical. Elytron with fine punctation and microreticulation. Ventral part with fine, inconspicuous punctation, slightly visible on metaventrite and metacoxae and stronger on abdominal ventrites; prosternum smooth medially; metaventrite and metacoxae with fine microreticulation; on abdominal ventrites microreticulation almost invisible; metacoxal plates with short, numerous, rather sparse, distinctly impressed longitudinal strioles, abdominal ventrites 1 and 2 with numerous, long, longitudinal strioles from margin to margin, on abdominal ventrites 3 and 4 strioles situated laterally and turn to middle, almost horizontal, abdominal ventrites 5 and 6 without strioles but with fine punctation that sparser medially and forms a dense lateral area at each side.

***Structures***: Head relatively broad. Pronotum short and broad; lateral margins distinctly convergent anteriorly. Base of prosternum rounded anteriorly, convex medially; blade of prosternal process relatively narrow, convex in middle.

***Male***: Protibia straight, not modified. Proclaws simple, relatively long, subequal. Median lobe of aedeagus with two lobes of dorsal sclerite rather narrow; left lobe distinctly shorter than right one; in lateral left view, left dorsal lobe with long lateral crest and apex curved downwards; its dorsal surface with weak denticulation usually visible in left lateral view; right dorsal lobe with distinct median impression in right lateral view and with very “swollen”, rounded, pea-like apex. Lobes of ventral sclerite weakly sclerotised laterally, visible in left and right lateral views, mostly membranous, subequal, straight apically; sclerotised part of left ventral lobe long and straight apically, approximately 1/2 length of left dorsal lobe. Paramere with setae not clearly divided into distal and proximal; more distally situated setae distinctly denser than more proximal ones (Fig. 57).

***Female***: As male.

##### Variability.

There is a variation in the colouration and dorsal striolation described above.

##### Affinities.

The species is similar to *A.moreguinensis* sp. nov., their comparison see under this species.

##### Etymology.

The species is named after Raja Ampat Regency where it is widely distributed. The name is an adjective in the nominative singular.

##### Distribution.

New Guinean endemic. Indonesia: Papua Province: Raja Ampat and Sorong regencies (Fig. 84).

##### Habitat.

Unknown.

#### ﻿24. Austrelatus rouaffer sp. nov.

Taxon classificationAnimaliaColeopteraDytiscidae

B77062EA-13F8-5013-BC06-ED7852248FD6

https://zoobank.org/87F06161-7CB8-4515-A6C5-96640642E45E

Figs 66 , 70 , 84 , 90 , 93

##### Type locality.

Indonesia: Papua Province: Puncak Regency, Iratoi, near Rouaffer River, 03°14'25.1"S, 137°19'58.7"E, 164 m a.s.l.

##### Type material.

***Holotype***: male “Indonesia: Papua, Rouaffer, Iratoi, hill in forest, 164 m, 6.ix.2014, -3,2403 137,3329, Pele (Pap028)” (MZB).

***Paratypes***: 29 males, 14 females with the same label as the holotype (MZB, KSP, NHMW, ZSM). 6 males, 2 females “Indonesia: Papua, S Iratoi, hunting camp, 150 m, 28.v.2015, -3,28017423860728 137,334125172346, Sumoked (Pap042)”, one male with an additional green text label “7231” (KSP, ZSM).

##### Description.

***Body size and form***: Beetle small, with oblong-oval (Fig. 66).

***Measurements***: TL 4.8–5.5 mm, TL-H 4.35–5 mm, MW 2.35–2.7 mm, TL/MW 2.04; PL 0.7–0.8 mm, PW 2.05–2.35 mm, PL/PW 0.34–0.35; DBE 0.85–0.9 mm, DBE/PW 0.38–0.42.

Holotype: TL 5.3 mm, TL-H 4.7 mm, MW 2.6 mm, TL/MW 2.04; PL 0.8 mm, PW 2.3 mm, PL/PW 0.35; DBE 0.9 mm, DBE/PW 0.39.

***Colouration***: Dorsally piceous, with yellowish red median part of head, anterior angles of pronotum and an apical spot on elytron (Fig. 66).

Head yellowish red in middle and piceous anteriorly and posteriorly, seldom uniformly yellowish red. Pronotum piceous, with yellowish red anterior angles. Elytron piceous, with a distinct yellow elongate, small to large apical spot; seldom with two small basal spots, which can be confluent. Scutellum piceous. Antennae, other head appendages, and pro- and mesolegs proximally yellowish brown, metalegs reddish brown, legs darker distally. Venter dark brown. Teneral beetles paler.

***Surface sculpture***: Elytron with 11 complete, strongly impressed dorsal striae; submarginal stria present: 11+1 (Fig. 66).

Head without or with few strioles between eyes, with rather dense punctation (spaces between punctures 1–3× size of punctures); punctures relatively coarse (diameter of punctures equal to diameter of microreticulation cells); head with a row of coarse setigerous punctures along inner margin of each eye and a short row at frontal angle of each eye; a slightly longer puncture row forms fronto-clypeal depression at each head side; microreticulation distinct. Pronotum with numerous strioles, often also on disc; pronotal punctation finer than on head; setigerous punctures form a row along pronotal margins, absent in posterior middle; disc of pronotum with indistinct longitudinal median scratch. Pronotum with fine microreticulation. Elytron with 11 complete, strongly impressed dorsal striae; seldom striae 1 and 5 shortly reduced basally, stria 3, 5, 7 apically, stria 9 interrupted; sometimes strioles present between striae 10 and 11 almost forming a stria; seldom, very small solitary strioles present between suture and stria 1, striae 5 and 6, 9 and 10, 11 and submarginal stria; submarginal stria present, well-developed, long, reaching middle or even more of elytron. Elytron with fine punctation and microreticulation. Ventral part with fine, inconspicuous punctation, invisible on metaventrite and metacoxae and weak on abdominal ventrites; prosternum smooth medially; metaventrite and metacoxae with weak microreticulation; on abdominal ventrites microreticulation almost invisible; metacoxal plates with numerous, distinctly impressed longitudinal strioles, abdominal ventrites 1 and 2 with numerous, long, longitudinal strioles from margin to margin, on abdominal ventrites 3 and 4 strioles situated laterally and turn to middle, almost horizontal, abdominal ventrites 5 and 6 without strioles but with fine punctation that sparser medially and forms a dense, rugose lateral area at each side.

***Structures***: Head relatively broad. Pronotum short and broad; lateral margins distinctly convergent anteriorly. Base of prosternum broadly rounded anteriorly, convex medially; blade of prosternal process large, broad, convex in middle.

***Male***: Protibia straight, not modified. Proclaws relatively short, subequal in length; anterior claw slightly more strongly curved downwards than posterior. Median lobe of aedeagus with two lobes of dorsal sclerite rather narrow; left dorsal lobe distinctly shorter that right one, with a lateral crest interrupted into apical and basal parts; apex of left dorsal lobe distinctly curved downwards and to left, dorsally with denticulation (spinulae) visible in lateral left view due to curvature to left; right dorsal lobe with small, indistinct, elongate median impression and modified apex: large, swollen, rounded; left lobe of ventral sclerite with its sclerotised area rather large, broad, slightly concave, rounded apically, shorter than right ventral and dorsal lobes and slightly more than 1/2 length of left dorsal lobe. Paramere with setae distinctly divided into distal and proximal; proximal setae distinctly sparser and shorter than distal, sometimes present only as few setae in right paramere (Fig. 70).

***Female***: Dimorphic. Forms with strioles are rare. Ratio shiny to with strioles is 13: 1 in the locality Pap028.

##### Variability.

There is an insignificant variation in the colouration and dorsal striolation described above.

##### Affinities.

In general shape of median lobe, especially in shape of the sclerotised area of left ventral lobe and in shape of the lateral crest of the left dorsal lobe, the species is similar to *A.lembenensis* sp. nov., *A.fojaensis* sp. nov., and *A.innominatus* sp. nov. The species can be distinguished from the first two species by elytron with 11+1 striae and without basal yellowish spots, pronotum with numerous strioles, and shape of the median lobe sclerites. From *A.innominatus* sp. nov., it differs by pronotum with numerous strioles, elytron with 11+1 striae more complete and more strongly impressed and without basal yellowish spots and shape of the median lobe sclerites.

##### Etymology.

The species is named after Rouaffer River. The species name is a noun in the nominative singular standing apposition.

##### Distribution.

New Guinean endemic. Indonesia: Papua Province: Puncak Regency (Fig. 84).

##### Habitat.

At the type locality, the species was collected in a large forest stream (Fig. 90); at the locality PAP042 in a forest pool (Fig. 93).

#### ﻿25. Austrelatus rugosus sp. nov.

Taxon classificationAnimaliaColeopteraDytiscidae

D9178DB4-0798-51B8-8FF8-A0F1458651BA

https://zoobank.org/31825EF0-0F68-49FC-B295-6811CAE866DD

Figs 75 , 79 , 84 , 87 , 90

##### Type locality.

Indonesia: Indonesia: Papua Province: Puncak Regency, Iratoi, near Rouaffer River, 03°14'25.1"S, 137°19'58.7"E, 164 m a.s.l.

##### Type material.

***Holotype***: male “Indonesia: Papua, Rouaffer, Iratoi, hill in forest, 164 m, 6.ix.2014, -3,2403 137,3329, Sumoked (PAP028)” (MZB).

***Paratypes***: IN: Papua: Puncak Regency: 8 males, 9 females, with the same label as the holotype, two males with green text additional labels “6473”, “6474” (KSP, MZB, NHMW, ZSM).

Nabire Regency: 8 males, 8 females “Irian Jaya: Paniai Prov. road Nabire – Ilaga, km 65, 29.8.1996, 250 m (96 # 6)” (NHMW, ZSM). 3 males “West New Guinea/Paniai Prov./IR 22 track Nabire-Ilaga km 62 250 m, 24.7.1991, forest pools leg: Balke & Hendrich” (CLH, ZSM). 1 female “West New Guinea/Paniai Prov./IR 18 River n. Nabire, 2 m, 15.7.1991 leg: Balke & Hendrich leg.” (CLH). 1 female “West New Guinea/Paniai Prov./IR 19 track Nabire-Ilaga km 54 Basecamp, 750–800 m, 16.-27.7.1991 leg: Balke & Hendrich” (ZSM). 1 male “Irian Jaya: Paniai Prov. road Nabire – Ilaga, km 54, 10.9.1996, 900 m (96 # 19)” (NHMW).

West Papua: Kaimana Regency: 1 male, 2 females “Irian Jaya: Fak Fak dist. Lake Yamur area, IV.1998 ca. 50 – 100 m, Waldtümpel, Konyorah” (NHMW).

Teluk Wondama Regency: 6 males “Irian Jaya: Wandammen Bay, Wasior, Sararti 100–200 m, 7–9.I.2001 leg. A. Riedel” (SMNS).

##### Description.

***Body size and form***: Beetle small, with oblong-oval habitus (Fig. 75).

***Measurements***: TL 4.15–4.7 mm, TL-H 3.8–4.25 mm, MW 2–2.3 mm, TL/MW 2.04–2.08; PL 0.6–0.7 mm, PW 1.75–2 mm, PL/PW 0.34–0.35; DBE 0.75–0.8 mm, DBE/PW 0.4–0.42.

Holotype: TL 4.65 mm, TL-H 4.2 mm, MW 2.25 mm, TL/MW 2.07; PL 0.65 mm, PW 1.9 mm, PL/PW 0.34; DBE 0.8 mm, DBE/PW 0.42.

***Colouration***: Dorsally reddish brown to dark brown, with reddish head and pronotum, elytron with yellow-red to dark brown basal band or spots and vague spot on apex (Fig. 75).

Head yellowish red to reddish brown, sometimes darker narrowly behind eyes. Pronotum yellowish red to reddish brown, paler on sides, often darker at anterior and posterior margins. Elytron reddish brown to dark brown, sometimes concolourous with head and pronotum, with yellowish red to reddish brown basal band at its middle due to confluence of two spots; one of spots normally larger, therefore band with notched posterior margin; sometimes band can be extended to suture due to presence of one more spot; seldom spots not confluent, distinct; band can be very distinct or very vague so that elytron seems to be uniformly coloured or slightly paler basally; elytron with distinct or vague, rather small, elongate spot apically. Scutellum yellowish red to brown, usually concolourous with elytra. Antennae and other head appendages yellow. Pro- and mesolegs yellow and metalegs yellowish red proximally and darker distally. Venter mostly reddish brown, with yellowish red prosternum and abdominal ventrites 1–3.

***Surface sculpture***: Elytron with 11 distinct, complete dorsal striae; submarginal stria present: 11+1. Dorsal surface with tiny strioles (Fig. 75).

Head with few strioles only between eyes or medially, with rather sparse punctation (spaces between punctures 1–4× size of punctures); punctures relatively small (diameter of punctures equal to or slightly smaller than diameter of cells of microreticulation); head with a row of setigerous punctures along inner margin of each eye and a short row at frontal angle of each eye; a slightly longer puncture row forms fronto-clypeal depression at each head side; head with relatively strong microreticulation. Pronotum with numerous, dense or rather sparse, strioles in whole surface, seldom only laterally and posteriorly, without longitudinal wrinkles; pronotal punctation finer and sparser than on head; setigerous punctures form a broad row along pronotal margins, absent in posterior middle; disc of pronotum with thin, longitudinal median scratch. Pronotal microreticulation distinct. Elytron with 11 complete, strongly or less strongly impressed dorsal striae; odd striae shortly reduced apically; submarginal stria present, very long, almost reaching elytral base. Elytron with fine or rather distinct punctation and distinct microreticulation; additionally, to distinct punctures, tiny strioles can be present, especially in females. Ventral part with fine, inconspicuous punctation, slightly visible on metaventrite and metacoxae and stronger on abdominal ventrites; prosternum smooth medially; metaventrite and metacoxae with fine microreticulation medially; on abdominal ventrites microreticulation almost invisible; metacoxal plates with short, numerous, rather sparse, strongly impressed longitudinal strioles, abdominal ventrites 1 and 2 with numerous, long, longitudinal strioles from margin to margin, on abdominal ventrites 3 and 4 strioles situated laterally and turn to middle, almost horizontal, abdominal ventrites 5 and 6 without strioles but with fine punctation that sparser medially and forms a dense lateral area at each side.

***Structures***: Head relatively broad. Pronotum short and broad; lateral margins distinctly convergent anteriorly. Base of prosternum rounded anteriorly, convex medially; blade of prosternal process relatively narrow, convex in middle.

***Male***: Protibia straight, not modified. Proclaws simple, relatively short, subequal. Median lobe of aedeagus with two lobes of dorsal sclerite rather narrow; left lobe distinctly shorter than right one; in lateral left view, left dorsal lobe with a lateral crest interrupted into apical and basal parts, lobe concave along rather long basal crest; apex of left dorsal lobe relatively short and slightly curved downwards; its dorsal surface without denticulation, but with a strong median crest visible in left lateral view; right dorsal lobe with well-developed, more laterally situated median impression in right lateral view, with broad, “swollen”, rounded apex. Lobes of ventral sclerite weakly sclerotised laterally, visible in left and right lateral views, mostly membranous, equal, straight apically; sclerotised part of left ventral lobe long, thin, and straight apically, more than 2/3 of length of left dorsal lobe. Paramere with setae not clearly divided into distal and proximal; more distally situated setae usually denser than more proximal ones (Fig. 79).

***Female***: There are females without evident differences from males in surface sculpture; there are some with coarser and denser dorsal punctation and more strongly impressed reticulation (in Iratoi, Wasior and Fakfak); there are specimens with numerous, dense, tiny elytral strioles (in Nabire Province). However, there are no strongly striolated, matt forms.

##### Variability.

There is a strong variation in the colouration and dorsal striolation described above. For the specimens from the type locality (96 # 6, road Nabire-Enarotali), more elongate body shape, paler dorsal colouration (when elytron almost concolourous with head and pronotum), more weakly impressed elytral striae, and presence of tiny elytral strioles instead of punctures (especially in females) are characteristic. The specimens from Iratoi, Wasior, and Fakfak are more oval, darker, with more strongly impressed elytral striae, and without tiny elytral strioles. Shape of the median lobe constant, with an insignificant variation in sclerite shape.

##### Affinities.

Based on shape of the median lobe (especially shape of the sclerotised part of left ventral lobe), pronotal and elytral striation, and in some measure dorsal colouration, the species is close to *A.vagauensis* sp. nov. but distinctly differs from it in much smaller body size and straighter median lobe sclerites. The species is also very similar to *A.debulensis* sp. nov. in dorsal colouration and striation and especially in shape of median lobe. However, we consider it as a separate species because of more strongly developed dorsal striolation, shorter and more strongly curved downwards apical part of left dorsal lobe and more laterally situated median impression of the right ventral lobe.

##### Etymology.

The species name is a Latin adjective meaning rugose, wrinkled, to describe a distinct striolation of the pronotum and elytra in both sexes.

##### Distribution.

New Guinean endemic. Indonesia: West Papua Province: Kaimana and Teluk Wondama regencies and Papua Province: Nabire and Puncak regencies (Fig. 84).

##### Habitat.

At the type locality, the species was collected in a large forest stream (Fig. 90). At the Nabire-Ilaga track, all specimens were collected in shallow (up to 20 cm water depth), shaded or at least partly shaded forest pools (e.g., Fig. 87) and puddles of different size, rich in rotten leaves and twigs; few specimens were also found in water-filled track hollows on forest tracks.

#### ﻿26. Austrelatus sandaunensis sp. nov.

Taxon classificationAnimaliaColeopteraDytiscidae

363775D9-29BE-5FA5-94F6-49023E31B663

https://zoobank.org/CF860F0D-8A65-4F3D-B242-2B416769F799

Figs 69 , 73 , 84

##### Type locality.

Papua New Guinea: Sandaun, Mianmin, 04°53.292'S, 141°34.118'E, 670 m a.s.l.

##### Type material.

***Holotype***: male “Papua New Guinea: Sandaun, Mianmin, 670 m 20.x.2008, 4.53.292S 141.34.118E, Ibalim (PNG 191)” (ZSM).

***Paratypes***: 7 males, 4 females with the same label as the holotype (NHMW, ZSM). 1 female “Papua New Guinea: Sandaun, Mianmin, 670 m, 22.x.2008, 04.53.329S 141.35.263E S. Ibalim PNG189” (ZSM). 1 male “Papua New Guinea: Sandaun, Mianmin (river), 1080 m, 24.x.2008, 04.55.780S 141.38.185E, Ibalim (PNG 195)”, “3773” [green label] (ZSM). 1 male “Papua New Guinea: Sandaun, Mianmin (pool), 1080 m, 24.x.2008, 04.55.780S 141.38.185E, Ibalim (PNG 196)” (ZSM). 1 male, 1 female “Papua New Guinea: Sandaun, Mianmin (river), 700 m, 21.x.2008, 04.52.858S 141.31.706E Ibalim (PNG 197)” (ZSM). 3 males “Papua New Guinea: Sandaun, Mianmin (pool), 700 m, 21.x.2008, 04.52.858S 141.31.706E, Ibalim (PNG 198)” (NHMW, ZSM).

##### Description.

***Body size and form***: Beetle medium-sized, with oblong-oval habitus (Fig. 69).

***Measurements***: TL 5.1–6 mm, TL-H 4.6–5.35 mm, MW 2.5–3 mm, TL/MW 2–2.04; PL 0.75–0.9 mm, PW 2.2–2.5 mm, PL/PW 0.34–0.36; DBE 0.9–1 mm, DBE/PW 0.39–0.41.

Holotype: TL 5.6 mm, TL-H 5.1 mm, MW 2.75 mm, TL/MW 2.04; PL 0.85 mm, PW 2.45 mm, PL/PW 0.35; DBE 0.95 mm, DBE/PW 0.39.

***Colouration***: Dorsally piceous, with yellowish red head, narrow pronotal sides and a basal band and one apical spot on elytron (Fig. 69).

Head yellowish red to reddish brown, piceous narrowly behind eyes. Pronotum dark brown to piceous on disc and paler towards sides, yellowish red on them. Elytron piceous, with three yellowish red to reddish brown basal spots, usually confluent, forming a basal band, not reaching suture and lateral margin; first spot between striae 1 and 3 vague or absent; therefore, band usually formed by two more lateral spots; elytron with a distinct, small, elongate spot apically. Scutellum yellowish red to dark brown. Antennae and other head appendages yellow. Pro- and mesolegs yellow and metalegs yellowish red proximally and darker distally. Venter yellowish red to brown, sometimes darker medially and paler laterally, especially on abdominal ventrites; prosternum yellowish red.

***Surface sculpture***: Elytron with 11 complete dorsal striae; submarginal stria present: 11+1 (Fig. 69).

Head without strioles, with rather dense punctation (spaces between punctures 1–2× size of punctures); punctures relatively coarse (diameter of punctures equal to or larger than diameter of microreticulation cells); head with a row of coarse setigerous punctures along inner margin of each eye and a short row at frontal angle of each eye; a slightly longer puncture row forms fronto-clypeal depression at each head side; microreticulation distinct. Pronotum usually with several strioles at posterior margin or rather in posterolateral angles; with fine longitudinal wrinkles at posterior margin; pronotal punctation finer than on head; setigerous punctures form a row along pronotal margins, absent in posterior middle; disc of pronotum with indistinct longitudinal median scratch. Pronotum with distinct microreticulation. Elytron with 11 dorsal complete striae, often with fine, single strioles basally between striae 10 and 11; odd striae shortly reduced apically; submarginal striae present, weakly developed, short, apical, interrupted. Elytron with fine punctation and microreticulation. Ventral part with fine, inconspicuous punctation, invisible on metaventrite and metacoxae and weak on abdominal ventrites; prosternum smooth medially; metaventrite and metacoxae with distinct microreticulation; on abdominal ventrites microreticulation fine; metacoxal plates with numerous, strongly impressed longitudinal strioles, abdominal ventrites 1 and 2 with numerous, long, longitudinal strioles from margin to margin, on abdominal ventrites 3 and 4 strioles situated laterally and turn to middle, almost horizontal, abdominal ventrites 5 and 6 without strioles but with fine punctation that sparser medially and forms a dense, rugose lateral area at each side.

***Structures***: Head relatively broad. Pronotum short and broad; lateral margins distinctly convergent anteriorly. Base of prosternum rounded anteriorly, convex medially; blade of prosternal process relatively narrow, convex in middle.

***Male***: Protibia straight, not modified. Proclaws simple, relatively long, equal. Median lobe of aedeagus with two lobes of dorsal sclerite rather narrow, subequal; in lateral left view, left dorsal lobe with short, apical crest and weaker lateral crest; its apex more or less straight, pressed to right dorsal lobe; right dorsal lobe with weakly developed, inconspicuous median impression in right lateral view, more or less rounded apex and small crest on its left side where apex of left dorsal lobe placed in left lateral view. Lobes of ventral sclerite weakly sclerotised laterally visible in left and right lateral views, mostly membranous, straight apically; left ventral lobe distinctly shorter than right ventral lobe; sclerotised part of left ventral lobe long, thin, and straight apically in left lateral view. Paramere with setae not clearly divided into distal and proximal; sometimes few the most proximally setae standing something separately (Fig. 73).

***Female***: With coarser and denser dorsal punctation, especially on elytron. There are no striolated, matt forms.

##### Variability.

There is an insignificant variation in the colouration and dorsal striolation described above.

##### Affinities.

Based on shape of the median lobe, the species is very similar to *A.toricelli* sp. nov. but differs from it in complete 11 dorsal striae of elytron and different shape of the median lobe sclerites: differently shaped left dorsal lobe and left ventral lobe distinctly shorter that right ventral lobe.

##### Etymology.

The species is named after Sandaun Province. The name is an adjective in the nominative singular.

##### Distribution.

New Guinean endemic. Papua New Guinea: Sandaun Province. The species is known only from the Mianmin area (Fig. 84).

##### Habitat.

The species was collected in stream-side puddles.

#### ﻿27. Austrelatus sarmiensis sp. nov.

Taxon classificationAnimaliaColeopteraDytiscidae

932823CF-72F0-5B18-9E07-3662F6CCD93E

https://zoobank.org/EF3F4034-B7F1-42AA-B37A-2F27DC83AAE5

Figs 74 , 78 , 84

##### Type locality.

Indonesia: Papua Province: Sarmi Regency, Sarmi area, 01°58'17.0"S, 138°50'56.9"E, 70 m a.s.l.

##### Type material.

***Holotype***: male “Indonesia: Papua, Sarmi area 70 m, 25.ix.2014, -1.9713 138.8491, Menufandu (Pap032)” (MZB).

***Paratype***: 1 female with the same label as the holotype (ZSM).

##### Description.

***Body size and form***: Beetle small, with oblong-oval habitus (Fig. 74).

***Measurements***: TL 4.95–5.2 mm, TL-H 4.45–4.7 mm, MW 2.4–2.6 mm, TL/MW 2–2.06; PL 0.75–0.8 mm, PW 2.1–2.2 mm, PL/PW 0.36; DBE 0.85–0.9 mm, DBE/PW 0.41.

Holotype: TL 5.2 mm, TL-H 4.7 mm, MW 2.6 mm, TL/MW 2; PL 0.8 mm, PW 2.2 mm, PL/PW 0.36; DBE 0.9 mm, DBE/PW 0.41.

***Colouration***: Dorsally piceous, with yellowish red head (with brown spot medially) and pronotal sides, and with three reddish, vague, indistinct spots on elytral base and more distinct yellowish red spot on elytral apex (Fig. 74).

Head yellowish red, piceous narrowly behind eyes, brown anteriorly, and with large, brown median spot. Pronotum piceous, yellowish red narrowly on sides, broader at anterior angles. Elytron piceous, with three very vague, indistinct, yellowish red to reddish basal spots; most distinct spot at base of stria 10; at apex, with a distinct, rather small, elongate spot. Scutellum reddish brown to brown. Antennae and other head appendages yellow. Pro- and mesolegs yellow and metalegs yellowish red proximally and darker distally. Venter mostly piceous, with yellowish red prosternum.

***Surface sculpture***: Elytron with 11 distinct, complete dorsal striae; submarginal stria present: 11+1 (Fig. 74).

Head with strioles between eyes, with rather dense punctation (spaces between punctures 1–3× size of punctures); punctures relatively small (diameter of punctures slightly smaller than diameter of cells of microreticulation); head with a row of setigerous punctures along inner margin of each eye and a short row at frontal angle of each eye; a slightly longer puncture row forms fronto-clypeal depression at each head side; head with relatively strong microreticulation. Pronotum with numerous strioles in whole surface, without longitudinal wrinkles; pronotal punctation finer and sparser than on head; setigerous punctures form a broad row along pronotal margins, absent in posterior middle; disc of pronotum with thin, longitudinal median scratch. Pronotal microreticulation distinct. Elytron with 11 distinct, complete dorsal striae; odd striae shortly reduced apically; stria 10 shortly reduced basally; submarginal stria present, rather short, not reaching ½ of elytron. Elytron with fine punctation and distinct microreticulation. Ventral part with fine, inconspicuous punctation, slightly visible on metaventrite and metacoxae and stronger on abdominal ventrites; prosternum smooth medially; metaventrite and metacoxae with distinct microreticulation medially; on abdominal ventrites microreticulation almost invisible; metacoxal plates with short, numerous, strongly impressed longitudinal strioles, abdominal ventrites 1 and 2 with numerous, long, longitudinal strioles from margin to margin, on abdominal ventrites 3 and 4 strioles situated laterally and turn to middle, almost horizontal, abdominal ventrites 5 and 6 without strioles but with rather distinct punctation that sparser medially and forms a dense lateral area at each side.

***Structures***: Head relatively broad. Pronotum short and broad; lateral margins distinctly convergent anteriorly. Base of prosternum rounded anteriorly, convex medially; blade of prosternal process relatively broad, convex in middle.

***Male***: Protibia straight, not modified. Proclaws simple, relatively long, equal. Median lobe of aedeagus with two lobes of dorsal sclerite rather narrow; left lobe distinctly shorter than right one; left dorsal lobe almost straight to apex, with weak, longitudinal crest, its dorsal surface with distinct denticulation visible in left lateral view; right dorsal lobe with weakly developed, inconspicuous median impression in right lateral view, with broad, rounded apex. Lobes of ventral sclerite weakly sclerotised laterally, visible in left and right lateral views, mostly membranous, subequal, straight apically; sclerotised part of left ventral lobe long, thin, and straight apically, 2/3 of length of left dorsal lobe. Paramere with setae not divided into distal and proximal; more distally situated setae denser than more proximal ones (Fig. 78).

***Female***: With coarser and denser dorsal punctation, especially on elytron. There are no striolated, matt forms.

##### Affinities.

Based on shape of the median lobe, pronotal and elytral striolation, and in some measure dorsal colouration, the species is very similar to *A.vagauensis* sp. nov. but distinctly differs from it in much smaller body size and straighter median lobe sclerites.

##### Etymology.

The species is named after Sarmi area. The name is an adjective in the nominative singular.

##### Distribution.

New Guinean endemic. Indonesia: Papua Province: Sarmi Regency. The species is known only from the type locality (Fig. 84).

##### Habitat.

The species was collected in stream-side puddles and pools of an intermittent stream.

#### ﻿28. Austrelatus securiformis sp. nov.

Taxon classificationAnimaliaColeopteraDytiscidae

F0BF922B-D927-5AC9-8AF6-01DF5326CEAE

https://zoobank.org/81D13E76-3C65-47A4-B18B-7EE49C8B0818

Figs 38 , 42 , 83 , 93

##### Type locality.

Indonesia: Papua Province: Puncak Regency, S Iratoi, hunting camp, 03°16'48.6"S, 137°20'02.9"E, 150 m a.s.l.

##### Type material.

***Holotype***: male “Indonesia: Papua, S Iratoi, hunting camp, 150 m, 28.v.2015, -3,2801 137,3341, Sumoked (PAP042)”, “7230” [green text] (MZB).

##### Description.

***Body size and form***: Beetle small, with oblong-oval habitus (Fig. 38).

***Measurements***: Holotype: TL 5.3 mm, TL-H 4.8 mm, MW 2.65 mm, TL/MW 2; PL 0.8 mm, PW 2.3 mm, PL/PW 0.35; DBE 0.9 mm, DBE/PW 0.39.

***Colouration***: Dorsally piceous, with yellowish brown head, pronotal sides, and an apical spot on elytron (Fig. 38).

Head yellowish brown, darker narrowly behind eyes. Pronotum piceous on disc and paler towards sides, yellowish brown on them, yellow at anterior angles. Elytron piceous, with a distinct, elongate, yellow apical spot. Scutellum piceous. Antennae, other head appendages, and legs proximally yellow; legs darker distally, yellowish brown, especially metalegs. Venter brown, with paler prosternum.

***Surface sculpture***: Elytron with 6 complete dorsal striae; submarginal stria present but interrupted, apical: 6+1 (Fig. 38).

Head without strioles, with dense punctation (spaces between punctures 1–3× size of punctures); punctures relatively coarse (diameter of punctures usually equal to diameter of microreticulation cells or larger than it); head with a row of setigerous punctures along inner margin of each eye and a short row at frontal angle of each eye; a slightly longer puncture row forms fronto-clypeal depression at each head side; microreticulation strong. Pronotum with strioles laterally at posterior margin, especially in posterolateral angles; with fine longitudinal wrinkles at posterior margin; pronotal punctation distinctly finer than on head; setigerous punctures form a row along pronotal margins, absent in posterior middle; disc of pronotum with indistinct longitudinal median scratch. Pronotum with distinct microreticulation. Elytron with 6 dorsal striae; striae 1–4 and 6 complete, stria 5 usually shortly reduced basally; submarginal striae weakly developed, present apically, interrupted. Elytron with fine punctation and distinct microreticulation. Ventral part with fine, inconspicuous punctation, invisible on metaventrite and metacoxae and more distinct on abdominal ventrites; prosternum smooth medially; metaventrite and metacoxae with distinct microreticulation; on abdominal ventrites microreticulation weak; metacoxal plates with numerous, strongly impressed longitudinal strioles, abdominal ventrites 1 and 2 with numerous, long, longitudinal strioles from margin to margin, on abdominal ventrites 3 and 4 strioles situated laterally and turn to middle, almost horizontal, abdominal ventrites 5 and 6 without strioles but with distinct punctation that sparser medially and forms a dense, rugose lateral area at each side.

***Structures***: Head relatively broad. Pronotum short and broad; lateral margins distinctly convergent anteriorly. Base of prosternum narrowly rounded anteriorly, slightly convex medially; blade of prosternal process relatively narrow, convex in middle.

***Male***: Protibia straight, not modified. Proclaws subequal in length, relatively short, curved downwards basally. Median lobe of aedeagus with two lobes of dorsal sclerite rather narrow, left dorsal lobe shorter that right one; in lateral view, apex of left dorsal lobe slightly curved upwards, with small crest; left dorsal lobe dorsally with distinct denticulation (spinulae) visible also in lateral left view; right dorsal lobe with small, indistinct, elongate median impression and modified apex: relatively large, swollen, rounded; left lobe of ventral sclerite with its sclerotised area large, curved to left, axe-shape truncate apically, distinctly shorter than right ventral lobe and dorsal lobes. Paramere with setae not divided into distal and proximal; more distally situated setae slightly denser than more proximal ones (Fig. 42).

***Female***: Unknown.

##### Affinities.

In dorsal colouration and elytron with 6+1 striae, the species is similar to *A.testegensis* sp. nov. but differs from it in absence of strioles between the elytral striae and shape of the proclaws and median lobe.

##### Etymology.

The species is named for the axe-like apex of the left ventral lobe of the median lobe. The name is Latin word combination *securis* and *forma*. It is an adjective in the nominative.

##### Distribution.

New Guinean endemic. Indonesia: Papua Province: Puncak Regency. The species is known only from its type locality (Fig. 83).

##### Habitat.

The species was collected in a forest pool (Fig. 93).

#### ﻿29. Austrelatus testegensis sp. nov.

Taxon classificationAnimaliaColeopteraDytiscidae

CFAD9D8F-9CE0-5DA1-B257-B6577E188CD0

https://zoobank.org/A27A8F25-4C11-4C2E-BD69-E284903159A7

Figs 39 , 43 , 83

##### Type locality.

Indonesia: West Papua Province: Manokwari Regency, Testega, 01°22'07.3"S, 133°35'27.1"E, 1212 m a.s.l.

##### Type material.

***Holotype***: male “Indonesia: Papua Barat, Testega, 1212 m, 3.v.2015, -1,3686 133,5908, UNIPA team (BH054)” (MZB).

##### Description.

***Body size and form***: Beetle small, with oblong-oval habitus (Fig. 39).

***Measurements***: Holotype: TL 5.1 mm, TL-H 4.6 mm, MW 2.4 mm, TL/MW 2.13; PL 0.7 mm, PW 2.1 mm, PL/PW 0.33; DBE 0.9 mm, DBE/PW 0.43.

***Colouration***: Dorsally piceous, with yellowish red head and pronotal sides (Fig. 39).

Head yellowish red, darker narrowly behind eyes. Pronotum piceous on disc and yellowish red on lateral sides, yellow at anterior angles. Elytron uniformly piceous, paler near suture and at apex. Scutellum piceous. Antennae, other head appendages, and legs proximally yellow; legs darker distally, yellowish red, especially metalegs. Venter yellowish red, with paler prosternum.

***Surface sculpture***: Elytron with six complete dorsal striae; submarginal stria present: 6+1 (Fig. 39).

Head without strioles, with relatively sparse punctation (spaces between punctures 1–4× size of punctures); punctures fine (diameter of punctures usually equal to diameter of microreticulation cells or smaller than it); head with a row of setigerous punctures along inner margin of each eye and a short row at frontal angle of each eye; a slightly longer puncture row forms fronto-clypeal depression at each head side; microreticulation distinct. Pronotum with strioles at posterior margin, especially in posterolateral angles, and few anteriorly at middle; with fine longitudinal wrinkles at posterior margin; pronotal punctation finer than on head; setigerous punctures form a row along pronotal margins, absent in posterior middle; disc of pronotum with indistinct longitudinal median scratch. Pronotum with distinct microreticulation. Elytron with six dorsal striae; striae 2–4 and 6 complete, striae 1 and 5 shortly reduced basally; several strioles present between striae, especially numerous between striae 2–4; submarginal striae well-developed, present in apical half. Elytron with fine punctation and distinct microreticulation. Ventral part with fine, inconspicuous punctation, invisible on metaventrite and metacoxae and more distinct on abdominal ventrites; prosternum smooth medially; metaventrite and metacoxae with distinct microreticulation; on abdominal ventrites microreticulation almost invisible; metacoxal plates with numerous, weakly impressed longitudinal strioles, abdominal ventrites 1 and 2 with numerous, long, longitudinal strioles from margin to margin, on abdominal ventrites 3 and 4 strioles situated laterally and turn to middle, almost horizontal, abdominal ventrites 5 and 6 without strioles but with very fine punctation that sparser medially and forms a dense, rugose lateral area at each side.

***Structures***: Head broad. Pronotum short and broad; lateral margins distinctly convergent anteriorly. Base of prosternum broadly rounded anteriorly, convex medially; blade of prosternal process rather broad, convex in middle.

***Male***: Protibia straight, not modified. Proclaws simple, rather long, subequal in length. Median lobe of aedeagus with two lobes of dorsal sclerite rather narrow; left dorsal lobe distinctly shorter that right one, with weak, long lateral crest and apex evenly curved downwards and to left, dorsally with distinct denticulation (spinulae) visible also in lateral left view; right dorsal lobe with small, indistinct, elongate median impression and modified apex: relatively large, slightly swollen, broadly rounded; left lobe of ventral sclerite with its sclerotised area large, rounded apically, shorter than right ventral and dorsal lobes and approximately ½ of length of left dorsal lobe. Paramere with setae not divided into distal and proximal; more distally situated setae denser than more proximal ones (Fig. 43).

***Female***: Unknown.

##### Affinities.

In general shape of median lobe, especially in shape of the sclerotised area of left ventral lobe and in shape of the lateral crest of the left dorsal lobe, the species is similar to *A.fakfak* sp. nov., *A.manokwariensis* sp. nov., and *A.wanggarensis* sp. nov. The latter can be distinguished from *A.testegensis* sp. nov. by 11+1 elytral striae, presence of elytral maculation, and shape and size of the median lobe sclerites. *Austrelatustestegensis* sp. nov. is most similar in the shape of median lobe sclerites to *A.fakfak* sp. nov. From it and *A.manokwariensis* sp. nov., *A.testegensis* sp. nov. differs by the uniform colouration of the elytron and presence of strioles on the pronotum and especially on elytron, as well as in shape and size of the median lobe sclerites.

##### Etymology.

The species is named after its type locality, Testega Village. The name is an adjective in the nominative singular.

##### Distribution.

New Guinean endemic. Indonesia: West Papua Province: Manokwari Regency. The species is known only from its type locality (Fig. 83).

##### Habitat.

The species was collected in stream-side puddles.

#### ﻿30. Austrelatus toricelli sp. nov.

Taxon classificationAnimaliaColeopteraDytiscidae

7348419D-99BB-53AB-93E8-23ADBF2D7FCA

https://zoobank.org/2753F39B-6316-48C5-A137-31EC383F4CB9

Figs 47 , 51 , 83

##### Type locality.

Papua New Guinea: Sandaun Province, Toricelli Mountains, south from Sibilanga Station, 03°39'07.3"S, 142°29'59.5"E, 350 m a.s.l.

##### Type material.

***Holotype***: male “Papua New Guinea: Sandaun, Toricelli Mts., 2h walk fr Sibilanga Stn, 350 m, 19.iv.2006, 03.39.121S 142.29.991E, Balke (PNG 44)” (ZSM).

##### Description.

***Body size and form***: Beetle medium-sized, with oblong-oval habitus (Fig. 47).

***Measurements***: Holotype: TL 5.7 mm, TL-H 5.15 mm, MW 2.75 mm, TL/MW 2.07; PL 0.85 mm, PW 2.4 mm, PL/PW 0.35; DBE 0.95 mm, DBE/PW 0.4.

***Colouration***: Dorsally dark brown, with almost piceous elytra, yellowish red head, pronotal sides, and spots on elytral base and apex (Fig. 47).

Head yellowish red, dark brown narrowly behind eyes. Pronotum yellowish red to reddish brown, with darker anterior and posterior margins and narrowly disc and paler sides; darkest parts along middle of anterior and posterior margins. Elytron piceous, dark brown narrowly on disc, with two yellowish red basal spots medially, confluent, forming a short band, not reaching lateral elytral margin; at base of striae 1 and 2, with a very vague brownish spot; at apex, with a distinct, rather small, elongate spot. Scutellum reddish brown. Antennae and other head appendages yellow. Pro- and mesolegs yellow and metalegs yellowish red proximally and darker distally. Venter mostly yellowish red.

***Surface sculpture***: Elytron with 10 dorsal striae (most of them complete) due to strong reduction to few strioles of stria 1 (using 11- stria pattern); submarginal stria present: 10+1 (Fig. 47).

Head without strioles, with sparse, inconspicuous punctation (spaces between punctures 1–4× size of punctures); punctures relatively small, weakly impressed (diameter of punctures slightly smaller than diameter of cells of microreticulation); head with a row of setigerous punctures along inner margin of each eye and a short row at frontal angle of each eye; a slightly longer puncture row forms fronto-clypeal depression at each head side; these rows weakly impressed; head with relatively strong microreticulation. Pronotum with sparse strioles along posterior margin, denser laterally, with longitudinal wrinkles at middle of posterior margin; pronotal punctation slightly finer than on head; setigerous punctures form a broad row along pronotal margins, absent in posterior middle; disc of pronotum with thin, longitudinal median scratch. Pronotal microreticulation rather weakly impressed on disc. Elytron with ten more or less complete dorsal striae. Using 11-stria pattern: striae 2, 4, 6, 8, 10, and 11 complete, stria 1 absent or present as fine strioles in apical half, striae 3, 5, and 7 interrupted in whole their length, stria 9 present only in basal 1/3 of elytron. Elytron with very fine, inconspicuous punctation; microreticulation weak. Ventral part with fine, inconspicuous punctation, slightly visible on metaventrite and metacoxae and stronger on abdominal ventrites; prosternum smooth medially; metaventrite and metacoxae with distinct microreticulation medially; on abdominal ventrites microreticulation almost invisible; metacoxal plates with short, numerous longitudinal strioles, abdominal ventrites 1 and 2 with numerous, long, longitudinal strioles from margin to margin, on abdominal ventrites 3 and 4 strioles situated laterally and turn to middle, almost horizontal, abdominal ventrites 5 and6 without strioles but with rather distinct punctation that sparser medially and forms a dense lateral area at each side.

***Structures***: Head relatively broad. Pronotum short and broad; lateral margins distinctly convergent anteriorly. Base of prosternum rounded anteriorly, convex medially; blade of prosternal process relatively broad, convex in middle.

***Male***: Protibia straight, not modified. Proclaws simple, relatively long, equal. Median lobe of aedeagus with two lobes of dorsal sclerite rather narrow, subequal; in lateral left view, left dorsal lobe apically with longitudinal crest, its apex almost straight, pressed to right dorsal lobe; right dorsal lobe with weakly developed, inconspicuous median impression in right lateral view, more or less rounded apex and small crest on its left side where apex of left dorsal lobe placed in left lateral view. Lobes of ventral sclerite weakly sclerotised laterally, visible in left and right lateral views, mostly membranous, subequal, straight apically; sclerotised part of left ventral lobe long, thin, and straight apically in left lateral view. Paramere with setae not divided into distal and proximal; more distally situated setae slightly denser than more proximal ones (Fig. 51).

***Female***: Unknown.

##### Affinities.

Based on general shape of the median lobe, the species is very similar to *A.sandaunensis* sp. nov. but differs from it in elytral striation and shape of median lobe sclerites; for details, see under *A.sandaunensis* sp. nov.

##### Etymology.

The species is named after its type locality, Toricelli Mts. The name is a noun standing in apposition.

##### Distribution.

New Guinean endemic. Papua New Guinea: Sandaun Province, Toricelli Mts. The species is known only from the type locality (Fig. 83).

##### Habitat.

The species was collected in a puddle by the forest edge.

#### ﻿31. Austrelatus vagauensis sp. nov.

Taxon classificationAnimaliaColeopteraDytiscidae

27295C7E-374A-5683-A17A-0BEF75CEA180

https://zoobank.org/C62E8B7F-0B4F-4DA9-9DB9-595AD4CD6628

Figs 68 , 72 , 84

##### Type locality.

Papua New Guinea: Morobe Province, Herzog Mts, Vagau, ca. 1200 m a.s.l.

##### Type material.

***Holotype***: male “Stn. No. 144.”, “New Guinea: Morobe Dist., Herzog Mts., Vagau, C.4,000 ft. 4–17.i.1965”, “M.E. Bacchus. B.M. 1965-120” (BMNH).

***Paratypes***: PNG: Morobe: 3 males, 6 females with the same label as the holotype (BMNH, NHMW). 2 males, 2 females “June 23, 1988 Papua New Guinea Morobe, Lake Trist MP Kowalski”, “Loc. 7.9 S, 146.96 E Elev. 1710 m Pandanus swamp NW edge of Lake Trist” (CMPK, NHMW).

##### Description.

***Body size and form***: Beetle medium-sized to large, with oblong-oval habitus (Fig. 68).

***Measurements***: TL 6.1–7 mm, TL-H 5.5–6.25 mm, MW 2.9–3.35 mm, TL/MW 2–2.1; PL 0.85–1 mm, PW 2.5–2.9 mm, PL/PW 0.33–0.35; DBE 1–1.1 mm, DBE/PW 0.38–0.41.

Holotype: TL 6.4 mm, TL-H 5.85 mm, MW 3.1 mm, TL/MW 2.07; PL 0.9 mm, PW 2.7 mm, PL/PW 0.33; DBE 1.05 mm, DBE/PW 0.39.

***Colouration***: Dorsally piceous, with yellowish red head medially, pronotal sides and three small basal spots and one apical spot on elytron (Fig. 68).

Head yellowish red, dark brown behind eyes and reddish brown anteriorly, often also reddish brown medially, so that head has a broad, yellowish red V-like pattern or a reddish brown, median spot. Pronotum piceous on disc and paler towards sides, broadly yellowish red on them. Elytron piceous, with three rather distinct, small, yellowish red spots on elytral base: one between striae 1 and 3, second between striae 5 and 7 and third between striae 9 and 10; these spots never confluent; elytron with a distinct, narrow, yellow lateral band ending up with a slightly broader elongate apical spot. Scutellum yellowish red. Antennae, other head appendages, and legs proximally yellowish red; legs darker distally, yellowish red, especially metalegs. Venter piceous, with paler prosternum. Teneral beetles paler. The holotype is teneral specimen.

***Surface sculpture***: Elytron with 11 dorsal strongly impressed striae; submarginal stria present: 11+1 (Fig. 68).

Head with strioles between eyes, with rather dense punctation (spaces between punctures 1–3× times size of punctures); punctures relatively fine (diameter of punctures equal to diameter of microreticulation cells or smaller than it); head with a row of coarse setigerous punctures along inner margin of each eye and a short row at frontal angle of each eye; a slightly longer puncture row forms fronto-clypeal depression at each head side; microreticulation weak. Pronotum with numerous strioles; pronotal punctation finer and denser than on head; setigerous punctures form a row along pronotal margins, absent in posterior middle; disc of pronotum with indistinct longitudinal median scratch. Pronotum with fine microreticulation. Elytron with 11 strongly impressed dorsal striae; stria 5 shortly reduced basally; striae 1, 3, 5, 7, and 9 shortly reduced apically; submarginal striae present, long, well-developed, reaching ½ or more of elytron. Elytron with fine punctation and microreticulation. Ventral part with fine, inconspicuous punctation, invisible on metaventrite and metacoxae and weak on abdominal ventrites; prosternum smooth medially; metaventrite and metacoxae with weak microreticulation; on abdominal ventrites microreticulation almost invisible; metacoxal plates with numerous, strongly impressed longitudinal strioles, abdominal ventrites 1 and 2 with numerous, long, longitudinal strioles from margin to margin, on abdominal ventrites 3 and 4 strioles situated laterally and turn to middle, almost horizontal, abdominal ventrites 5 and 6 without strioles but with fine punctation that sparser medially and forms a dense, rugose lateral area at each side.

***Structures***: Head relatively broad. Pronotum short and broad; lateral margins distinctly convergent anteriorly. Base of prosternum rounded anteriorly, convex medially; blade of prosternal process rather small, short, rounded, convex in middle, with protruding apex.

***Male***: Protibia almost straight, not modified. Proclaws rather long, subequal in length; anterior more strongly curved than posterior one. Median lobe of aedeagus with two lobes of dorsal sclerite rather narrow; left dorsal lobe distinctly shorter that right one, with small apical crest; apex of left dorsal lobe curved downwards and to left, its very tip upwards; dorsally and laterally with denticulation (spinulae) visible in lateral left view; right dorsal lobe with distinct, but shallow, elongate median impression and an weak angular convexity above it; apex of right dorsal lobe large, swollen, rounded; left lobe of ventral sclerite with its sclerotised area long, thin, narrowly pointed apically, shorter than right ventral and dorsal lobes and 4/5 of length of left dorsal lobe. Paramere with setae not clearly divided into distal and proximal; more distally situated setae longer and slightly denser than more proximal ones; with several, short, thin the most proximal setae standing separately (Fig. 72).

***Female***: With more numerous pronotal strioles and elytron with denser punctation and additionally to it with very tiny strioles between striae. However, there are no strongly striolated, matt forms.

##### Variability.

There is a insignificant variation in the body size, colouration and dorsal striolation.

##### Affinities.

In body shape, elytral striation and dorsal colouration, the species is similar to *A.garainensis* sp. nov. and *A.kaszabi*. The species can be distinguished from them by shape of its median lobe sclerites, especially by the weaker sclerotised area of the left ventral lobe, with its apex not curved like a hook but straight. See also under *A.sarmiensis* sp. nov.

##### Etymology.

The species is named after Vagau Village. The name is an adjective in the nominative singular.

##### Distribution.

New Guinean endemic. Papua New Guinea: Morobe Province (Fig. 84).

##### Habitat.

Lentic. Northwest of Lake Trist, the species was collected in a Pandanus swamp, where it was obtained from smaller puddles and pools rich in rotten debris.

#### ﻿32. Austrelatus wanggarensis sp. nov.

Taxon classificationAnimaliaColeopteraDytiscidae

262BEF1B-4186-5E34-93E1-58D0417E814D

https://zoobank.org/7EEF6D34-CB97-4AB5-84B3-C647F5473FA5

Figs 67 , 71 , 84 , 88

##### Type locality.

Indonesia: Papua Province: Nabire Regency, Wanggar – Kali Bumi.

##### Type material.

***Holotype***: male “W.-Neuguinea /Paniai Prov./ Wanggar- Kali Bumi / IR 14 30.9 & 1.10.90 leg: Balke & Hendrich” (ZSM).

##### Description.

***Body size and form***: Beetle small, with oblong-oval habitus (Fig. 1).

***Measurements***: Holotype: TL 4.9 mm, TL-H 4.45 mm, MW 2.3 mm, TL/MW 2.13; PL 0.65 mm, PW 2 mm, PL/PW 0.33; DBE 0.85 mm, DBE/PW 0.43.

***Colouration***: Dorsally piceous, with yellowish red head, pronotal sides, and basal band and one apical spot on elytron (Fig. 67).

Head yellowish red, darker narrowly behind eyes. Pronotum dark brown on disc and paler towards sides, yellowish red on them. Elytron piceous, with a yellowish red, rather straight basal band not reaching suture and lateral margin and with a distinct elongate yellow apical spot. Scutellum piceous. Antennae, other head appendages, and pro- and mesolegs proximally yellowish red, metalegs reddish brown, legs darker distally. Venter reddish brown.

***Surface sculpture***: Elytron with 11 complete dorsal striae; submarginal stria present: 11+1 (Fig. 67).

Head without strioles, with rather dense punctation (spaces between punctures 1–3× size of punctures); punctures relatively fine (diameter of punctures usually equal to diameter of microreticulation cells); head with a row of coarse setigerous punctures along inner margin of each eye and a short row at frontal angle of each eye; a slightly longer puncture row forms fronto-clypeal depression at each head side; microreticulation distinct. Pronotum usually with only few indistinct strioles in posterolateral angles; with fine longitudinal wrinkles at posterior margin; pronotal punctation finer than on head; setigerous punctures form a row along pronotal margins, absent in posterior middle; disc of pronotum with indistinct longitudinal median scratch. Pronotum with fine microreticulation. Elytron with 11 dorsal striae; striae 2–4 and 6 complete, striae 1 and 5 usually shortly reduced basally; submarginal striae present. Elytron with fine punctation and microreticulation. Ventral part with fine, inconspicuous punctation, invisible on metaventrite and metacoxae and weak on abdominal ventrites; prosternum smooth medially; metaventrite and metacoxae with weak microreticulation; on abdominal ventrites microreticulation almost invisible; metacoxal plates with numerous, distinctly impressed longitudinal strioles, abdominal ventrites 1 and 2 with numerous, long, longitudinal strioles from margin to margin, on abdominal ventrites 3 and 4 strioles situated laterally and turn to middle, almost horizontal, abdominal ventrites 5 and 6 without strioles but with fine punctation that sparser medially and forms a dense, rugose lateral area at each side.

***Structures***: Head broad. Pronotum short and broad; lateral margins distinctly convergent anteriorly. Base of prosternum narrowly rounded anteriorly, convex medially; blade of prosternal process rather short and narrow, convex in middle.

***Male***: Protibia straight, not modified. Proclaws simple, rather long, subequal in length. Median lobe of aedeagus with two lobes of dorsal sclerite rather narrow; left dorsal lobe shorter that right one, with weak, long lateral crest and apex curved downwards, not to left, dorsally with distinct denticulation (spinulae) invisible in lateral left view due to weak curvation to left; right dorsal lobe with small, indistinct, elongate median impression and modified apex: relatively large, swollen, more or less rounded; left lobe of ventral sclerite with its sclerotised area long, rounded apically, shorter than right ventral and dorsal lobes and more than 2/3 of length of left dorsal lobe. Paramere with setae not divided into distal and proximal; more distally situated setae denser than more proximal ones (Fig. 71).

***Female***: Unknown.

##### Affinities.

In general shape of median lobe, especially in shape of the sclerotised area of left ventral lobe and in shape of the lateral crest of the left dorsal lobe, the species is similar to *A.fakfak* sp. nov., *A.testegensis* sp. nov., and *A.manokwariensis* sp. nov. The species can be distinguished from them by 11+1 elytral striae and shape of the median lobe sclerites.

##### Etymology.

The species is named after Wanggar. The name is an adjective in the nominative singular.

##### Distribution.

New Guinean endemic. Indonesia: Papua Province: Nabire Regency. The species is known only from its type locality (Fig. 84).

##### Habitat.

The species was collected in a shallow (20 cm water depth), partly shaded, larger rest pool of an intermittent stream. The pool was rich in rotten leaves and twigs (Fig. 88).

**Figures 22–25.**
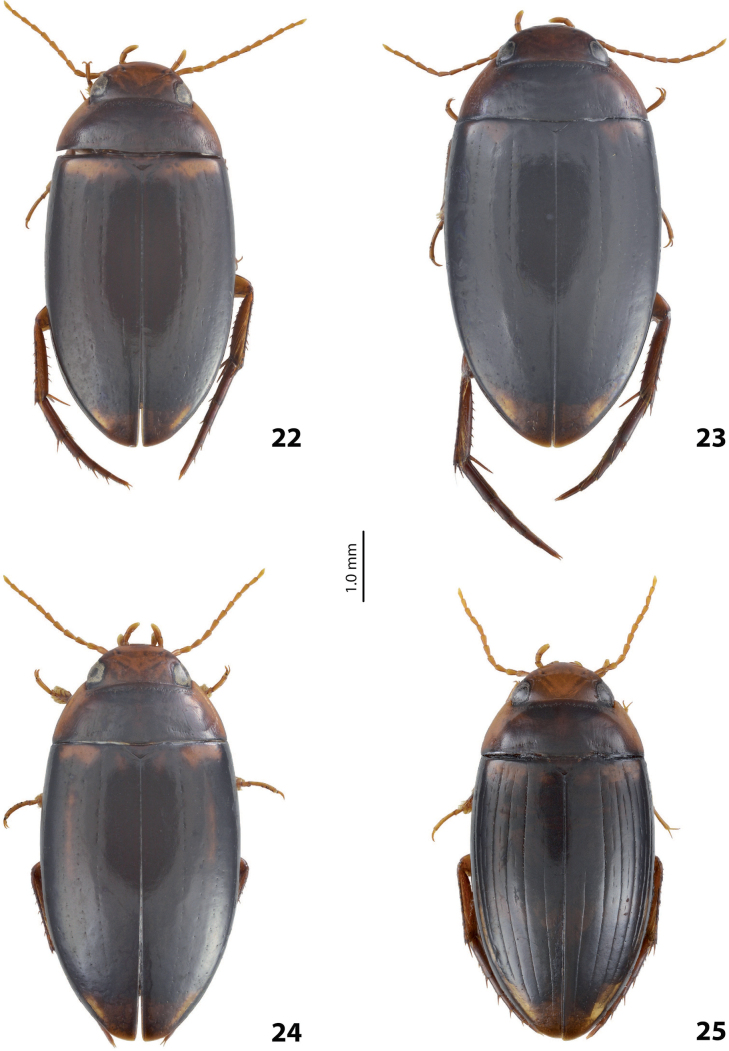
Habitus and colouration of **22***Austrelatusfebrisauri* sp. nov. **23***A.oksibilensis* sp. nov. **24***A.pseudooksibilensis* sp. nov., without elytral striae **25***A.pseudooksibilensis* sp. nov., with elytral striae.

**Figure 26.**
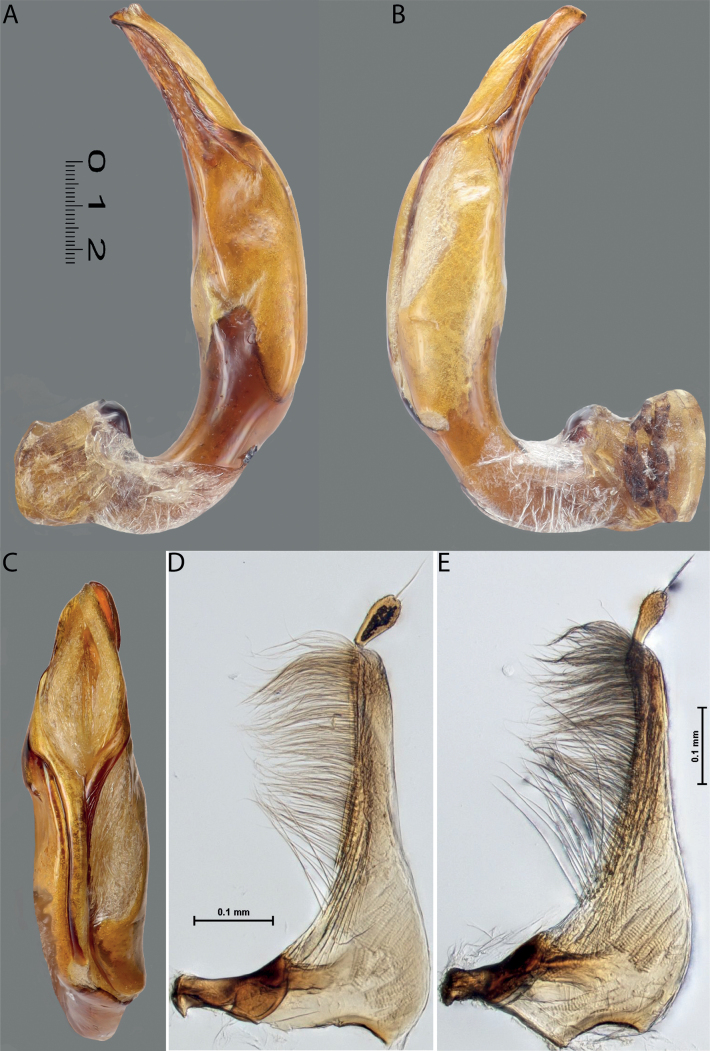
*Austrelatusfebrisauri* sp. nov., median lobe **A** left lateral view **B** right lateral view **C** ventral view **D** left paramere in external view **E** right paramere in internal view. Scale bar: one unit – 0.1 mm (**A–C**).

**Figure 27.**
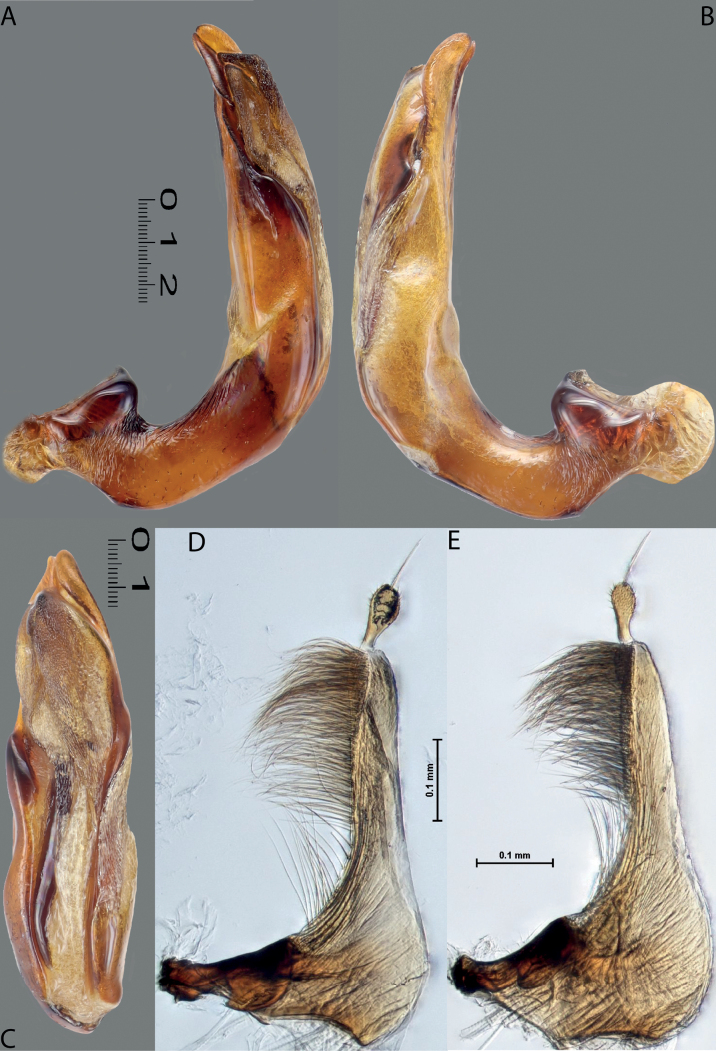
*Austrelatusoksibilensis* sp. nov., median lobe **A** left lateral view **B** right lateral view **C** ventral view **D** left paramere in external view **E** right paramere in internal view. Scale bar: one unit – 0.1 mm (**A–C**).

**Figure 28.**
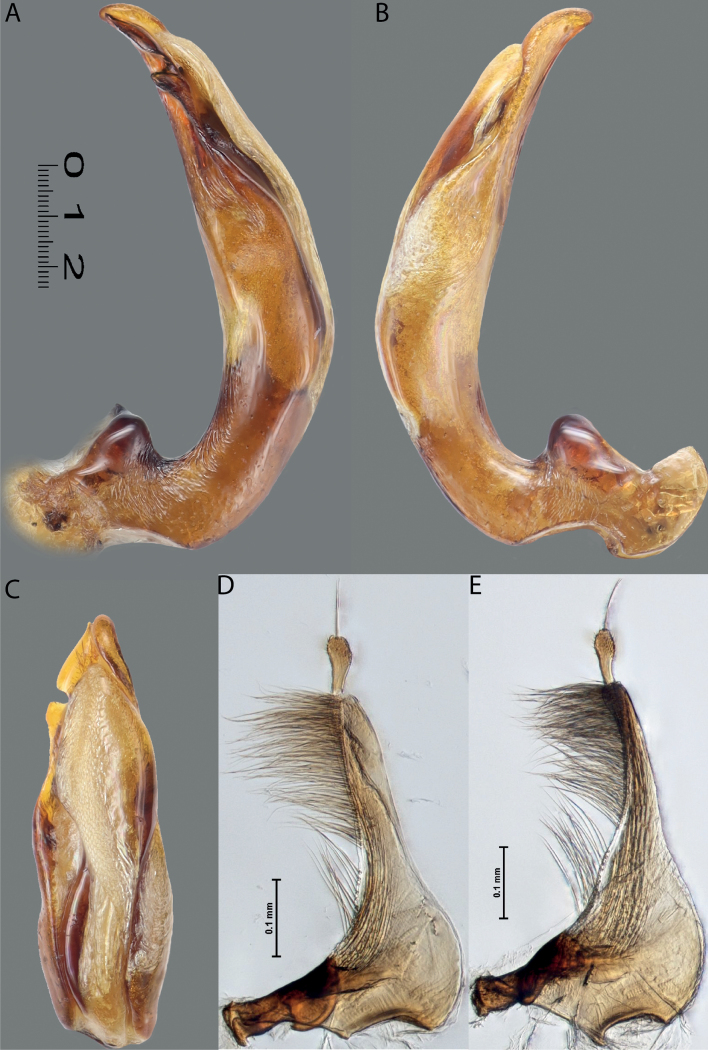
*Austrelatuspseudooksibilensis* sp. nov., median lobe **A** left lateral view **B** right lateral view **C** ventral view **D** left paramere in external view **E** right paramere in internal view. Scale bar: one unit – 0.1 mm (**A–C**).

**Figures 29–32.**
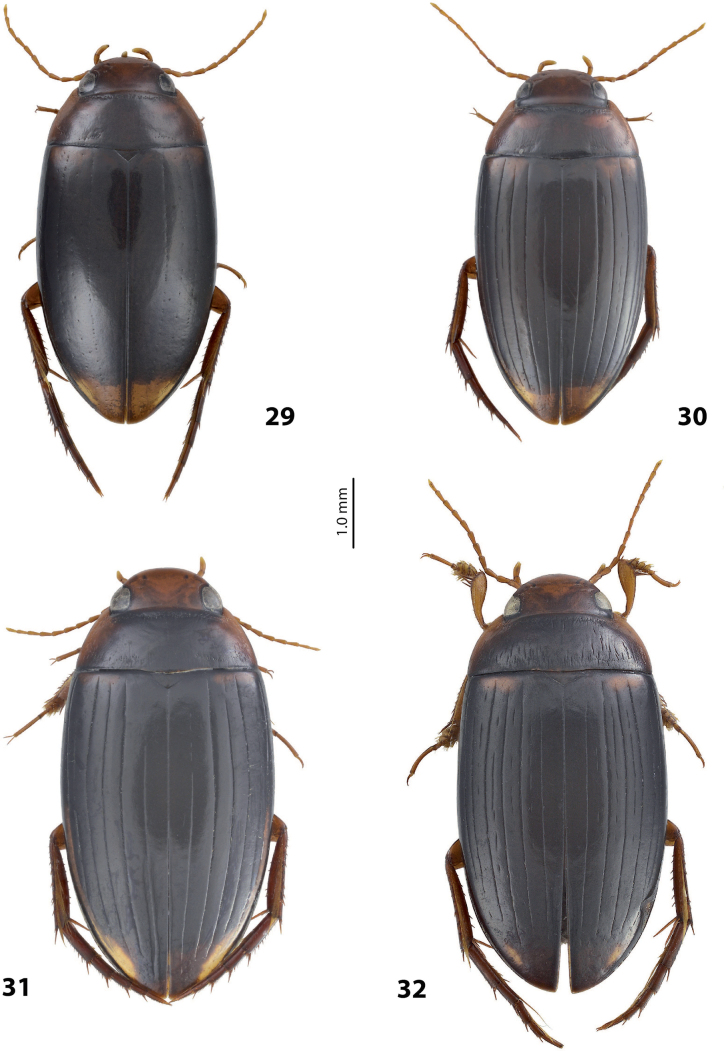
Habitus and colouration of **29***Austrelatusbrazza* sp. nov., without elytral striae **30***A.brazza* sp. nov., with elytral striae **31***A.lisae* sp. nov. **32***A.bormensis* sp. nov.

**Figure 33.**
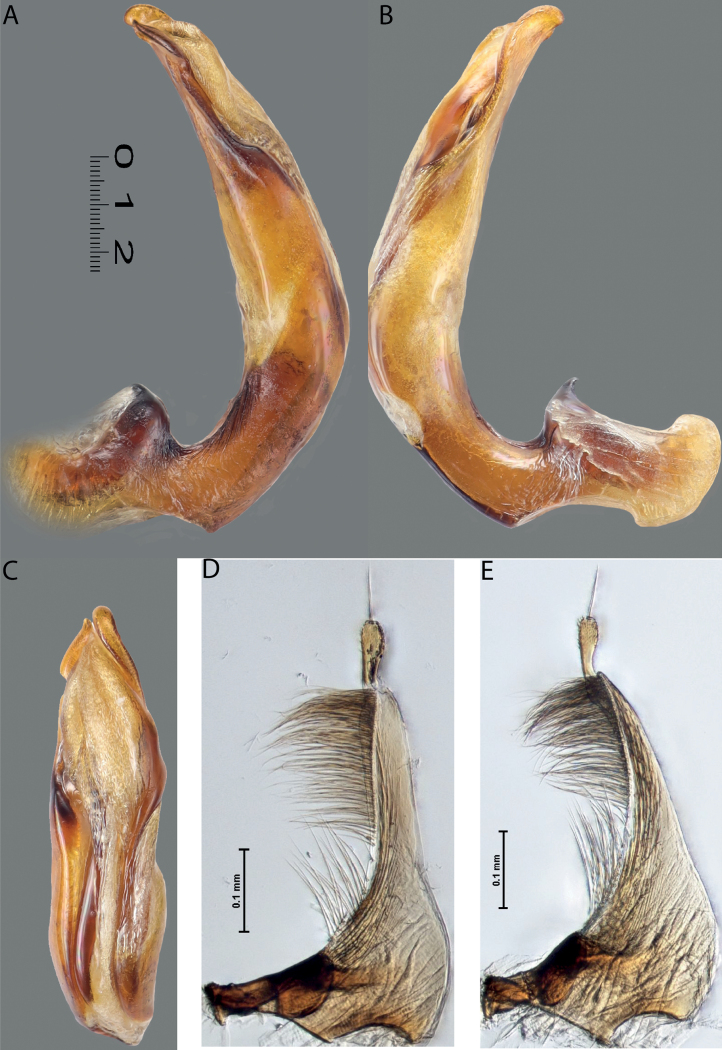
*Austrelatusbrazza* sp. nov., median lobe **A** left lateral view **B** right lateral view **C** ventral view **D** left paramere in external view **E** right paramere in internal view. Scale bar: one unit – 0.1 mm (**A–C**).

**Figure 34.**
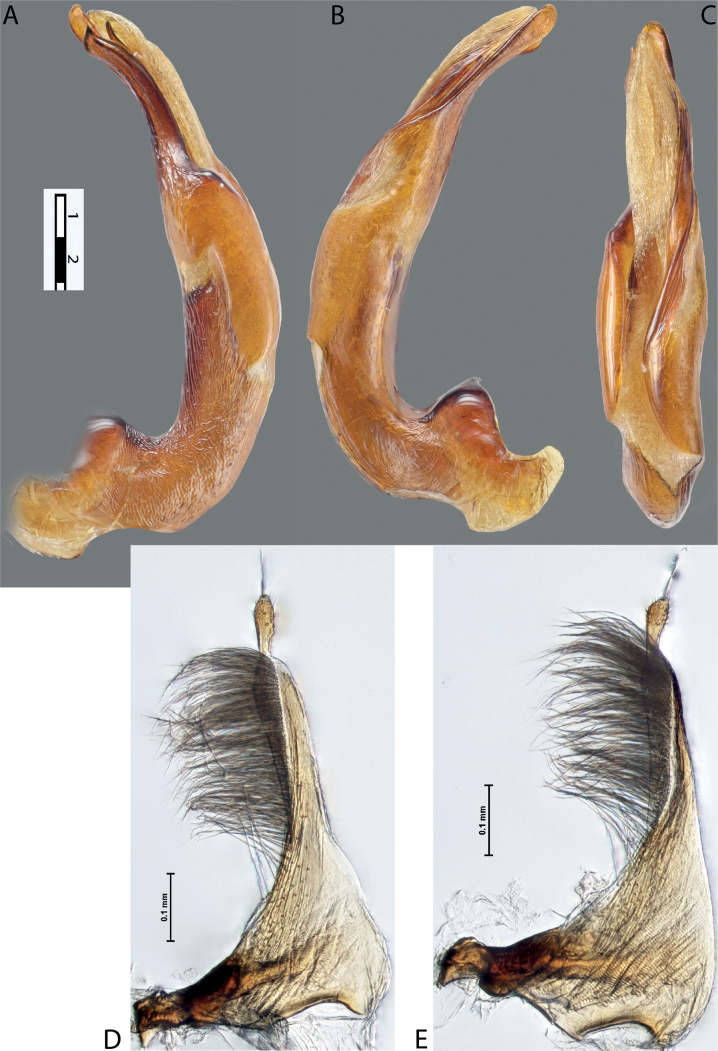
*Austrelatuslisae* sp. nov., median lobe **A** left lateral view **B** right lateral view **C** ventral view **D** left paramere in external view **E** right paramere in internal view. Scale bar: one unit – 0.1 mm (**A–C**).

**Figure 35.**
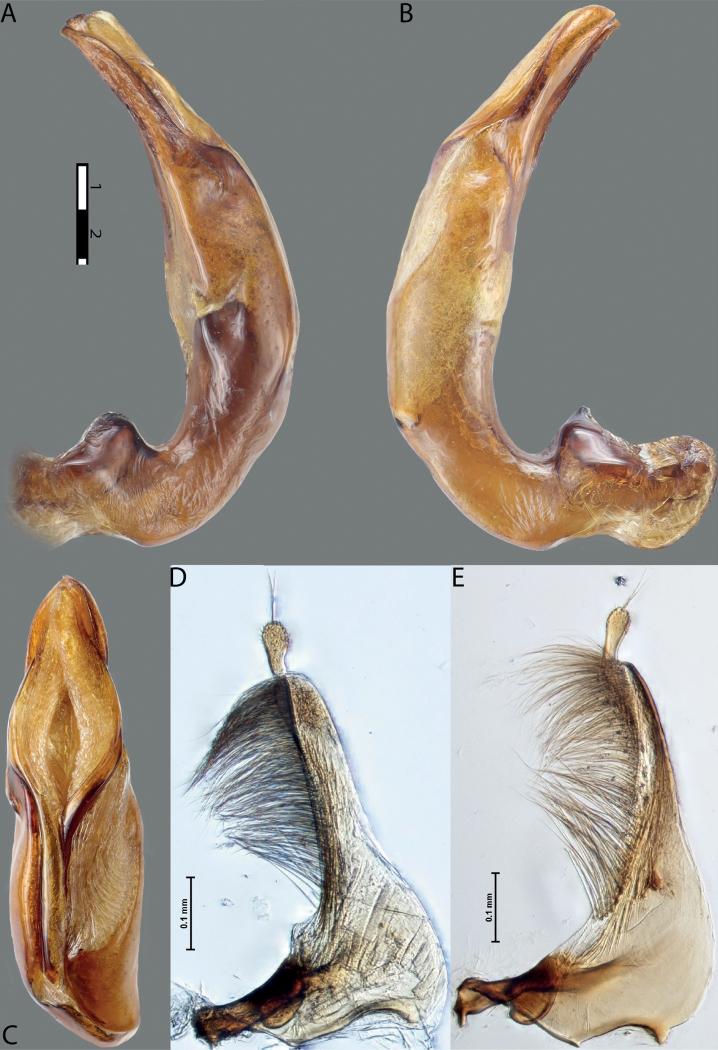
*Austrelatusbormensis* sp. nov., median lobe **A** left lateral view **B** right lateral view **C** ventral view **D** left paramere in external view **E** right paramere in internal view. Scale bar: one unit – 0.1 mm (**A–C**).

**Figures 36–39.**
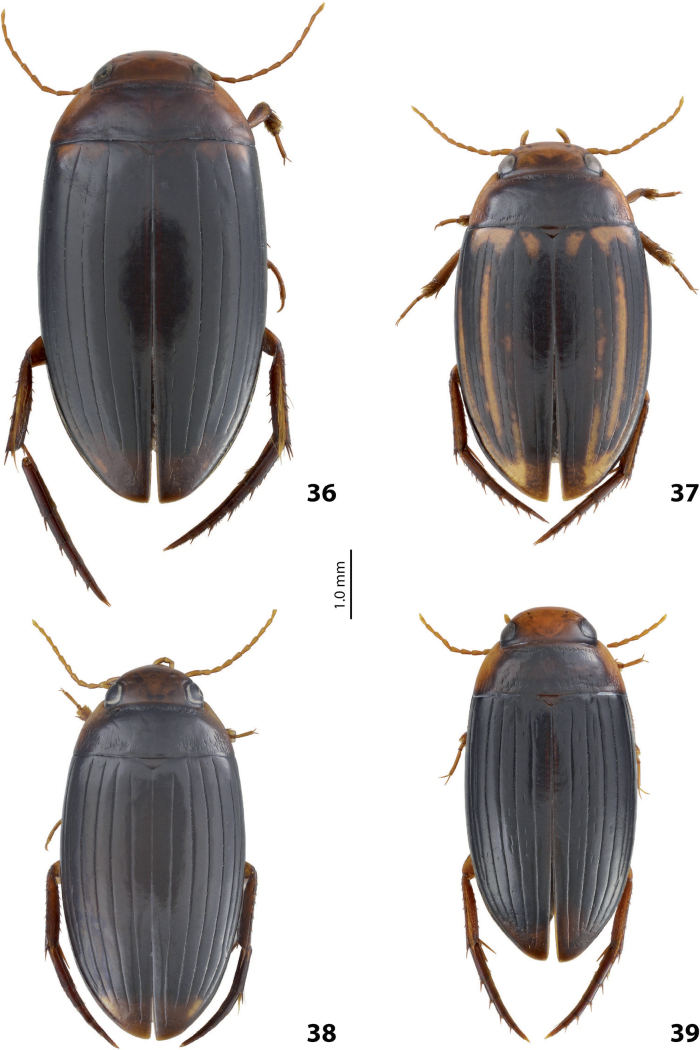
Habitus and colouration of **36***Austrelatusneoguineensis* (Zimmermann, 1919), lectotype **37***A.mirificus* sp. nov. **38***A.securiformis* sp. nov. **39***A.testegensis* sp. nov.

**Figure 40.**
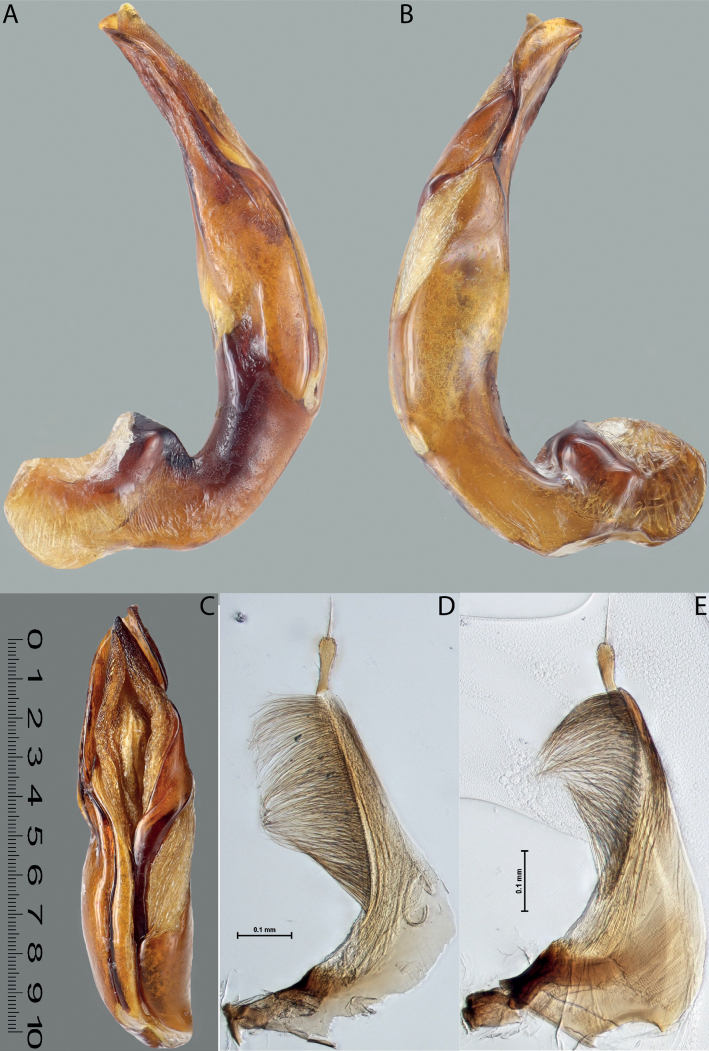
*Austrelatusneoguineensis* (Zimmermann, 1919), median lobe **A** left lateral view **B** right lateral view **C** ventral view **D** left paramere in external view **E** right paramere in internal view. Scale bar: one unit – 0.1 mm (**A–C**).

**Figure 41.**
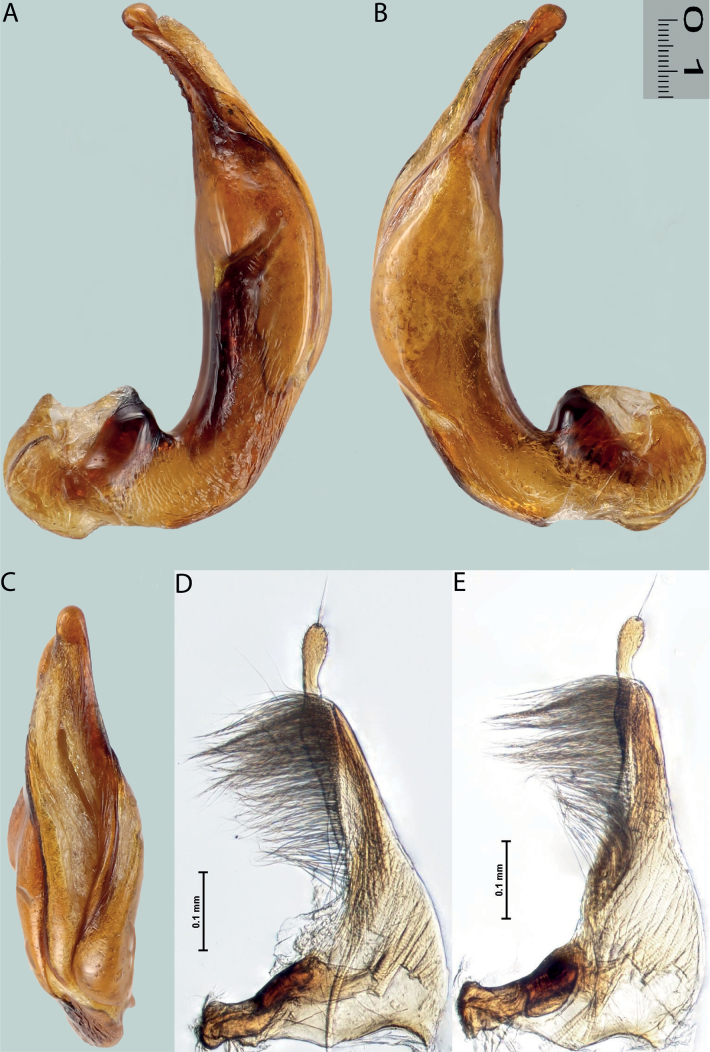
*Austrelatusmirificus* sp. nov., median lobe **A** left lateral view **B** right lateral view **C** ventral view **D** left paramere in external view **E** right paramere in internal view. Scale bar: one unit – 0.1 mm (**A–C**).

**Figure 42.**
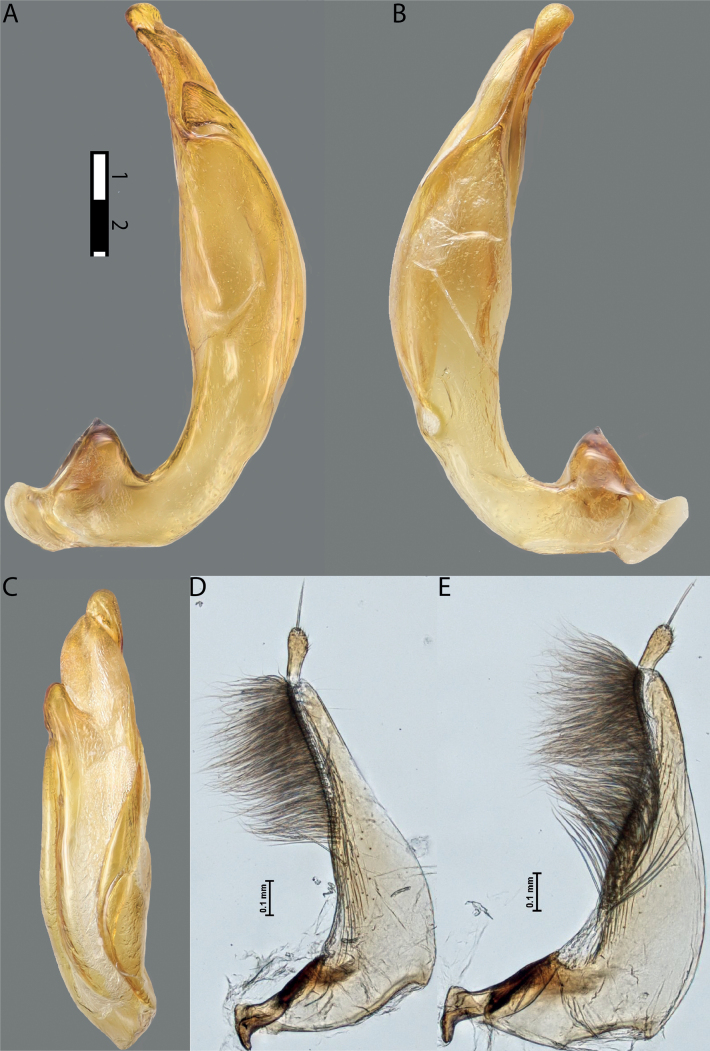
*Austrelatussecuriformis* sp. nov., median lobe **A** left lateral view **B** right lateral view **C** ventral view **D** left paramere in external view **E** right paramere in internal view. Scale bar: one unit – 0.1 mm (**A–C**).

**Figure 43.**
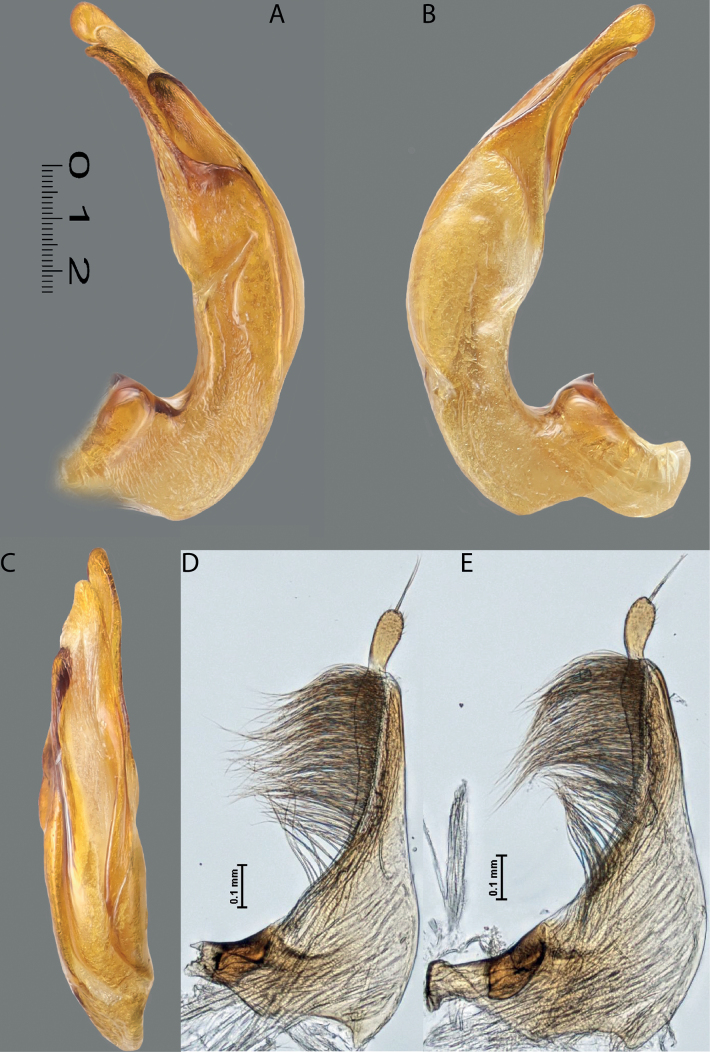
*Austrelatustestegensis* sp. nov., median lobe **A** left lateral view **B** right lateral view **C** ventral view **D** left paramere in external view **E** right paramere in internal view. Scale bar: one unit – 0.1 mm (**A–C**).

**Figures 44–47.**
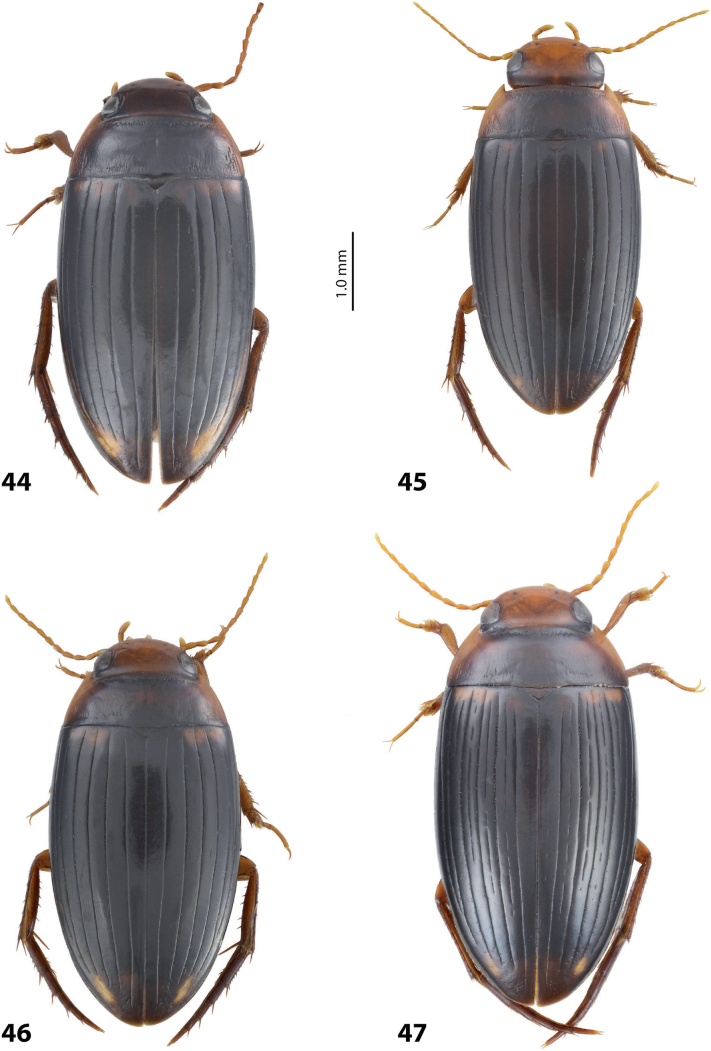
Habitus and colouration of **44***Austrelatusfojaensis* sp. nov. **45***A.fakfak* sp. nov. **46***A.manokwariensis* sp. nov. **47***A.toricelli* sp. nov.

**Figure 48.**
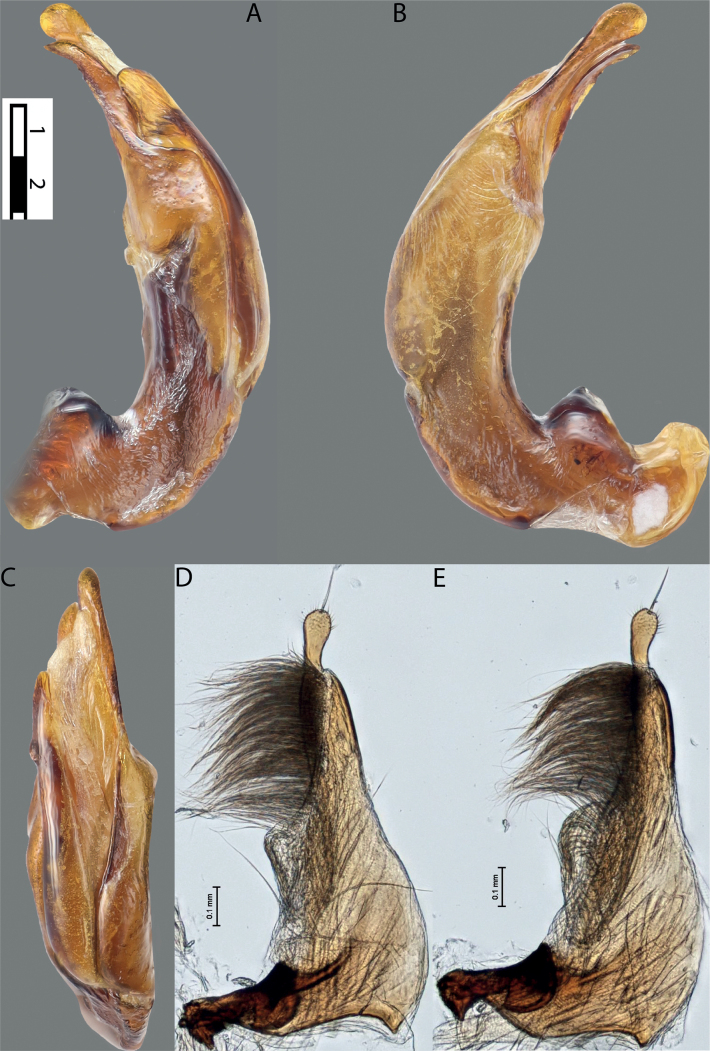
*Austrelatusfojaensis* sp. nov., median lobe **A** left lateral view **B** right lateral view **C** ventral view **D** left paramere in external view **E** right paramere in internal view. Scale bar: one unit – 0.1 mm (**A–C**).

**Figure 49.**
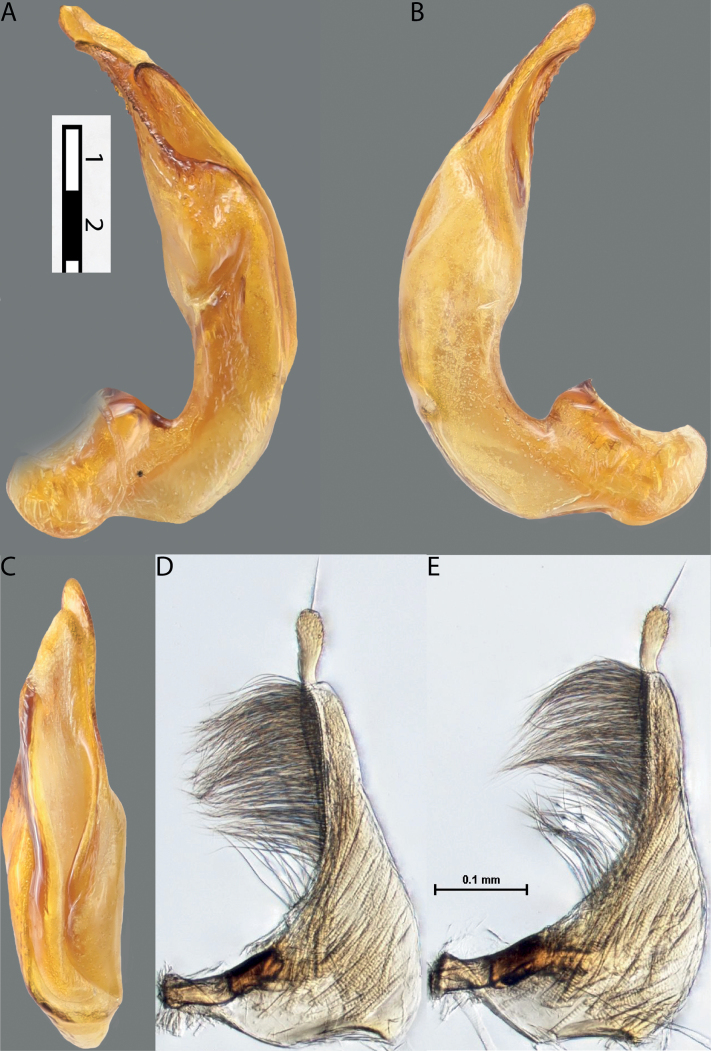
*Austrelatusfakfak* sp. nov., median lobe **A** left lateral view **B** right lateral view **C** ventral view **D** left paramere in external view **E** right paramere in internal view. Scale bar: one unit – 0.1 mm (**A–C**).

**Figure 50.**
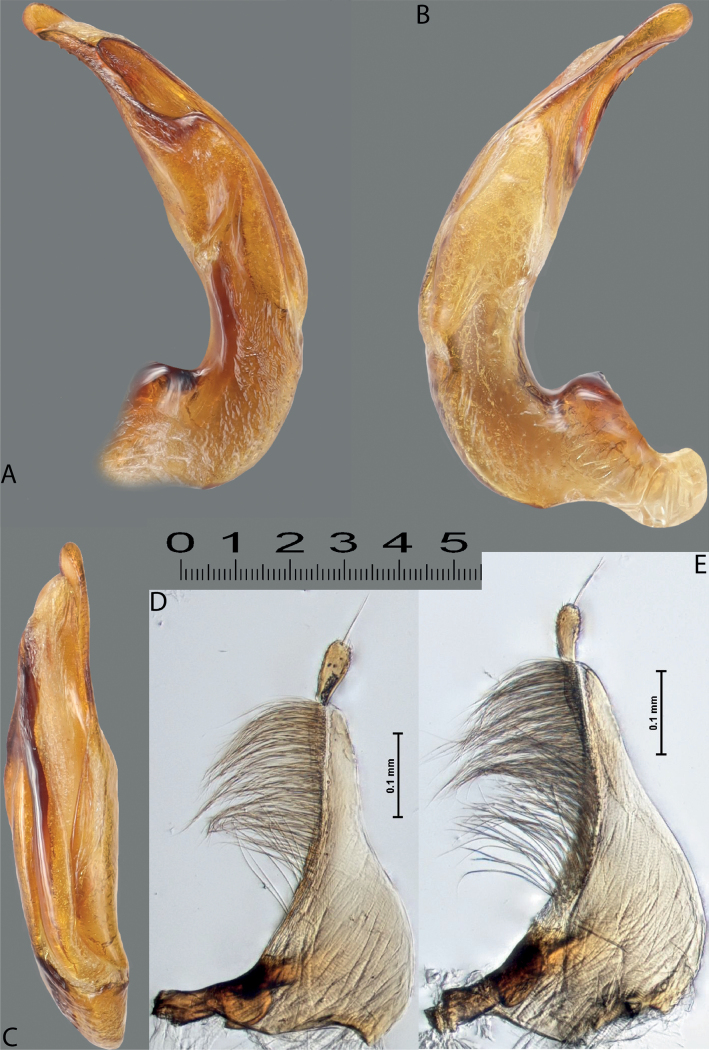
*Austrelatusmanokwariensis* sp. nov., median lobe **A** left lateral view **B** right lateral view **C** ventral view **D** left paramere in external view **E** right paramere in internal view. Scale bar: one unit – 0.1 mm (**A–C**).

**Figure 51.**
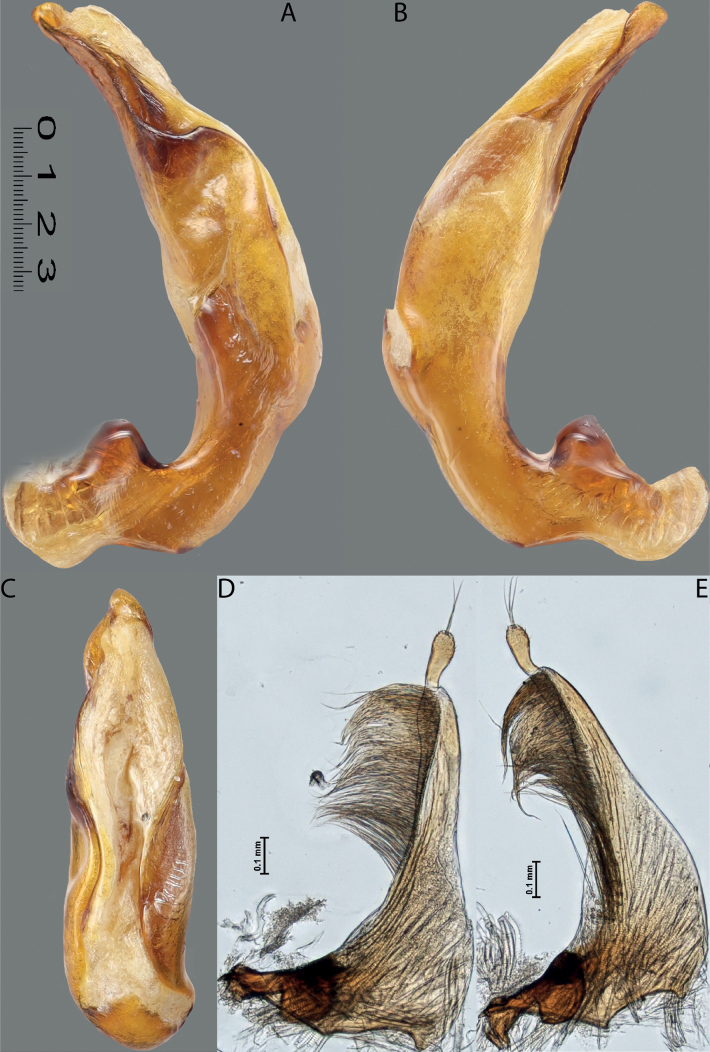
*Austrelatustoricelli* sp. nov., median lobe **A** left lateral view **B** right lateral view **C** ventral view **D** left paramere in external view **E** right paramere in internal view. Scale bar: one unit – 0.1 mm (**A–C**).

**Figures 52–55.**
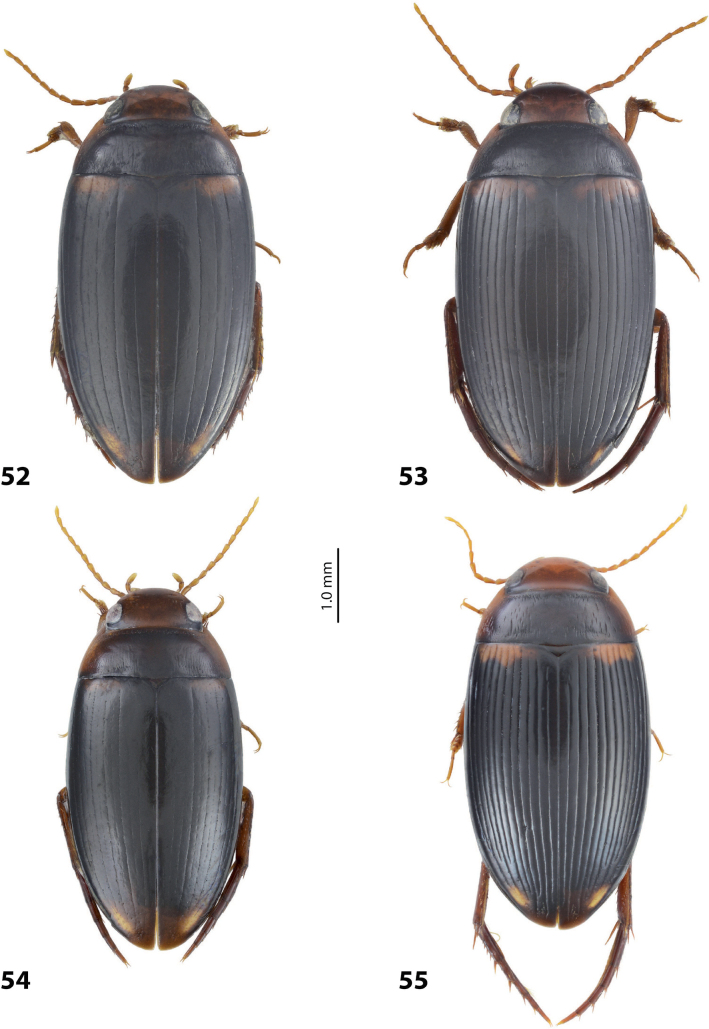
Habitus and colouration of **52***Austrelatusinnominatus* sp. nov., with reduced elytral striae **53***A.innominatus* sp. nov., with complete elytral striae **54***A.rajaampatensis* sp. nov., with reduced elytral striae **55***A.rajaampatensis* sp. nov., with complete elytral striae.

**Figure 56.**
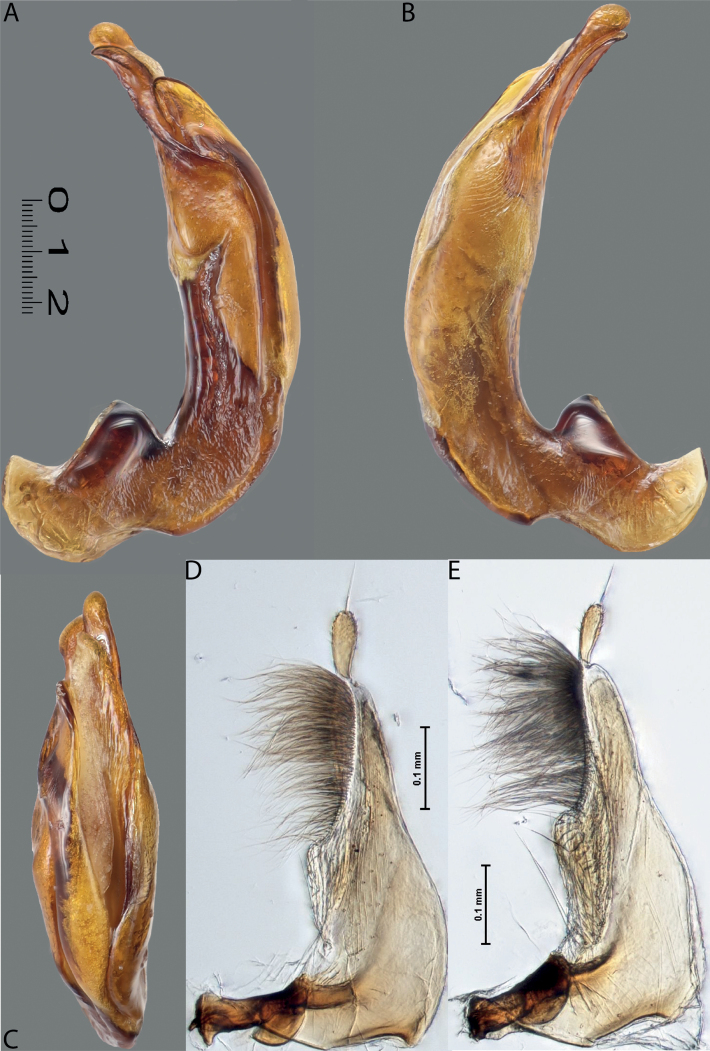
*Austrelatusinnominatus* sp. nov., median lobe **A** left lateral view **B** right lateral view **C** ventral view **D** left paramere in external view **E** right paramere in internal view. Scale bar: one unit – 0.1 mm (**A–C**).

**Figure 57.**
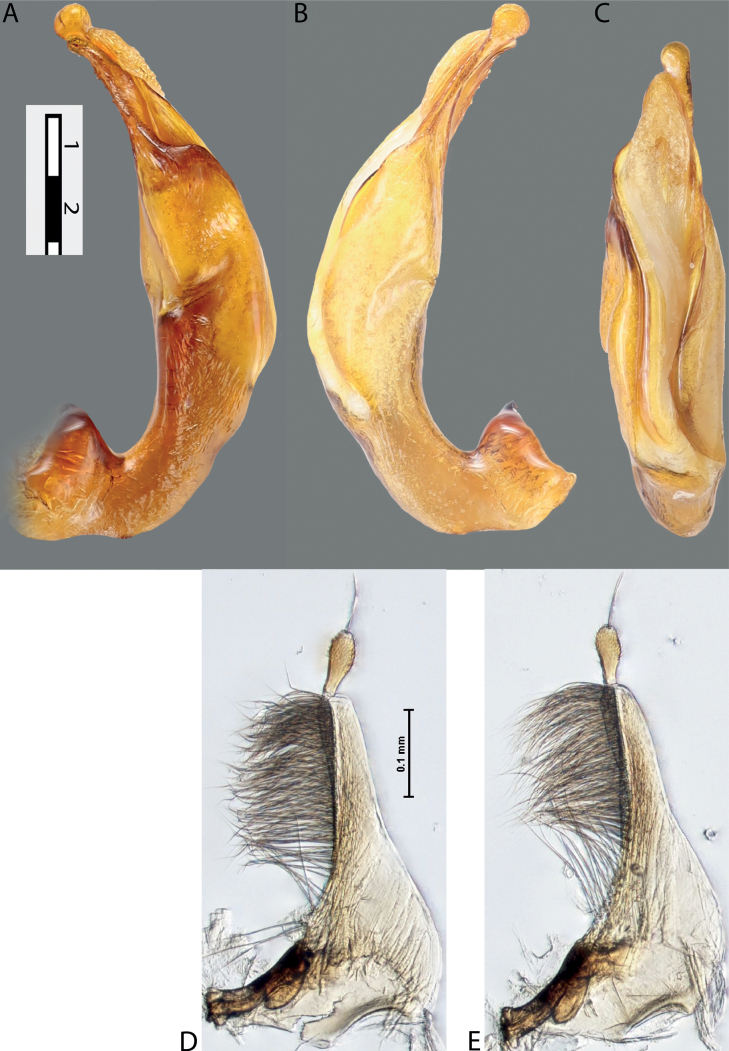
*Austrelatusrajaampatensis* sp. nov., median lobe **A** left lateral view **B** right lateral view **C** ventral view **D** left paramere in external view **E** right paramere in internal view. Scale bar: one unit – 0.1 mm (**A–C**).

**Figures 58–61.**
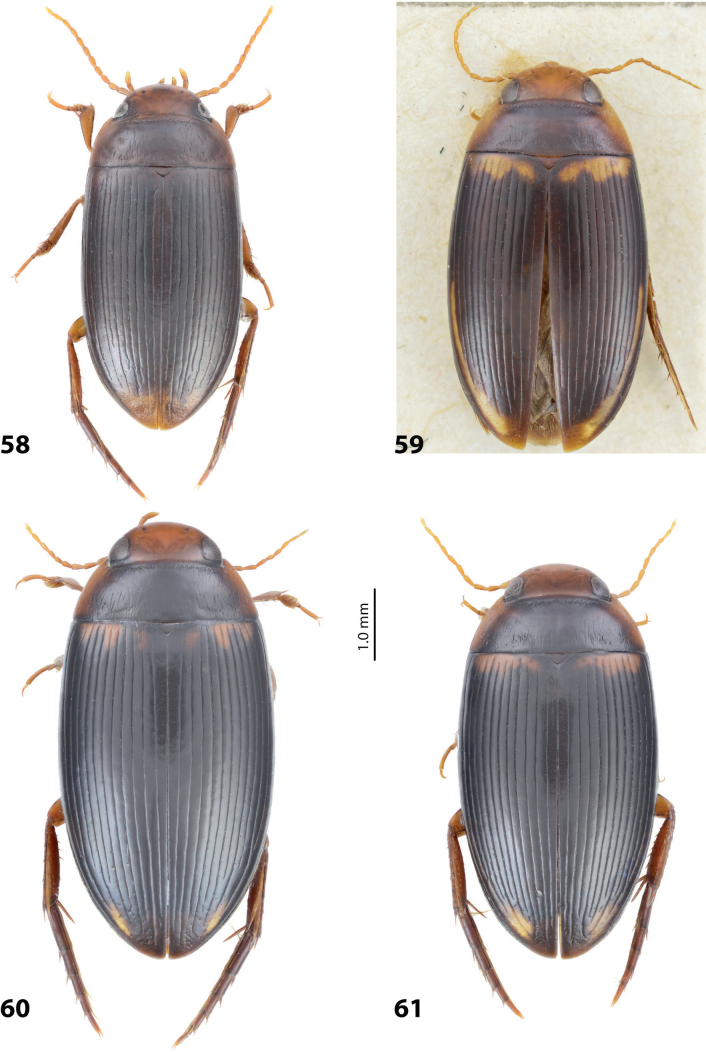
Habitus and colouration of **58***Austrelatusbaliem* sp. nov. **59***A.kaszabi* (Guignot, 1956), holotype **60***A.garainensis* sp. nov. **61***A.mimika* sp. nov.

**Figure 62.**
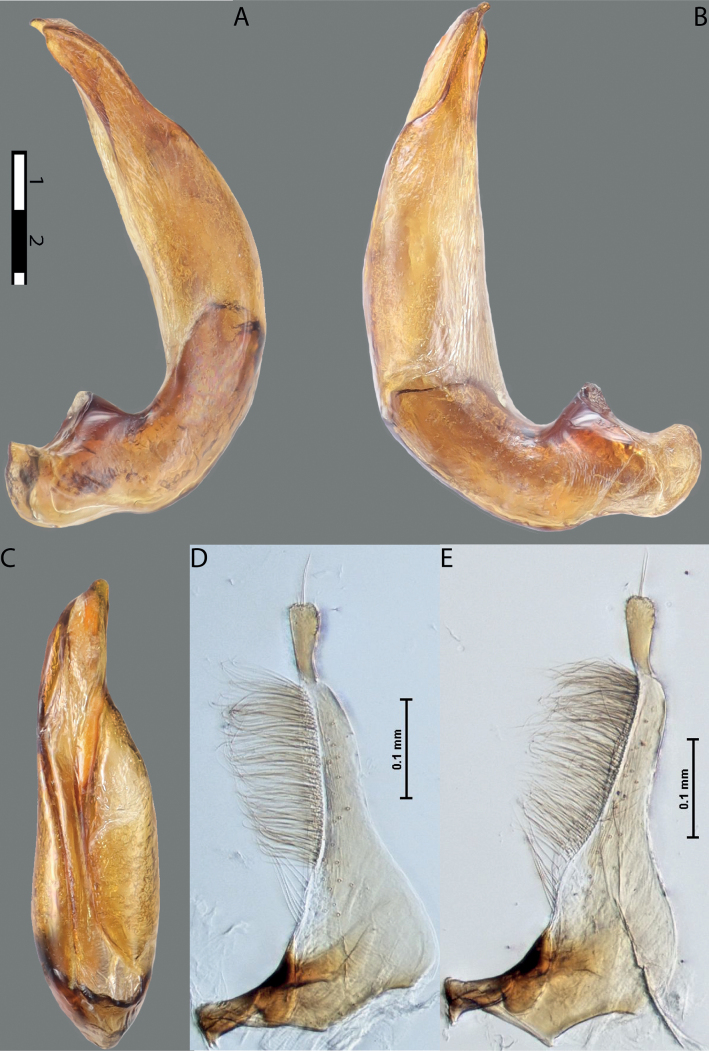
*Austrelatusbaliem* sp. nov., median lobe **A** left lateral view **B** right lateral view **C** ventral view **D** left paramere in external view **E** right paramere in internal view. Scale bar: one unit – 0.1 mm (**A–C**).

**Figure 63.**
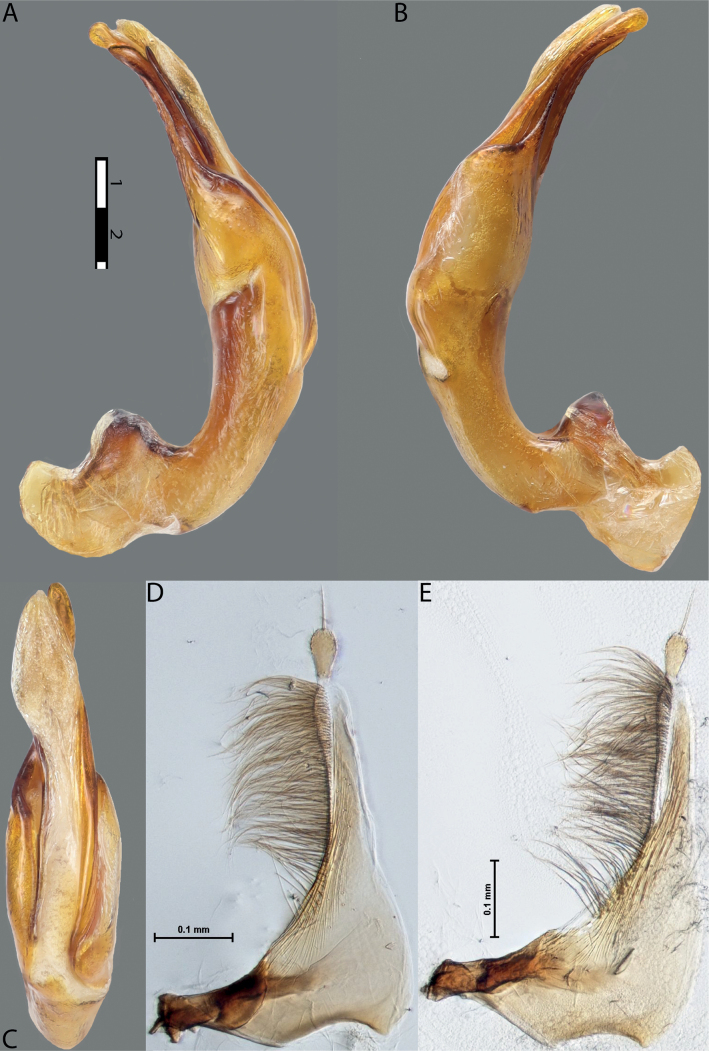
*Austrelatuskaszabi* (Guignot, 1956), median lobe **A** left lateral view **B** right lateral view **C** ventral view **D** left paramere in external view **E** right paramere in internal view. Scale bar: one unit – 0.1 mm (**A–C**).

**Figure 64.**
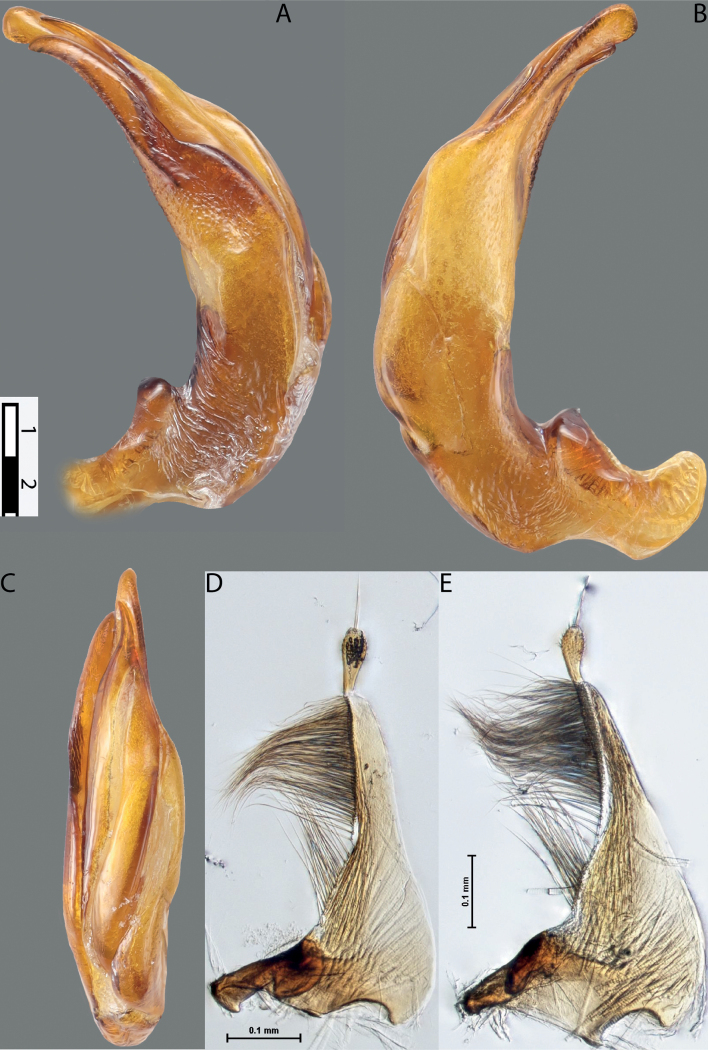
*Austrelatusgarainensis* sp. nov., median lobe **A** left lateral view **B** right lateral view **C** ventral view **D** left paramere in external view **E** right paramere in internal view. Scale bar: one unit – 0.1 mm (**A–C**).

**Figure 65.**
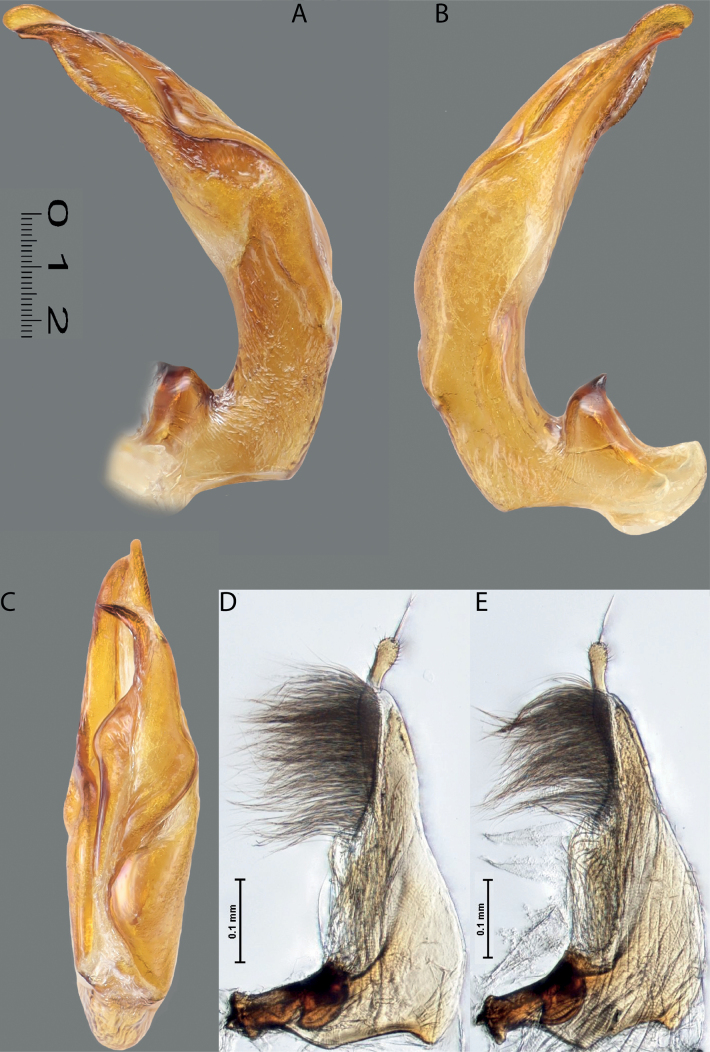
*Austrelatusmimika* sp. nov., median lobe **A** left lateral view **B** right lateral view **C** ventral view **D** left paramere in external view **E** right paramere in internal view. Scale bar: one unit – 0.1 mm (**A–C**).

**Figures 66–69.**
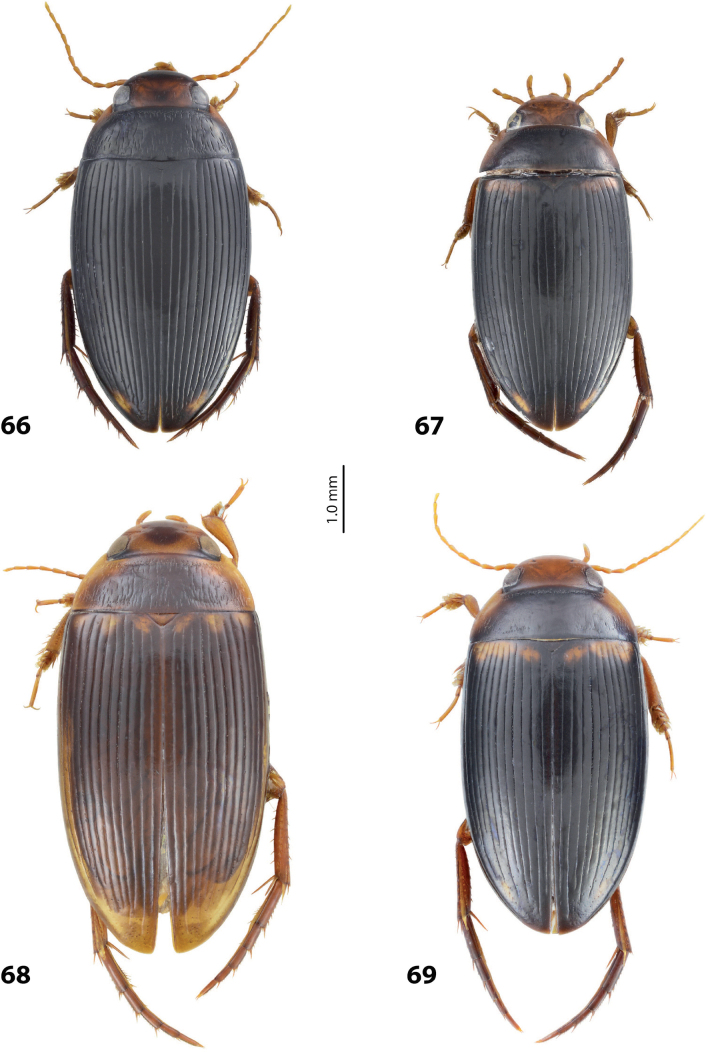
Habitus and colouration of **66***Austrelatusrouaffer* sp. nov. **67***A.wanggarensis* sp. nov. **68***A.vagauensis* sp. nov. **69***A.sandaunensis* sp. nov.

**Figure 70.**
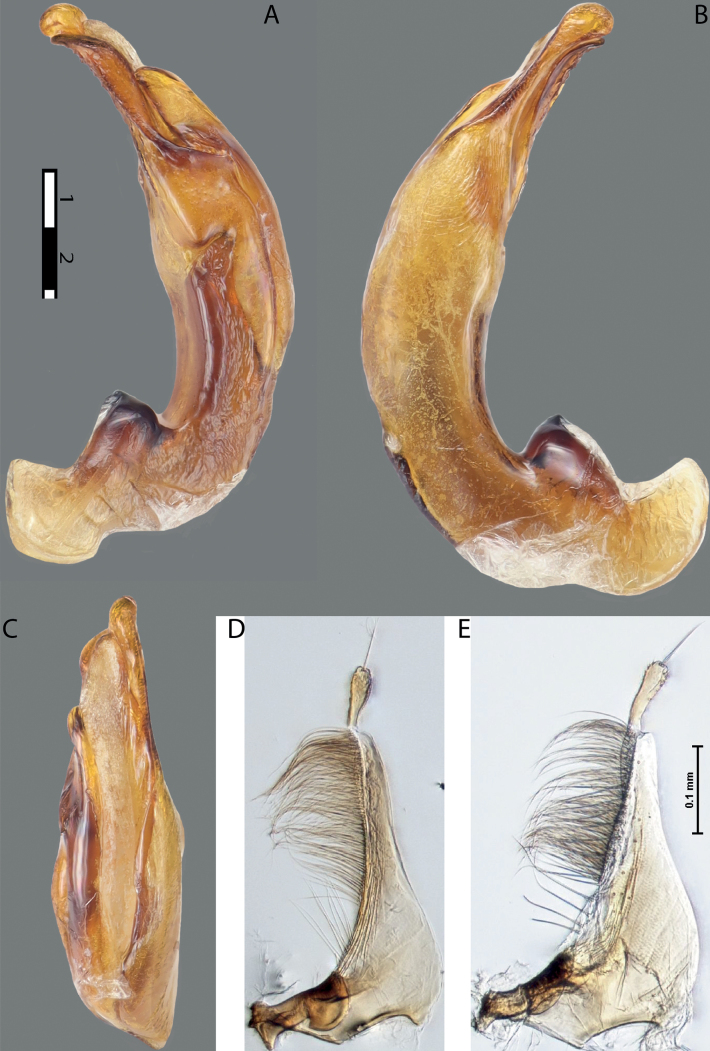
*Austrelatusrouaffer* sp. nov., median lobe **A** left lateral view **B** right lateral view **C** ventral view **D** left paramere in external view **E** right paramere in internal view. Scale bar: one unit – 0.1 mm (**A–C**).

**Figure 71.**
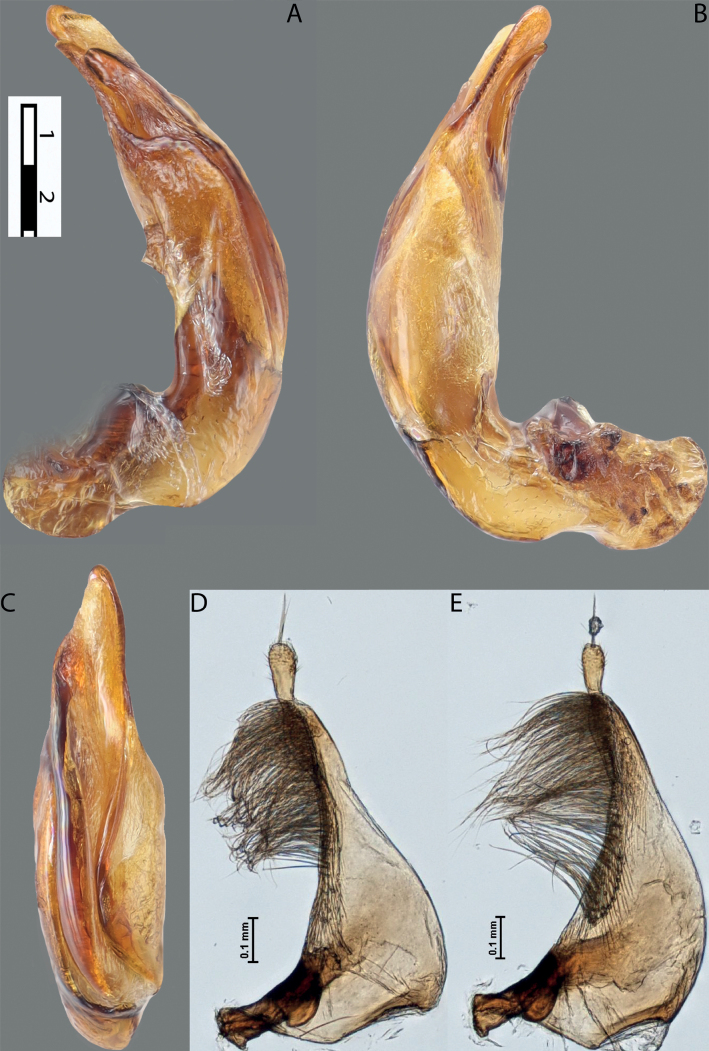
*Austrelatuswanggarensis* sp. nov., median lobe **A** left lateral view **B** right lateral view **C** ventral view **D** left paramere in external view **E** right paramere in internal view. Scale bar: one unit – 0.1 mm (**A–C**).

**Figure 72.**
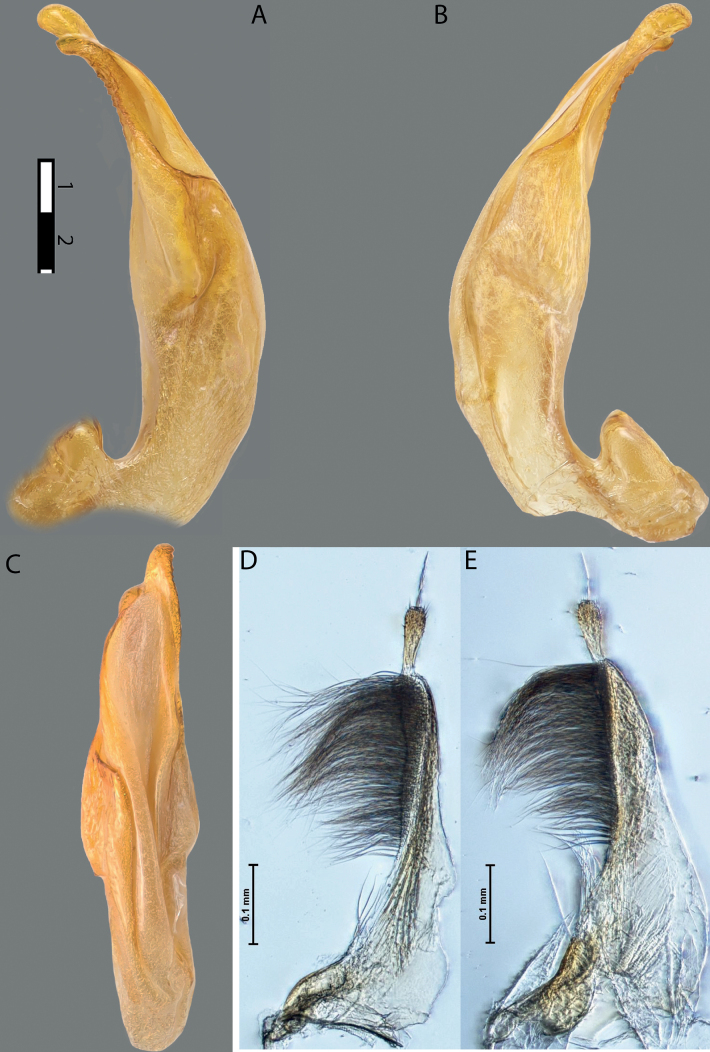
*Austrelatusvagauensis* sp. nov., median lobe **A** left lateral view **B** right lateral view **C** ventral view **D** left paramere in external view **E** right paramere in internal view. Scale bar: one unit – 0.1 mm (**A–C**).

**Figure 73.**
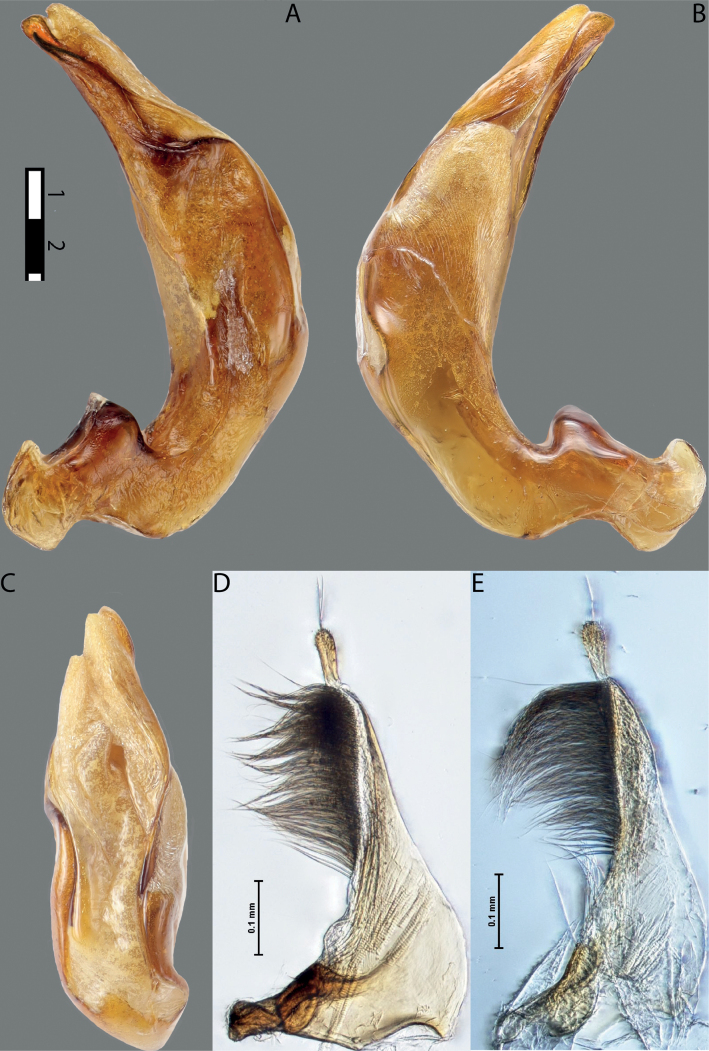
*Austrelatussandaunensis* sp. nov., median lobe **A** left lateral view **B** right lateral view **C** ventral view **D** left paramere in external view **E** right paramere in internal view. Scale bar: one unit – 0.1 mm (**A–C**).

**Figures 74–77.**
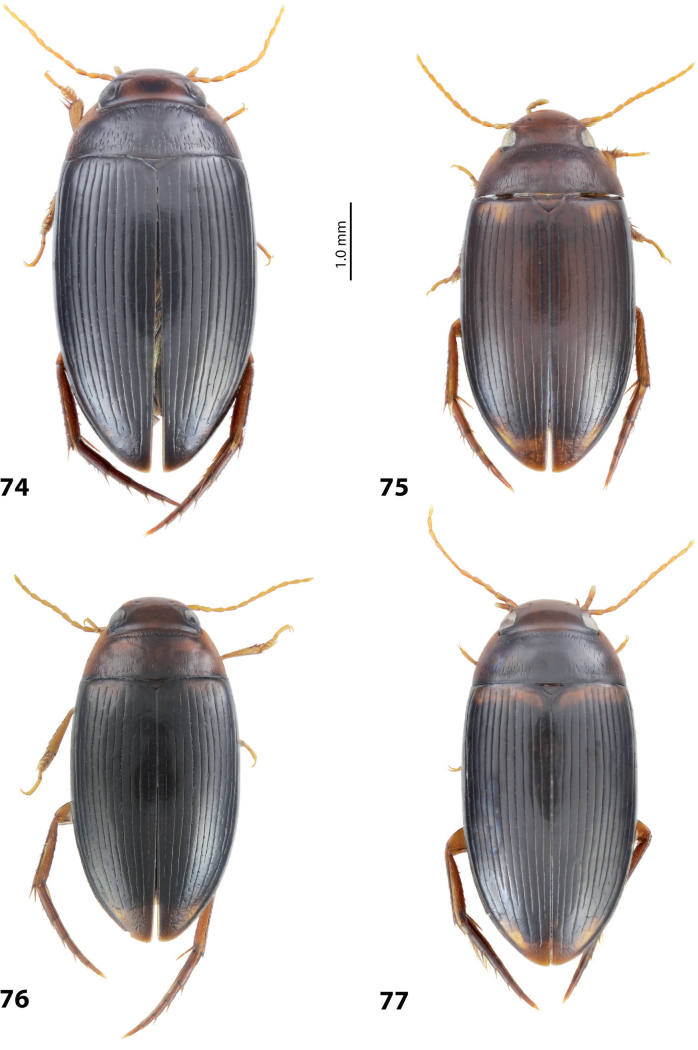
Habitus and colouration of **74***Austrelatussarmiensis* sp. nov. **75***A.rugosus* sp. nov. **76***A.debulensis* sp. nov. **77***A.moreguinensis* sp. nov.

**Figure 78.**
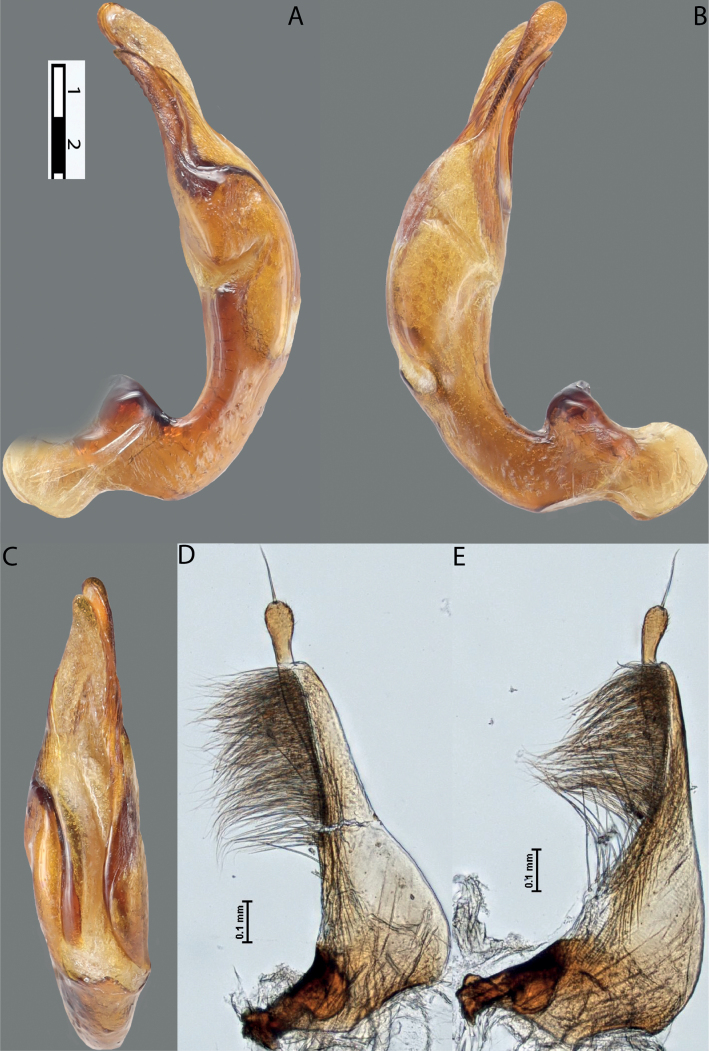
*Austrelatussarmiensis* sp. nov., median lobe **A** left lateral view **B** right lateral view **C** ventral view **D** left paramere in external view **E** right paramere in internal view. Scale bar: one unit – 0.1 mm (**A–C**).

**Figure 79.**
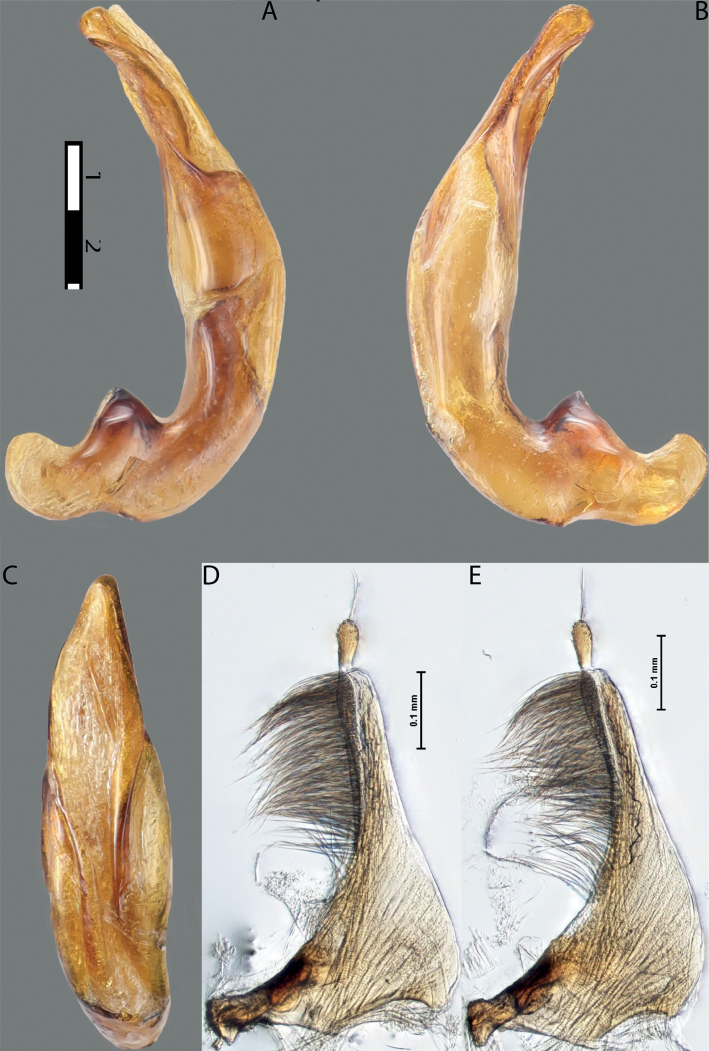
*Austrelatusrugosus* sp. nov., median lobe **A** left lateral view **B** right lateral view **C** ventral view **D** left paramere in external view **E** right paramere in internal view. Scale bar: one unit – 0.1 mm (**A–C**).

**Figure 80.**
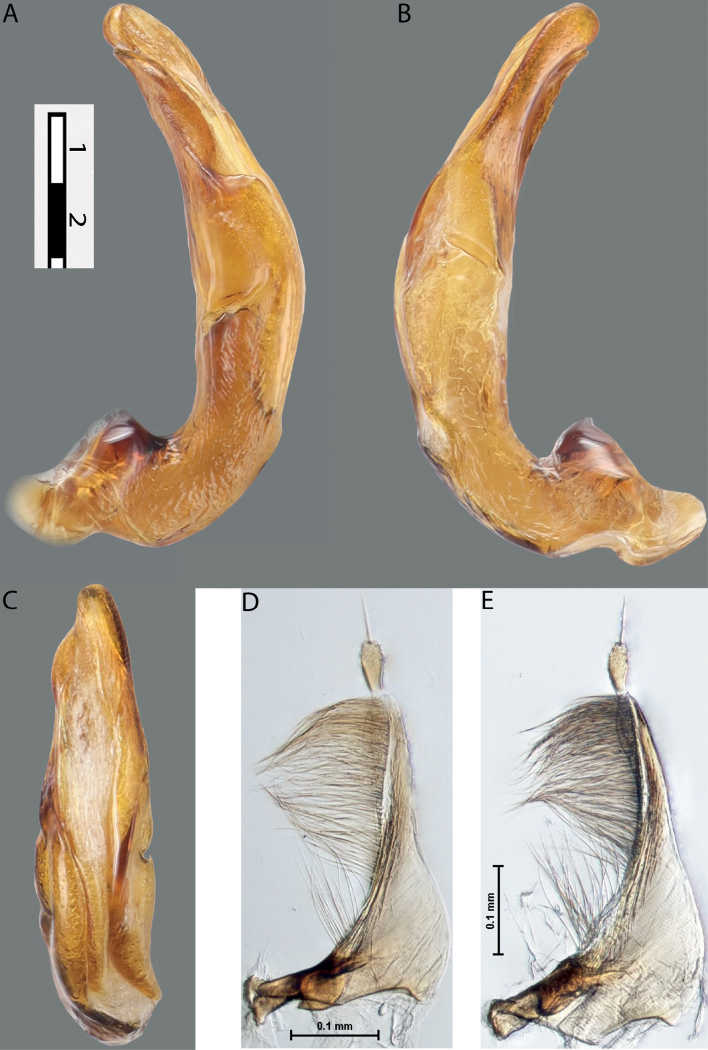
*Austrelatusdebulensis* sp. nov., median lobe **A** left lateral view **B** right lateral view **C** ventral view **D** left paramere in external view **E** right paramere in internal view. Scale bar: one unit – 0.1 mm (**A–C**).

**Figure 81.**
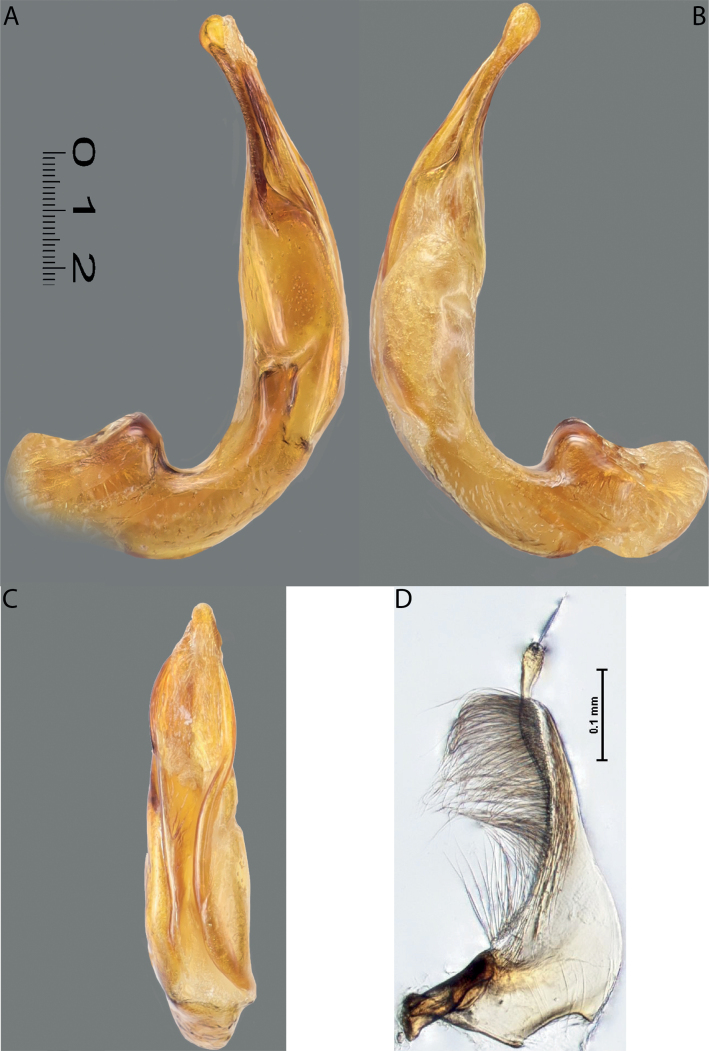
*Austrelatusmoreguinensis* sp. nov., median lobe **A** left lateral view **B** right lateral view **C** ventral view **D** left paramere in external view **E** right paramere in internal view. Scale bar: one unit – 0.1 mm (**A–C**).

**Figure 82.**
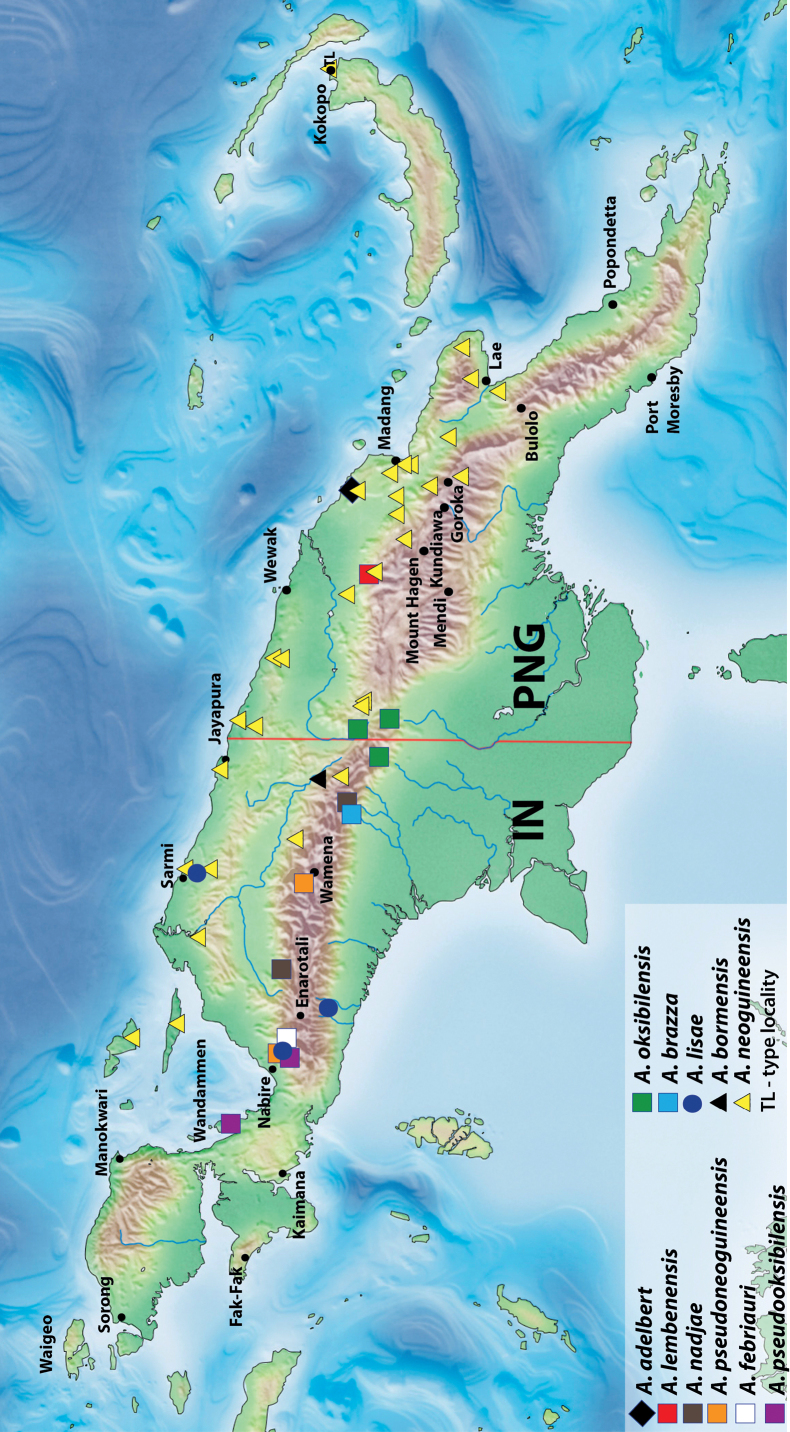
Map of New Guinea showing distribution of 11 *Austrelatus* species.

**Figure 83.**
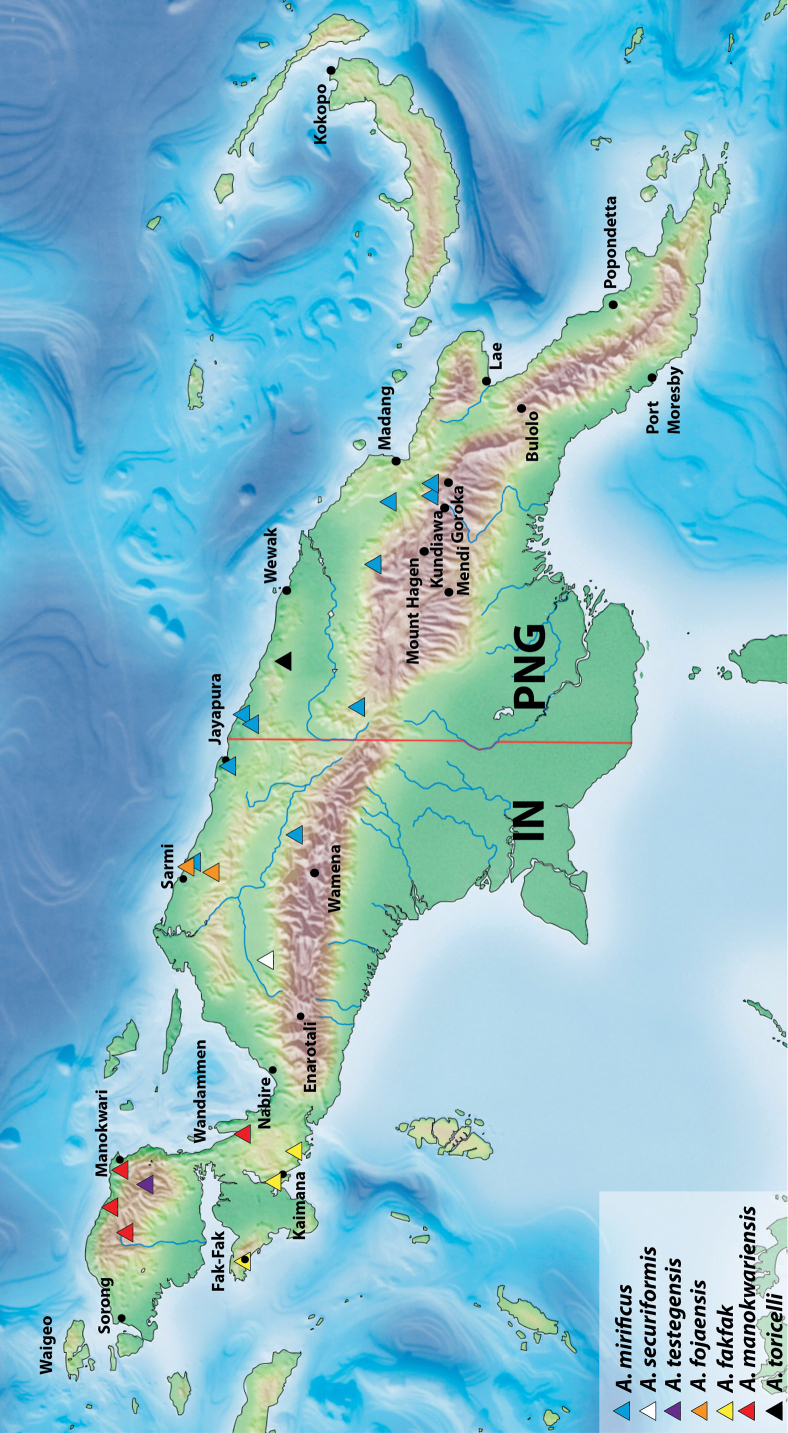
Map of New Guinea showing distribution of seven *Austrelatus* species.

**Figure 84.**
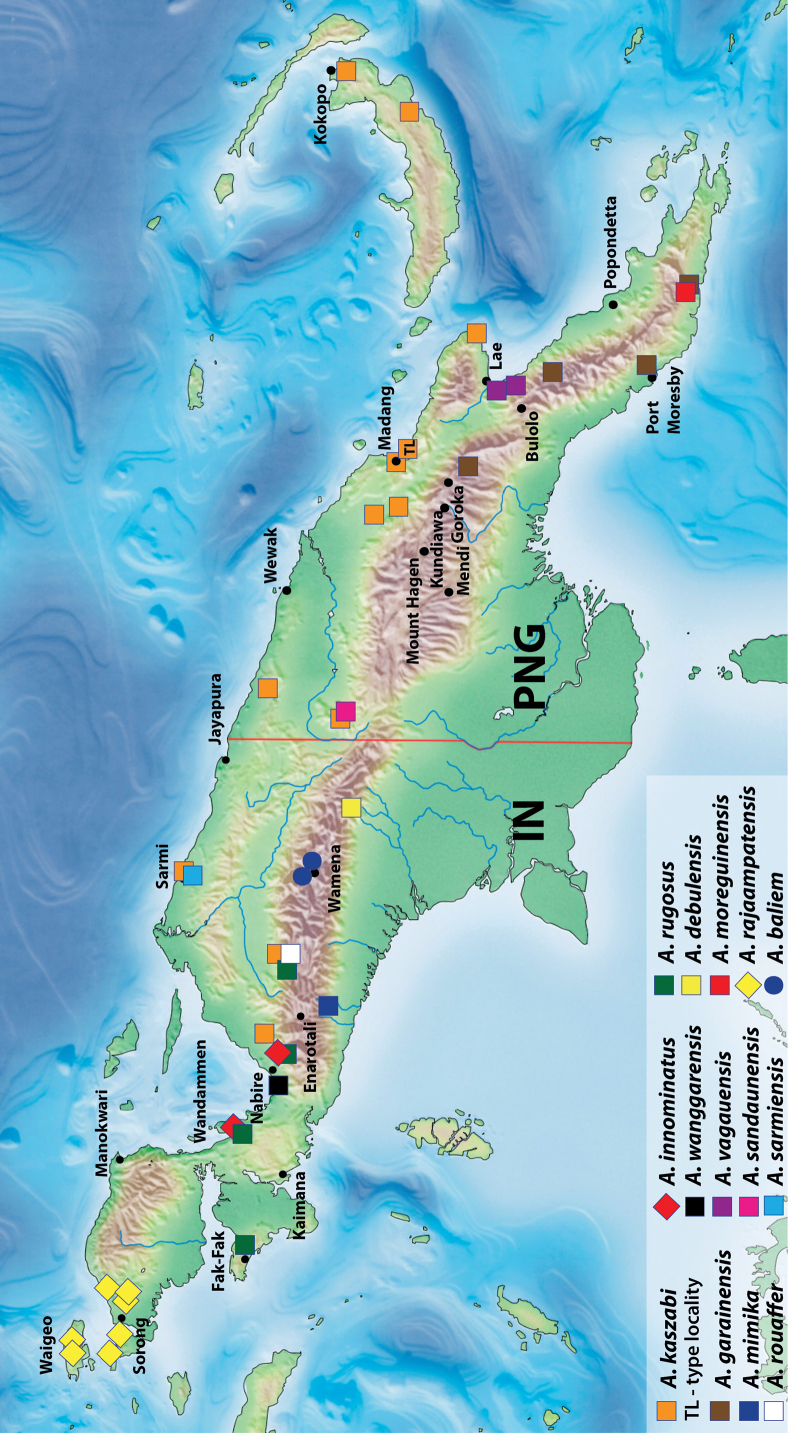
Map of New Guinea showing distribution of 14 *Austrelatus* species.

**Figures 85, 86.**
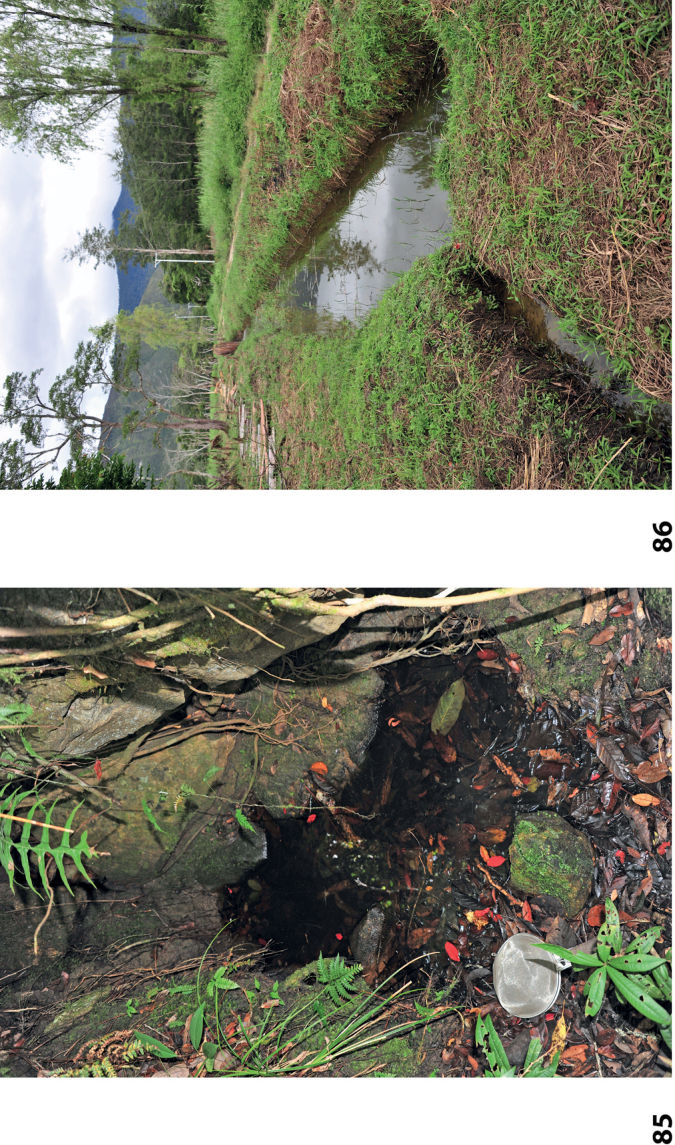
Habitats of *Austrelatusbaliem* sp. nov., Indonesia: Papua, Baliem valley **85** 15 km NE of Wamena, Jiwika, shady forest pools **86** 14 km NNE of Wamena, wetland and gardens near Jiwika, an irrigation canal. Photographs by JH.

**Figures 87, 88.**
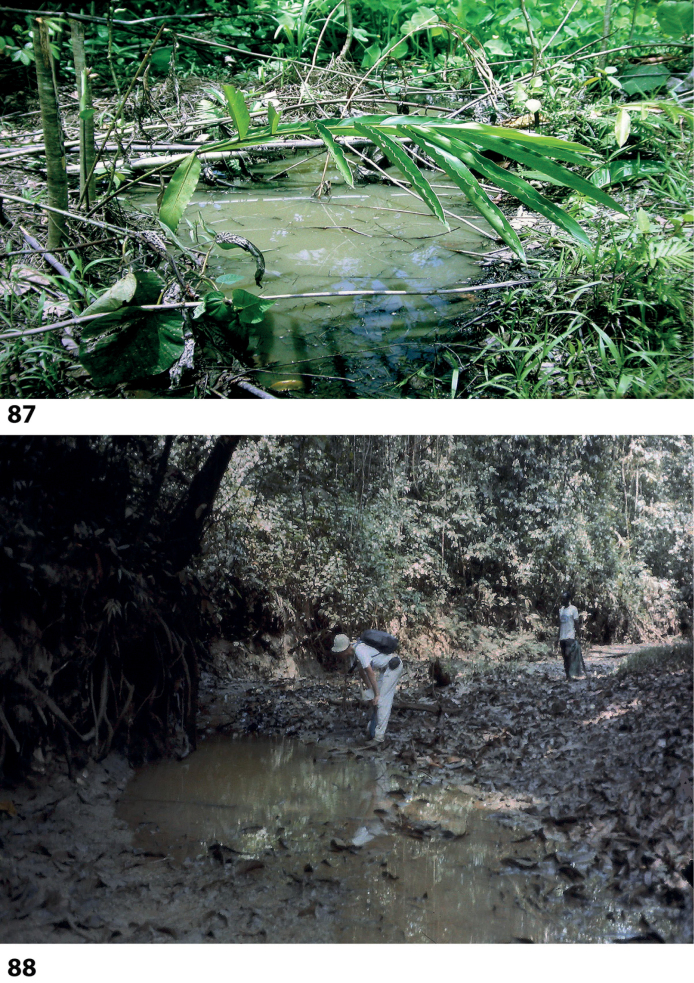
Habitats of *Austrelatus* species **87** small forest pool at track Nabire-Ilaga, Km 62: type localities of *A.innominatus* sp. nov. and *A.pseudooksibilensis* sp. nov. and habitats of *A.febrisauri* sp. nov., *A.rugosus* sp. nov., and *A.kaszabi* (Guignot, 1956) **88** partly shaded rest pool of an intermittent stream at the road Wanggar to Bali Bumi: type locality of *A.wanggarensis* sp. nov. Photographs by LH.

**Figures 89, 90.**
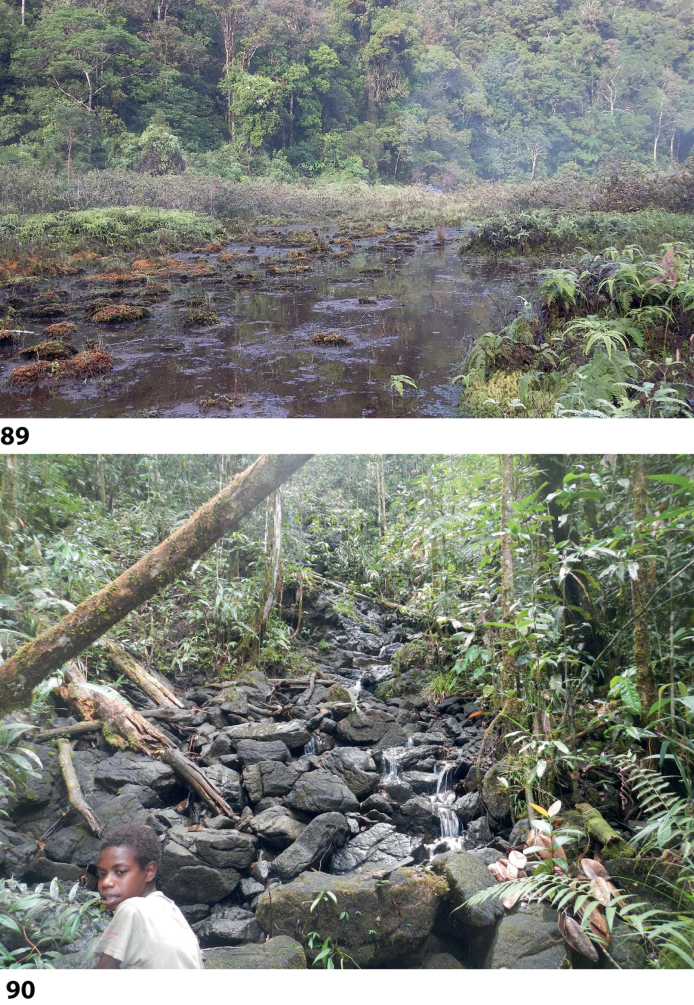
Habitats of *Austrelatus* species, Indonesia: Papua **89** Foja Mts, bog in rainforest, (PAP058): type locality of *A.fojaensis* sp. nov. **90** Rouaffer, Iratoi, forest stream, (PAP028): type locality of *A.setiphallus* sp. nov., *A.rugosus* sp. nov. and *A.rouaffer* sp. nov. and habitat of *A.kaszabi* (Guignot, 1956). Photographs by MB.

**Figures 91, 92.**
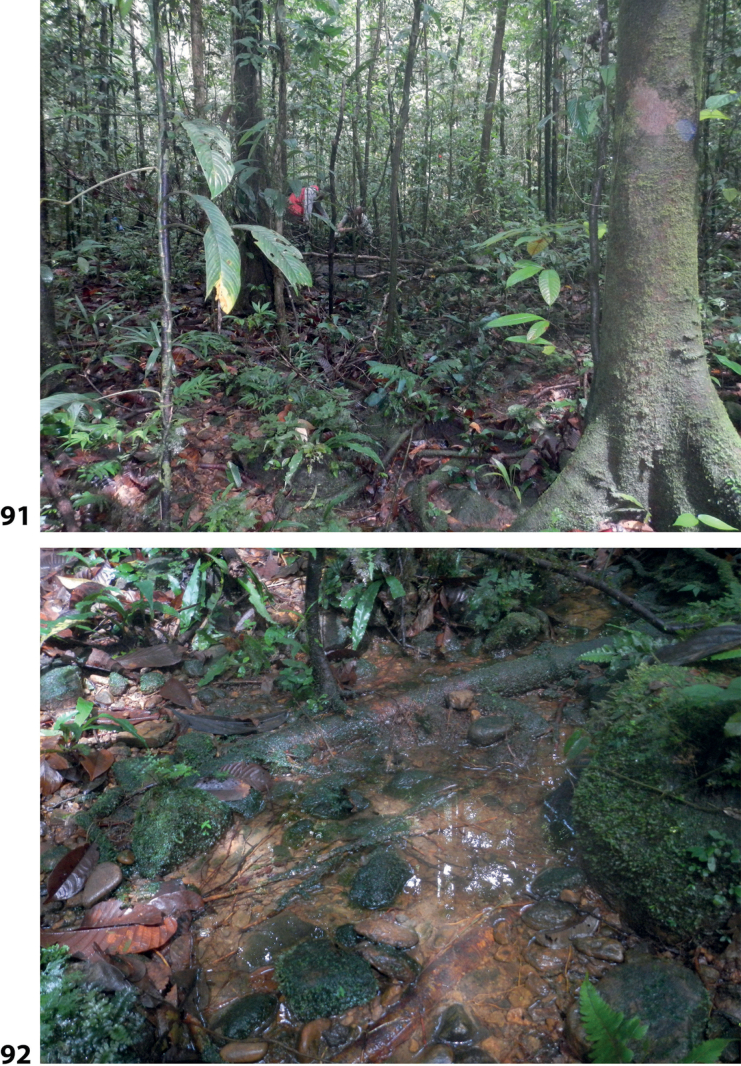
Habitat of *Austrelatus* species, Indonesia: Papua, Dekai, upper Brazza, puddles or pools among rotten leaves and twigs (PAP044): type localities of *A.brazza* sp. nov. and *A.debulensis* sp. nov., and habitat of *A.nadjae* sp. nov. Photographs by MB.

**Figure 93.**
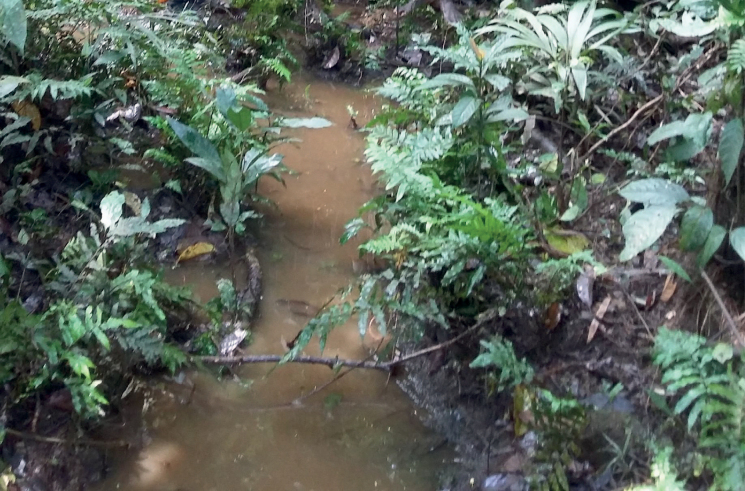
Habitat of *Austrelatus* species, Indonesia: Papua, S Iratoi, forest pool, (PAP042): type locality of *A.securiformis* sp. nov. and habitat of *A.rouaffer* sp. nov. and *A.nadjae* sp. nov. Photograph by MB.

### ﻿Key to the species of the *Austrelatusneoguineensis* group

**Table:** 

1	Elytron without distinct striae, with 2–5 rows of punctures or strioles	**2**
–	Elytron with distinct striae	**9**
2	Dorsal colouration very distinct: yellow to orange head, black pronotum with yellow to orange lateral bands, elytron with well defined, broad basal and apical yellow to orange parts. TL 5.9–6.4 mm, with oblong-oval habitus (Fig. 14). Male genitalia: median lobe with dorsal and ventral sclerites not much modified: almost straight, subequal in length, with more or less pointed apexes; left dorsal lobe with a lateral crest; left ventral lobe without a lateral sclerotised area (Fig. 18)	***A.adelbert***
–	Dorsal colouration different, uniformly piceous or with a basal yellowish red band or spot(s), often yellowish red apically. Male genitalia: median lobe similar (in *A.baliem* sp. nov., Fig. 62) or with dorsal and ventral sclerites much more modified, with apexes of different shape and left ventral lobe with a lateral sclerotised area (e.g., Figs 19, 40, 63)	**3**
3	Lobes of dorsal sclerite of median lobe narrower, with apex of right lobe swollen, of pea-shape (in lateral view)	**4**
–	Lobes of dorsal sclerite of median lobe broader, with pointed apexes of bird beak-shape (in lateral view)	**5**
4	Beetle more elongate, with less developed yellow pattern on elytron, TL 5–5.25 mm (Fig. 15). Male genitalia as in Fig. 19	***A.lembenensis* sp. nov.**
–	Beetle distinctly more oval, with more strongly developed yellow pattern on elytron, TL 4.8–5.1 mm (Fig. 16). Male genitalia as in Fig. 20	***A.nadjae* sp. nov.**
5	Elytron uniformly dark brown to piceous, sometimes with one yellow to brown latero-basal spot. TL 5.2–5.85 mm (Fig. 17). Male genitalia as in Fig. 21: lateral margin of left dorsal lobe without incisions in apical part	***A.pseudoneoguineensis* sp. nov.**
–	Elytron with more intensive yellow to reddish brown pattern: basal spots or band, apical spot, rarely narrow, longitudinal dorsal bands	**6**
6	Male anterior proclaw thicker and more strongly curved downwards apically than posterior one due to slight, subapical incision of its inner margin. Male genitalia as e.g., in Fig. 28: lateral margin of left dorsal lobe with distinct incision in apical part; left lobe of ventral sclerite with its sclerotised area large, well-developed, its apex bilobed: left part short, broad, and rounded, right one long, thin, hooked; this sclerotised area hidden under right ventral lobe and between left and right lobes of dorsal sclerite, usually invisible (only hook’s apex can be visible) in left lateral view	**7**
–	Male anterior proclaw simple. Male genitalia as in Fig. 26: lateral margin of left dorsal lobe without an incision in apical part; left lobe of ventral sclerite with its sclerotised area visible in left lateral view, elongate, weakly sclerotised, apex broadly pointed, not modified. TL ≤ 5.5 mm (Fig. 22)	***A.febrisauri* sp. nov.**
7	Elytron with more extended yellow to reddish brown pattern: usually with basal spots, apical spot, and narrow dorsal elytral band. Beetle smaller, TL ≤ 5.6 mm (Figs 24, 25). Male genitalia as in Fig. 28: lateral margin of left dorsal lobe with stronger incision in apical part	***A.pseudoksibilensis* sp. nov.**
–	Elytron with weakly expressed yellow to reddish brown pattern: usually with basal spots, apical spot, and narrow subapical lateral band. Male genitalia as in Figs 27, 33: lateral margin of left dorsal lobe with weak incision in apical part	**8**
8	Elytron without striae: 0+0, but with distinct puncture lines. Beetle slightly larger, TL 5–6.1 mm. Dorsally darker: usually piceous, with reddish yellow head, pronotal sides, and elytral basal and apical spots (Fig. 23). Male genitalia as in Fig. 27: apical part of left dorsal lobe (before incision) more elongate and part after incision flatter; apical part of right dorsal lobe more elongate	***A.oksibilensis* sp. nov.**
–	Elytron without or with 6–10 dorsal striae, submarginal stria absent: (0–10)+0. Beetle slightly smaller, TL = 4.95–5.6 mm. Dorsally paler: usually brown, with reddish yellow head, pronotal sides, and elytral basal and apical spots (Figs 29, 30). Male genitalia as in Fig. 33: apical part of left dorsal lobe (before incision) smaller, shorter, and part after incision more prominent; apical part of right dorsal lobe shorter, more rounded	***A.brazza* sp. nov.**
9	Elytron with 2–10 more or less complete striae: usually with 6 striae (some striae can be absent or additional striae or strioles can be present in between); with or without submarginal stria	**10**
–	Elytron with 11 complete dorsal striae, striae 1–3, 10 can be reduced basally; with or without submarginal stria	**23**
10	Male anterior proclaw more strongly curved downwards apically than posterior one due to slight incision of its inner margin. Male genitalia as e.g., in Fig. 34: left lobe of ventral sclerite with its sclerotised area large, well-developed, its apex bilobed (left part short, broad, rounded and right one long, thin, hooked) or elongate, curved to left, hook-like; this sclerotised area hidden under right ventral lobe and between left and right lobes of dorsal sclerite, usually hook’s apex can be visible in left lateral view	**11**
–	Male anterior proclaw simple. Male genitalia as e.g., in Fig. 35: left lobe of ventral sclerite with its sclerotised area more or less visible in left lateral view, more weakly sclerotised, elongate or rather broad, with its apex broadly pointed, truncate or rounded	**13**
11	Beetle larger, TL ≥ 6 mm. Elytron with 6+0 striae (Fig. 31). Male anterior proclaw less strongly modified, thinner. Male genitalia in Fig. 34: left dorsal lobe shorter than right one, without incision on its lateral margin; left lobe of ventral sclerite with its sclerotised area not bilobed apically, elongate, curved to left, hook-like	***A.lisae* sp. nov.**
–	Beetle smaller, TL ≤ 5.6 mm. Elytron with (0–10)+0 striae. Male anterior proclaw thicker, more strongly modified: incision of its inner margin subapical, stronger. Male genitalia: left dorsal lobe almost as long as right one, with distinct incision on its lateral margin; left lobe of ventral sclerite with its sclerotised area bilobed apically: left part short, broad, rounded and right one long, thin, hooked	**12**
12	Elytron without or with 6–10 more or less complete dorsal striae, submarginal stria absent: (0–10)+0 (Fig. 29). Elytron with weakly expressed yellow to reddish brown pattern: usually with basal spots, apical spot, and narrow subapical lateral band. Male genitalia as in Fig. 33: lateral margin of left dorsal lobe with weak incision in apical part	***A.brazza* sp. nov.**
–	Elytron with 3–9 more or less complete dorsal striae, submarginal stria absent: (3–9)+0. Elytron with more extended yellow to reddish brown pattern: usually with basal spots, apical spot, and narrow dorsal elytral band (Figs 24, 25). Male genitalia as in Fig. 28: lateral margin of left dorsal lobe with stronger incision in apical part	***A.pseudoksibilensis* sp. nov.**
13	Male genitalia as in Figs 35, 40: lobes of dorsal sclerite subequal; right dorsal lobe broad, flat, with pointed apex. Beetle usually larger, TL ≥ 5.5 mm.	**14**
–	Male genitalia as e.g., in Fig. 41: left lobe of dorsal sclerite subequal to or shorter than right one; right dorsal lobe narrower, with rounded, “swollen”, sometimes pea-like apex. Beetle usually smaller, TL ≤ 5.5 mm	**15**
14	Beetle usually smaller, TL 5.4–6.1 mm, narrower, MW 2.5–3.05 mm, more elongate, TL/MW 2–2.16. Pronotum with numerous strioles. Number of elytral striae very variable: (5–10)+(0–1) (Fig. 32). Protibia modified: thinner proximally and broader medially and distally due to its curved ventral margin. Male genitalia in Fig. 35: lobes of ventral sclerite straight apically; sclerotised area of left ventral lobe long, with thin, straight apex in left lateral view	***A.bormensis* sp. nov.**
–	Beetle usually larger, TL 5.2–6.9 mm, broader, MW 2.6–3.35 mm, more oval, TL/MW 2–2.06. Pronotum with few strioles at posterolateral angles. Number of elytral striae 6+(0–1) (Fig. 36). Protibia straight. Male genitalia as in Figs 11, 40: lobes of ventral sclerite usually slightly curved upwards apically; sclerotised area of left ventral lobe small, short, sometimes indistinct in left lateral view	***A.neoguineensis***
15	Habitus oval, TL/MW 1.84–1.9, TL 4.85–5.5 mm, MW 2.6–2.9 mm. Dorsally with yellow head, pronotal sides, and very prominent yellow elytral colouration of basal band/spots and narrow longitudinal bands; if elytral colour pattern maximally reduced, then present as basal band and interrupted apicolateral band; if fully developed, elytra almost completely yellow, with piceous disc and narrow spaces laterally. Number of elytral striae very variable: (2–10)+(0–1) (Fig. 41). Male genitalia as in Fig. 41	***A.mirificus* sp. nov.**
–	Habitus oblong-oval, TL/MW ≥ 2. Elytron without yellow pattern or with more or less distinct basal and apical spots but without longitudinal bands	**16**
16	Elytron without basal spots, apical spot present or absent	**17**
–	Elytron usually with distinct basal and apical spots	**18**
17	Elytron only with apical spot; with 6+1 striae; submarginal stria apical, interrupted. TL 5.3 mm (Fig. 38). Male genitalia as in Fig. 42: left ventral lobe with a large sclerotised area, with axe-like apex curved to left	***A.securiformis* sp. nov.**
–	Elytron without yellow apical spot; with 6+1 striae and small strioles between them; submarginal stria well-developed. TL 5.1 mm (Fig. 39). Male genitalia as in Fig. 43: left ventral lobe with its sclerotised area broad, elongate, with rounded apex	***A.testegensis* sp. nov.**
18	Elytron with 6+1 striae	**19**
–	Elytron with 10+(0–1) more or less complete striae, seldom with 6 complete and 4 reduced and interrupted striae	**21**
19	Male genitalia as in Fig. 48: left dorsal lobe with strongly curved downwards apex and with distinct lateral crest interrupted into apical and basal parts; left ventral lobe shorter, broader, more concave. TL 5.1–5.5 mm (Fig. 44)	***A.fojaensis* sp. nov.**
–	Male genitalia: left dorsal lobe with weakly curved downwards apex and with weak, long, lateral crest; left ventral lobe more elongate and narrower	**20**
20	Beetle larger, TL 4.4–5 mm. Elytron piceous, with indistinct, yellowish red, two basal and one small apical spots (Fig. 45). Male genitalia in Fig. 49: right dorsal lobe more elongate	***A.fakfak* sp. nov.**
–	Beetle larger, TL 4.6–5.7 mm. Elytron piceous, with two distinct yellowish red, often confluent basal spots and apical spot larger (Fig. 46). Male genitalia in Fig. 50: right dorsal lobe more “swollen”, shorter, more rounded	***A.manokwariensis* sp. nov.**
21	Male genitalia as in Fig. 56: sclerotised area of left ventral lobe more distinct, strongly developed, with broad, rounded apex. Elytron usually with 10–11 complete dorsal striae, seldom with 6 complete and 4 reduced and interrupted striae; submarginal stria present. TL 4.7–5.6 mm (Figs 52, 53)	***A.innominatus* sp. nov.**
–	Male genitalia: sclerotised area of left ventral lobe weakly developed, with long, thin, straight apex in left lateral view	**22**
22	Beetle larger, TL 5.7 mm. Elytron with 10+1 striae (Fig. 47). Male genitalia as in Fig. 51: apex of left ventral lobe curved upwards; apex of right dorsal lobe less rounded	***toricelli* sp. nov.**
–	Beetle smaller, TL 4.15–4.7 mm. Elytron with (10–11)+(0–1) striae (Figs 54, 55). Male genitalia as in Fig. 57: apex of left ventral lobe curved downwards; apex of right dorsal lobe rounded, pea-like	***A.rajaampatensis* sp. nov.**
23	Median lobe of aedeagus with dorsal and ventral sclerites not much modified: almost straight, subequal in length, with more or less pointed apexes; left dorsal lobe with a lateral crest and tiny surface scales; left ventral lobe without a lateral sclerotised area (Fig. 62). Beetle smaller, TL 4.65–5.6 mm, with oblong-oval to elongate habitus. Elytron with 11+(0–1) striae. Dorsally piceous, with brownish head and pronotal sides, sub-matt due to rather strong microreticulation (Fig. 58)	***A.baliem* sp. nov.**
–	Median lobe of aedeagus with dorsal and ventral sclerites modified, with apexes of different shape; left dorsal lobe differently shaped, with or without lateral crest(s), without surface scales, its apex usually curved downwards; right dorsal lobe with a “swollen”, often pea-like apex; left ventral lobe with a distinct lateral sclerotised area (e.g., Figs 12, 63). Beetle usually with more oval habitus, shiner	**24**
24	Male genitalia as in Figs 63–65: left ventral lobe with very large, strong sclerotised area, its apex hook-likely curved to left	**25**
–	Male genitalia as e.g., in Figs 70, 72: left ventral lobe with weaker sclerotised area, its apex pointed or rounded	**27**
25	Male genitalia as in Figs 64, 65: lobes of ventral sclerite almost completely sclerotised, only with narrow membranous areas medially; sclerotised area of left ventral lobe with large, apical hook, well-visible in lateral left and ventral views	**26**
–	Male genitalia as in Figs 12, 63: lobes of ventral sclerite weakly sclerotised, with large membranous areas medially; sclerotised area of left ventral lobe with smaller apical hook, slightly visible in lateral left view and not visible ventral view due to large membranous areas of ventral lobes. TL 5–6.45 mm (Fig. 59)	***A.kaszabi***
26	Beetle larger, TL 5.4–6.5 mm. Elytron with three or two yellowish red basal spots confluent forming posteriorly notched basal band (Fig. 60). Male genitalia as in Fig. 64: left dorsal lobe distinctly shorter that right one, with long lateral crest; sclerotised area of left ventral lobe as long as left dorsal lobe, with long, thin slightly curved to left apex	***A.garainensis* sp. nov.**
–	Beetle smaller, TL 5.25–5.6 mm. Elytron with posteriorly notched basal band formed by three confluent spots (Fig. 61). Male genitalia as in Fig. 65: left dorsal lobe as long as right one, with its lateral crest interrupted into short apical and long and very broad basal parts; sclerotised area of left ventral lobe shorter than left dorsal lobe, broad, with large, angulate prolongation to left medially and with long, thin, hook-likely curved to left apex	***A.mimika* sp. nov.**
27	Male genitalia as in Figs 56, 70, 71: left ventral lobe more strongly sclerotised, apex of sclerotised area broader, rounded	**28**
–	Male genitalia as e.g., in Figs 72, 80: left ventral lobe weakly sclerotised, apex of sclerotised area thin, narrow	**30**
28	Elytron piceous, without yellow basal band, very seldom with one or two very small basal spots. Elytron with 11+1 complete, strongly impressed striae. Pronotum with numerous, strongly impressed strioles (Fig. 66). Females dimorphic. Male genitalia as in Fig. 70	***A.rouaffer* sp. nov.**
–	Elytron with yellow basal band. Elytron with 11+1 striae; striae weaklier impressed, especially striae 1–3; stria 1 can be interrupted or reduced basally. Pronotum without or with few weak strioles laterally. Females not dimorphic	**29**
29	Male genitalia as in Fig. 56: sclerotised area of left ventral lobe distinctly broader, apex of left dorsal lobe more curved down, with crest. Beetle usually larger, TL > 5 mm (Figs 52, 53)	***A.innominatus* sp. nov.**
–	Male genitalia as in Fig. 71: sclerotised area of left ventral lobe distinctly more elongate, apex of left dorsal lobe less curved down, without crest. Beetle smaller, TL 4.9 mm (Fig. 67)	***A.wanggarensis* sp. nov.**
30	Beetle larger, TL 6.1–7 mm. Pronotum with numerous strioles. Elytron with 11+1 complete, strongly impressed striae, with three yellowish red basal spots (Fig. 68). Male genitalia as in Fig. 72: apex of left dorsal lobe curved downwards and distinctly to left; its dorsal and lateral surface with distinct denticulation visible in left lateral view	***A.vagauensis* sp. nov.**
–	Beetle smaller, TL < 6 mm	**31**
31	Beetle larger, TL > 5.5 mm (Fig. 69). Male genitalia as in Fig. 73	***A.sandaunensis* sp. nov.**
–	Beetle smaller, TL < 5.5 mm	**32**
32	Elytron with yellow basal pattern vague (Fig. 74). Male genitalia as in Fig. 78: left dorsal lobe almost straight to apex, with weak, longitudinal crest, its dorsal surface with distinct denticulation visible in left lateral view	***A.sarmiensis* sp. nov.**
–	Elytron with yellow basal pattern more distinct. Male genitalia as in Figs 57, 79–81: shape of sclerites different	**33**
33	Male genitalia as in Figs 79, 80: dorsal lobes subequal, apex of right dorsal lobe less protruding. Beetle usually smaller, TL 4.1–4.7 mm	**34**
–	Male genitalia as in Figs 57, 81: left dorsal lobe distinctly shorter right dorsal lobe, apex of right dorsal lobe more protruding. Beetle usually larger, TL 4.4–5.6 mm	**35**
34	Elytron paler, with yellow basal spots starting at elytron middle, seldom near suture; sports can be confluent and look like a short posteriorly notched band at middle of elytron base. Pronotal and elytral striation more strongly developed; pronotum with numerous strioles. TL 4.15–4.7 mm (Fig. 75). Male genitalia as in Fig. 79: apexes of dorsal lobes shorter; apical part of left dorsal lobe shorter and more strongly curved downwards; median impression of the right ventral lobe situated more laterally in right lateral view	***A.rugosus* sp. nov.**
–	Elytron darker, with yellowish red basal band, it can be less prominent or slightly reduced near suture. Pronotal and elytral striation more weakly developed: pronotum without numerous strioles. TL 4.1–4.5 mm (Fig. 76). Male genitalia as in Fig. 80: apexes of dorsal lobes more elongate; apical part of left dorsal lobe longer and more straight; median impression of the right ventral lobe situated more ventrally in right lateral view	***A.debulensis* sp. nov.**
35	Male genitalia as in Fig. 81: apex of left dorsal lobe more curved to left; apex of right dorsal lobe more elongate. Elytron with 11+1 striae: dorsal striae complete; submarginal stria well-developed, sometimes reaching ½ of elytron (Fig. 81)	***A.moreguinensis* sp. nov.**
–	Male genitalia as in Fig. 57: apex of left dorsal lobe more curved downwards, apex of right dorsal lobe more rounded, pea-like. Elytron with (10–11)+(0–1) striae: dorsal striae complete or interrupted and reduced, especially striae 1–3; submarginal stria present or absent	***A.rajaampatensis* sp. nov.**

## Supplementary Material

XML Treatment for Austrelatus

XML Treatment for Austrelatus clarki

XML Treatment for Austrelatus fumato

XML Treatment for Austrelatus setiphallus

XML Treatment for Austrelatus adelbert

XML Treatment for Austrelatus baliem

XML Treatment for Austrelatus bormensis

XML Treatment for Austrelatus brazza

XML Treatment for Austrelatus debulensis

XML Treatment for Austrelatus fakfak

XML Treatment for Austrelatus febrisauri

XML Treatment for Austrelatus fojaensis

XML Treatment for Austrelatus garainensis

XML Treatment for Austrelatus innominatus

XML Treatment for Austrelatus kaszabi

XML Treatment for Austrelatus lembenensis

XML Treatment for Austrelatus lisae

XML Treatment for Austrelatus manokwariensis

XML Treatment for Austrelatus mimika

XML Treatment for Austrelatus mirificus

XML Treatment for Austrelatus moreguinensis

XML Treatment for Austrelatus nadjae

XML Treatment for Austrelatus neoguineensis

XML Treatment for Austrelatus oksibilensis

XML Treatment for Austrelatus pseudoneoguineensis

XML Treatment for Austrelatus pseudooksibilensis

XML Treatment for Austrelatus rajaampatensis

XML Treatment for Austrelatus rouaffer

XML Treatment for Austrelatus rugosus

XML Treatment for Austrelatus sandaunensis

XML Treatment for Austrelatus sarmiensis

XML Treatment for Austrelatus securiformis

XML Treatment for Austrelatus testegensis

XML Treatment for Austrelatus toricelli

XML Treatment for Austrelatus vagauensis

XML Treatment for Austrelatus wanggarensis

## ﻿Additional information

### Author ORCIDs

Helena Shaverdo http://orcid.org/0000-0001-5034-7342

Jiří Hájek https://orcid.org/0000-0001-5779-1542

Lars Hendrich https://orcid.org/0000-0001-8366-0749

Suriani Surbakti https://orcid.org/0000-0003-3984-2187

Michael Balke https://orcid.org/0000-0002-3773-6586

### Data availability

All of the data that support the findings of this study are available in the main text or Supplementary Information.

## ﻿Appendix 1

**Material studied for the species with larger numbers of specimens**

***Austrelatusgarainensis* sp. nov.**

**Additional paratypes: *PNG*: *Morobe***: 45 males, 57 females “Papua New Guinea: Garaina, 800 m, vi.2008, 07.53.091S 147.07.915E Ibalim & Sosanika PNG217” (BMNH, NHMW, ZSM). 22 males, 29 females “Papua New Guinea: Garaina, 800 m, 27.vi.2009 (PNG220) 7.52.669S 147.07.196E Ibalim & Sosanika”, one male additionally with a green label “3846” (NHMW, ZSM). 25 males, 18 females “Papua New Guinea: Morobe, Garaina, 770 m, 25.vi.2008, 07.50.859S 147.08.614E, Ibalim & Sosanika, (PNG222)” (NHMW, ZSM). 5 females “Papua New Guinea: Garaina, 820 m, 24.vi.2008 07.52.287S 147.06.297E, Ibalim & Sosanika, (PNG224)” (ZSM). 1 male “Papua N.G.: Morobe Prov. Garaina, 700 m 21.3.1998 leg. A. Riedel” (NHMW).

***EHL***: 1 female “6441” [green text], “Papua New Guinea: Eastern Highlands, Bena – pass to Goroka valley, 1550 m, 5.iv.2006, 06.14.567S 145.29.634E, Balke & Sagata (PNG33)” (ZSM). 1 male “Papua New Guinea: Eastern Highlands, Bena Brigde, Unggai Bena, 1393 m, 27.viii.2005, K.Sagata, (WB136)” (ZSM).

***Central***: 4 males, 2 females “Papua New Guinea: Central, Moreguina [10°00'57"S, 148°28'27"E] 16.viii.2008 Posman (PNG183), one male additionally with a green label “3815” (NHMW, ZSM). 6 males, 2 females “Papua New Guinea: Central, 755 m 28.x.2009, S9 25 47.5 E147 32 59.1, Sagata (PNG229)” (NHMW, ZSM). 1 female “Papua New Guinea: Central, Moroka area, Kailaki Wareaga ridge, 768 m, 27.x.2009, S9 25 42.4 E147 31 06.8, Sagata (PNG227)” (ZSM). 1 male “New Guinea: Central Prov. 20 km E Pt.Moresby 9°27'S Varirata Nat. Park 147°21'E 800 m 23.IV.1989 water sample from palm-leaf leg. M. Holynsko” [hw] (ZSM).

***National Capital District***: 7 males, 11 females “Papua New Guinea: National Capital District, Varirata NP, 600 m, 16.xii.2007, 09.26.13S 147.22.09E, Balke & Sagata (PNG 159)”, two males additionally with green labels “2848” and “2849” (NHMW, ZSM).

***Milne Bay***: 2 males “Papua New Guinea: Milne Bay, Normanby Isl., Sewa Bay, Sibonai, 35 m,”, “30.vi.2017, 10°02.418'S 150°58.461'E, Riedel”, “8725” and “8726” [green text] (ZSM).

***Austrelatusinnominatus* sp. nov.**

**Additional paratypes: *IN*: *Papua*: *Nabire Regency***: 4 males, 3 females “Ir 23-W. New Guinea, track Nabire-Ilaga KM 62, 250 m, 24.vii.1991 Balke & Hendrich leg.” (NHMW, ZSM). 1 male, 2 females “Indonesia: Papua, Road Nabire-Enarotali KM 55, 774 m, 22.x.2011, 03.29.796S 135.43.885E, Balke (PAP09)”, male and one female with additional green text labels “5129” and “5131” (ZSM). 3 males, 3 females “West New Guinea/Paniai Prov./IR 20 track Nabire-Ilaga KM 59, ca.750 m, 18.7.1991 leg: Balke & Hendrich leg.” (CLH). 1 female “IR 21 – W. New Guinea, track Nabire-Ilaga KM 65, Kali Utowa, 250M, 18.-19.vii.1991 Balke & Hendrich leg.” (ZSM). 1 female “Irian Jaya: Paniai Prov. road Nabire – Ilaga, km 38 18.9.1996, 150 m, leg. M. Balke (96 # 26)” (NHMW). 1 female “Irian Jaya: Nabire Prov. rd. Nabire – Ilaga, Km 35 Kali Cemara, 100 m, 27.9.1997, leg. Balke (IR97#6)” (NHMW). 1 male “West New Guinea/Paniai Prov./IR 18 River n. Nabire, 2 m, 15.7.1991 leg: Balke & Hendrich leg.” (CLH). 1 male “Irian Jaya: Nabire Prov. rd. Nabire – Ilaga, Km 35 Kali Cemara, 100 m, 23.10.1997, leg. Balke (IR97#14)” (NHMW). 2 males, 4 females “Irian Jaya: Paniai Prov. road Nabire – Ilaga, km 65, 29.8.1996, 250 m leg. M. Balke (96 # 6)” (NHMW). 1 male “Irian Jaya: Paniai Prov. road Nabire – Ilaga, km 117 Unipo, 2.9.1996, 150 m leg. M. Balke (96 # 12)” (ZSM).

***West Papua*: *Teluk Wondama Regency***: 1 male “IRIAN JAYA: Wandammen Bay, Wasior, Sararti 100–200 m, 7-9.I.2001 Leg. A.RIEDEL” (SMNS).

***Austrelatuskaszabi* (Guignot, 1956)**

**Additional material**:

***IN*: *Papua*: *Nabire Regency***: 2 males, 2 females “Irian Jaya: Paniai Prov. road Nabire – Ilaga, km 117 Unipo, 2.9.1996, 150 m leg. M. Balke (96 # 12)” (NHMW). 1 male “Indonesia: Papua, Road Nabire-Enarotali KM 62, 340 m, 22.x.2011, 03.31.684S 135.42.802E, UNCEN team (PAP11)” (ZSM). 2 females “Indonesia: Papua, Road Nabire-Enarotali KM 80, 250 m, 22.x.2011, 03.33.860S 135.46.473E, UNCEN team (PAP12)” (ZSM).

***Sarmi Regency***: 1 male, 2 females “Indonesia: Papua, Sarmi area 70 m, 25.ix.2014, -1.9713 138.8491, UNCEN team (Pap032)”, male with an additional green label “6470” (ZSM).

***Puncak Regency***: 1 male, 1 female “Indonesia: Papua, Rouaffer, Iratoi, hill in forest, 164 m, 6.ix.2014, -3,2403 137,3329, Sumoked (PAP028)” (ZSM).

***PNG*: *Sandaun***: 23 male, 23 female: 1 male “Papua New Guinea: Sandaun, Mianmin, 670 m 20.x.2008, 4.53.292S 141.34.118E, Ibalim (PNG 191)” (NHMW, ZSM). 4 males, 3 females “Papua New Guinea: Sandaun, Mianmin, 670 m, 22.x.2008, 04.53.329S 141.35.263E S. Ibalim PNG189”, one male with an additional green label “3775” (NHMW, ZSM). 2 males, 2 females “Papua New Guinea: Sandaun, Mianmin (pool), 990 m, 23.x.2008, 4.54.570S 141.35.490E, Ibalim (PNG 193)” (ZSM). 2 females “Papua New Guinea: Sandaun, Mianmin (pool), 1080 m, 24.x.2008, 04.55.780S 141.38.185E, Ibalim (PNG 196)” (ZSM). 6 males, 1 female “Papua New Guinea: Sandaun, Mianmin (river), 700 m, 21.x.2008, 04.52.858S 141.31.706E Ibalim (PNG 197)” (ZSM). 9 males, 9 females “Papua New Guinea: Sandaun, Mianmin (pool), 700 m, 21.x.2008, 04.52.858S 141.31.706E, Ibalim (PNG 198)” (NHMW, ZSM). 4 males, 3 females “Papua New Guinea: Sandaun, Mianmin area, >700 m, 7.i.2010, Ibalim & Pius (PNG231)”, two males and one female with additional green text labels “6015”, “6019” and “6014” respectively (ZSM). 1 male “Papua New Guinea: Sandaun, Toricelli Mts., 2h walk fr Sibilanga Stn, 350 m, 19.iv.2006, 03.39.121S 142.29.991E, Balke (PNG 44)”, “3233” [green label] (ZSM). 1 male, 1 female “Papua New Guinea: Sandaun, Bewani Stn., stream@base of Bewani Mts., 200–300 m, 12.iv.2006, nr. 03.05.130S 141.10.227E, Balke & Sagata (PNG 37)” (ZSM). 1 female “Papua New Guinea: Sandaun, Bewani Stn., forest puddles @ base of Bewani Mts., 300 m, 12.iv.2006, nr. 03.05.130S 141.10.227E, Balke & Sagata (PNG 38)”, “3211” [green label] (ZSM).

***East Sepik***: 2 males, 2 females “Papua New Guinea: East Sepik, Prince Alexander Mts., Wewak-Angoram, 300 m, 21.iv.2006, 03.42.129S 143., Balke (PNG 46)” (ZSM).

***Madang***: 1 male, 2 females “Papua New Guinea: Madang, Wannang, 270 m, 31.x.2008, 05.15.458S 145.02.389E, Posman (PNG187)” (ZSM). 1 male “Papua New Guinea: Madang, Wannang, 230 m 3.x.2008, 05.17.235S 145.06.160E, Posman (PNG188)”, “3766” [green label] (ZSM). 2 males, 1 female “Papua New Guinea: Wanang, village, 20.ix.2013, Boukal, 13/2013” (ZSM). 3 males, 2 females “Papua New Guinea: Wanang I, river, 25.ix.2013, Boukal, 44/2013” (NHMW, ZSM). 1 female “Papua New Guinea: Wanang III, 4–20.vii.2013, V.Kolar.” (ZSM). 8 males, 6 females “Papua New Guinea: Wanang, Digitam Tributary, 23.ix.2013, Boukal, 33/2013” (NHMW, ZSM). 3 males, 2 females “Papua New Guinea: Digitam creek, smallside pools, 23.ix.2013, Boukal, 37/2013” (NHMW, ZSM). 3 males, 2 females “Papua New Guinea: Wanang, Kusa, 24.ix.2013, Boukal, 39/2013” (NHMW, ZSM). 1 male, 1 female “Papua New Guinea: Madang, Brahmin, 150 m, 26IX2002, 05.44.953S 145.20.844E Balke & Sagata (PNG 024)” (ZSM). 1 male “277” [green label], “PNG: Madang Pr. Brahmin (PNG 24), 26IX2002, Balke, MB 277” (ZSM). 5 males, 10 females “Papua New Guinea: Madang, Usino, 260 m, 15.iii.2007, 05.31.125S 145.25.316E, Kinibel (PNG 158)” (NHMW, ZSM). 8 males, 15 females “Papua New Guinea: Madang, middle ramu [Ramu River], Akaraski, 50 m, 12.iii.2007, 05.04.787S 144.43.137E, Kinibel (PNG 157)”, one male and three females with additional green labels “2867”, “2880”, “2868” and “2879”, respectively (NHMW, ZSM). 1 female “Ibisca Niugini, PNG 20–22.xi.2012 Wanang -5,227670193 145,0797424”, “FIT-WAN-D-2/8-d03 / Plot 4 / P0573 Vial 17617-CODYTI” (ZSM). 1 female “Ibisca Niugini, PNG 20–22.xi.2012 Wanang -5,227670193 145,0797424”, “FIT-WAN-R-2/8-d04 / Plot 18 / P0685 Vial 03974-CODYTI” (ZSM). 1 female “Ibisca Niugini, PNG 20–22.xi.2012 Wanang -5,227670193 145,0797424”, “FIT-WAN-O-2/8-d04 / Plot 15 / P0661 Vial 22269-CODYTI” (ZSM). 1 ex. “Ibisca Niugini, PNG 20–22.xi.2012 Wanang -5,227670193 145,0797424”, “FIT-WAN-Q-2/8-d04 / Plot 17 / P0677 Vial 22277-CODYTI” (ZSM). 1 female “Ibisca Niugini, PNG 22–24.xi.2012 Wanang -5,227670193 145,0797424”, “FIT-WAN-R-3/8-d06 / Plot 18 / P0686 Vial 16573-CODYTI” (ZSM). 1 female “Ibisca Niugini, PNG 24–26.xi.2012 Wanang -5,227670193 145,0797424”, “FIT-WAN-O-4/8-d08 / Plot 15 / P0663 Vial 17658-CODYTI” (ZSM). 1 male “Ibisca Niugini, PNG 26–28.xi.2012 Wanang -5,227670193 145,0797424”, “FIT-WAN-P-5/8-d10 / Plot 16 / P0672 Vial 22295-CODYTI” (ZSM). 1 female “Ibisca Niugini, PNG 30.xi.-2.xii.2012 Wanang -5,227670193 145,0797424”, “FIT-WAN-O-7/8-d13 / Plot 15 / P0666 Vial 17747-CODYTI” (ZSM). 1 female “Ibisca Niugini, PNG 30.xi.-2.xii.2012 Wanang -5,227670193 145,0797424”, “FIT-WAN-P-7/8-d14 / Plot 16 / P0674 Vial 17730-CODYTI” (ZSM). 1 female “Ibisca Niugini, PNG 30.xi.-2.xii.2012 Wanang -5,227670193 145,0797424”, “FIT-WAN-Q-7/8-d14 / Plot 17 / P0682 Vial 22373-CODYTI” (ZSM). 1 female “Ibisca Niugini, PNG 2–4.xii.2012 Wanang FIT-WAN-D-8/8-d15 / Plot 4 / P0579 Vial 17568-CODYTI” (ZSM).

***East New Britain***: 2 males “Papua New Guinea: East New Britain, Irene, Wouwo, 860 m, 12.ii.2009, 05.60.892S 151.40.782E [coordinates are wrong] Sagata (PNG200)”, one of them with an additional green label “3877” (ZSM). 2 males, 1 female “Papua New Guinea: East New Britain, Wouwo, Pomio, 860 m, 12.ii.2009, 05.44.556S 151.46.44E [coordinates are wrong], Sagata (PNG200)” (ZSM). 11 males, 6 females “Papua New Guinea: East New Britain, Irene, Pomio, 200 m, 9.ii.2009, 05.60.892S 151.40.782E [coordinates are wrong] Sagata (PNG199)”, two of males with additional green labels “3813” and “3814” (NHMW, ZSM). 3 males, 5 females “PNG: E New Britain Prov. 30km SW Kokopo, Arabam, 200 m 04°35'75"S, 152°06'84"E 21.II-04.III.2000 leg. A. Weigel KL” (NHMW, ZSM).

***Austrelatusmirificus* sp. nov.**

**Additional paratypes. *PNG*: *Sandaun***: 1 male “Papua New Guinea: Sandaun, Mianmin, 670 m, 22.x.2008, 4.53.329S 141.35.263E, Ibalim (PNG 189)” (ZSM). 3 males, 3 females “Papua New Guinea: Sandaun, Mianmin (pool), 990 m, 23.x.2008, 4.54.570S 141.35.490E, Ibalim (PNG 193)” (ZSM). 1 female “Papua New Guinea: Sandaun, Mianmin (pool), 1080 m, 24.x.2008, 04.55.780S 141.38.185E, Ibalim (PNG 196)” (ZSM). 2 males, 2 females “Papua New Guinea: Sandaun, Mianmin (river) 700 m, 21.x.2008, 04.52.858S 141.31.706E Ibalim (PNG 197)”, one male with an additional green label “3788” (ZSM). 3 males, 2 females “Papua New Guinea: Sandaun, Mianmin area, >600 m, 13.i.2010, Ibalim & Pius (PNG235)”, one male and female with additional green text labels “6029” and “6030”, respectively (ZSM). 9 males, 9 females “Papua New Guinea: Sandaun, Mianmin area, >600 m, 13.i.2010, Ibalim & Pius (PNG236)” (NHMW, ZSM). 1 male “Papua New Guinea: Sandaun: Sandaun, Sokamin (WB98) 9.x.2003, K. Sagata, MB 689”, “MB689” [green label] (ZSM). 8 males, 4 females “Papua New Guinea: Sandaun, Bewani Stn., stream @ base of Bewani Mts., 200–300 m, 12.iv.2006, nr. 03.05.130S 141.10.227E, Balke & Sagata (PNG 37)”, one male with an additional green label “3229” (NHMW, ZSM). 6 males, 6 females “Papua New Guinea: Sandaun, Bewani Stn., forest puddles @ base of Bewani Mts., 300 m, 12.iv.2006, nr. 03.05.130S 141.10.227E, Balke & Sagata (PNG 38)”, one male with an additional green label “3212” (NHMW, ZSM). 1 female “Papua New Guinea: Sandaun, Road Vanimo-Bewani close coast, 400–480 m, 13.iv.2006, 02.44.372S 141.15.067E, Balke & Sagata (PNG 41)”, “3217” [green label] (ZSM).

***East Sepik***: 1 male “Papua New Guinea: East Sepik, Lembena, 198 m, 3.ix.2009, 04.56.974S 143.56.995E, Ibalim & Pius (PNG241)” (ZSM). 1 female “Papua New Guinea: East Sepik, Lembena, 198 m, 3.ix.2009, 04 46 [!] 974S 143.56.995E, Ibalim & Pius (PNG243)” (ZSM). 1 male “Papua New Guinea: East Sepik, Lembena, 136 m, 3.ix.2009, 04.56.911S 143.56.870E, Ibalim & Pius (PNG244)” (ZSM). 3 males “Papua New Guinea: East Sepik, Lembena, 297 m, 8.ix.2009, 04.57.329S 143.57.297E, Ibalim & Pius (PNG247)”, “6005”, “6006”, “6009” [green text] (ZSM). 1 female “Papua New Guinea: East Sepik, Lembena, 117 m, 8.ix.2009, 04.57.513S 143.57.296E, Ibalim & Pius (PNG248)” (ZSM). 3 males, 1 female “Papua New Guinea: East Sepik, Lembena, 335 m, 10.ix.2009, 04.56.895S 143.59.375E, Ibalim & Pius (PNG250)” (ZSM). 5 males, 4 females “Papua New Guinea: East Sepik, Lembena, 335 m, 10.ix.2009, 04.56.859S 143.57.379E, Ibalim & Pius (PNG251)” (NHMW, ZSM). 3 males, 3 females “Papua New Guinea: East Sepik, Lembena, 335 m, 10.ix.2009, 04.56.921S 143.57.478E, Ibalim & Pius (PNG252)” (NHMW, ZSM). 1 male, 1 female “Papua New Guinea: East Sepik Province, Amboin Patrol Post, Karawari Lodge, 7 Feb.1983, A.C. Messer” (ZSM).

***Madang***: 1 male “Papua New Guinea: Madang, Akameku-Brahmin, Bismarck Range, 750 m, 25.xi.2006, nr 05.49.307S 145.24.389E, Balke & Kinibel (PNG 114)” (ZSM). 5 males, 4 females “Papua New Guinea: Madang, Akameku-Brahmin, Bismarck Range, 250–500 m, 25.xi.2006, nr 05.47.026S 145.24.131E, Balke & Kinibel (PNG 115)” (NHMW, ZSM). 1 male “4172” [green label], “Papua New Guinea: Madang, Wannang, 270 m, 31.x.2008, 05.15.458S 145.02.389E, Posman, (PNG187)” (ZSM). 1 female “Ibisca Niugini, PNG 29–31.x.2012 Mount Wilhelm 200 m -5,739897251 145,3297424 MW0200 / P0750 Vial 07429” (ZSM). 1 female “Ibisca Niugini, PNG 2–4.xi.2012 Mount Wilhelm 200 m -5,739897251 145,3297424 MW0200 / P0744 Vial 09101” (ZSM). 1 male “Ibisca Niugini, PNG 8–10.xi.2012 Mount Wilhelm 200 m -5,739897251 145,3297424 MW0200 / P0763 Vial 14319” (ZSM). 1 female “Ibisca Niugini, PNG 26–28.x.2012 Mount Wilhelm 700 m -5,731960773 145,2521667 MW0700 / P1218 Vial 16217” (ZSM). 1 female “Ibisca Niugini, PNG 27–29.x.2012 Mount Wilhelm 700 m”, “-5,731960773 145,2521667, FIT-MW700-F-2/8-d03 / Plot 6 / P1139 Vial 15944-CODYTI” (ZSM). 1 male, 1 female “Ibisca Niugini, PNG 27–29.x.2012 Mount Wilhelm 700 m”, “-5,731960773 145,2521667, FIT-MW700-A-2/8-d03 / Plot 1 / P1099 Vial 15960-CODYTI” (ZSM). 1 female “Ibisca Niugini, PNG 27–29.x.2012 Mount Wilhelm 700 m -5,731960773 145,2521667”, “FIT-MW700-D-2/8-d03 / Plot 4 / P1123 Vial 15972-CODYTI” (ZSM). 1 female “Ibisca Niugini, PNG 27–29.x.2012 Mount Wilhelm 700 m -5,731960773 145,2521667”, “FIT-MW700-E-2/8-d03 / Plot 5 / P1131 Vial 15937-CODYTI” (ZSM). 1 male, 2 females “Ibisca Niugini, PNG 28–30.x.2012 Mount Wilhelm 700 m -5,731960773 145,2521667 MW700 / P1179 Vial 16186” (ZSM). 1 male “Ibisca Niugini, PNG 28–30.x.2012 Mount Wilhelm 700 m -5,731960773 145,2521667 MW700 / P1235 Vial 16164” (ZSM). 1 female “Ibisca Niugini, PNG 28–30.x.2012 Mount Wilhelm 700 m -5,731960773 145,2521667”, “FIT-MW700-M-2/8-d04 / Plot 13 / P1195 Vial 16167-CODYTI” (ZSM). 1 female “Ibisca Niugini, PNG 30.x.-1.xi.2012 Mount Wilhelm 700 m -5,731960773 145,2521667 MW0700 / P1212 Vial 15970” (ZSM). 1 female “Ibisca Niugini, PNG 30.x-1.xi.2012 Mount Wilhelm 700 m”, “-5,731960773 145,2521667, FIT-MW700-M-3/8-d06 / Plot 13 / P1196 Vial 15980-CODYTI” (ZSM). 1 male, 1 female “Ibisca Niugini, PNG 31.x-2.xi.2012 Mount Wilhelm 700 m”, “-5,731960773 145,2521667, FIT-MW700-D-4/8-d07 / Plot 4 / P1125 Vial 16045-CODYTI” (ZSM). 1 male, 2 females “Ibisca Niugini, PNG 31.x-2.xi.2012 Mount Wilhelm 700 m”, “-5,731960773 145,2521667, FIT-MW700-E-4/8-d07 / Plot 5 / P1133 Vial 15919-CODYTI” (ZSM). 2 males “Ibisca Niugini, PNG 31.x-2.xi.2012 Mount Wilhelm 700 m”, “-5,731960773 145,2521667, FIT-MW700-C-4/8-d07 / Plot 3 / P1117 Vial 15664-CODYTI” (ZSM). 1 female “Ibisca Niugini, PNG 31.x-2.xi.2012 Mount Wilhelm 700 m”, “-5,731960773 145,2521667, FIT-MW700-A-4/8-d07 / Plot 1 / P1101 Vial 15828” (ZSM). 1 male “Ibisca Niugini, PNG 2–4.xi.2012 Mount Wilhelm 700 m”, “-5,731960773 145,2521667 FIT-MW700-D-5/8-d09 / Plot 4 / P1126 Vial 16018-CODYTI” (ZSM). 1 male “Ibisca Niugini, PNG 4–6.xi.2012 Mount Wilhelm 700 m FIT-MW700-D-6/8-d11 / Plot 4 / P1127 Vial 07061” (ZSM). 1 male “Ibisca Niugini, PNG 4–6.xi.2012 Mount Wilhelm 700 m -5,731960773 145,2521667”, “FIT-MW700-E-6/8-d11 / Plot 5 / P1135 Vial 07232-CODYTI” (ZSM). 1 male “Ibisca Niugini, PNG 5–7.xi.2012 Mount Wilhelm 700 m -5,731960773 145,2521667 MW0700 / P1191 Vial 16058” (ZSM). 1 male “Ibisca Niugini, PNG 5–7.xi.2012 Mount Wilhelm 700 m -5,731960773 145,2521667 MW0700 / P1183 Vial 16046” (ZSM). 1 female “Ibisca Niugini, PNG 26–28.x.2012 Mount Wilhelm 1200 m”, “-5,720873833 145,2694702 FIT-MW1200-P-1/8-d02 / Plot 16 / P1608 Vial 18767” (ZSM). 1 female “Ibisca Niugini, PNG 28–30.x.2012 Mount Wilhelm 1200 m -5,720873833 145,2694702 MW1200 / P1585 Vial 18825” (ZSM). 1 female “Ibisca Niugini, PNG 29–31.x.2012 Mount Wilhelm 1200 m”, “-5,720873833 145,2694702 FIT-MW1200-H-3/8-d05 / Plot 8 / P1546 Vial 17249” (ZSM). 1 female “Ibisca Niugini, PNG 1–3.xi.2012 Mount Wilhelm 1200 m -5,720873833 145,2694702 MW1200 / P1571 Vial 16947” (ZSM). 1 female “Ibisca Niugini, PNG 3–5.xi.2012 Mount Wilhelm 1200 m -5,720873833 145,2694702 MW1200 / P1612 Vial 17292” (ZSM). 1 female “Ibisca Niugini, PNG 3–5.xi.2012 Mount Wilhelm 1200 m -5,720873833 145,2694702 MW1200 / P1636 Vial 17324” (ZSM). 2 female “Ibisca Niugini, PNG 4–6.xi.2012 Mount Wilhelm 1200 m”, “-5,720873833 145,2694702 FIT-MW1200-A-6/8-d11 / Plot 1 / P1493 Vial 17274” (ZSM). 1 male “Ibisca Niugini, PNG 9–11.xi.2012 Mount Wilhelm 1200 m,” “-5,720873833 145,2694702 FIT-MW1200-P-8/8-d16 / Plot 16 / P1615 Vial 17012-CODYTI” (ZSM).

***IN*: *Papua*: *Jayapura Regency***: 1 male “Indonesia: Papua, Cyclops Mts., Doyo [2°30'44.6"S, 140°28'58.9"E], 710 m, 27.XI.2007, Riedel.” (ZSM). 1 female “Indonesia: Papua, Cyclops Mts., Doyo, 1120 m, 27.XI.2007, Riedel.” (ZSM). 1 female “Indonesia: Papua, Cyclops Mts., (5), 1120 m, 27.XI.2007, 02°31.516'S 140°30.436'E, A. Riedel leg.” (ZSM). 1 female “Indonesia: Papua, Cyclops Mts., (15), 710 m, 02.XII.2007, 02°32.516'S 140°30.412'E, A. Riedel leg.” (ZSM).

***Yalimo Regency***: 1 male “INDONESIA: West Papua, Jayawijaya Prov., Elelim, 450–750 m, 17.xii.2004, Riedel” (ZSM).

***Sarmi Regency***: 1 male “Indonesia: Papua, Sarmi, Waaf, N Foja Mts, waterfall in forest, 120 m, 23.ix.2014, -2 3317793 138.7500472, UNCEN team (Pap031)” (ZSM).

***Austrelatusneoguineensis* (Zimmermann, 1919)**

**Additional material. *IN*: *Papua*: *Biak Numfor Regency***: 2 males “Indonesia: Papua, Biak, Nimbotong Nimbokramp, 110 m 21.iv.2006, Tindige leg.” and additionally with green labels “3158” and “3159” (ZSM). 7 males, 4 females “Indonesia: Papua, Biak, Nimbotong Nimbokramp, 110 m 21.iv.2006, Tindige ()” (NHMW, ZSM). 1 male “Indonesia: Papua, Biak East, Anggaduben, sealevel, 3.iv.2006, Tindige” (ZSM).

***Yapen Islands Regency***: 5 males “IRAN JAYA: Japen Isl. Mantembu 150–450 m, 18.2.1999 leg. Riedel” (NHMW). 3 females “Indonesia: Papua, Japen Mantembu A.Riedel” (ZSM). 1 male “61” [green label], “Indonesia: Japen Isl., Serui, Mantembu, 16.XII.2000, Riedel, MB 61” (ZSM).

***Mamberano Raya Regency***: 7 males, 6 females “IRAN JAYA: Jayapura Prov. Mamberano, Rouffaer Mts., Noiadi, 150–200 m 17.3.1999, leg. Riedel” (NHMW).

***Sarmi Regency***: 3 males, 2 females “Indonesia: Papua, Sarmi, Waaf, N Foja Mts, waterfall in forest, 120 m, 23.ix.2014, -2 3317793 138.7500472, Menufandu (Pap031)”, one male with an additional green text label “6466” (ZSM). 1 male “Indonesia: Papua, Sarmi area 70 m, 25.ix.2014, -1.971 138.8491, Menufandu (Pap032)” (ZSM).

***Jayapura Regency***: 4 males, 3 females “W.-Neuguinea Cyclops Mts. 4 km nördl. Sentani, 600 m 8.-13.9.1990/ IR 7 leg: Balke & Hendrich” (CLH, ZSM). 1 female “5058” [green text], “Indonesia: Papua, Setani-Maribu, streampools, 115 m, 14.x.2011, 2.30.514S 140.22.828E, UNCEN team (PAP01)” (ZSM).

***Yalimo Regency***: 2 males, (?2 females) “INDONESIA: West Papua, Jayawijaya Prov., Elelim, 450–750 m, 17.xii.2004, Riedel”, one male with an additional green label “1550” (ZSM).

***Pegunungan Bintang Regency***: 16 males, 11 females “IRIAN JAYA 12.8.1992 Zentralmassiv, Borme 140°25'E 04°24'S 900 m, leg. Balke (8)” (NHMW, ZSM). 1 female “IRIAN JAYA Zentralmassiv 140°25'E 04°24'S”, “Kali Eilme, 900 m, 18.8.1992 leg. Balke (16)” (NHMW).

***PNG*: *Sandaun***: 1 male “4198” [green label], “Papua New Guinea: Sandaun, Mianmin, Fak River, 775 m, 13.x.2003, 4 53 53.00S 141 36 39.40E, K. Sagata (WB32)” (ZSM). 2 males “Papua New Guinea: Sandaun, Mianmin 2, 1105 m, 20.x.2003, 04 52 526S 141 37.038E, K. Sagata (WB68)”, with additional green labels “4199” and “MB690” (ZSM). 1 female “Papua New Guinea: Sandaun, Mianmin 2, 1105 m, 20.x.2003, 04 52 526S 141 37.038E, K. Sagata (WB69)” (ZSM). 3 males, 2 females “Papua New Guinea: Sandaun, Sokamin village, 1200 m, 9.x.2003, 4 51.883S 141 37.534E, K. Sagata (WB95)” (ZSM). 1 male “6016” [green text], “Papua New Guinea: Sandaun, Mianmin area, > 700 m, 7.i.2010, Ibalim & Pius (PNG231)” (ZSM). 14 males, 14 females “Papua New Guinea: Sandaun, Mianmin area, >600 m, 13.i.2010, Ibalim & Pius (PNG235)”, two males with additional labels with green text “6031” and “6032” (NHMW, ZSM). 5 males “Papua New Guinea: Sandaun, Mianmin area, > 600 m, 13.i.2010, Ibalim & Pius (PNG236)” (ZSM). 2 males, 3 female “Papua New Guinea: Sandaun, Mianmin area, > 700 m, 14.i.2010, 04 54.540S 141 36.953E, Ibalim & Pius (PNG238)”, the females with additional labels with green text “6011”, “6012”, “6013” (ZSM). 6 males, 1 female “Papua New Guinea: Sandaun, Mianmin, 670 m 22.x.2008, 4.53.329S 141.35.263E, Ibalim (PNG 189)”, one male with additional green label “3774” (ZSM). 13 males, 10 females “Papua New Guinea: Sandaun, Mianmin, 670 m 20.x.2008, 4.53.292S 141.34.118E, Ibalim (PNG 191)” (NHMW, ZSM). 19 males, 8 females “Papua New Guinea: Sandaun, Mianmin (river), 990 m, 23.x.2008, 4.54.570S 141.35.490E, Ibalim (PNG 192)”, one male with additional green label “3785” (NHMW, ZSM). 135 males, 72 females “Papua New Guinea: Sandaun, Mianmin (pool), 990 m, 23.x.2008, 4.54.570S 141.35.490E, Ibalim (PNG 193)”, one male with additional green label “3776” (NHMW, ZSM). 1 male “Papua New Guinea: Sandaun, Mianmin, 820 m, 19.x.2008, 4.54.049S 141.36.823E, Ibalim (PNG 194)” (ZSM). 19 males, 8 females “Papua New Guinea: Sandaun, Mianmin (river), 1080 m, 24.x.2008, 04.55.780S 141.38.185E, S. Ibalim (PNG195)” (NHMW, ZSM). 41 males, 35 females “Papua New Guinea: Sandaun, Mianmin (pool), 1080 m, 24.x.2008, 04.55.780S 141.38.185E, S. Ibalim (PNG 196)”, two males with additional green labels “3769” and “3770” (NHMW, ZSM). 12 males, 11 females “Papua New Guinea: Sandaun, Mianmin, 1080 m, 24.x.2008, 04.55.780S 141.38.185E, S. Ibalim PNG 196” (NHMW, ZSM). 66 males, 21 females “Papua New Guinea: Sandaun, Mianmin (river), 700 m, 21.x.2008, 04.52.858S 141.31.706E, Ibalim (PNG 197)”, one male with additional green label “3787” (NHMW, ZSM). 78 males, 35 females “Papua New Guinea: Sandaun, Mianmin (pool), 700 m, 21.x.2008, 04.52.858S 141.31.706E, Ibalim (PNG 198)” (NHMW, ZSM). 11 males, 7 females “3230” [green label], “Papua New Guinea: Sandaun, Bewani Stn., stream @ base of Bewani Mts., 200–300 m, 12.iv.2006, nr. 03.05.130S 141.10.227E, Balke & Sagata (PNG 37)” (NHMW, ZSM). 13 males, 8 females “Papua New Guinea: Sandaun, Bewani Stn., forest puddles @ base of Bewani Mts., 300 m, 12.iv.2006, nr. 03.05.130S 141.10.227E, Balke & Sagata (PNG 38)”, two males with additional green labels “3213” and “3214” (NHMW, ZSM). 3 females “Papua New Guinea: Sandaun, Road Vanimo-Bewani close coast, 400–480 m, 13.iv.2006, 02.44.372S 141.15.067E, Balke & Sagata (PNG 41)”, two of them additionally with green labels “3218” and “3219” (ZSM). 1 male “3232”, “Papua New Guinea: Sandaun, Toricelli Mts., village below Sibilanga Stn., 400 m, 18.iv.1994, nr 03.26.750S 142.29.949E, Balke (PNG 42)” (ZSM). 10 males, 3 females “Papua New Guinea: Sandaun, Toricelli Mts., 2h walk fr Sibilanga Stn., 350 m, 19.iv.1994, 03.39.121S 142.29.991E, Balke (PNG 44)”, one male with additional green label “3207” (NHMW, ZSM). 1 male “on water. Gathered in sago plantation” [hw], “Terr. Papua & New Guinea: Lumi. 31.x.1957. J. Smart.”, “Brit. Mus 1957-693” (BMNH).

***East Sepik***: 7 males, 20 females “Papua New Guinea East Sepik Province Amboin Patrol Post Karawari Lodge 14Jan. 1983, A.C.Messer” (NHMW, ZSM). 68 males, 42 females “Papua New Guinea East Sepik Province Amboin Patrol Post Karawari Lodge 7 Feb. 1983, A.C.Messer” (NHMW, ZSM). 32 males, 30 females “Papua New Guinea East Sepik Province Amboin Patrol Post Karawari Lodge 14 Feb. 1983, A.C.Messer” (NHMW, ZSM). 56 males, 42 females “Papua New Guinea East Sepik Province Amboin Patrol Post Karawari Lodge 26Mar. 1983, A.C.Messer” (NHMW, ZSM). 5 males, 2 females “Papua New Guinea: East Sepik, Prince Alexander Mts., Wewak-Angoram, 300 m, 21.iv.2006, 03.42.129S 143., Balke (PNG 46)”, two males additionally with green labels “3220” and “3221” (ZSM). 4 males, 1 female “Papua New Guinea: East Sepik, Lembena, 198 m, 3.ix.2009, 04.56.974S 143.56.995E, Ibalim & Pius (PNG241)” (ZSM). 1 male, 4 females “Papua New Guinea: East Sepik, Lembena, 198 m, 3.ix.2009, 04 46 974S 143 56.995E, Ibalim & Pius (PNG243)” (ZSM). 3 males, 6 females “Papua New Guinea: East Sepik, Lembena, 136 m, 3.ix.2009, 04 56.911S 143 56.870E, Ibalim & Pius (PNG244)” (ZSM). 3 males, 2 females “Papua New Guinea: East Sepik, Lembena, 136 m, 3.ix.2009, 04 56.887S 143 56.752E, Ibalim & Pius (PNG245)” (ZSM). 3 females “Papua New Guinea: East Sepik, Lembena, 297 m, 8.ix.2009, 04.57.329S 143.57.297E, Ibalim & Pius (PNG247)” and additional labels with green text “6004”, “6007”, “6008” (ZSM). 19 males, 27 females “Papua New Guinea: East Sepik, Lembena, 117 m, 8.ix.2009, 04.57.513S 143.57.296E, Ibalim & Pius (PNG248)”, one male and four females with additional labels with green text “5995”, “5994”, “5996”, “5997”, “5998” respectively (NHMW, ZSM). 3 males “Papua New Guinea: East Sepik, Lembena, 110 m, 10.ix.2009, 04 57.512S 143 57.366E, Ibalim & Pius (PNG249)” (ZSM). 6 males, 3 females “Papua New Guinea: East Sepik, Lembena, 335 m, 10.ix.2009, 04 56.859S 143 59.375E, Ibalim & Pius (PNG250)” (ZSM). 6 males, 4 females “Papua New Guinea: East Sepik, Lembena, 335 m, 10.ix.2009, 04.56.859S 143.57.379E, Ibalim & Pius (PNG251)” (ZSM). 10 males, 8 females “Papua New Guinea: East Sepik, Lembena, 335 m, 10.ix.2009, 04.56.921S 143.57.478E, Ibalim & Pius (PNG252)” (NHMW, ZSM).

***Madang***: 1 male, 3 females “Papua New Guinea: Madang, Adelbert Mts., Sewan – Keki, 700 m, 04°42'215"S, 145°25'154"E, 4.v.2006, leg Balke & Manaono (PNG 51)” (ZSM). 5 males, 5 females “Papua New Guinea: Madang, Adalbert [sic!] Mts., Keki, 850 m, 4.v.2006, nr 04.42.300S 145.25.089E, Balke & Manaono (PNG 52)” (NHMW, ZSM). 1 male “Papua New Guinea: Madang, Adalbert [sic!] Mts., below Keki, 790 m, 5.v.2006, 04.42.300S 145.25.089E, Balke & Manaono (PNG 53)” (ZSM). 1 male “Papua New Guinea: Madang, Adalbert [sic!] Mts., creek nr Keki, 790 m, 26.xi.2006, 04.42.300S 145.25.089E, Binatang Boys leg. (PNG 53a)” (ZSM). 32 male, 26 females “Papua New Guinea: Madang, Adelbert Mts., Keki to Sewan, 650 m, 7.v.2006, 04.41.802S 145.25.460E, Balke (PNG 54)”, one male with an additional green label “3203” (NHMW, ZSM). 5 males, 2 females “Papua New Guinea: Madang, Adalbert [sic!] Mts., 400 m, 29.xi.2006, 04.43.058S 145.24.437E, Binatang Boys (PNG 119)” (ZSM). 5 males, 4 females “Papua New Guinea: Madang, Keki – Sewan, Adalbert [sic!] Mts., 700 m, 30.xi.2006, nr 04.41.802S 145.25.460E, Binatang Boys (PNG 120)” (ZSM). 4 females “Papua New Guinea: Wanang, village, 20.ix.2013, Boukal, 13/2013” (ZSM). 6 males, 8 females “Papua New Guinea: Wanang, Digitam Tributary, 23.ix.2013, Boukal, 33/2013” (NHMW, ZSM). 1 male, 2 females “Papua New Guinea: Wanang area, Digitam creek, 23.ix.2013, Boukal, 38+37+?36/2013” (ZSM). 1 male “Papua New Guinea: Digitam creek, smallside pools, 23.ix.2013, Boukal, 37/2013” (ZSM). 1 male “PNG: Madang, Wanang vill. env., Wanang cons.area, nr Wanang Swire field St. Digitam Creek 23.ix.2013, leg. Boukal” [hw, H.Shaverdo], “6444” [green text] (ZSM). 4 males, 4 females “Papua New Guinea: Wanang, Kusa, 24.ix.2013, Boukal, 39/2013” (NHMW, ZSM). 2 males, 2 females “Papua New Guinea: Wanang I, river, 25.ix.2013, Boukal, 44/2013” (NHMW, ZSM). 2 males, 1 female “Papua New Guinea: Madang, Wannang, 270 m, 31.x.2008, 05.15.458S 145.02.389E, Posman, (PNG187)”, one male and female additionally with green labels “4170” and “3791” respectively (ZSM). 17 males, 10 females “Papua New Guinea: Madang, Wannang, 230 m 3.x.2008, 05.17.235S 145.06.160E, Posman (PNG188)”, one of males additionally with a green label “3764” (NHMW, ZSM). 2 males, 1 female “PNG Madang Province OHU Village, 14.1.2001 leg.: Lukas Cizek Coll. HENDRICH” (ZSM). 3 females “Papua New Guinea: Madang, Brahmin, 150 m, 26IX2002, 05.44.953S 145.20.844E Balke & Sagata (PNG 024)” (ZSM). 16 males, 18 females “Papua New Guinea: Madang, Akameku-Brahmin, Bismarck Range, 250–500 m, 25.xi.2006, nr 05.47.026S 145.24.131E, Balke & Kinibel (PNG 115)” (NHMW, ZSM). 1 male “Papua New Guinea: Madang, nr. Brahmin, 200 m, 25.xi.1994, nr. 05.47.026S 145.24.131E, Balke & Kinibel (PNG 116)” (ZSM). 8 males, 1 female “Papua New Guinea: Madang, Simbai – Aiome, 980 m, 11.iii.2007, 05.12.241S 144.42.553E, Kinibel (PNG 155)” (NHMW, ZSM). 1 male, 4 females “Papua New Guinea: Madang, Usino, 260 m, 15.iii.2007, 05.31.125S 145.25.316E, Kinibel (PNG 158)” (ZSM). 1 male, 3 females “Papua New Guinea: Madang, below Bundi, 500 m, 26.IX.2002 Balke & Sagata (PNG 023)”, one male and female additionally with green labels “270” and “271”, respectively (ZSM). 2 females “Ibisca Niugini, PNG 11.ii-11.iv.2012 Mount Wilhelm 200 m -5,739897251 145,3297424 MW0200 / P0760 Vial 07137” (MNHN, ZSM). 1 female “Ibisca Niugini, PNG 11.v.-11.vii.2012 Mount Wilhelm 200 m -5,739897251 145,3297424 MW0200 / P0817 Vial 9583” (MNHN). 1 male “Ibisca Niugini, PNG 11.ix.-11.xi.2012 Mount Wilhelm 200 m -5,739897251 145,3297424 MW0200 / P0803 Vial 14334” (MNHN). 1 female “Ibisca Niugini, PNG 25–27.x.2012 Mount Wilhelm 200 m -5,739897251 145,3297424 MW0200 / P0756 Vial 07480” (ZSM). 1 female “Ibisca Niugini, PNG 26–28.x.2012 Mount Wilhelm 200 m”, “-5,739897251 145,3297424 FIT-MW200-M-1/8-d02 / Plot 13 / P0804 Vial 07211” (ZSM). 1 female “Ibisca Niugini, PNG 28–30.x.2012 Mount Wilhelm 200 m”, “-5,739897251 145,3297424 FIT-MW200-R-2/8-d04 / Plot 18 / P0845 Vial 07491-CODYTI” (ZSM). 4 females “Ibisca Niugini, PNG 1–3.xi.2012 Mount Wilhelm 200 m -5,739897251 145,3297424 MW0200 / P0847 Vial 14645” (MNHN, ZSM). 2 females “Ibisca Niugini, PNG 1–3.xi.2012 Mount Wilhelm 200 m -5,739897251 145,3297424 MW0200 / P0855 Vial 14628” (MNHN, ZSM). 1 female “Ibisca Niugini, PNG 1–3.xi.2012 Mount Wilhelm 200 m -5,739897251 145,3297424 MW0200 / P0799 Vial 14694” (ZSM). 1 female “Ibisca Niugini, PNG 26–28.x.2012 Mount Wilhelm 200 m”, “-5,739897251 145,3297424 FIT-MW200-K-4/8-d08 / Plot 11 / P0791 Vial 14641” (ZSM). 1 female “Ibisca Niugini, PNG 3–5.xi.2012 Mount Wilhelm 200 m”, “-5,739897251 145,3297424 FIT-MW200-Q-5/8-d10 / Plot 17 / P0840 Vial 09663-CODYTI” (ZSM). 1 female “Ibisca Niugini, PNG 3–5.xi.2012 Mount Wilhelm 200 m”, “-5,739897251 145,3297424 FIT-MW200-T-5/8-d10 / Plot 20 / P0864 Vial 09550-CODYTI” (ZSM). 2 females “Ibisca Niugini, PNG 3–5.xi.2012 Mount Wilhelm 200 m -5,739897251 145,3297424 MW0200 / P0848 Vial 09529” (ZSM). 2 females “Ibisca Niugini, PNG 4–6.xi.2012 Mount Wilhelm 200 m”, “-5,739897251 145,3297424 FIT-MW200-G-6/8-d11 / Plot 7 / P0761 Vial 07191” (ZSM). 1 male, 1 female “Ibisca Niugini, PNG 5–7.xi.2012 Mount Wilhelm 200 m -5,739897251 145,3297424 MW0200 / P0865 Vial 09576” (MNHN, ZSM). 1 female “Ibisca Niugini, PNG 6–8.xi.2012 Mount Wilhelm 200 m”, “-5,739897251 145,3297424 FIT-MW200-G-7/8-d13 / Plot 7 / P0762 Vial 09608” (ZSM). 1 female “Ibisca Niugini, PNG 7–9.xi.2012 Mount Wilhelm 200 m”, “-5,739897251 145,3297424 FIT-MW200-R-7/8-d14 / Plot 18 / P0850 Vial 07413-CODYTI” (ZSM). 1 female “Ibisca Niugini, PNG 7–9.xi.2012 Mount Wilhelm 200 m -5,739897251 145,3297424 MW0200 / P0850 Vial 07413” (ZSM). 1 female “Ibisca Niugini, PNG 7–9.xi.2012 Mount Wilhelm 200 m -5,739897251 145,3297424 MW0200 / P0842 Vial 07490” (ZSM). 1 male “Ibisca Niugini, PNG 7–9.xi.2012 Mount Wilhelm 200 m -5,739897251 145,3297424 MW0200 / P0834 Vial 09532” (ZSM). 2 males, 4 females “Ibisca Niugini, PNG 9–11.xi.2012 Mount Wilhelm 200 m”, “-5,739897251 145,3297424 FIT-MW200-R-8/8-d16 / Plot 18 / P0851 Vial 14373-CODYTI” (ZSM). 1 female “Ibisca Niugini, PNG 9–11.xi.2012 Mount Wilhelm 200 m”, “-5,739897251 145,3297424 FIT-MW200-S-8/8-d16 / Plot 19 / P0859 Vial 14056-CODYTI” (ZSM). 1 female “Ibisca Niugini, PNG 9–11.xi.2012 Mount Wilhelm 200 m”, “-5,739897251 145,3297424 FIT-MW200-P-8/8-d16 / Plot 16 / P0835 Vial 14281-CODYTI” (ZSM). 1 female “Ibisca Niugini, PNG 9–11.xi.2012 Mount Wilhelm 200 m”, “-5,739897251 145,3297424 FIT-MW200-T-8/8-d16 / Plot 20 / P0867 Vial 14305-CODYTI” (ZSM). 1 female “Ibisca Niugini, PNG 25–27.x.2012 Mount Wilhelm 700 m”, “-5,731960773 145,2521667 FIT-MW0700-C-1/8-d01 / Plot 3 / P1114 Vial 16039-CODYTI” (ZSM). 1 male “Ibisca Niugini, PNG 25–27.x.2012 Mount Wilhelm 700 m”, “-5,731960773 145,2521667 FIT-MW0700-E-1/8-d01 / Plot 5 / P1130 Vial 15914-CODYTI” (ZSM). 1 female “Ibisca Niugini, PNG 26–28.x.2012 Mount Wilhelm 700 m -5,731960773 145,2521667 MW0700 / P1178 Vial 16181” (ZSM). 2 males, 1 female “Ibisca Niugini, PNG 28–30.x.2012 Mount Wilhelm 700 m -5,731960773 145,2521667 MW0700 / P1179 Vial 16186” (ZSM). 1 female “Ibisca Niugini, PNG 27–29.x.2012 Mount Wilhelm 700 m”, “-5,731960773 145,2521667 FIT-MW0700-D-2/8-d03 / Plot 4 / P1123 Vial 15972-CODYTI” (ZSM). 2 females “Ibisca Niugini, PNG 27–29.x.2012 Mount Wilhelm 700 m”, “-5,731960773 145,2521667 FIT-MW0700-A-2/8-d03 / Plot 1 / P1099 Vial 15960-CODYTI” (MNHN, ZSM). 1 male, 2 females “Ibisca Niugini, PNG 28–30.x.2012 Mount Wilhelm 700 m -5,731960773 145,2521667 MW0700 / P1235 Vial 16164” (ZSM). 2 females “Ibisca Niugini, PNG 28–30.x.2012 Mount Wilhelm 700 m -5,731960773 145,2521667 MW0700 / P1243 Vial 16156” (ZSM). 1 male, 2 females “Ibisca Niugini, PNG 28–30.x.2012 Mount Wilhelm 700 m -5,731960773 145,2521667 MW0700 / P1179 Vial 16186” (ZSM). 1 female “Ibisca Niugini, PNG 28–30.x.2012 Mount Wilhelm 700 m -5,731960773 145,2521667 MW0700 / P1227 Vial 16277” (ZSM). 1 female “Ibisca Niugini, PNG 29–31.x.2012 Mount Wilhelm 700 m”, “-5,731960773 145,2521667 FIT-MW0700-C-3/8-d01 / Plot 3 / P1116 Vial 07314-CODYTI” (ZSM). 1 male “Ibisca Niugini, PNG 1–3.xi.2012 Mount Wilhelm 700 m -5,731960773 145,2521667 MW0700 / P1213 Vial 16236” (ZSM). 1 male, 2 females “Ibisca Niugini, PNG 3–5.xi.2012 Mount Wilhelm 700 m -5,731960773 145,2521667 MW0700 / P1214 Vial 16090” (ZSM). 1 female “Ibisca Niugini, PNG 3–5.xi.2012 Mount Wilhelm 700 m -5,731960773 145,2521667 MW0700 / P1222 Vial 16098” (ZSM). 1 female “Ibisca Niugini, PNG 3–5.xi.2012 Mount Wilhelm 700 m”, “-5,731960773 145,2521667 MW0700-R-5/8-d10 / Plot 18 / P1238 Vial 15969-CODYTI” (ZSM). 1 female “Ibisca Niugini, PNG 4–6.xi.2012 Mount Wilhelm 700 m”, “-5,731960773 145,2521667 MW0700-F-6/8-d11 / Plot 6 / P1143 Vial 07287” (ZSM). 3 females “Ibisca Niugini, PNG 4–6.xi.2012 Mount Wilhelm 700 m -5,731960773 145,2521667”, “FIT-MW700-E-6/8-d11 / Plot 5 / P1135 Vial 07232-CODYTI” (MNHN, ZSM). 1 male “Ibisca Niugini, PNG 4–6.xi.2012 Mount Wilhelm 700 m FIT-MW700-D-6/8-d11 / Plot 4 / P1127 Vial 07061” (ZSM). 1 male “Ibisca Niugini, PNG 5–7.xi.2012 Mount Wilhelm 700 m -5,731960773 145,2521667 MW0700 / P1183 Vial 16046” (ZSM). 1 male “Ibisca Niugini, PNG 6–8.xi.2012 Mount Wilhelm 700 m -5,731960773 145,2521667 MW0700 / P1144 Vial 15649” (ZSM). 1 female “Ibisca Niugini, PNG 6–8.xi.2012 Mount Wilhelm 700 m”, “-5,731960773 145,2521667 MW0700-C-7/8-d13 / Plot 3 / P1120 Vial 15881” (ZSM). 1 female “Ibisca Niugini, PNG 9–11.xi.2012 Mount Wilhelm 700 m -5,731960773 145,2521667 MW0700 / P1217 Vial 16106” (ZSM). 1 female “Ibisca Niugini, PNG 25–27.x.2012 Mount Wilhelm 1200 m -5,720873833 145,2694702 MW1200 / P1512 Vial 18820” (ZSM). 1 female “Ibisca Niugini, PNG 27–29.x.2012 Mount Wilhelm 1200 m -5,720873833 145,2694702 MW1200 / P1561 Vial 16873” (ZSM). 1 female “Ibisca Niugini, PNG 28–30.x.2012 Mount Wilhelm 1200 m -5,720873833 145,2694702 MW1200 / P1593 Vial 17462” (ZSM). 1 female “Ibisca Niugini, PNG 28–30.x.2012 Mount Wilhelm 1200 m -5,720873833 145,2694702 MW1200 / P1577 Vial 18802” (ZSM). 1 female “Ibisca Niugini, PNG 1–3.xi.2012 Mount Wilhelm 1200 m -5,720873833 145,2694702 MW1200 / P1595 Vial 18799” (ZSM). 1 female “Ibisca Niugini, PNG 30.xi.-2.xii.2012 Mount Wilhelm 1700 m -5,227670193 145,0797424”, “FIT-WAN-F-7/8-d13 / Plot 6 / P0594 Vial 17790-CODYTI” (ZSM). 1 female “Ibisca Niugini, PNG 20–22.xi.2012 Wanang -5,227670193 145,0797424”, “FIT-WAN-K-2/8-d04 / Plot 11 / P0629 Vial 22254-CODYTI” (ZSM). 1 female “Ibisca Niugini, PNG 22–24.xi.2012 Wanang -5,227670193 145,0797424”, “FIT-WAN-G-3/8-d05 / Plot 7 / P0598 Vial 07876-CODYTI” (ZSM). 1 male, 1 female “Ibisca Niugini, PNG 24–26.xi.2012 Wanang -5,227670193 145,0797424”, “FIT-WAN-J-4/8-d07 / Plot 10 / P0623 Vial 49903-CODYTI” (ZSM). 1 female “Ibisca Niugini, PNG 24–26.xi.2012 Wanang -5,227670193 145,0797424”, “FIT-WAN-D-4/8-d07 / Plot 4 / P0575 Vial 02451-CODYTI” (ZSM). 1 female “Ibisca Niugini, PNG 26–28.xi.2012 Wanang -5,227670193 145,0797424”, “FIT-WAN-D-5/8-d09 / Plot 4 / P0576 Vial 17685-CODYTI” (ZSM). 1 male “Ibisca Niugini, PNG 26–28.xi.2012 Wanang -5,227670193 145,0797424”, “FIT-WAN-J-5/8-d09 / Plot 10 / P06246 Vial 17673-CODYTI” (ZSM). 1 female “Ibisca Niugini, PNG 30.xi.-2.xii.2012 Wanang -5,227670193 145,0797424”, “FIT-WAN-K-7/8-d14 / Plot 11 / P0634 Vial 17738-CODYTI” (ZSM). 1 male “Ibisca Niugini, PNG 30.xi.-2.xii.2012 Wanang -5,227670193 145,0797424”, “FIT-WAN-J-7/8-d13 / Plot 10 / P0626 Vial 17492-CODYTI” (ZSM). 1 male “Ibisca Niugini, PNG 2–4.xii.2012 Wanang FIT-WAN-D-8/8-d15 / Plot 4 / P0579 Vial 17568-CODYTI” (ZSM).

***WHL***: 1 male, 5 females “Papua New Guinea: Western Highlands, Jimi Valley, Sendiap Station, 500–600 m, 5.iii.2007, 05.20.728S 144.27.401E, Kinibel (PNG 146)”, one male and one female additionally with green labels “2869” and “2870” respectively (ZSM). 1 male, 4 females “Papua New Guinea: Western Highlands, Jimi Valley, above Sendiap Station, 950 m, 6.iii.2007, 05.20.587S 144.28.847E, Kinibel (PNG 147)”, one male and one female additionally with green labels “4279” and “4278” respectively (ZSM).

***EHL***: 2 males “Papua New Guinea: Eastern Highlands, Bena – pass to Goroka valley, 1550 m, 5.iv.2006, 06.14.567S 145.29.634E, Balke & Sagata (PNG33)” (ZSM). 5 males, 5 females “Papua New Guinea: Eastern Highlands, below Yonki, 850 m, 4.iv.2006, 06.11.332S 146.03.052E, Balke & Sagata (PNG31)” (NHMW, ZSM).

***Morobe***: 1 female “3209” [green label], “Papua New Guinea: Morobe, Huon Pen., rd to Kwapsanek, 460 m, 31.iii.2006, 06.32.736S 146.59.616E, Balke & Sagata (PNG 26)” (ZSM). 3 males, 1 female “Papua New Guinea: Morobe, Sattelberg, Siki River, ca. 700 m, 20.x.2009,6.29.352S 147.46.544E, Idaho (12c) (PNG214)” (NHMW, ZSM). 2 females “Papua New Guinea: Morobe, Sattelberg, Zige River, ca. 700 m, x.2009, 6.29.233S 147.46.482E, Idaho (12a) (PNG212)”, one female additionally with a green label “3824” (ZSM). 2 males, 1 female “Papua New Guinea: Morobe, Sattelberg, Zige River, 970 m, 20.x.2009, 6.29.233S 147.46.482E, Idaho (11) (PNG211)”, one male additionally with a green label “3829” (ZSM). 30 males, 25 females “Papua New Guinea: Morobe, Herzog Mts., Bundun, 700–800 m, 2.iv.2006, 06.51.598S 146.37.07E, Balke & Sagata (PNG 27)”, three males with additional green labels “3200”, “3201” and “3227” (NHMW, ZSM).

***Austrelatusrajaampatensis* sp. nov.**

**Additional paratypes: *IN*: *West Papua*: *Raja Ampat Regency*: *Batanta***: 7 males, 5 females “W-PAPUA Raja Ampat Prov. Batanta Isl. bor., 9 km W Yensawai, Ross-River 0°49'23"S 130°35'52"E 17.I.2004 leg. A.Skale” (CAS, NHMW). 1 male, 1 female “Indonesia: Papua, Batanta Selatan, Wailebet, 100 m, 17.ii.2006, inland 00.53.957S 130.39.951E, Tindige & Prativi (BH 15)” (ZSM).

***Waigeo***: 2 males “W-PAPUA Raja Ampat Prov. Waigeo Isl., Lopintol, Balon-River, 0°07'S, 130°53'E 11.I.2004 leg. A.Skale,” (CAS). 5 males, 4 females “W-PAPUA Raja Ampat Prov. Waigeo Isl., Lopintol 0°07'54"S, 130°53'45"E 11.i.2004 leg. A.Skale UWP” (CAS, NHMW). 1 female “MB761” [green label], “W Papua: Raja Ampat, Waigeo, Lopintol, 11/2005, A. Skale,” (ZSM). 1 male “Indonesia: Papua, Waigeo, Waifoi, <50 m 10.ii.2006, 00.06.088S 130.42.855E, Tindige (BH 10)” (ZSM). 2 males, 1 female “Irian Jaya: Sorong Prov., Waigeo Isl., Kabui Bay, Wawiay, 14–15.11.1996, 0–250 m. leg. A. Riedel” (NHMW).

***Salawati***: 1 male, 3 females “W-PAPUA Raja Ampat Prov. Salawati Isl. or., 2–4 km N Kalobo 01°00'56"S, 131°04'58"E 26.I.2004 leg. A.Skale” (CAS). 1 male, 1 female “Indonesia: Papua, Salawati Utara, 100–250 m, 18.ii.2006, 00.57.954S 130.40.531E, Tindige (BH 18)” (ZSM).

***Sorong Regency***: 1 male, 2 females “Indonesia: West Papua, Malawor 50 m, 28.i.2001 Riedel leg.” (ZSM). 2 males “Indonesia: Papua Barat, Sorong-Teminabuan, 50 m, 2.x.2014, -1,1092904 131,6125645, Sumoked (BH046)”, with additional green text labels “6451” and “6452” (ZSM). 4 males, 12 females “Indonesia: Papua Barat, Sorong-Teminabuan, 50 m, 2.x.2014, Sumoked (BH046) -1,1092904 131,6125645” (ZSM). 1 female “6489”, “Indonesia: Papua Barat, Sorong-Teminabuan, 130 m, 2.x.2014, -1,1357267 131,9000149, Sumoked (BH047)” (ZSM).
